# A Multi-Omic View of Host-Pathogen-Commensal Interplay in *Salmonella*-Mediated Intestinal Infection

**DOI:** 10.1371/journal.pone.0067155

**Published:** 2013-06-26

**Authors:** Brooke L. Deatherage Kaiser, Jie Li, James A. Sanford, Young-Mo Kim, Scott R. Kronewitter, Marcus B. Jones, Christine T. Peterson, Scott N. Peterson, Bryan C. Frank, Samuel O. Purvine, Joseph N. Brown, Thomas O. Metz, Richard D. Smith, Fred Heffron, Joshua N. Adkins

**Affiliations:** 1 Biological Sciences Division, Pacific Northwest National Laboratory, Richland, Washington, United States of America; 2 Department of Molecular Microbiology and Immunology, Oregon Health and Science University, Portland, Oregon, United States of America; 3 Department of Infectious Diseases, J. Craig Venter Institute, Rockville, Maryland, United States of America; 4 Environmental Molecular Sciences Laboratory, Pacific Northwest National Laboratory, Richland, Washington, United States of America; Charité-University Medicine Berlin, Germany

## Abstract

The potential for commensal microorganisms indigenous to a host (the ‘microbiome’ or ‘microbiota’) to alter infection outcome by influencing host-pathogen interplay is largely unknown. We used a multi-omics “systems” approach, incorporating proteomics, metabolomics, glycomics, and metagenomics, to explore the molecular interplay between the murine host, the pathogen *Salmonella enterica* serovar Typhimurium (*S*. Typhimurium), and commensal gut microorganisms during intestinal infection with *S*. Typhimurium. We find proteomic evidence that *S.* Typhimurium thrives within the infected 129/SvJ mouse gut without antibiotic pre-treatment, inducing inflammation and disrupting the intestinal microbiome (e.g., suppressing *Bacteroidetes* and *Firmicutes* while promoting growth of *Salmonella* and *Enterococcus*). Alteration of the host microbiome population structure was highly correlated with gut environmental changes, including the accumulation of metabolites normally consumed by commensal microbiota. Finally, the less characterized phase of *S.* Typhimurium’s lifecycle was investigated, and both proteomic and glycomic evidence suggests *S.* Typhimurium may take advantage of increased fucose moieties to metabolize fucose while growing in the gut. The application of multiple omics measurements to *Salmonella*-induced intestinal inflammation provides insights into complex molecular strategies employed during pathogenesis between host, pathogen, and the microbiome.

## Introduction

Non-typhoidal Salmonellosis, characterized by gastroenteritis in immunocompetent humans, is an increasing health burden in the United States and throughout the world [1]. While mouse models of *Salmonella enterica* serovar Typhimurium (*S.* Typhimurium) infection have been widely used and are invaluable in the study of host-pathogen interactions, *Salmonella*-infected mice typically develop systemic, typhoid-like disease, which limits their applicability for studying the gastrointestinal phase of illness [2]. Because of these model limitations, a significant gap exists in our understanding of pathogenic mechanisms during *Salmonella*-induced gastroenteritis, the role of commensal microbiota in the gut, and the host response to infection.

Commensal microorganisms, or “microbiota,” are acquired shortly after birth and execute important functions in the host, including maturation of the gut associated lymphoid tissue (GALT; [3]), energy harvesting [4], [5], inhibition of pro-inflammatory responses against the commensal organism population [3], and a myriad of other metabolic processes. A significant role of the microbiota is to protect the host from a variety of pathogens, a characteristic referred to as “colonization resistance.” While the mechanisms by which this occurs are largely unknown and likely include multiple strategies [6], [7], recent studies have demonstrated increased susceptibility to infection in animals with altered microbiota (such as germ-free, gnotobiotic, or antibiotic treated animals) [8]. Because investigation of *Salmonella*’s lifestyle in the mouse gut has relied primarily on the use of antibiotic treatment prior to oral inoculation [2], [9], effectively disrupting the commensal microbiota, few studies have addressed the dynamic molecular interplay of *Salmonella* and an intact, natural microbiota during gastrointestinal infection [10]. Understanding the influence of the intact microbiota [7], [8] during infection has been further complicated by multiple factors: the complexity of microbial communities, knowledge of their composition and growth requirements, and the difficulty in gaining a global view of microbe-microbe and microbe-host interactions.

To study the complex interactions during *Salmonella*-induced gastroenteritis, we utilized a mouse model of persistent *S*. Typhimurium infection [11]. In the 129/SvJ mouse model, gastrointestinal *S*. Typhimurium infection occurs without antibiotic pre-treatment, allowing *Salmonella,* commensal organisms, and host factors to be shed in the feces throughout infection, and providing a non-invasive window to the course of gastrointestinal infection through time ([11], this study). Our results demonstrate that during intestinal infection in the 129/SvJ mouse, *S*. Typhimurium induces and then thrives in the inflammatory gut environment, while the population of commensal organisms is effectively reduced. Concurrent with the shift in microbial community structure, characterized by decreased *Barnesiella* and outgrowth of *Salmonella* and Enterococci, we observed alterations in metabolites present in the gut, including an accumulation of sugars normally metabolized by commensal organisms. Finally, our data suggest *S*. Typhimurium may respond to increased fucosylated glycans in the infected gut and consume fucose, providing more insight into the gastrointestinal lifestyle of *S*. Typhimurium. Utilization of an appropriate animal model and “pan-omics” technologies (a union of proteomics, metabolomics, glycomics, and genomics) has facilitated global analyses of the complex molecular strategies employed during pathogenesis.

## Materials and Methods

### Bacterial Strains and Culture Conditions

*Salmonella enterica* serovar Typhimurium (ATCC 14028s) was used as the wild-type strain for mouse infections. Bacteria were grown overnight at 37°C with constant shaking. Prior to mouse infection, the bacteria were washed with PBS, and serially diluted to achieve a concentration of 10^9^ CFU/ml based on the optical density at 600 nm. Bacterial stock was kept on ice before infection. After infection, the same bacterial stock was plated to LB agar to verify CFU accuracy.

The fuc gene regulon (including *fucO, fucA*, STM14_3588, *fucP, fucI, fucK, fucU*, and *fucR* genes) deletion strain was constructed in the parent WT strain *S*. Typhimurium 14028s. The λ Red recombination system was employed twice to delete the gene cluster as described previously [12]. Linearized PCR products containing a kanamycin resistance cassette (kan) and flanking 40-nt sequences homologous to fucR gene were introduced into recipient cells to replace *fucR*. FLP recombinase produced from pCP20 eliminated the antibiotic resistance gene via site-specific recombination [12] and resulted in in-frame and non-polar deletions of *fucR*. The second round of λ Red recombination was conducted at the *fucO* gene, creating three FRT (FLP recognition target) sites in the strain. Following multiple site-specific recombination events, the kan cassette was removed. Deletion of the whole fuc cluster was confirmed by colony PCR using primers located upstream of *fucO* and downstream of *fucR*. Primer sequences used for strain construction were as follows: 1) fucR-RF1 (for ΔfucR) G CCG CGC AGT AGT CGC GGC CTA CTG CGT GCG AAT AGG CCA ATT CCG GGG ATC CGT CGA, 2) fucR-RR1 (for ΔfucR) G CGT ACT GCG CCC GCA AGT AGC TTA CTA CCC CAC TCT TTT GTG TAG GCT GGA GCT GCT, 3) fucO-RF1 (for ΔfucO) GCA GCG CCA TCC GGC AAA ATT ACC AGG CGGTAT GAT ACA GATTCC GGG GAT CCGTCG A, 4) fucO-RR1 (for ΔfucO) GGA TTA CGT ATT GAA GAGTGA TGT CAG GAG ACA GGC GAT GGT GTA GGCTGG AGCTGCT, 5) fuc-DF1 (for Δfuc colony PCR) GCC TAC AGG ACT GCA CGA AAT G, and 6) fuc-DR1 (for Δfuc colony PCR) GAA GAG GTG TCG CAT AAC CTG G.

### Mouse Infections

6- to 8-week-old female 129/SvJ mice (Jackson Laboratories, Bar Harbor, ME) were used for oral infections. Mouse experiments in this study were performed in accordance with the Guide for the Care and Use of Laboratory Animals. The animal protocol was approved by the Oregon Health & Science University Institutional Animal Care and Use Committee (OHSU IACUC) under permit number A085/2011. All efforts were made to minimize animal suffering during the experiments. Animals were euthanized by carbon dioxide asphyxiation followed by cervical dislocation, either at the time point determined by the experiment or when they appeared moribund. The protocol from reference [11] was followed. Briefly, food was withheld from all mice overnight prior to infection. A final inoculum of 1.6×10^8^ CFU *S.* Typhimurium/mouse was delivered by oral gavage to 10 mice (two cages of five mice each = *Salmonella*-infected). An equal number of mock-infected animals (two cages of five mice each = control) were administered an equal volume of sterile saline. Our infecting dose (10^8^ CFU/mouse) aimed to establish a persistent infection [11] that would ensure observation of *S*. Typhimurium proteins in downstream analyses. On the day prior to infection, and at seven time points post-infection, mice from one cage were transferred to a clean cage and fecal samples produced at that time were collected, pooled, and immediately frozen at −80°C. Samples were collected on days −1, 1, 3, 6, 10, 14, 21, and 28. On day 28, mice were sacrificed as described above, and colonization of spleen and mesenteric lymph node were determined (*Salmonella* infected mice: 2.1–7.3×10^3^ CFU/spleen, 2.2–5.0×10^3^ CFU/MLN; mock-infected mice: not detected). Mouse deaths prior to day 28 occurred as follows (one death per day listed): experimental group 1 deaths on days 7, 25, and 26; experimental group 2 deaths on days 16, 17, 20, and 25. Incidence of mouse death was more frequent than previously documented [11], which was possibly due to subtle differences between the virulent strain *S.* Typhimurium 14028s (used here) and SL1344 (used in [11]). We used the 14028s strain because the genome sequence of this organism, but not SL1344, was available at the time for accurate proteomic analysis [13].

For competitive index measurements, three groups of 129/SvJ mice (3 mice/group) from the Jackson Laboratory were starved for 18 hours before oral inoculation. MA6054 was the WT reference strain that produces an arabinose-inducible β-galactosidase and can be distinguished from the test strain (Δfuc) on LB agar containing X-Gal (5-bromo-4-chloro-3-indolyl-D-galactopyranoside; 40 µg/ml) and arabinose (1 mM) [14]. The Δfuc and MA6054 strains were cultured individually in LB overnight prior to infection. Each strain was washed and diluted in PBS to 1×10^8^ CFU/ml. The two strains were mixed at a ratio of 1∶1, and 100 µl (10^7^ CFU/mouse) of the mixture was used to orally infect the mice. The infecting inoculum was serially diluted in PBS and spread on LB agar plates with X-Gal and arabinose to determine the infecting dose of each strain. Fecal pellets were collected at the following time points and pooled by group: day −1, 1, 3, 6, 10, and 14 post-infection. For each time point, four fecal pellets were collected within 1 hour time period, and diluted in PBS before plating on *Salmonella-Shigella* selective agar plates. One hundred black colonies were picked from *Salmonella-Shigella* plates and sub-cultured on LB agar plates with X-Gal and arabinose. After overnight incubation at 37°C, colonies of the reference strain were blue and those of the test strain were white. The competitive index was then calculated as [number of test strains/number of reference strains] output/[number of test strains/number of reference strains] input [15].

### Fecal Sample Preparation

Frozen fecal samples were thawed on ice, and starting material weight was measured. Samples ranged from four to 25 fecal pellets per pooled sample. After thawing, 150 mM ammonium bicarbonate buffer was added to the sample (between 1–2.5 ml based upon starting weight; volumes were recorded and used for downstream normalization), which was subsequently vortexed to disrupt fecal pellets. The resulting slurry was filtered through a 70 µm sieve to separate and remove large debris (mostly undigested food particles). Filtrate was centrifuged (900×g for 10 min), and the protein-rich pellet thought to contain cellular material was retained as “P1.” The supernatant was centrifuged to further clarify the sample (15,000×g for 10 min). The pellet was retained as “P2” and the supernatant retained as “SN2.” Preliminary analyses demonstrated that P2 samples contained prohibitively small protein yields; therefore, P2 samples were not investigated further. Proteomic and 16s rDNA sequencing analyses were performed using P1, and metabolite and glycan analyses were performed using SN2.

### Protein Extraction and Digestion

P1 samples (see Fecal sample preparation) were resuspended in lysis buffer (25 mM Tris-HCl, 5 mM EDTA, 0.05% Triton X-100, 50 µg/ml lysozyme, 3.3 U/ml mutanolysin) and incubated on ice for 15 min. An equal volume of 0.1 mm silica beads was added and the sample vortexed six times for 30 sec (3 min total); samples were incubated on ice between vortexing. The lysate was centrifuged (3500×g for 15 min) and the supernatant was used for further analysis.

Protein lysates were digested by trypsin (Promega). Briefly, protein concentration was determined by a BCA protein assay (Thermo Scientific, Rockford, IL). Of note, we tested whether overall protein load increased in infected animal feces. For each sample, the total µg of protein was divided by the starting weight of fecal matter, resulting in a µg protein/µg mass feces ratio. Values (averages) were as follows: 1.06×10^−3^ (control group 1), 1.17×10^−3^ (control group 2), 7.50×10^−4^ (infected group 1) and 8.27×10^−4^ (infected group 2), demonstrating that overall protein load did not increase significantly in infected samples. Samples were adjusted to equal protein concentration and combined with 7 M urea and 5 mM dithiothreitol (DTT). Samples were incubated at 60°C for 30 min with shaking. Trypsin was resuspended in 150 mM ammonium bicarbonate and warmed to 37°C for 10 min prior to use. Samples were diluted 10-fold with 150 mM ammonium bicarbonate, and CaCl_2_ was added to a final concentration of 1 mM. Finally, trypsin was added to each sample (final concentration 1 unit trypsin:50 units protein) and incubated for 3 h at 37°C with gentle shaking. Peptide samples then underwent strong cation exchange solid phase extraction.

### Protein Analysis: Mass Spectrometry

Peptide samples from both experimental groups were analyzed using a nano capillary liquid chromatography (LC) column [16], [17] coupled to an LTQ Orbitrap mass spectrometer (Thermo Fisher Scientific, San Jose, CA) via an in-house-manufactured interface. The heated capillary temperature and spray voltage were 200°C and 2.2 kV, respectively. For each cycle, the ten most abundant ions from MS analysis were selected for MS/MS analysis. Experimental group 2 samples also were analyzed using LC and an LTQ Orbitrap Velos (Thermo Fisher Scientific, San Jose, CA) for high resolution measurements (Group 1 samples were not subjected to high resolution measurements because of limited sample material). For this instrument, the heated capillary temperature and spray voltage were 300°C and 2.3 kV, respectively. For each cycle, the twenty most abundant ions from MS analysis were selected for MS/MS analysis. For all analyses, data acquisition occurred over an *m/z* range of 400 to 2000.

### Protein Analysis: Data Analysis

Raw spectra were searched using SEQUEST ([18]) against three different protein databases: *Mus musculus* [19], *Salmonella enterica* serovar Typhimurium 14028 [13], and mouse gut commensal metagenome (Peterson C. et al., manuscript in preparation). Results were filtered using the Mass spectrum generating function (MS-GF [20]) SpecProb value as follows: *Mus musculus* (SpecProb ≤1×10^−11^; false discovery rate (FDR) = 1–1.5%), *S*. Typhimurium (SpecProb ≤5×10^−11^; FDR = 6.1–6.8%) and commensal microbiota (SpecProb ≤5×10^−12^; FDR = 3.4%). Peptide observation counts and spectral counts were calculated using Microsoft Access. Proteins with a spectral count of one that were observed at only one time point in all LC-MS/MS analyses were removed. Spectral count and total number of protein observations are used as noted in text, figures, and tables. Statistical differences between control and infected spectral counts from mouse, *Salmonella*, and commensal proteins were identified using QuasiTel [21]. The analysis was conducted in the R software environment [22]. P-values adjusted for false discovery rate were used to filter the QuasiTel results. Early time points (days −1 and +1) were filtered out of the statistical analysis because minimal differences in protein abundance between control and treated mice were expected. These early time points were used as a negative control in the statistical analysis, to determine if differences in protein abundance could be detected in the absence of disease (no significant changes were found). In addition, the last time point, day 28, was removed due to several mice succumbing to disease. The QuasiTel analysis was performed to determine if there were significant differences between control and treated mice on days 3, 6, 10, 14 and 21.

### PCR Amplification and Sequencing Preparation

DNA was extracted from P1 samples (see Fecal sample preparation) using the MasterPure Gram Positive DNA Purification Kit (Epicentre Biotechnologies; Madison, WI). Quadruplicate 50 µl PCR reactions for each sample (50 ng of starting material) were assembled using Phusion Hot Start II DNA Polymerase (Thermo Scientific; Lafayette, CO) and a primer containing both the V3 to V5 variable region of the 16S rRNA and a unique barcode sequence (see Table S4). The samples were amplified using the following protocol: 98°C for 2 min, 40 cycles of 98°C for 10 s, 53°C for 30 s, and 72°C for 1 min, and 72°C for 5 min. The completed reactions were resolved on a 1% agarose gel, and the DNA band near 550 base pairs was excised and purified using the QIAquick Gel Extraction Kit (Qiagen; Valencia, CA). Equal amounts of each uniquely labeled DNA (calculated through serial dilution RT-PCR analysis) were combined, purified using the QIAquick PCR Cleanup Kit (Qiagen; Valencia, CA), and submitted for sequencing that followed the standard ABI Sanger protocol. Total number of 16 s reads ranged from 5128 to 52525, with a mean 23961 and median of 25404 reads.

### RNA Extraction and RT-PCR Analysis

Total RNA of the mouse microbiome was extracted from mouse fecal matter using the RNA PowerSoil Total RNA Isolation Kit (MoBio Laboratories, Inc.; Carlsbad, CA) according to manufacturer’s protocol. The RNA was then purified using the RNEasy Mini Kit (Qiagen; Valencia, CA) according to the manufacturer’s protocol. 0.5 µg of total RNA from each sample was combined with random hexamers and SuperScript III reverse transcriptase (Invitrogen; Grand Island, NY) to create cDNA and subsequently cleaned up as previously described with the following modification: a 25 mM dNTP mix was used omitting the amino-allyl dUTP [23]. Quantitative RT-PCR was performed on the LightCycler 480 System (Roche Applied Sciences, Indianapolis, IN) using the LightCycler 480 SYBR Green I Master Mix with 1.25 µM final concentration of each primer. Quadruplicate reactions using 5.0 ng of cDNA were amplified with the following protocol: 95°C for 5 minutes and then 50 cycles of 95°C for 10 seconds, 56°C for 10 seconds, and 72°C for 10 seconds. Second derivative maximum and melting curve analysis was done in the LightCycler 480 software and an average of the quadruplicates was calculated for delta Ct comparisons.

### Metabolite Extraction

All chemicals and reagents used in metabolomics analyses were purchased from Sigma-Aldrich (St. Louis, MO), except for ammonium bicarbonate (Merck, Darmstadt, Germany), mixture of fatty acid methyl esters (FAMEs) and deuterated myristic acid (Agilent Technologies, Santa Clara, CA). Deionized and purified water was used to prepare buffer and standard solutions (Nanopure Infinity ultrapure water system, Barnstead, Newton, WA). SN2 samples (see Fecal sample preparation) were transferred to 0.6 ml microcentrifuge tubes, and water soluble metabolites were extracted with four volumes of chilled (−20°C) chloroform:methanol mixture (2∶1) [24]. After separating the two phases via centrifugation (12,000×g, 5 min), the upper aqueous layers were transferred to glass vials and dried under a vacuum concentrator (SpeedVac; Thermo Scientific, Waltham, MA). All extracted metabolites were subjected to chemical derivatization to enhance their stability and volatility during GC-MS analysis. Methoxyamine in pyridine (30 mg/ml) was added to each dried sample, and incubated at 37°C with shaking for 90 min to protect carbonyl groups and reduce the number of tautomeric peaks. *N*-methyl-*N*-(trimethylsilyl) trifluoroacetamide (MSTFA) with 1% trimethylchlorosilane (TMCS) was then added, followed by incubation at 37°C with shaking for 30 min to transform hydroxyl and amine groups to trimethylsilyated (TMS) forms [25]. The samples were then allowed to cool to room temperature and were analyzed using gas chromatography (GC)-MS.

### Metabolite Analysis

The GC-MS system used in this study consisted of an Agilent GC 7890A coupled with a single quadrupole MSD 5975C (Agilent Technologies, Santa Clara, CA). GC separations were performed on a HP-5MS column (30 m×0.25 mm×0.25 µm; Agilent Technologies). The injection mode was splitless and 1 µl of each sample was injected; the injection port temperature was held at 250°C throughout the analysis. The GC oven was initially maintained at 60°C for 1 min and then ramped to 325°C by 10°C/min, followed by a 5 min hold at 325°C [26]. GC-MS raw data files were deconvoluted using Metabolite Detector that has a format conversion to netCDF [27]. Retention indices were calculated based on the analysis of a mixture of FAMEs (C8–C30), and used for chromatographic alignment. Detected features were identified using the Agilent Fiehn GC/MS Metabolomics RTL Library [26] (that contains the mass spectra and retention indices of 700 metabolites), as well as the NIST08 GC-MS library. Finally, unidentified and identified features were transferred to DAnTE [28] for further visualization and statistical analysis after log transformation.

### Glycan Analysis: Sample Prep

N-linked glycans were enzymatically released and purified according to an established method [29] with the following minor modifications. The sodium bicarbonate digestion buffer was replaced with sodium phosphate. Equal volumes of 100 mM sodium phosphate and SN2 sample (see Fecal sample preparation) were mixed to form a starting sample to which 10 mM dithiothreitol (DTT) was added and the sample was heated to 95°C in for 2 min to denature proteins. The pH for the PNGase glycan release was adjusted to 7.1 using 1.0 M HCL prior to microwave digestion for 40 min. The reaction was run at a constant microwave power of 20W. Digested samples were cold precipitated with ethanol to remove proteins and the samples were dried. Prior to purification, samples were reduced by treatment with sodium borohydride, desalted, and then concentrated using solid phase extraction (C8 and graphitized carbon cartridges in serial on a GX274 liquid handler; Gilson Inc., Middleton WI). Because the final samples would be separated again when analyzed using LC-MS, the elution fractions from the graphitized carbon cartridge were combined into a single fraction and dried completely. Dried samples were reconstituted in 20 µL of water prior to analysis. 5 µL of sample was used for each injection to the LC column.

### Glycan Analysis: LC-MS

The glycan samples were analyzed using nanocapillary LC-MS (Exactive Orbitrap™; Thermo Scientific Inc., San Jose CA). The 75 µm i.d. ×45 cm long graphite carbon LC column was packed in-house with 3 µm porous Hypercarb particles (Thermo Scientific). A constant flow LC gradient was maintained using Agilent 1200 series nano pumps. The analytical gradient ramped from 1% B to 45% B in 90 min, and then to 95% B 5 min later. Mobile Phase A consisted of water acidified with 0.1% formic acid, and mobile Phase B, of acetonitrile acidified with 0.2% TFA. Gradients and valve switching were controlled by LCMSNet software (an in-house application coded with the.Net framework and built on a custom plug-in based automation framework). The software automated sample analysis and maintained mass spectrometer in continuous operation by alternating between two analytical columns on the LC system. The inlet capillary Exactive Orbitrap™ was heated to 250°C, and a 1.6 KV potential was used for the electrospray. A PAL auto sampler (controlled using LCMSNet software) loaded samples onto the column.

### Glycan Analysis: Data Analysis

The overall pipeline for data analysis was to use DeconTools [30] for LC-MS data preprocessing, MultiAlign for retention time alignment and feature clustering, and Glycolyzer software for N-glycan annotation. Preprocessing involved peak picking, noise thresholding, and monoisotopic identification using THRASH [31], and is based off of the former Decon2LS engine [32]. The glycan averageose C_6.0000_H_9.8124_N_0.3733_O_4.3470_S_0.0_ with an average mass of 156.64662 Da was used in place of the typical peptide averagine. MultiAlign performed retention time alignment and single linkage clustering of monoisotopic masses, and each time course study was aligned to the uninfected starting sample in the set. DeconTools and MultiAlign are open source programs developed at PNNL and freely available at http://omics.pnl.gov/. The Glycolyzer software used to find glycans from the features generated in MultiAlign was written with IgorPro (WaveMetrics, Portland OR). Previously developed theoretical retrosynthetic N-glycan libraries [33] were used to annotate features; a15 ppm root mean square (RMS) mass accuracy cutoff was used for annotation. All of the glycans reported were detected in families (that differed from other members by one monosaccharide) to improve assignment confidence. When glycan isomers were detected with the same mass and multiple retention times, the abundances from each isomer were added to give the reported abundance.

### Glycan Analysis: Study Design

Samples were arranged between the two columns on the LC system to minimize systematic variation within a time series. The control group 1 and infected group 1 series were analyzed on column 1, and control group 2 and infected group 2 series were analyzed on column 2. Comparing pre-infection samples from all four experimental groups was used to qualitatively evaluate any variation between the two columns. Shifts in retention time are accounted for with MultiAlign.

## Results

### Monitoring Protein Expression in the Gut through Time

In humans, gastrointestinal illness caused by non-typhoidal *Salmonella* species, including *S*. Typhimurium, is characterized by acute intestinal inflammation, fever, and neutrophil influx [34]. Because mice do not normally develop gastrointestinal illness, and commensal organisms must be disrupted in order for *S*. Typhimurium to induce gastroenteritis in mice [9], most studies of *S*. Typhimurium behavior in a gut environment have not considered the impact and potential modulatory role of commensal microorganisms. To elucidate the interplay between the host, pathogen, and commensal microbial community, we employed a pan-omics strategy to investigate a persistent oral *Salmonella* infection of 129/SvJ mice. The 129/SvJ mouse model was chosen for several reasons: 129/SvJ mice 1) are genetically “resistant” to *S*. Typhimurium infection (i.e., they express Slc11A1 [35]), allowing a longer time course of study with minimal mouse death, 2) do not require antibiotic pre-treatment in order to promote gastrointestinal infection, and 3) shed *S*. Typhimurium in the feces for at least one year post-infection [11]. We monitored the dynamics of gastrointestinal *Salmonella* infection through time by orally infecting two groups of mice with *S*. Typhimurium (and two control groups with saline only), collecting fecal samples at eight time points pre- and post-infection, and processing the material for downstream omics analyses.

**Figure 1:**
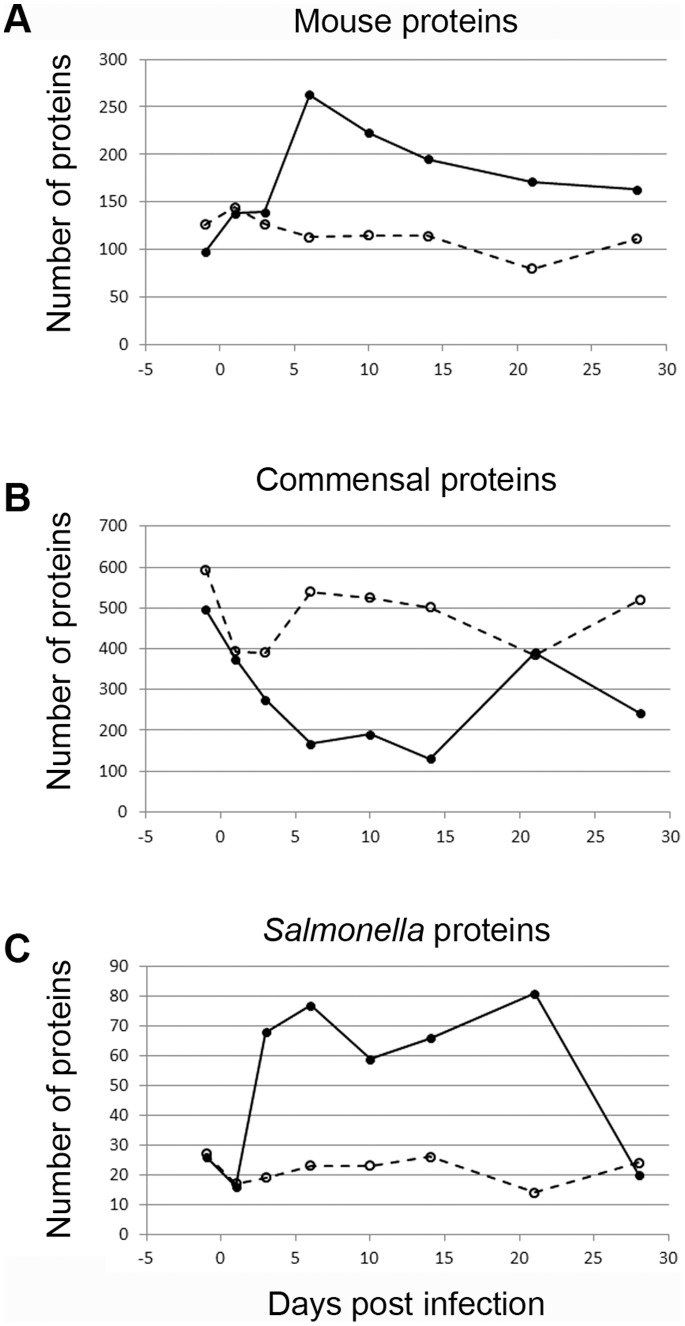
Dynamics of host, commensal, and *S*. Typhimurium protein identification through infection. Fecal samples were processed and analyzed for the presence of (A) mouse-, (B) commensal microbiota-, and (C) *S*. Typhimurium 14028s-derived proteins in infected (solid line, solid circles) and uninfected control (dashed line, open circles) mice. Data shown here represent the summed protein identifications from mass spectral analyses (including biological replicates). Independent replicate plots are available in Fig. S1.

Proteins derived from the mouse, commensal microbiota, and *S.* Typhimurium were observed in fecal samples (Figure 1: data summed from all analyses; individual experimental group data shown in Figure S1). Observed mouse proteins increased through day 6 (Figures 1A, S1A), and then gradually decreased. Statistical analysis of proteins detected in infected and control mice during the height of *Salmonella* infection (days 3 through 21) identified 22 mouse proteins that significantly changed by infection (p<0.01; Table S3). The number of commensal microbiota-derived proteins decreased quickly after infection (by day 3–6; Figures 1B, S1B), with 46 significantly altered. Concurrently, *S*. Typhimurium protein observations suggested a primary and secondary wave of proliferation (days 6 and 21), followed by a decrease to low levels in mice surviving at day 28 (Figures 1C, S1C). Forty-three *S*. Typhimurium proteins were significantly increased during infection. The low level of *S*. Typhimurium proteins identified in control (uninfected) animals are conserved between Salmonellae and commensal bacteria, and do not change appreciably from background levels through time. These results support the hypothesis that *S*. Typhimurium proliferates in the infected gut, stimulates a host immune response, and disrupts the microbial population structure in the gut by direct and/or indirect mechanisms.

### S. Typhimurium Induces Inflammation in 129/SvJ mice, Including Potential Novel Host Immune Responses

The mouse model of systemic *S*. Typhimurium infection has provided much insight into the host response to *Salmonella*, mechanisms of pathogen clearance, and requirements for generation of protective immunity against subsequent infection [36]–[38]. However, the host responses that arise during gastrointestinal infection in a mouse are largely unknown. In humans, *S*. Typhimurium-induced inflammation is characterized by neutrophil transmigration into the intestinal lumen, fever, and complement activation [34]. To investigate the hypothesis that a similar inflammatory gut environment develops following *S*. Typhimurium infection in 129/SvJ mice, we identified host proteins in fecal samples through time (Figure 1A; Table S1).

The response to *S*. Typhimurium infection was dominated by known and potentially novel innate immune factors. We observed evidence of transmigration and activation of neutrophils, up-regulation of cell surface molecules used to coordinate immune responses, complement activation, and inflammatory antibacterial responses (Table S2). Interestingly, many proteins detected have not previously been associated with an anti-*Salmonella* response during infection *in vivo*. Proteins such as cell surface A33 antigen, deleted in malignant brain tumors 1 protein (DMBT1), lithostathine-1 and -2 (Reg-I and –II), and serum amyloid P component (SAP) may therefore represent novel parts of the response to natural *S*. Typhimurium infection (or, pathogen exposure in a more general sense) in a gut populated by commensal organisms. Host proteins from fecal samples highlight the inflammatory nature of the infected gut environment, including novel host factors in gastrointestinal *S*. Typhimurium infection.

### Salmonella Alters the Native Gut Community

*S*. Typhimurium can withstand the antimicrobial activity of host inflammatory molecules like Reg-IIIβ [39], yet commensal organisms are generally more susceptible to such insults [39], [40]. The host inflammatory response to *S*. Typhimurium, as well as direct anti-commensal activities of *S*. Typhimurium that may exist, cause a striking disruption in the commensal population during gastrointestinal infection (Figure 1B, Table S2). We hypothesized that the inflammatory, infected gut environment may uniquely alter specific commensal organism lineages. To test this hypothesis, we performed 16 s rDNA sequencing to profile the microbial community structure during infection through time in 129/SvJ mice (for which the commensal population had not yet been characterized).

**Figure 2:**
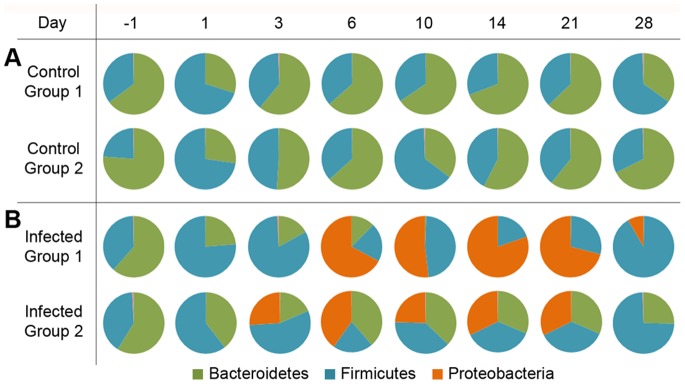
*Salmonella* infection disrupts the commensal microbial community at the phylum level. Phylum level 16 s rDNA analysis of cellular sample fractions revealed a pre-infection microbial population composed of primarily *Bacteroidetes* (green) and *Firmicutes* (teal) (left side), which fluctuates at day 1 in all experimental groups after withdrawal of food overnight prior to infection. (A) Control animals maintain relatively stable microbial populations, whereas the gut of (B) infected animals is taken over by *Proteobacteria* (orange; specifically *Salmonella*). By day 28 (right side), surviving animals are clearing *S*. Typhimurium from the intestinal lumen. Values represented are the percentage of each phylum; for figure clarity, only those present at >1% of the total population are included.

The initial microbial community of the mouse gut contained primarily *Bacteroidetes* and *Firmicutes* (Figure 2, day −1) [41]. Interestingly, the *Barnesiella* genus, which has recently been isolated from both chicken and human fecal samples [42]–[44], dominated the phylum *Bacteroidetes* population in all cases, with *Blautia* comprising abundant *Firmicutes* (Figure S2). Following overnight food withdrawal prior to oral infection, all mice exhibited a temporary increase in organisms of the *Oscillibacter* genus (Figures 2, S2, day 1). Control mice regained the initial microbial population balance by day 3–6 and maintained relatively stable composition thereafter (Figures 2A, S2A). *Proteobacteria* proliferated in two waves in *Salmonella*-infected mice (specifically due to increased Salmonellae; Figures 2B, S2B, S3), then ultimately decreased by day 28, presumably due to host- and potentially commensal-mediated clearance of the infection.

As *Salmonella* survives and proliferates in the inflamed gut, resident organisms are largely out-competed; in experimental group 1 in particular, *Bacteroidetes* are nearly eliminated from the gut by day 10 (Figure 2B). Conversely, the Enterococci exhibit elevated fitness in the *S*. Typhimurium infected gut. Finally, by day 28 surviving mice appear to be reestablishing the original commensal population (Figure 2B; possibly from organisms retained in the gut or via another reservoir [45]). Extended time courses would likely reveal transient shedding of *S*. Typhimurium from the physiological reservoir (mesenteric lymph nodes) into the intestinal tract during persistent infection [11]. Although the infection kinetics demonstrated biological variability between experimental groups (Salmonellae increasing at day 6 for group 1 vs. day 3 for group 2), this timing was consistent within each group through the different omics analyses presented here. The combined impacts of host inflammatory molecules and direct mechanisms of *S*. Typhimurium effectively disrupt the commensal microbial population in the gut, thereby reducing *Bacteroidetes* and facilitating the outgrowth of the *Firmicute, Enterococcus*.

### Salmonella Infection-induced Gut Metabolite Changes

**Figure 3:**
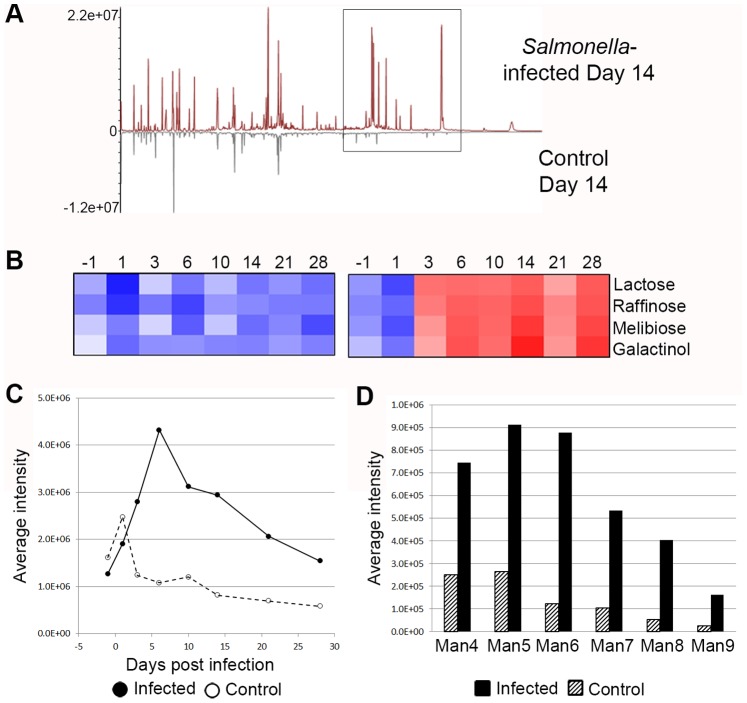
Environmental changes in the *Salmonella*-infected gut. GC-MS metabolite analysis of soluble factors in fecal samples revealed that metabolites in infected animals are altered from those of control animals. A direct comparison of metabolite profiles at day 14 (A) shows the appearance of metabolites in infected samples (box). These peaks represent sugars which accumulate in the infected gut, in the absence of commensal microorganisms that normally metabolize these structures. (B) Intensity of selected peaks in infected animals is represented in a heat map (data shown are representative of one of two biological replicates (experimental group 1); experimental group 2 is presented in Figure S4). Additionally, N-linked glycan abundance increases with *S*. Typhimurium-induced gastroenteritis. Abundance of five fucosylated glycans (C) increases in infected animals (solid line with solid circles) over control animals (open circles with dashed line). In addition, (D) high mannose glycans were increased in infected animals (solid fill bars) over control animals (pattern filled bars). Values in (C) and (D) are averaged from both biological replicates (representing pooled samples from groups of mice in each case). Individual replicate data is provided in Figure S6. Heat map shows intensity data following log 2 and Z-score transformation, where red is more intense and blue is less intense.

Gut microbes perform many metabolic functions within the host, including breakdown of a diverse range of incompletely digested carbohydrates that pass through the mammalian digestive tract, which contributes to healthy digestion and nutrient absorption. Significant alterations in the gut environment upon *S*. Typhimurium infection, induced by the inflammatory host response (Tables S1, S2) and microbial population shift (Figure 2, Table S1B), suggested that the metabolite composition of the gut may be altered. Analysis of water-soluble metabolites derived from the gut revealed a mixture of candidate host-, microbe-, and/or diet-derived molecules. Infected animals demonstrated metabolite changes compared to control animals by day 3 post infection (Figures S4A–B), coinciding with observed changes in proteomic (Figure 1) and 16 s rDNA (Figure 2) analyses. Direct comparison of metabolites in control and Salmonella-infected animals (day 14, when *S*. Typhimurium is thriving in the infected gut; Figures 1C, S3) showed clear differences (Figures 3A, S4C), particularly for sugar moieties that accumulate in the infected gut (lactose, galactinol, melibiose, and raffinose; Figures 3B, S4C–D). *Salmonella* and murine host metabolic pathways lack the enzymes necessary to utilize most of these sugars; rather, commensal microorganisms, such as *Bacteroidetes*, express an extensive glycosidase repertoire [46]. Reduction of specific microbial lineages in infected animals (Figure 2B) leads to undigested carbohydrate accumulation (Figures 3, S4). Melibiose represents the exception, as *S*. Typhimurium can metabolize this sugar. However, catabolism of melibiose occurs only when other nutrient sources, such as glucose, are unavailable [47]. Because melibiose accumulates during *S*. Typhimurium-induced intestinal inflammation and we did not observe evidence of melibiose utilization in *Salmonella* proteomics analyses (see the following section; Table S1C), we speculate that the inflamed gut environment contains more energetically desirable sugars in the absence of commensal microorganisms, or *Salmonella* is utilizing this sugar at a level below our ability to detect. Metabolomic analysis supports the idea that changes during *S*. Typhimurium infection in the microbial population structure and composition (Figure 2), as well as the influence of inflammatory mediators (Table S2) alters the gut environment (Figures 3, S4).

### Salmonella Protein Expression in the Mouse Gut

Introduction of the pathogen *S*. Typhimurium into the gut environment initiates the inflammatory host response and disrupts metabolites and commensal microorganisms. Our study provides a global perspective on the gastrointestinal lifestyle of *Salmonella*. Proteomic analysis revealed nearly 140 *S*. Typhimurium proteins expressed during infection (Figures 1C, S1C, and Table S1C). Identification of several stress response-related proteins and outer membrane proteins and lipoproteins during infection suggests that *S*. Typhimurium likely responds to various host environments, and may promote surface modification mechanisms to resist host immune insults [48]. In the inflammatory gut, *S*. Typhimurium respires anaerobically to out-compete fermentative commensal microbes by using tetrathionate as an alternative terminal electron acceptor for metabolism of compounds such as ethanolamine [49]. Ethanolamine utilization by *S*. Typhimurium is important during oral infection, but not when *S*. Typhimurium is delivered intraperitoneally, which stresses its unique role during pathogenesis in the gut environment [50]. We observed EutM (a protein involved in ethanolamine use [49]) and the two tetrathionate utilization proteins TtrS and TtrA (although detected at low relative abundance), suggesting that this metabolic strategy may be used by *S*. Typhimurium during infection not only in streptomycin-treated mice, but when microbiota are present.

In the intestinal lumen, *S*. Typhimurium expressed sugar utilization proteins, including those involved in galactose, glucose, maltose, and mannitol metabolism (Table S1C). Interestingly, we observed expression of three fucose utilization proteins (FucI, FucU, FucA), as well as expression of fuc genes by qRT-PCR (Figure S5). FucA and FucU have rarely, if ever, been observed in our extensive proteomic studies of *S*. Typhimurium grown in multiple *in vitro* growth conditions (Table S1C; these and other infrequent protein identifications in the gut environment are noted), suggesting *S*. Typhimurium modulates its protein expression in the intestinal environment in response to substrates such as fucose to promote growth.

### Glycan Content of the Infected Gut Suggests Salmonella may Respond to Increased Fucose

Observation of fucose utilization proteins in the *Salmonella*-infected samples suggested that *S*. Typhimurium might respond to and metabolize fucose in a gut environment absent of extensive commensal competition. Glycosylation of abundant gastrointestinal proteins (such as epithelial surface glycoproteins, mucin, and immunoglobulins) often contain fucose moieties [51], and serve as a nutrient source for commensal organisms [46], [52], [53]. To determine whether protein fucosylation was altered in the infected gut, we analyzed the N-linked glycan profile of our samples through time while tracking the abundance of a select family of five fucosylated glycans detected at all time points (Figure S6A). The total fucosylated glycan content increased with infection, likely induced by a combination of the following non-mutually exclusive factors: 1) loss of commensal organisms responsible for metabolizing fucose moieties in the healthy gut that results in accumulation, 2) part of an increased host immune response, as the kinetics of glycan increase and host protein increase coincide (Figures 1A, 3C, S6B–C), or 3) intestinal cell damage during infection. In addition, we observed a significant increase in the entire high mannose glycan series during infection (Figures 3D, S6D–G). High mannose glycans (Man4-Man9) are modified in the endoplasmic reticulum and represent relatively minimally processed glycans. Detection of increased high mannose glycans suggests that glycoproteins are being trafficked outside of cells prior to additional glycan processing in the Golgi apparatus [54]. The source of these glycans is currently unknown, and further targeted investigation will help to elucidate this observation. Our data support the hypothesis that *S*. Typhimurium metabolizes fucose in the gut as part of its gastrointestinal lifestyle, and suggests that glycan release might be an underappreciated immune response mechanism of the host.

### Summary

**Figure 4:**
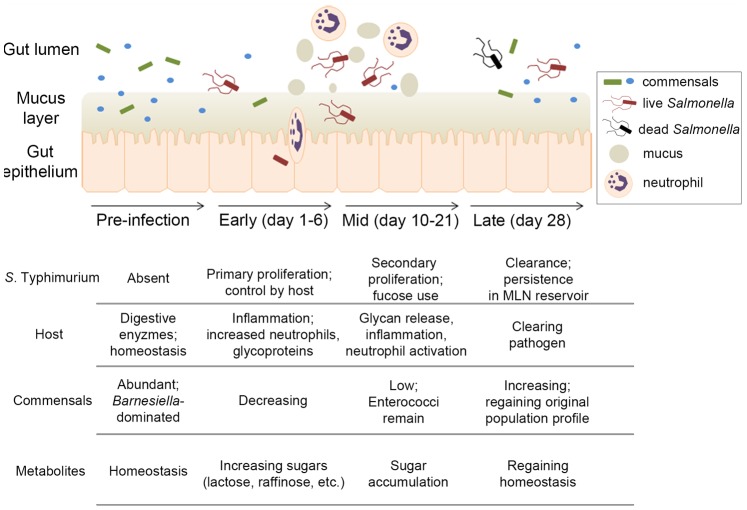
Model of host-pathogen-commensal interactions during *S*. Typhimurium-induced gastroenteritis. Using a pan-omics approach, we have developed a model of the interplay between the mouse, *S*. Typhimurium, and the commensal population during gastrointestinal infection. Prior to pathogen introduction, a *Barnesiella*-dominated commensal population exists in the homeostatic gut. Early in infection, *S*. Typhimurium proliferates, stimulates an inflammatory response characterized by neutrophil activation, and disrupts this microbial community. As the commensal population profile changes, so do metabolites in the gut that are normally metabolized by the microbial community. Fucosylated glycan content also increases in the *Salmonella*-infected gut, potentially as part of an immune response or pathogen clearance mechanism. *S*. Typhimurium senses and responds to fucose availability during gastrointestinal infection, as evidenced by increased expression of fucose utilization proteins. Finally, pathogen clearance from the gut occurs, allowing the gastrointestinal environment to begin to return to pre-infection conditions.

The interplay between a susceptible host, pathogen, and the commensal microbiota during *S*. Typhimurium intestinal infection is complex, and our understanding benefits from concurrently monitoring multiple factors. From fecal-derived samples, our systems approach revealed protein, metabolite, and glycan components from 129/SvJ mice, *S*. Typhimurium, and gut microbiota, generating a model of interactions during infection (Figure 4). Prior to infection, a state of homeostasis exists in the intestinal lumen with maintenance of commensal organisms in association with the mucus layer. Oral *Salmonella* infection disrupts the commensal population, allowing *S*. Typhimurium to proliferate. Concurrently, the host immune system is activated, sending neutrophils to the intestinal lumen and inducing various inflammatory markers. Loss of commensal microbes (likely due in part to the host inflammatory response) and their associated functions is evident mid-way through infection, when metabolites such as fucose and other sugars normally utilized by commensal bacteria accumulate in the gut. During this time, *Salmonella* thrives, sensing increased host glycan presence and utilizing available fucose moieties, among other functions. Resolution of infection by later time points is observed, with a decrease in *S*. Typhimurium abundance, reestablishment of metabolite composition, and outgrowth of indigenous microbiota. This model of interactions during *Salmonella*-induced intestinal inflammation provides a framework both consistent with known factors and revealing new components and insights to this complex model of infection.

## Discussion

The presence of commensal microorganisms on exposed body surfaces has long been known, but the limitations of cultivation technologies have largely underestimated their abundance and diversity. Only with the more recent advent of culture-independent molecular biology techniques has the composition of these populations and their contribution to health and disease become increasingly appreciated. To investigate the potential influence of commensal microbiota on host-pathogen interplay during infection, we used multiple complementary technologies that allowed us to monitor interactions between the mouse host, enteric pathogen *S*. Typhimurium, and the commensal gut microbiota during gastrointestinal infection.

While *S*. Typhimurium systemic infection has been extensively studied in the mouse model, the gastrointestinal lifestyle of *Salmonella* has been less studied due primarily to the natural course of infection in the laboratory mouse. Studies in mice that have focused on intestinal infection most often require antibiotic pre-treatment to rid the gut of native microbiota [9]. As we were interested in studying the biology of commensal organisms normally inhabiting the gut during enteric infection, we used the 129/SvJ model of persistent *Salmonella* infection, in which gastrointestinal infection occurs without antibiotic treatment and pathogenic organisms are shed in fecal material, to achieve the experimental goals of this study.

In humans, the host response to *Salmonella*-induced gastroenteritis is characterized by acute inflammation, and our proteomic analysis of *S*. Typhimurium infection in 129/SvJ mice demonstrates induction of an inflammatory gut environment (Figure 1A, Table S1A). Not only were proteins known to be integral to the innate immune response against *S*. Typhimurium identified, but potentially novel host immune factors were observed (Table S2). These novel immune factors may influence or contribute to the disruption of the commensal microbial population, similar to the recently reported impact of Reg-III [39], [40], and follow-up studies will be focused on understanding if they play a role in the anti-*Salmonella* immune response during gastrointestinal infection.

The impact of *Salmonella* infection on the native commensal organisms in the gut is significant [10], as demonstrated by the number of proteins identified from this population (Figures 1B, S1B) and the 16s rDNA sequencing analysis (Figure 2). A microbial population initially dominated by the phyla *Bacteroidetes* and *Firmicutes* in uninfected mice exhibited a shift in population balance following overnight starvation, characterized by proliferation of *Oscillibacter* species. While not our focus, the microbial community alterations found upon diet change add evidence that diet, the microbiota, and many aspects of health are substantially interdependent [5], and suggest *Oscillibacter* may play a role in mediating such effects.

Proliferation of Salmonellae in infected animals occurs as the indigenous population is flushed from the intestinal lumen (Figures 1C, 2, S2, S3). The mechanisms used by *Salmonella* to induce this clearance in the gut, whether direct via antimicrobial mechanisms or indirect via induction of inflammation, are not completely clear. Interestingly, Enterococci appear to thrive in the *Salmonella*-infected gut environment (Figure S2). *Enterococcus* species persistence during *S*. Typhimurium infection in the streptomycin-treated mouse model [55], and resistance to the antimicrobial Reg-IIIβ (but not to Reg-IIIγ [39], [40]) suggest that Enterococci may possess mechanisms to withstand inflammatory environments and perhaps thrive in the absence of other microbiota. The commensal organism *Enterococcus faecalis* can act as an opportunistic pathogen, and therefore its outgrowth may contribute to the gastrointestinal disease state during *Salmonella* infection. While *S*. Typhimurium is being cleared in mice surviving at day 28 (Figure S2), the pre-infection balance of the commensal population has not yet been fully regained, particularly in experimental group 1 where *Enterococcus* dominates. Further study will elucidate whether Enterococci are more resistant to antimicrobial mechanisms of the host, whether they thrive somehow in response to or in the presence of *S*. Typhimurium, and whether the commensal microbiota population fully recovers from disruption during infection.

The alteration in microbial population structure also was evident in complementary metabolomics analyses. Disruption of the commensal microbial population and concurrent growth of *Salmonella* shifts the microbial balance by day 3–6, functionally changing the metabolite profile (Figures 3, S4). One striking example of the metabolite changes caused by infection is the accumulation of specific sugars in the gut. Lactose, melibiose, raffinose, and galactinol are normally digested in the colon by commensal microbiota; however, these metabolic functions are lost upon their removal from the gut, and neither *S*. Typhimurium nor the murine host is able to compensate. Further metabolomic investigation may elucidate the impact of increased sugars on gastrointestinal function during infection. Incorporation of metabolomic analysis in this study provided complementary data to support the conclusion that the gut environment was altered in individual microbial lineages during infection and that functional traits had been disrupted.

Changes in the gut were caused by introduction of the pathogen *S*. Typhimurium, which must respond appropriately to environmental alterations to survive, proliferate, and cause disease. The lifestyle of *S*. Typhimurium in a normal mouse intestine has been understudied, especially from the broad systems-level perspective described here. Our results demonstrate that *Salmonella* proliferates and thrives in the infected gut (Figures 2, S2), and is able to withstand a range of stresses imposed by the host, as supported by identification of *S*. Typhimurium proteins indicative of a stress response (Table S1C). Interestingly, proteomic analysis suggested that *S*. Typhimurium may use fucose in the gut as a nutrient source, which was supported by evidence of *fuc* gene expression *in vivo* (Figure S5). Fucosylated glycoproteins are abundant in the gut, and we observed an increase in fucosylated N-linked glycans in infected animals (Figures 3C, S6B–C). Increased fucose in the intestinal lumen is likely due to multiple factors, including a decreased consumption rate correlated with the reduction in commensal organism competition, shedding of host-derived fucosylated glycoproteins during inflammation and/or infection [4], [56], or release due to intestinal epithelial cell damage. Commensal organisms, such as *Bifidobacterium*, *Ruminococcus*, and *Bacteroides* are known to utilize glycan sugars as a nutrient source in the gut [46], [52], [53]. Therefore, it follows that in the absence of these organisms during *S*. Typhimurium intestinal infection (Figures 1B, 2), sugar moieties may be more available and abundant. Release of fucose during inflammation may represent a mechanism by which the host responds to infection [4], [56], [57], potentially acting as a signal to nearby host cells during the immune response [58], or serving to interact with *S.* Typhimurium in a way that facilitates removal of the pathogen. Additionally, we observed increased high mannose glycans in the infected animals (Figures 3C, S6D–G). Although the source and reason for this result are currently unknown, we speculate that high mannose glycans are derived from specific host proteins (such as cell surface A33 antigen, which we identified and is known to possess such glycans) [59] (Tables S1, S2), are a byproduct of epithelial cell death and shedding into the lumen (during which incomplete glycans might be inadvertently released), or may represent a novel host response to infection.

The expression of *Salmonella* proteins involved in fucose utilization led us to investigate whether the ability to use fucose in the gastrointestinal environment confers a growth advantage to *S*. Typhimurium. Oral competition experiments were performed in 129/SvJ mice: WT *S*. Typhimurium was mixed in a 1∶1 ratio with a mutant lacking all *fuc* genes (Δ*fucOAPIKUR*), and both the WT and mutant bacteria shed in fecal samples were quantified. As *Salmonella* is known for its robust metabolism, we hypothesized that the inability to use fucose would have a minimal impact or no effect at all. Surprisingly, a growth difference between the WT and mutant strain was evident, showing a slight growth advantage for the *fuc* mutant strain (Figure S7). These data suggest that fucose metabolism does likely occur *in vivo* as part of the gastrointestinal lifestyle of *Salmonella*, yet the environmental cues and regulatory control of carbohydrate (including fucose) metabolism in the gut environment is more complex than anticipated. Factors such as anatomical location within the gut, nutrient availability, microbiota composition, and bacterial population density may likely influence metabolic capacity during infection. These results suggest the possibility that fucose metabolism in the intestinal lumen may normally act as part of a “gastrointestinal signal” to *Salmonella*, and the absence of this metabolic capacity may stimulate an altered virulence program in the bacterium. Deciphering the complexity of nutrient utilization in the microbiota-occupied gut is beyond the scope of the present investigation; however, further studies will help to determine the nuances that influence *S*. Typhimurium fucose metabolism in the gastrointestinal tract and how the course of infection may be impacted as a result.

To conclude, this study demonstrates that the union of complementary omics technologies to an under-investigated biological system allows for concurrent monitoring of multiple factors during *S*. Typhimurium gut infection and development of a working model of these interactions (Figure 4). Host, commensal, and pathogen behavior in this complex environment, including known and novel factors, provide insights into the lifestyle of *S*. Typhimurium in the gut and the host response to infection. Importantly, this type of multi-omics approach could be adapted to investigate many complex model systems of infection.

## Supporting Information

Figure S1 **Proteomic analysis of individual biological replicates.** Samples from two biological replicates were run on 2 instruments as follows: experimental group 1 samples were analyzed by LTQ Orbitrap only (1), while experimental group 2 samples were analyzed by LTQ Orbitrap (2a) and Velos Orbitrap (2b). Protein identifications in individual biological replicates and sample runs for (A) mouse, (B) commensal organisms and (C) *S*. Typhimurium. Data from analyses of biological replicate 1 and 2 on LTQ Orbitrap are on the left axis; data from analysis of biological replicate 2 on the Velos Orbitrap is on the right axis. Values are total protein identifications (spectral counts). Symbols represent the following samples: open circles (control group 1), solid circles (infected group 1), open triangles (control group 2a), solid triangles (infected group 2a), open squares (control group 2b), and solid squares (infected group 2b).
(PDF)

Figure S2 ***Salmonella*** **infection disrupts the commensal microbial community at the genus level.** Presentation of the top 10 most abundant genera (each representing greater than 0.5% of the total population as determined by 16s rDNA sequencing) reveals a pre-infection microbial population composed of primarily *Barnesiella* (green), with a small fraction of *Blautia* (purple) species (left side), which fluctuates at day 1 with a transient *Oscillibacter* (gray) increase in all experimental groups after overnight starvation. (A) Control animals maintain relatively stable microbial populations, whereas the gut of (B) infected animals is taken over by *Salmonella* (in orange). Values represented are the percentage of each genus within the top 10 genera (>97% of total population); for clarity, genera of low abundance were removed during figure generation.
(PDF)

Figure S3 **16S rDNA analysis of the** ***Salmonella*** **and other non-** ***Salmonella*** **Proteobacterial genera through time.** (A) Raw data shows the proliferation of *Salmonella* during infection and clearance by day 28. (B) Other Proteobacteria are present at very low levels (note axis scale in A and B), and do not significantly contribute to observed Proteobacteria increase in infected animals. Symbols represent the following samples: open triangles (control group 1), solid triangles (infected group 1), open circles (control group 2), and solid circles (infected group 2).
(PDF)

Figure S4 ***S*** **. Typhimurium infection induces metabolite changes in the gut environment.** GC-MS metabolite analysis of soluble factors in fecal samples revealed that (A,B) the profile of metabolites in infected animals (right panel, red) is distinct from that of control animals (left panel, black). Chromatograms presented here, with early time points in the foreground and later time points in the background, demonstrate significantly increased abundance of specific metabolites by day 3 post infection in *Salmonella*-infected animals. (A) represents experimental group 1 data, while (B) represents experimental group 2 data. The same scaling for the y-axis was applied to all chromatograms for relative chromatographic comparison. A direct comparison of metabolite profiles at day 14 (C) shows the appearance of metabolites in infected samples (box). These peaks represent sugars which accumulate in the infected gut, in the absence of commensal microorganisms that normally metabolize these structures. (D) Intensity of these peaks in infected animals is represented in a heat map. Data shown in (C) and (D) are representative of experimental group 2. Heat map shows intensity data following log 2 and Z-score transformation, where red is more intense and blue is less intense.
(PDF)

Figure S5 **Expression of** ***fuc*** **genes** ***in vivo*** **following** ***Salmonella*** **infection.** qRT-PCR analysis revealed increased expression of genes in the *fuc* regulon of *S*. Typhimurium during infection. Data presented as fold change over expression in uninfected animals.
(PDF)

Figure S6 **Fucose and high mannose glycan detection in individual biological replicates.** (A) Five fucosylated glycan moieties that were observed at each time point were selected for analysis. These fucosylated glycans increased during infection in both (B) experimental group 1 and (C) experimental group 2, in comparison to control animals. Data from infected animals are represented with closed circles and a solid line, while data from control animals are shown as open circles with a dashed line. High mannose series glycans are present at low levels in control samples from experimental group 1 (D) and experimental group 2 (F), and are increased in infected animals in experimental group 1 (E) and experimental group 2 (G). Symbols represent the following species in parts C-F: Man4 (open circles), Man5 (solid circles), Man6 (open squares), Man7 (solid squares), Man8 (open diamonds), Man9 (solid diamonds). Intensity values represent averaged data from pooled fecal samples, by experimental group.
(PDF)

Figure S7 **Inability to utilize fucose demonstrates in vivo phenotype.** WT and Δfuc strains were mixed at a 1∶1 ratio and used to orally infect 129/SvJ mice. Quantification of each strain in the shed feces demonstrated a slight growth advantage *in vivo* of the Δfuc strain. Two replicate experiments were performed, shown in (A) and (B). Each filled circle represents the average competitive index value, calculated from three groups (with three mice per group). Error bars represent the standard deviation values.
(PDF)

Table S1 **All filter-passing protein identifications.** (A) Mouse-derived, (B) microbiota-derived, and (C) *Salmonella*-derived protein identifications.
(PDF)

Table S2 **Selected mouse proteins identified during infection.** The immune response to gastrointestinal *S*. Typhimurium infection in humans is characterized by an inflammatory response and neutrophil activation. Proteomic results support a similar host response in 129/SvJ mice, which includes (but is not limited to) expression of neutrophil-related proteins, cell surface proteins, and inflammatory molecules. Selected protein names and known/proposed functions are shown. Asterisks highlight host proteins that have not, to our knowledge, been previously linked to the anti-*Salmonella* immune response.
(PDF)

Table S3 **Proteins determined to be statistically significantly different in control and infected mice.**
(PDF)

Table S4 **Primer sequences used for 16s community profiling.** Primer sequences are listed for each sample, including four experimental groups (control 1, control 2, infected 1, and infected 2), with eight time points per group (−1, 1, 3, 6, 10, 14, 21, and 18 days post-infection).
(XLSX)
