# Supplementary figures and images for: A Multi-Omic View of Host-Pathogen-Commensal Interplay in Salmonella-Mediated Intestinal Infection
Source: PLoS One. 2013 Jun 26;8(6):e67155. doi: 10.1371/journal.pone.0067155 (PMC3694140; doi:10.1371/journal.pone.0067155)

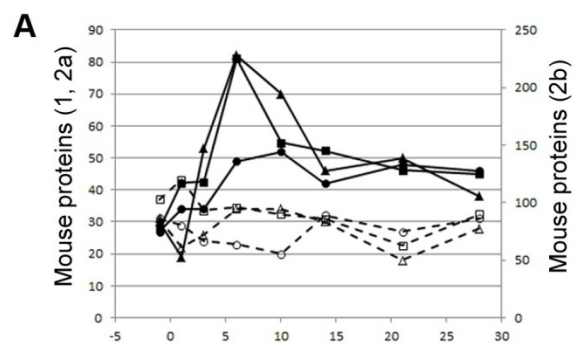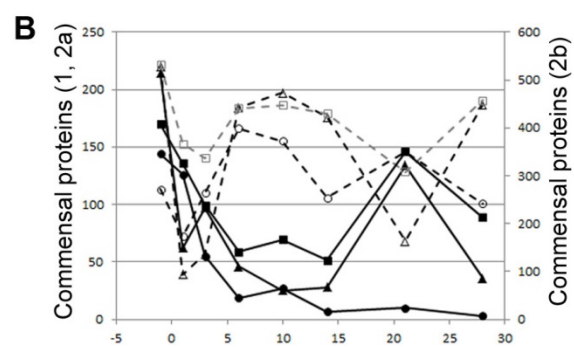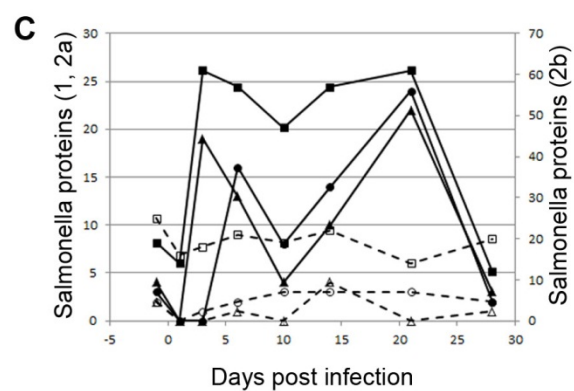

Supplement: Figure S1 — Proteomic analysis of individual biological replicates. Samples from two biological replicates were run on 2 instruments as follows: experimental group 1 samples were analyzed by LTQ Orbitrap only (1), while experimental group 2 samples were analyzed by LTQ Orbitrap (2a) and Velos Orbitrap (2b). Protein identifications in individual biological replicates and sample runs for (A) mouse, (B) commensal organisms and (C) S. Typhimurium. Data from analyses of biological replicate 1 and 2 on LTQ Orbitrap are on the left axis; data from analysis of biological replicate 2 on the Velos Orbitrap is on the right axis. Values are total protein identifications (spectral counts). Symbols represent the following samples: open circles (control group 1), solid circles (infected group 1), open triangles (control group 2a), solid triangles (infected group 2a), open squares (control group 2b), and solid squares (infected group 2b). (PDF) [file pone.0067155.s001.pdf]

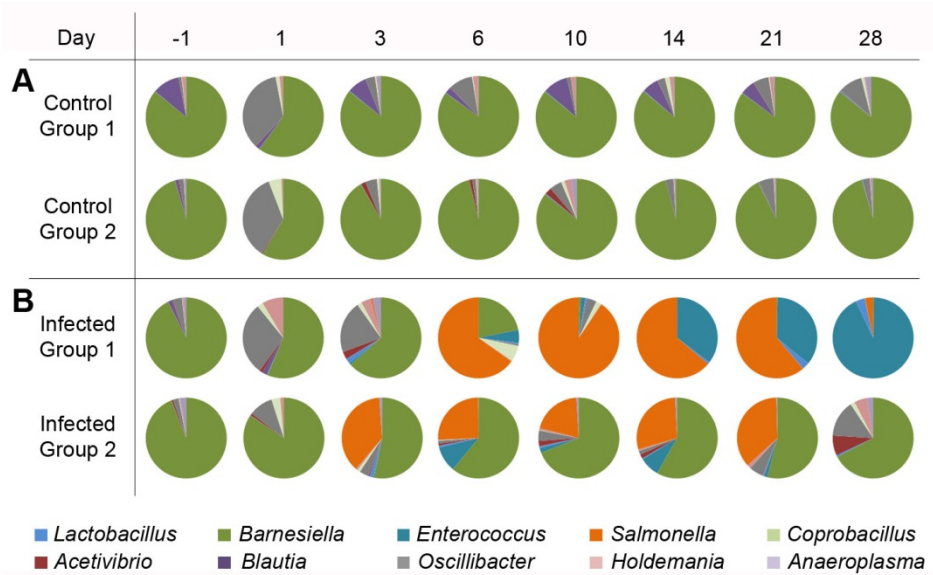

Supplement: Figure S2 — Salmonella infection disrupts the commensal microbial community at the genus level. Presentation of the top 10 most abundant genera (each representing greater than 0.5% of the total population as determined by 16s rDNA sequencing) reveals a pre-infection microbial population composed of primarily Barnesiella (green), with a small fraction of Blautia (purple) species (left side), which fluctuates at day 1 with a transient Oscillibacter (gray) increase in all experimental groups after overnight starvation. (A) Control animals maintain relatively stable microbial populations, whereas the gut of (B) infected animals is taken over by Salmonella (in orange). Values represented are the percentage of each genus within the top 10 genera (>97% of total population); for clarity, genera of low abundance were removed during figure generation. (PDF) [file pone.0067155.s002.pdf]

**A**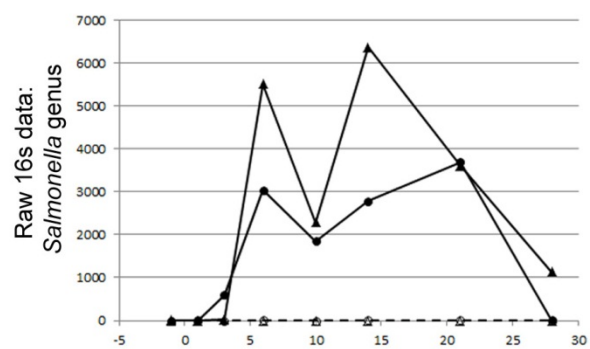**B**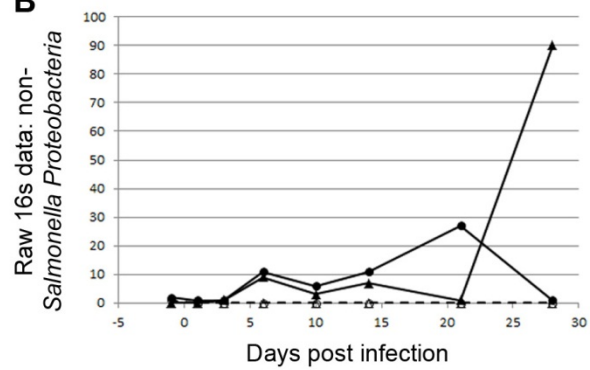

Supplement: Figure S3 — 16S rDNA analysis of the Salmonella and other non- Salmonella Proteobacterial genera through time. (A) Raw data shows the proliferation of Salmonella during infection and clearance by day 28. (B) Other Proteobacteria are present at very low levels (note axis scale in A and B), and do not significantly contribute to observed Proteobacteria increase in infected animals. Symbols represent the following samples: open triangles (control group 1), solid triangles (infected group 1), open circles (control group 2), and solid circles (infected group 2). (PDF) [file pone.0067155.s003.pdf]

**A**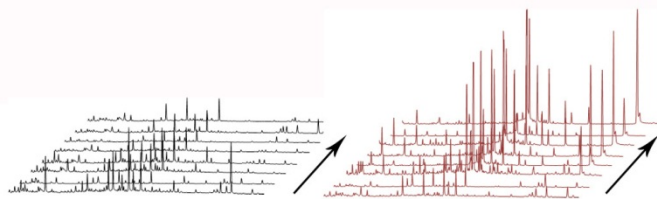**B**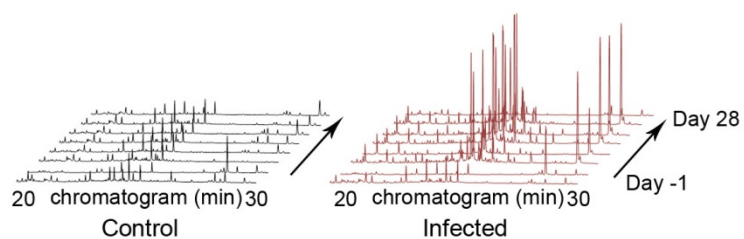**C**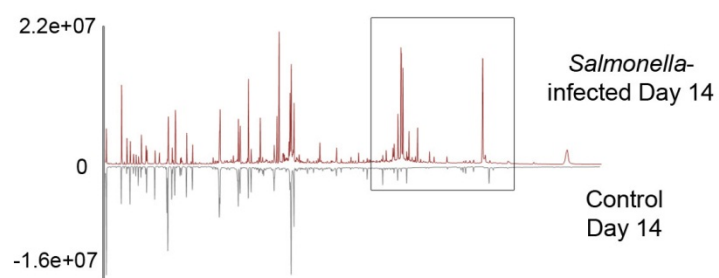**D**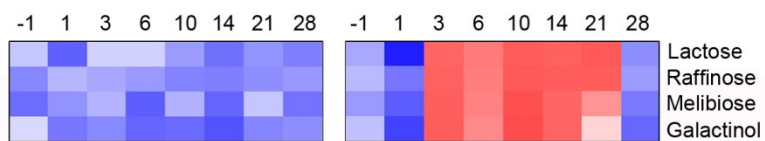

Supplement: Figure S4 — S . Typhimurium infection induces metabolite changes in the gut environment. GC-MS metabolite analysis of soluble factors in fecal samples revealed that (A,B) the profile of metabolites in infected animals (right panel, red) is distinct from that of control animals (left panel, black). Chromatograms presented here, with early time points in the foreground and later time points in the background, demonstrate significantly increased abundance of specific metabolites by day 3 post infection in Salmonella-infected animals. (A) represents experimental group 1 data, while (B) represents experimental group 2 data. The same scaling for the y-axis was applied to all chromatograms for relative chromatographic comparison. A direct comparison of metabolite profiles at day 14 (C) shows the appearance of metabolites in infected samples (box). These peaks represent sugars which accumulate in the infected gut, in the absence of commensal microorganisms that normally metabolize these structures. (D) Intensity of these peaks in infected animals is represented in a heat map. Data shown in (C) and (D) are representative of experimental group 2. Heat map shows intensity data following log 2 and Z-score transformation, where red is more intense and blue is less intense. (PDF) [file pone.0067155.s004.pdf]

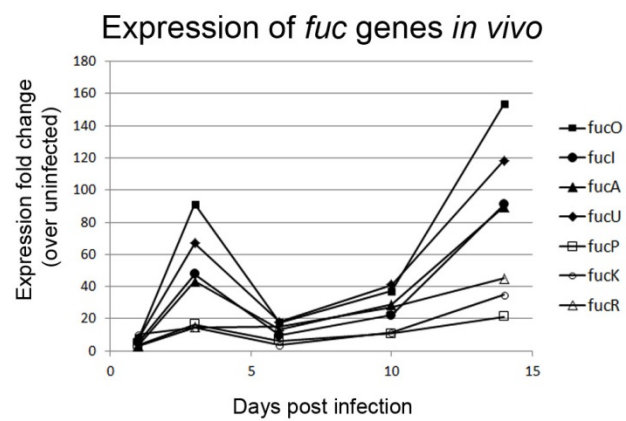

Supplement: Figure S5 — Expression of fuc genes in vivo following Salmonella infection. qRT-PCR analysis revealed increased expression of genes in the fuc regulon of S. Typhimurium during infection. Data presented as fold change over expression in uninfected animals. (PDF) [file pone.0067155.s005.pdf]

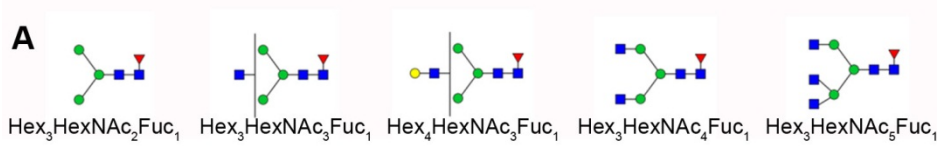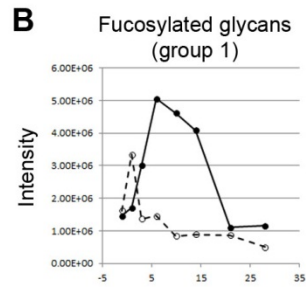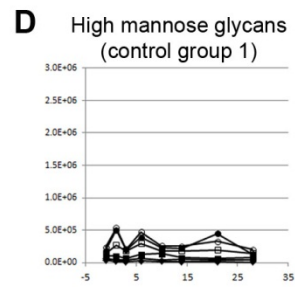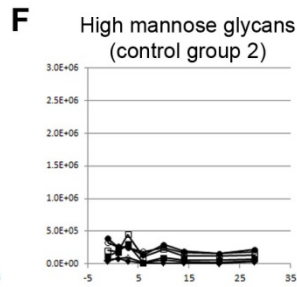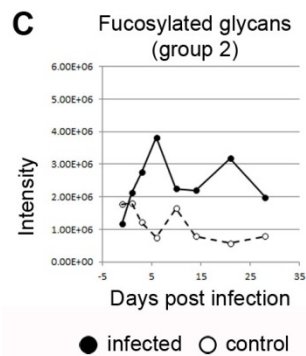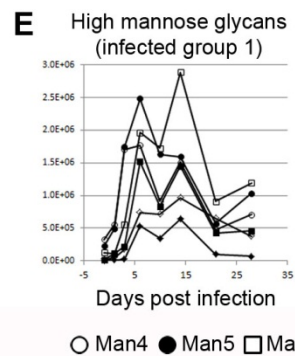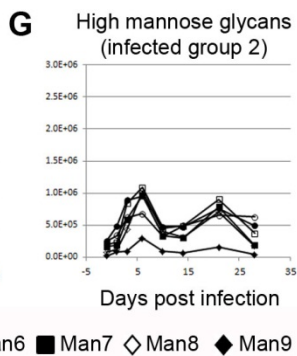

Supplement: Figure S6 — Fucose and high mannose glycan detection in individual biological replicates. (A) Five fucosylated glycan moieties that were observed at each time point were selected for analysis. These fucosylated glycans increased during infection in both (B) experimental group 1 and (C) experimental group 2, in comparison to control animals. Data from infected animals are represented with closed circles and a solid line, while data from control animals are shown as open circles with a dashed line. High mannose series glycans are present at low levels in control samples from experimental group 1 (D) and experimental group 2 (F), and are increased in infected animals in experimental group 1 (E) and experimental group 2 (G). Symbols represent the following species in parts C-F: Man4 (open circles), Man5 (solid circles), Man6 (open squares), Man7 (solid squares), Man8 (open diamonds), Man9 (solid diamonds). Intensity values represent averaged data from pooled fecal samples, by experimental group. (PDF) [file pone.0067155.s006.pdf]

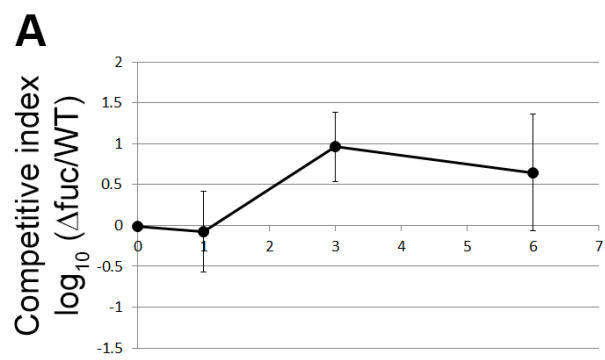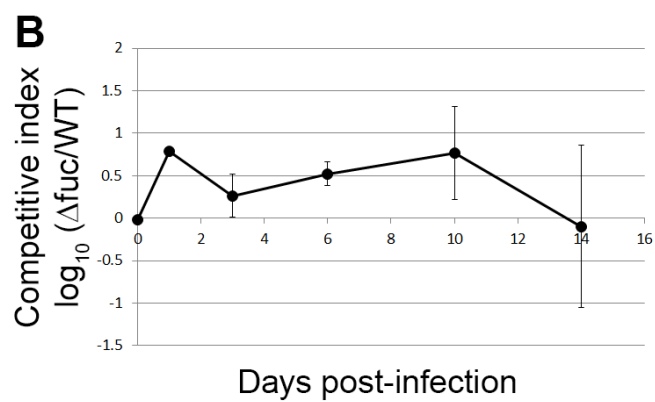

Supplement: Figure S7 — Inability to utilize fucose demonstrates in vivo phenotype. WT and Δfuc strains were mixed at a 1∶1 ratio and used to orally infect 129/SvJ mice. Quantification of each strain in the shed feces demonstrated a slight growth advantage in vivo of the Δfuc strain. Two replicate experiments were performed, shown in (A) and (B). Each filled circle represents the average competitive index value, calculated from three groups (with three mice per group). Error bars represent the standard deviation values. (PDF) [file pone.0067155.s007.pdf]
