# Supplementary material for: A Multi-Omic View of Host-Pathogen-Commensal Interplay in Salmonella-Mediated Intestinal Infection
Source: PLoS One. 2013 Jun 26;8(6):e67155. doi: 10.1371/journal.pone.0067155 (PMC3694140; doi:10.1371/journal.pone.0067155)
Supplement: Table S1 — All filter-passing protein identifications. (A) Mouse-derived, (B) microbiota-derived, and (C) Salmonella-derived protein identifications. (PDF) [file pone.0067155.s008.pdf]

Table S1A: Total filter-passing mouse protein identifications

				Biological replicate 1															
				CONTROL								INFECTED							
gi number	Reference	Protein description	DAY ->	-1	1	3	6	10	14	21	28	-1	1	3	6	10	14	21	28
gi|10946884	NP_067456.1	trehalase precursor																	
gi|110347469	NP_031402.3	alpha-2-macroglobulin precursor													1		1	1	1
gi|110347479	NP_031458.2	intestinal-type alkaline phosphatase precursor		3	3	2	5	4	4	3	5	2	4	3	3	2	3	2	3
gi|110347564	NP_031778.2	ceruloplasmin isoform b																	
gi|11037800	NP_067623.1	cell surface A33 antigen precursor													9	4	3	1	2
gi|110556625	NP_997091.3	calcium-activated chloride channel regulator 4		1	4		1		2	1	1	2	3	1	3	5	3	1	1
gi|110625761	NP_081406.1	AFG3-like protein 2																	
gi|111185905	NP_031990.2	ecotropic viral integration site 5 protein							1									1	2
gi|111607447	NP_032430.2	integrin beta-2																	
gi|112181182	NP_031773.2	cytochrome c oxidase subunit 5A, mitochondrial precursor																	
gi|112181302	NP_001019787.2	putative GTP-binding protein Parf													1				1
gi|112293264	NP_031978.2	protein disulfide-isomerase A3 precursor																	
gi|112293275	NP_032558.2	epithelial cell adhesion molecule																	
gi|113195678	NP_034814.2	lysosome-associated membrane glycoprotein 1																	
gi|113195684	NP_034799.2	keratin, type II cytoskeletal 6B						1	1	2			2	1	2	2		2	
gi|114052444	NP_056594.2	neutrophil elastase precursor														1		1	
gi|114145487	NP_059503.2	ras-related GTP-binding protein C																	
gi|114145561	NP_112447.2	keratin, type II cytoskeletal 8																	
gi|114158675	NP_079705.2	leukocyte elastase inhibitor A					1	1	2					2	2	3	1	1	2
gi|114326446	NP_071855.2	myosin-9 isoform 1																	
gi|114326546	NP_075907.2	phosphoglycerate mutase 1																	
gi|115648048	NP_035343.2	receptor-type tyrosine-protein phosphatase F precursor																	
gi|116089273	NP_032138.3	rab GDP dissociation inhibitor beta																	
gi|118601068	NP_032869.2	prolactin-inducible protein homolog precursor																	
gi|119120879	NP_033231.2	solute carrier family 3, member 1					1		1			1		1	6	4	1	1	1
gi|119392092	NP_444309.2	solute carrier family 15 member 1		1			1		2				2					2	
gi|119433657	NP_835585.3	histone H2A type 2-B																	
gi|121674797	NP_032943.2	palmitoyl-protein thioesterase 1 precursor																	
gi|124053457	NP_034950.2	multiple PDZ domain protein													1		1	1	
gi|124244098	NP_031459.3	embryonic-type alkaline phosphatase precursor						1					1						1
gi|124248572	NP_035822.2	prefoldin subunit 3																	
gi|124249111	NP_001074245.1	coxsackie adenovirus receptor-like		1			1								1		1		
gi|124339826	NP_034608.2	heat shock 70 kDa protein 1B																	
gi|124430537	NP_001074443.1	sorcin isoform 1																	
gi|124486895	NP_001074743.1	6-phosphogluconate dehydrogenase, decarboxylating																	
gi|124487227	NP_001074876.1	phospholipase B1, membrane-associated isoform 1		1	1								7		3	1		2	
gi|124487275	NP_001074606.1	sucrase-isomaltase, intestinal		6	3	3	4	3	9	2	3	4	6	8	5	2	4	2	3
gi|124487297	NP_001074547.1	lactase-phlorizin hydrolase preproprotein						1	5						2	3	2		
gi|124487323	NP_001074551.1	intestinal alkaline phosphatase		13	10	3	10	6	8	8	8	8	9	8	9	5	4	6	5
gi|124487419	NP_034798.2	keratin, type II cytoskeletal 2 epidermal		1															
gi|124517663	NP_034860.2	annexin A1														2			
gi|125628640	NP_808278.2	dual oxidase 2													1				
gi|126032329	NP_034236.2	elongation factor 1-alpha 1																	
gi|126116585	NP_032499.2	keratin, type II cytoskeletal 1				1					1								
gi|126518317	NP_033908.2	complement C3																	
gi|12746442	NP_075547.1	selenocysteine-specific elongation factor							1	1									
gi|12963555	NP_075664.1	omega-amidase NIT2																	
gi|12963733	NP_076046.1	choline transporter-like protein 4												1					
gi|12963737	NP_076054.1	exportin-2																	
gi|132626295	NP_032427.2	integrin alpha-M isoform 2 precursor																	
gi|13374559	NP_075671.1	chymotrypsin-like																	
gi|133778951	NP_032437.3	CUB and zona pellucida-like domain-containing protein 1		2			1	2		1	1	1	1	2		1		2	1
gi|133778955	NP_034510.3	H-2 class I histocompatibility antigen, L-D alpha chain precursor																	

gi|133778994	NP_573476.2	X-prolyl aminopeptidase (aminopeptidase P) 2, membrane-bound isoform 1		1	2		1					1	6	1	3	3	2	1	1
gi|13384886	NP_079745.1	colipase precursor						1	1										2
gi|13385840	NP_080612.1	hypothetical protein LOC67719 precursor		29		17	9	6	16	12	11	11	4	20	23	14	19	36	47
gi|13385916	NP_080695.1	chymotrypsin-like elastase family, member 3B		4		4	4	1	2	4	6	1	7	3	7	47	21	14	26
gi|134031994	NP_032970.2	proteasome subunit alpha type-2																	
gi|134288869	NP_033893.2	ADP-ribosyl cyclase 2																	
gi|134288917	NP_084514.2	cytoplasmic dynein 1 heavy chain 1																	
gi|14192922	NP_033738.1	actin, alpha cardiac muscle 1		1		3		1	2			1							2
gi|145301549	NP_001077424.1	hemoglobin alpha, adult chain 2																	
gi|145553963	NP_075022.2	betaine--homocysteine S-methyltransferase 2																	
gi|147901863	NP_032612.2	meprin A subunit beta precursor		1	1			1	1		2	1	1	2	2	3		1	
gi|148229673	NP_001079003.1	hypothetical protein LOC546335															1		
gi|148277039	NP_783327.2	alpha-2-macroglobulin-P precursor																	
gi|148277591	NP_058087.2	syntenin-1 isoform 2																	
gi|148747546	NP_035588.2	serine protease inhibitor A3K precursor			1												1		
gi|149234244	XP_001478035.1	PREDICTED: similar to Capping protein (actin filament) muscle Z-line, alpha 1																	
gi|149240743	XP_001476708.1	PREDICTED: hypothetical protein																	
gi|149246448	XP_001472880.1	PREDICTED: similar to RIKEN cDNA 1810030J14 gene													6	2	2	3	2
gi|149248895	XP_001475565.1	PREDICTED: similar to Glyceraldehyde-3-phosphate dehydrogenase (GAPDH) isoform 1													2	1	1	1	
gi|149249272	XP_001476087.1	PREDICTED: similar to Glyceraldehyde-3-phosphate dehydrogenase (GAPDH)																	
gi|149249466	XP_001477320.1	PREDICTED: hypothetical protein																	
gi|149249766	XP_923442.2	PREDICTED: similar to fusion protein: ubiquitin (bases 43_513); ribosomal protein S27a (bases 217_532)														1			
gi|149250381	XP_001475400.1	PREDICTED: similar to cadherin 22																	
gi|149250554	XP_001472375.1	PREDICTED: hypothetical protein							1						3	1	2		
gi|149250606	XP_999253.2	PREDICTED: hypothetical protein		1															
gi|149251724	XP_889997.3	PREDICTED: similar to secretory protein														1			
gi|149251776	XP_001476579.1	PREDICTED: similar to Nucleophosmin (NPM) (Nucleolar phosphoprotein B23) (Numatrin) (Nucleolar protein NO38)														2	1	1	
gi|149252769	XP_001477261.1	PREDICTED: hypothetical protein											1						
gi|149253386	XP_001471566.1	PREDICTED: hypothetical protein																	
gi|149253488	XP_001473173.1	PREDICTED: similar to Tubulin, beta 4																	
gi|149253544	XP_001479464.1	PREDICTED: similar to cytochrome c				3													
gi|149254235	XP_001473686.1	PREDICTED: hypothetical protein											1		1	2	2	3	
gi|149254414	XP_001479012.1	PREDICTED: similar to Muc3 protein														1			
gi|149254987	XP_001478026.1	PREDICTED: similar to trypsinogen 15							1							1	1		
gi|149255095	XP_001476664.1	PREDICTED: similar to trypsinogen 15			1		1	1		1				2					
gi|149255293	XP_001480324.1	PREDICTED: similar to Murinoglobulin 1													1			1	
gi|149255339	XP_996033.2	PREDICTED: similar to calmodulin																	
gi|149255578	XP_001476107.1	PREDICTED: similar to immunoglobulin kappa chain		1	2		1							2	4	1		1	2
gi|149255583	XP_989825.2	PREDICTED: hypothetical protein																	
gi|149255600	XP_001476873.1	PREDICTED: similar to light chain of the monoclonal antibody MST2																1	
gi|149255605	XP_001477220.1	PREDICTED: similar to [Human Ig rearranged gamma chain mRNA, V-J-C region and complete cds.]																	
gi|149255611	XP_001477384.1	PREDICTED: similar to [Human Ig rearranged gamma chain mRNA, V-J-C region and complete cds.]							1		1								
gi|149255615	XP_001477602.1	PREDICTED: similar to immunoglobulin kappa-chain																	
gi|149255637	XP_001476714.1	PREDICTED: similar to Chain L, Structural Basis Of Antigen Mimicry In A Clinically Relevant Melanoma Antigen System isoform 1		8	10	4	5	8	8	9	7	6	5	11	17	8	11	14	9
gi|149255823	XP_001480334.1	PREDICTED: similar to Cofilin-1 (Cofilin, non-muscle isoform)																	
gi|149256882	XP_001478116.1	PREDICTED: similar to peptidoglycan recognition protein																1	
gi|149256898	XP_001471707.1	PREDICTED: hypothetical protein							1				1						
gi|149257837	XP_001471659.1	PREDICTED: hypothetical protein																	
gi|149258165	XP_001472728.1	PREDICTED: similar to beta-globin isoform 1																	
gi|149258188	XP_001471843.1	PREDICTED: hypothetical protein																	

gi|149260099	XP_001474833.1	PREDICTED: similar to Annexin A2 (Annexin II) (Lipocortin II) (Calpactin I heavy chain)<br>(Chromobindin-8) (p36) (Protein I) (Placental anticoagulant protein IV) (PAP-IV)																		
gi|149261589	XP_486478.3	PREDICTED: similar to ferritin light chain 2																		
gi|149263303	XP_204772.4	PREDICTED: similar to endothelial monocyte-activating polypeptide																		
gi|149263766	XP_001472141.1	PREDICTED: hypothetical protein		9	8	2	8	5	5	6	5	6	11	5	7	7	8	11	9
gi|149263798	XP_001474075.1	PREDICTED: similar to immunoglobulin epsilon		19	11		5	8	12	5	20	2	9	4	11	6	11	25	37
gi|149264032	XP_001475950.1	PREDICTED: similar to Hist1h2bj protein												1					
gi|149264124	XP_920080.3	PREDICTED: hypothetical protein																	
gi|149264591	XP_001477449.1	PREDICTED: hypothetical protein													1		1		
gi|149265958	XP_001474586.1	PREDICTED: similar to purine nucleoside phosphorylase																	
gi|149267864	XP_001479188.1	PREDICTED: similar to apolipoprotein D																	
gi|149269211	XP_908790.3	PREDICTED: similar to class Ib MHC antigen Qa-2 isoform 2														1			
gi|149269220	XP_001473590.1	PREDICTED: similar to MHC H2-K 24 polypeptide isoform 1																	
gi|149269696	XP_001475963.1	PREDICTED: hypothetical protein																	
gi|149270372	XP_001474350.1	PREDICTED: hypothetical protein																	
gi|149270446	XP_001478257.1	PREDICTED: similar to ribosomal protein S7																	
gi|149270512	XP_001479065.1	PREDICTED: hypothetical protein																	
gi|149271597	XP_001473196.1	PREDICTED: similar to spermine synthase																	
gi|149272386	XP_001477701.1	PREDICTED: similar to thymosin beta-4																	
gi|149274531	XP_001473875.1	PREDICTED: similar to heterogeneous nuclear ribonucleoprotein A2/B1																	
gi|149274769	XP_001478965.1	PREDICTED: similar to VH coding region																	
gi|149275191	XP_001473825.1	PREDICTED: hypothetical protein																	
gi|15022803	NP_035583.1	serine (or cysteine) proteinase inhibitor, clade B, member 9c isoform 2																	
gi|153945724	NP_033171.2	neuroplastin precursor																	
gi|153945747	NP_032672.2	murinoglobulin-2 precursor																	
gi|154090941	NP_997648.2	keratin, type I cytoskeletal 42																	
gi|154240724	NP_598766.2	ectonucleotide pyrophosphatase/phosphodiesterase family member 3			2				2		1		1						
gi|157951678	NP_033932.2	carbonic anhydrase 6 precursor		1						1	1		1	1					
gi|15826844	NP_035584.1	serine (or cysteine) proteinase inhibitor, clade B, member 6b																	
gi|160333613	NP_958751.2	serine (or cysteine) proteinase inhibitor, clade B, member 3C																	
gi|160333652	NP_081496.2	protease, serine, 32													1				
gi|160333869	NP_035258.2	pancreatic lipase-related protein 2		1						1			16	1	35	6	17	12	4
gi|160358829	NP_059067.2	hemopexin precursor																	
gi|160707976	NP_036125.4	protein piccolo isoform 1																	
gi|161016799	NP_038499.2	annexin A4											1			1		1	1
gi|161016822	NP_001104301.1	aspartyl aminopeptidase isoform a																	
gi|161016833	NP_031902.2	dipeptidase 1 precursor							1										
gi|162138915	NP_034079.2	carnitine O-palmitoyltransferase 2, mitochondrial precursor																	
gi|163310765	NP_033784.2	serum albumin precursor																	1
gi|163914390	NP_033906.2	plasma protease C1 inhibitor precursor																	
gi|16418335	NP_084072.1	leucine-rich alpha-2-glycoprotein																1	
gi|164519050	NP_032720.2	neutrophilic granule protein																2	2
gi|164565419	NP_001106839.1	carcinoembryonic antigen-related cell adhesion molecule 2 isoform a												1	1	2			1
gi|165932341	NP_291090.2	chymotrypsin-like elastase family, member 1																1	2
gi|167004347	NP_001107794.1	cadherin-related family member 5 isoform 1											2		2	2	1		
gi|16716569	NP_444473.1	protease, serine, 1			1								1	1					1
gi|169790797	NP_001116075.1	Fc fragment of IgG binding protein		1	2		1		1		2		1		1	2	4	3	2
gi|169790977	NP_001116119.1	major urinary protein 10																	
gi|170172530	NP_690052.2	immunoglobulin J chain precursor									1		1	1	2	2	2	1	
gi|170650599	NP_613057.2	elongation factor G, mitochondrial precursor					1	2				1							
gi|17647499	NP_058652.1	hemoglobin subunit beta-2																	
gi|18252782	NP_543120.1	antithrombin-III precursor											1						
gi|187283027	NP_001119790.1	hypothetical protein LOC242711		1	1		2		1	3	2		1	5	8	5	5	2	5
gi|188219582	NP_036067.2	glucosamine-6-phosphate isomerase 1																	
gi|189491671	NP_766081.1	protein diaphanous homolog 2										1							
gi|190684696	NP_033535.2	villin-1																	
gi|194328695	NP_035766.2	phospholipid scramblase 1																	

gi|19527028	NP_598569.1	vigilin																	
gi|19527078	NP_598623.1	fibrinogen gamma chain precursor																2	1
gi|19527082	NP_598628.1	epidermal growth factor receptor kinase substrate 8-like protein 3																	
gi|19527104	NP_598649.1	acid sphingomyelinase-like phosphodiesterase 3b precursor															1		
gi|20330802	NP_598738.1	serotransferrin precursor																1	
gi|209862915	NP_001129527.1	CD82 antigen isoform 2																	
gi|21105853	NP_631937.1	placenta-specific gene 8 protein													1				
gi|21312316	NP_082492.1	prostate stem cell antigen precursor			3		1					1	5						5
gi|213417962	NP_067369.2	Z-DNA-binding protein 1 isoform 1																	
gi|21362335	NP_653120.1	carboxypeptidase A5 precursor									6								
gi|21450325	NP_659172.1	flavin reductase																	
gi|21489935	NP_058654.1	keratin, type I cytoskeletal 14																	
gi|21704144	NP_663544.1	S-adenosylmethionine synthase isoform type-2				1		1		1	1			2		1			
gi|21704206	NP_663578.1	carboxylesterase 2 precursor																	
gi|22122475	NP_666122.1	tetraspanin 8		3	1		1		2	1		3	2	2	7	3	2	2	3
gi|22122641	NP_666231.1	niban-like protein 1																	
gi|22164776	NP_666175.1	keratin, type II cytoskeletal 79											1		1				
gi|22165388	NP_666261.1	hypothetical protein LOC230579											1						
gi|22219434	NP_671734.1	nuclear receptor-binding protein																	
gi|224809376	NP_067620.3	nicastrin precursor							1						1				
gi|224922803	NP_034404.3	glycerol-3-phosphate dehydrogenase, mitochondrial precursor																	
gi|225007644	NP_001139360.1	zinc finger protein ZFAT isoform 1																	
gi|225637487	NP_032512.2	aminopeptidase N		18	5	8	7	7	17	9	5	11	10	23	17	10	9	12	3
gi|225690549	NP_796240.3	ribosome-releasing factor 2, mitochondrial isoform 1																	
gi|225735649	NP_001139592.1	sulfated glycoprotein 1 isoform A preproprotein																2	1
gi|226246531	NP_112467.2	phosphoglycerate kinase 2																	
gi|226823250	NP_034954.2	myeloperoxidase precursor															1	1	
gi|226874906	NP_033562.3	14-3-3 protein epsilon																	
gi|226874935	NP_034712.2	inter-alpha-trypsin inhibitor heavy chain H2																	
gi|226958349	NP_033441.2	triosephosphate isomerase																1	
gi|226958351	NP_038762.1	sodium/potassium-transporting ATPase subunit alpha-4														2			
gi|226958497	NP_035448.2	serum amyloid P-component precursor													14	4	11	17	5
gi|227116347	NP_001153027.1	platelet glycoprotein 4													1			1	
gi|227499103	NP_663392.2	aminopeptidase B isoform 1																	
gi|227908835	NP_835180.2	probable carboxypeptidase PM20D1 precursor																	
gi|236468117	NP_001153622.1	amylase 2a4		1				1	2	2									2
gi|237874196	NP_001153858.1	dynein, axonemal, heavy chain 7B									1								
gi|23943876	NP_081194.1	zymogen granule membrane protein 16 precursor		24	1	9	19	9	20	20	9	23	7	9	6	9	14	11	8
gi|23956140	NP_079914.1	glycerophosphodiester phosphodiesterase domain-containing protein 1																	
gi|23956196	NP_081487.1	annexin A13																	
gi|23956222	NP_076224.1	actin-related protein 3																	
gi|242332572	NP_057930.2	ras GTPase-activating-like protein IQGAP1																	
gi|242397450	NP_035178.1	paired basic amino acid cleaving system 4																	
gi|24496768	NP_038949.2	serine protease 30 precursor																	
gi|25141335	NP_082045.1	copine-3														1		1	
gi|251823804	NP_660108.2	chitinase-3-like protein 4 precursor																	
gi|251823824	NP_082166.3	sushi domain-containing protein 2 isoform 1																	
gi|253970410	NP_653127.2	hypothetical protein LOC78354					1		1						1		1	2	3
gi|253970442	NP_997125.2	Niemann-Pick C1-like protein 1 precursor																	
gi|254281348	NP_034022.2	chitinase-3-like protein 3 precursor														1	1	1	
gi|254540166	NP_071705.3	78 kDa glucose-regulated protein precursor																	
gi|254692909	NP_001013044.2	transcription termination factor 2																	
gi|255069715	NP_077754.3	cytosol aminopeptidase									1								
gi|255522937	NP_079859.2	chymotrypsinogen B precursor		9	5	3	6	4	9	8	3	3	5	1	1	1	5	13	9
gi|255683322	NP_766413.2	probable G-protein coupled receptor 128 precursor																	
gi|256222930	NP_663549.2	coiled-coil domain-containing protein 136																	
gi|257467625	NP_001158127.1	Fc fragment of IgG binding protein-like			2						2								1
gi|258547152	NP_001158149.1	programmed cell death 6-interacting protein isoform 1																	

gi|260304980	NP_001159443.1	predicted gene 5803																	
gi|262073053	NP_081515.2	hypothetical protein LOC69864																	
gi|268836255	NP_573461.2	glycogen phosphorylase, liver form																	
gi|269308203	NP_082776.2	calreticulin-3 isoform 1																	
gi|27151739	NP_032780.2	odorant binding protein Ia			2														
gi|27229028	NP_080265.2	pancreatic secretory granule membrane major glycoprotein GP2					1												
gi|27370012	NP_766288.1	CAAX prenyl protease 1 homolog																	
gi|27370038	NP_766299.1	GTP-binding protein GUF1 homolog								1	1								
gi|27754136	NP_075675.2	acidic mammalian chitinase precursor		4	1	3	5	2	1	4	6		3	2	4	2		1	1
gi|282165827	NP_001028536.2	protocadherin 24		3	2		1	1		3	2	2	6	1	9	9	2		
gi|28316756	NP_783589.1	histone cluster 1, H2aa													1		2		
gi|283483997	NP_001164474.1	maltase-glucoamylase		6	4		3	1	8	1	1	4	1	2	1	2		5	
gi|294489324	NP_001170938.1	MHC class Ib T9																	
gi|294610643	NP_076055.2	mucin-2			1		1			2	1	1							1
gi|294997230	NP_996988.2	histocompatibility 2, class II antigen A, beta 1 precursor														3	1	2	4
gi|29789211	NP_082202.1	carboxypeptidase A4 precursor								1									
gi|29789343	NP_598677.1	eukaryotic translation initiation factor 3 subunit B																	
gi|29789389	NP_766172.1	inositol-tetrakisphosphate 1-kinase																	
gi|30410738	NP_848499.1	hypothetical protein LOC229688		1							1					1			
gi|30425250	NP_780706.1	beta-actin-like protein 2				1													
gi|30519911	NP_848713.1	transgelin-2																	
gi|30725780	NP_849251.1	protein MAL2															1		
gi|31542362	NP_031672.2	ADP-ribosyl cyclase 1																	
gi|31543113	NP_032905.2	plastin-2																	
gi|31543255	NP_032630.2	neprilysin		6	7			2	11		1	4	6	4	7	9	6	2	1
gi|31543942	NP_033528.3	vinculin																	
gi|31543974	NP_061223.2	14-3-3 protein beta/alpha																	
gi|31543976	NP_061359.2	14-3-3 protein gamma																	
gi|31560030	NP_077745.2	tropomyosin alpha-1 chain isoform 3																	
gi|31560433	NP_059500.2	sorting nexin-3																	
gi|31560677	NP_032548.2	lactotransferrin precursor																	
gi|31560731	NP_031534.2	V-type proton ATPase catalytic subunit A																	
gi|31980868	NP_034769.4	kallikrein-1 precursor																	
gi|31981542	NP_035308.2	myeloblastin precursor																	
gi|31981562	NP_035229.2	pyruvate kinase isozymes M1/M2																	
gi|31981570	NP_035212.2	polymeric immunoglobulin receptor precursor		1	1				1						1		4	4	5
gi|31981716	NP_034508.2	histocompatibility 2, class II antigen A, alpha precursor															1	1	1
gi|31981890	NP_033865.2	beta-2-microglobulin precursor																	
gi|31982030	NP_598557.3	rho GDP-dissociation inhibitor 1																	
gi|31982171	NP_032671.2	murinoglobulin-1 precursor																1	1
gi|31982186	NP_032643.2	malate dehydrogenase, mitochondrial precursor																	
gi|31982199	NP_032611.2	meprin A subunit alpha							1					1					
gi|33563236	NP_031512.1	rho GDP-dissociation inhibitor 2																	
gi|33563240	NP_033736.1	actin, alpha skeletal muscle																	
gi|33563252	NP_034326.1	fibrinogen, alpha polypeptide isoform 2														1		1	1
gi|33859474	NP_031784.1	complement receptor type 2														1		1	
gi|33859640	NP_035658.1	transaldolase																	
gi|33859686	NP_079976.1	phosphoglucomutase-2																	
gi|33859809	NP_862897.1	fibrinogen beta chain precursor														1			1
gi|34328436	NP_081138.2	CD177 antigen precursor																4	
gi|34538600	NP_904330.1	cytochrome c oxidase subunit I																	
gi|34538601	NP_904331.1	cytochrome c oxidase subunit II																	
gi|34915988	NP_033898.1	basigin isoform 1		1			1									2	1		
gi|36031132	NP_031530.2	sarcoplasmic/endoplasmic reticulum calcium ATPase 1																	
gi|37674236	NP_081201.2	pancreatic triacylglycerol lipase		33	12	13	30	9	32	26	8	10	36	20	8	8	21	24	43
gi|38076713	XP_357312.1	PREDICTED: similar to ferritin light chain 2																	
gi|40556608	NP_032328.2	heat shock protein HSP 90-beta																	
gi|42415475	NP_035162.1	protein disulfide-isomerase precursor																	

gi|45504394	NP_034708.1	integrin beta-1 precursor																	
gi|46559389	NP_997507.1	angiotensin-converting enzyme isoform 1																	
gi|50345978	NP_001002012.1	heat shock-related 70 kDa protein 2																	
gi|51010909	NP_001003405.1	trypsin 5		2		2	2	1	1	1		1	1	1	2		1	1	2
gi|51491845	NP_001003908.1	clathrin heavy chain 1																	
gi|51765047	XP_486246.1	PREDICTED: similar to Tubulin, alpha 3c isoform 1																	
gi|54312076	NP_079626.2	carboxypeptidase A1 precursor		35	4	18	7	14	13	28	54	9	14	32		1		2	4
gi|56090544	NP_001007553.1	intelectin-1b precursor																	
gi|56550071	NP_083982.1	pancreatic carboxypeptidase B1		1	2		1		5	3	2		7	1	2	2	1	7	9
gi|57222282	NP_001009546.1	N-acetylated-alpha-linked acidic dipeptidase-like protein		3			2	2	3	1	3	5	5	2	1	1	1		
gi|61097906	NP_598917.1	alpha-actinin-1																	
gi|61743961	NP_033773.1	AHNAK nucleoprotein isoform 1																	
gi|6671509	NP_031419.1	actin, cytoplasmic 1			2	2			2	1	1	2							
gi|6671539	NP_031464.1	fructose-bisphosphate aldolase A isoform 2																	
gi|6671664	NP_031623.1	calnexin precursor																	
gi|6671672	NP_031630.1	F-actin-capping protein subunit alpha-2																	
gi|6671678	NP_031633.1	carbonic anhydrase 4 precursor																	
gi|6671746	NP_031714.1	cofilin-2																	
gi|6677703	NP_033068.1	lithostathine-1 precursor													7		1	8	
gi|6677705	NP_033069.1	lithostathine-2 precursor													14	3	3	10	
gi|6677837	NP_033140.1	protein S100-A9														2	1	4	
gi|6678079	NP_033269.1	alpha-1-antitrypsin 1-1 precursor																	
gi|6678085	NP_033272.1	alpha-1-antitrypsin 1-4 precursor																	
gi|6678097	NP_033280.1	serpin B6 isoform b																	
gi|6678329	NP_033399.1	protein-glutamine gamma-glutamyltransferase 2																	
gi|6678439	NP_033456.1	anionic trypsin-2 precursor		17	4	8	10	9	8	11	10	8	14	10	7	5	5	5	2
gi|6678581	NP_033541.1	wiskott-Aldrich syndrome protein homolog							1										
gi|6679293	NP_033428.1	peptidoglycan recognition protein 1 precursor															1		2
gi|6679383	NP_032904.1	alpha-2-antiplasmin precursor																	
gi|6679439	NP_032933.1	peptidyl-prolyl cis-trans isomerase A																	
gi|6679509	NP_032979.1	Psp							1						1			2	2
gi|6679653	NP_031960.1	glutamyl aminopeptidase		5	2	1	1		4	2	1	6	6	3	8	11	4	2	3
gi|6679939	NP_032111.1	glyceraldehyde-3-phosphate dehydrogenase, testis-specific																	
gi|6679995	NP_032142.1	gamma-glutamyltranspeptidase 1																	1
gi|6680231	NP_032279.1	high mobility group protein B3																	
gi|6680441	NP_032401.1	gastrotropin											1						
gi|6680586	NP_032484.1	serine protease inhibitor A3C precursor											1				1		
gi|6680636	NP_031424.1	adenosine deaminase															1		4
gi|6680710	NP_031498.1	aquaporin-1																	
gi|6680836	NP_031617.1	calreticulin precursor																	
gi|6681297	NP_031945.1	chymotrypsin-like elastase family member 2A precursor				1									1	1	2	3	5
gi|67010039	NP_001019869.1	carboxypeptidase A2 precursor		3		2	1		1	2	5	2	1	6		2	1	5	2
gi|6753060	NP_033803.1	annexin A5																	
gi|6753138	NP_033851.1	sodium/potassium-transporting ATPase subunit beta-1							1				1	1	2	2	2	1	
gi|6753244	NP_033920.1	calmodulin																	
gi|6753322	NP_033967.1	T-complex protein 1 subunit delta																	
gi|6753428	NP_034027.1	creatine kinase U-type, mitochondrial precursor		2		1	1	1				1			1	1			1
gi|6753674	NP_034204.1	dipeptidyl peptidase 4 isoform 1		1	1				2		1				3		1	1	
gi|6753798	NP_034298.1	prothrombin																	
gi|6753912	NP_034369.1	ferritin heavy chain																	
gi|6754254	NP_034610.1	heat shock protein HSP 90-alpha																	
gi|6754388	NP_034714.1	intelectin-1a precursor																	
gi|6754480	NP_034792.1	keratin, type I cytoskeletal 13																	
gi|6754534	NP_034837.1	galectin-6																	
gi|6754576	NP_034869.1	mucin-13 precursor							1				1						
gi|6754658	NP_034907.1	Golli-Mbp isoform 1		1		1	5				1		1	1			1		
gi|6754976	NP_035164.1	peroxiredoxin-1																	
gi|6754980	NP_035166.1	regenerating islet-derived protein 3-beta precursor												2	6	3	6	5	6

gi|6755090	NP_035237.1	phospholipase A2 precursor			1														
gi|6755100	NP_035249.1	proliferation-associated protein 2G4																	
gi|6755112	NP_035255.1	phospholipid transfer protein precursor						1											
gi|6755144	NP_035280.1	galectin-3-binding protein precursor																	
gi|6755210	NP_036005.1	26S proteasome non-ATPase regulatory subunit 13																	
gi|6755256	NP_035354.1	glycogen phosphorylase, muscle form																	
gi|6755308	NP_035389.1	regenerating islet-derived protein 3-alpha precursor													2	1	3	3	5
gi|6755310	NP_035390.1	regenerating islet-derived protein 3-gamma precursor													1	2	1	4	3
gi|6755364	NP_035424.1	activated RNA polymerase II transcriptional coactivator p15														1			
gi|6755372	NP_036182.1	40S ribosomal protein S3																	
gi|6755863	NP_035761.1	endoplasmin																	
gi|6755963	NP_035824.1	voltage-dependent anion-selective channel protein 1																	
gi|6755965	NP_035825.1	voltage-dependent anion-selective channel protein 2															2		
gi|6755967	NP_035826.1	voltage-dependent anion-selective channel protein 3																	
gi|6756037	NP_035868.1	14-3-3 protein eta																	
gi|68226731	NP_032968.2	puromycin-sensitive aminopeptidase																	
gi|6996913	NP_031611.1	annexin A2																	
gi|70778976	NP_032854.2	phosphoglycerate kinase 1																	
gi|71043961	NP_075822.3	trypsinogen 7																	
gi|7110683	NP_035174.1	phosphoenolpyruvate carboxykinase, cytosolic [GTP]																	
gi|7304963	NP_038913.1	chloride intracellular channel protein 4																	
gi|7305189	NP_038593.1	integrin alpha-3																	
gi|7305453	NP_038678.1	protein S100-A8																2	
gi|7305599	NP_038725.1	transthyretin								1		1							
gi|75677435	NP_031463.2	fatty aldehyde dehydrogenase																	
gi|76443665	NP_001029047.1	chymotrypsin-C precursor		85	35	42	95	76	71	84	69	83	86	38	46	29	74	64	72
gi|76881807	NP_033270.3	alpha-1-antitrypsin 1-2 precursor			1				1	1	1	2	1		2	1			
gi|7710018	NP_038543.1	erythrocyte band 7 integral membrane protein																	
gi|7949064	NP_032482.1	kallikrein 1-related peptidase b5 precursor		2							2		1						
gi|82796081	XP_928726.1	PREDICTED: hypothetical protein		1										1					
gi|82799602	XP_913793.1	PREDICTED: similar to Glyceraldehyde-3-phosphate dehydrogenase																	
gi|82802624	XP_920189.1	PREDICTED: similar to hCG2001479		3	1	2	1	1	3	1	2	3	1	3		1		1	
gi|82882188	XP_922713.1	PREDICTED: similar to intelectin																	
gi|82895363	XP_919532.1	PREDICTED: hypothetical protein																	
gi|82935925	XP_913239.1	PREDICTED: similar to myosin, heavy polypeptide 3, skeletal muscle, embryonic																	1
gi|82998325	XP_904967.1	PREDICTED: hypothetical protein isoform 7																	
gi|83008262	XP_913818.1	PREDICTED: similar to Ab 414.2 heavy chain variable and joining region						1	1		1								1
gi|83014618	XP_909851.1	PREDICTED: similar to Igh protein		1			1		1		1		1	2	1	1	2	1	
gi|83020812	XP_920195.1	PREDICTED: similar to Adaptor-related protein complex AP-1, sigma 3																	
gi|83029092	XP_357633.3	PREDICTED: similar to Ig kappa chain V-V region L7 precursor																1	
gi|83582782	NP_081562.2	angiotensin-converting enzyme 2 precursor																1	
gi|8392847	NP_058556.1	alpha-centractin																	
gi|84781771	NP_001034085.1	trypsin 10				1													
gi|84781781	NP_001028485.1	28 kDa heat- and acid-stable phosphoprotein																	
gi|84872231	NP_950181.2	ectonucleotide pyrophosphatase/phosphodiesterase family member 4 precursor																	
gi|8567336	NP_059502.1	calcium-activated chloride channel regulator 1 precursor		1	1	2	1		1	2	4		2		1				
gi|85701680	NP_001028349.1	keratin, type II cytoskeletal 2 oral																	
gi|85701714	NP_001028371.1	calcium activated chloride channel											1		4	4	4		1
gi|85719299	NP_001034274.1	carcinoembryonic antigen-related cell adhesion molecule 1 isoform 1		2							2		1		2	1			
gi|87239967	NP_031795.2	deleted in malignant brain tumors 1 protein		5	2	2	4	2	1	3	4	2	2	2	25	16	42	37	32
gi|8850219	NP_059066.1	haptoglobin precursor																	
gi|9055168	NP_061300.1	neutral ceramidase		9	10	2	3	1	5	9	7	3	9	3	6	2	4	1	1
gi|94363353	XP_484859.3	PREDICTED: similar to MYL6 protein																	
gi|94366612	XP_988506.1	PREDICTED: hypothetical protein														1			
gi|94377669	XP_993008.1	PREDICTED: similar to kappa-tnp V-J																	
gi|94377671	XP_993123.1	PREDICTED: similar to anti-A/U antibody																	

gi|94378251	XP_981474.1	PREDICTED: similar to histone H4																	
gi|94397546	XP_982057.1	PREDICTED: similar to ZFH-5		1					1		1			1	1	1	1	1	
gi|94400086	XP_984563.1	PREDICTED: similar to Ribosomal protein S27a																	
gi|94400195	XP_001003388.1	PREDICTED: similar to MSP23 isoform 2																	
gi|94407590	XP_898907.2	PREDICTED: similar to odorant binding protein Ib			3				1	1			3	2					
gi|9790069	NP_062667.1	spliceosome RNA helicase Bat1																	
gi|9790073	NP_062727.1	cadherin-17 precursor														3			1
gi|9790141	NP_062798.1	actin-related protein 2/3 complex subunit 3																	
gi|9910294	NP_064340.1	keratin, type II cytoskeletal 71				1				1				1	1	1	1		1

				Biological replicate 2-a															
				CONTROL								INFECTED							
gi number	Reference	Protein description	DAY ->	-1	1	3	6	10	14	21	28	-1	1	3	6	10	14	21	28
gi|10946884	NP_067456.1	trehalase precursor						1	1		1	1			1				
gi|110347469	NP_031402.3	alpha-2-macroglobulin precursor												1	5				
gi|110347479	NP_031458.2	intestinal-type alkaline phosphatase precursor		2		2		2	2			3		2	1	1	2		2
gi|110347564	NP_031778.2	ceruloplasmin isoform b													1				
gi|11037800	NP_067623.1	cell surface A33 antigen precursor												2	3	2	2	1	
gi|110556625	NP_997091.3	calcium-activated chloride channel regulator 4												1	3				
gi|110625761	NP_081406.1	AFG3-like protein 2																	
gi|111185905	NP_031990.2	ecotropic viral integration site 5 protein					1	1	1			1			1			1	
gi|111607447	NP_032430.2	integrin beta-2													3				
gi|112181182	NP_031773.2	cytochrome c oxidase subunit 5A, mitochondrial precursor													1				
gi|112181302	NP_001019787.2	putative GTP-binding protein Parf																	
gi|112293264	NP_031978.2	protein disulfide-isomerase A3 precursor					3								1		1		4
gi|112293275	NP_032558.2	epithelial cell adhesion molecule													1				
gi|113195678	NP_034814.2	lysosome-associated membrane glycoprotein 1																	
gi|113195684	NP_034799.2	keratin, type II cytoskeletal 6B				1	1							1	2				
gi|114052444	NP_056594.2	neutrophil elastase precursor																	
gi|114145487	NP_059503.2	ras-related GTP-binding protein C																	
gi|114145561	NP_112447.2	keratin, type II cytoskeletal 8													1				
gi|114158675	NP_079705.2	leukocyte elastase inhibitor A												9	9			5	
gi|114326446	NP_071855.2	myosin-9 isoform 1														1	1		
gi|114326546	NP_075907.2	phosphoglycerate mutase 1				35				13					1		1	2	1
gi|115648048	NP_035343.2	receptor-type tyrosine-protein phosphatase F precursor				1										2			
gi|116089273	NP_032138.3	rab GDP dissociation inhibitor beta			5										2	2	1		
gi|118601068	NP_032869.2	prolactin-inducible protein homolog precursor		1	1		2									1			
gi|119120879	NP_033231.2	solute carrier family 3, member 1					1							4	4	2		2	
gi|119392092	NP_444309.2	solute carrier family 15 member 1					1					1		1	2			1	
gi|119433657	NP_835585.3	histone H2A type 2-B																	
gi|121674797	NP_032943.2	palmitoyl-protein thioesterase 1 precursor																	
gi|124053457	NP_034950.2	multiple PDZ domain protein																	
gi|124244098	NP_031459.3	embryonic-type alkaline phosphatase precursor									1			3	2			1	
gi|124248572	NP_035822.2	prefoldin subunit 3		1													1		
gi|124249111	NP_001074245.1	coxsackie adenovirus receptor-like																	
gi|124339826	NP_034608.2	heat shock 70 kDa protein 1B				1										4			
gi|124430537	NP_001074443.1	sorcin isoform 1			1	2										1			
gi|124486895	NP_001074743.1	6-phosphogluconate dehydrogenase, decarboxylating																	
gi|124487227	NP_001074876.1	phospholipase B1, membrane-associated isoform 1		1	1								3	1	1	1		1	1
gi|124487275	NP_001074606.1	sucrase-isomaltase, intestinal		10	1	5	5	11	5	1	2	9		9	7	6	5	10	3
gi|124487297	NP_001074547.1	lactase-phlorizin hydrolase preproprotein		2				1	1			1		2	1				
gi|124487323	NP_001074551.1	intestinal alkaline phosphatase		12	4		3	9	7		8	7	3	17	20	5	6	17	4
gi|124487419	NP_034798.2	keratin, type II cytoskeletal 2 epidermal																	
gi|124517663	NP_034860.2	annexin A1			2			3							1		1		
gi|125628640	NP_808278.2	dual oxidase 2													2				
gi|126032329	NP_034236.2	elongation factor 1-alpha 1						3					1	4		1	1		

gi|126116585	NP_032499.2	keratin, type II cytoskeletal 1													1				
gi|126518317	NP_033908.2	complement C3			2	1							2	1	1	3			2
gi|12746442	NP_075547.1	selenocysteine-specific elongation factor																	
gi|12963555	NP_075664.1	omega-amidase NIT2								2									
gi|12963733	NP_076046.1	choline transporter-like protein 4												1					
gi|12963737	NP_076054.1	exportin-2														2			
gi|132626295	NP_032427.2	integrin alpha-M isoform 2 precursor													1				
gi|13374559	NP_075671.1	chymotrypsin-like																1	
gi|133778951	NP_032437.3	CUB and zona pellucida-like domain-containing protein 1		2		1	2	2	1		1	2		1	1	1	2	1	
gi|133778955	NP_034510.3	H-2 class I histocompatibility antigen, L-D alpha chain precursor													1				
gi|133778994	NP_573476.2	X-prolyl aminopeptidase (aminopeptidase P) 2, membrane-bound isoform 1		3	2	1		1	2				1	4	5	2	4	1	2
gi|13384886	NP_079745.1	colipase precursor													2				
gi|13385840	NP_080612.1	hypothetical protein LOC67719 precursor		76	88	66	84	69	56	63	54	48	35	123	95	82	73	118	111
gi|13385916	NP_080695.1	chymotrypsin-like elastase family, member 3B		1	1	3	3	3	1	1	2	2	2	25	20	6	5	9	1
gi|134031994	NP_032970.2	proteasome subunit alpha type-2														2			
gi|134288869	NP_033893.2	ADP-ribosyl cyclase 2																	
gi|134288917	NP_084514.2	cytoplasmic dynein 1 heavy chain 1					1								2	1			
gi|14192922	NP_033738.1	actin, alpha cardiac muscle 1				1	1				1			4	1		30		1
gi|145301549	NP_001077424.1	hemoglobin alpha, adult chain 2				1						2	14	6	3	1			
gi|145553963	NP_075022.2	betaine--homocysteine S-methyltransferase 2																	
gi|147901863	NP_032612.2	meprin A subunit beta precursor		1		1	2	3	2		1	1		5	5	6	5	5	3
gi|148229673	NP_001079003.1	hypothetical protein LOC546335																	
gi|148277039	NP_783327.2	alpha-2-macroglobulin-P precursor									1	1							
gi|148277591	NP_058087.2	syntenin-1 isoform 2																	
gi|148747546	NP_035588.2	serine protease inhibitor A3K precursor																	
gi|149234244	XP_001478035.1	PREDICTED: similar to Capping protein (actin filament) muscle Z-line, alpha 1					1	1										1	
gi|149240743	XP_001476708.1	PREDICTED: hypothetical protein																	
gi|149246448	XP_001472880.1	PREDICTED: similar to RIKEN cDNA 1810030J14 gene		1			1	1						2	10	5	2	4	1
gi|149248895	XP_001475565.1	PREDICTED: similar to Glyceraldehyde-3-phosphate dehydrogenase (GAPDH) isoform 1														1			
gi|149249272	XP_001476087.1	PREDICTED: similar to Glyceraldehyde-3-phosphate dehydrogenase (GAPDH)											1			2	1		
gi|149249466	XP_001477320.1	PREDICTED: hypothetical protein		2											2				
gi|149249766	XP_923442.2	PREDICTED: similar to fusion protein: ubiquitin (bases 43_513); ribosomal protein S27a (bases 217_532)																	
gi|149250381	XP_001475400.1	PREDICTED: similar to cadherin 22																	
gi|149250554	XP_001472375.1	PREDICTED: hypothetical protein																	
gi|149250606	XP_999253.2	PREDICTED: hypothetical protein																	
gi|149251724	XP_889997.3	PREDICTED: similar to secretory protein													2				
gi|149251776	XP_001476579.1	PREDICTED: similar to Nucleophosmin (NPM) (Nucleolar phosphoprotein B23) (Numatrin) (Nucleolar protein NO38)												1				1	
gi|149252769	XP_001477261.1	PREDICTED: hypothetical protein			1														
gi|149253386	XP_001471566.1	PREDICTED: hypothetical protein				7										1	1	1	
gi|149253488	XP_001473173.1	PREDICTED: similar to Tubulin, beta 4						1				2	1					5	5
gi|149253544	XP_001479464.1	PREDICTED: similar to cytochrome c																	
gi|149254235	XP_001473686.1	PREDICTED: hypothetical protein													8	4			
gi|149254414	XP_001479012.1	PREDICTED: similar to Muc3 protein															1		
gi|149254987	XP_001478026.1	PREDICTED: similar to trypsinogen 15		7	1	2	2	4	3	4	2	3	5	4	3	2		3	4
gi|149255095	XP_001476664.1	PREDICTED: similar to trypsinogen 15				1				1		1		1	1	1		2	
gi|149255293	XP_001480324.1	PREDICTED: similar to Murinoglobulin 1																	
gi|149255339	XP_996033.2	PREDICTED: similar to calmodulin													1				
gi|149255578	XP_001476107.1	PREDICTED: similar to immunoglobulin kappa chain						1	2						1				1
gi|149255583	XP_989825.2	PREDICTED: hypothetical protein																1	
gi|149255600	XP_001476873.1	PREDICTED: similar to light chain of the monoclonal antibody MST2													1				
gi|149255605	XP_001477220.1	PREDICTED: similar to [Human Ig rearranged gamma chain mRNA, V-J-C region and complete cds.]			1			1										1	
gi|149255611	XP_001477384.1	PREDICTED: similar to [Human Ig rearranged gamma chain mRNA, V-J-C region and complete cds.]																	

gi|149255615	XP_001477602.1	PREDICTED: similar to immunoglobulin kappa-chain																	
gi|149255637	XP_001476714.1	PREDICTED: similar to Chain L, Structural Basis Of Antigen Mimicry In A Clinically Relevant Melanoma Antigen System isoform 1						2					1		1	1	1	1	
gi|149255823	XP_001480334.1	PREDICTED: similar to Cofilin-1 (Cofilin, non-muscle isoform)				1										2			
gi|149256882	XP_001478116.1	PREDICTED: similar to peptidoglycan recognition protein																	
gi|149256898	XP_001471707.1	PREDICTED: hypothetical protein																	
gi|149257837	XP_001471659.1	PREDICTED: hypothetical protein											2						
gi|149258165	XP_001472728.1	PREDICTED: similar to beta-globin isoform 1										3			1		1		
gi|149258188	XP_001471843.1	PREDICTED: hypothetical protein																	
gi|149260099	XP_001474833.1	PREDICTED: similar to Annexin A2 (Annexin II) (Lipocortin II) (Calpactin I heavy chain) (Chromobindin-8) (p36) (Protein I) (Placental anticoagulant protein IV) (PAP-IV)														4			
gi|149261589	XP_486478.3	PREDICTED: similar to ferritin light chain 2																	
gi|149263303	XP_204772.4	PREDICTED: similar to endothelial monocyte-activating polypeptide		1		1					1								
gi|149263766	XP_001472141.1	PREDICTED: hypothetical protein		5	1	1	7	10	10	1	9	8	1	10	10	1	3	14	2
gi|149263798	XP_001474075.1	PREDICTED: similar to immunoglobulin epsilon					3	3	3		1	1			6		1	3	
gi|149264032	XP_001475950.1	PREDICTED: similar to Hist1h2bj protein																	
gi|149264124	XP_920080.3	PREDICTED: hypothetical protein			1	2										4	1		
gi|149264591	XP_001477449.1	PREDICTED: hypothetical protein																	
gi|149265958	XP_001474586.1	PREDICTED: similar to purine nucleoside phosphorylase		2															
gi|149267864	XP_001479188.1	PREDICTED: similar to apolipoprotein D													1				
gi|149269211	XP_908790.3	PREDICTED: similar to class Ib MHC antigen Qa-2 isoform 2																	
gi|149269220	XP_001473590.1	PREDICTED: similar to MHC H2-K 24 polypeptide isoform 1																	
gi|149269696	XP_001475963.1	PREDICTED: hypothetical protein																	
gi|149270372	XP_001474350.1	PREDICTED: hypothetical protein													1	1			
gi|149270446	XP_001478257.1	PREDICTED: similar to ribosomal protein S7														2	1		
gi|149270512	XP_001479065.1	PREDICTED: hypothetical protein																1	
gi|149271597	XP_001473196.1	PREDICTED: similar to spermine synthase																	2
gi|149272386	XP_001477701.1	PREDICTED: similar to thymosin beta-4																	2
gi|149274531	XP_001473875.1	PREDICTED: similar to heterogeneous nuclear ribonucleoprotein A2/B1		1												1	1		
gi|149274769	XP_001478965.1	PREDICTED: similar to VH coding region																	
gi|149275191	XP_001473825.1	PREDICTED: hypothetical protein																	
gi|15022803	NP_035583.1	serine (or cysteine) proteinase inhibitor, clade B, member 9c isoform 2				1										2			
gi|153945724	NP_033171.2	neuroplastin precursor																	
gi|153945747	NP_032672.2	murinoglobulin-2 precursor																	
gi|154090941	NP_997648.2	keratin, type I cytoskeletal 42																	
gi|154240724	NP_598766.2	ectonucleotide pyrophosphatase/phosphodiesterase family member 3		1			1		1		2	1			3	1			
gi|157951678	NP_033932.2	carbonic anhydrase 6 precursor									1								
gi|15826844	NP_035584.1	serine (or cysteine) proteinase inhibitor, clade B, member 6b				1										2			
gi|160333613	NP_958751.2	serine (or cysteine) proteinase inhibitor, clade B, member 3C																	
gi|160333652	NP_081496.2	protease, serine, 32																	
gi|160333869	NP_035258.2	pancreatic lipase-related protein 2												4	7	1		3	1
gi|160358829	NP_059067.2	hemopexin precursor									1	1							
gi|160707976	NP_036125.4	protein piccolo isoform 1																	
gi|161016799	NP_038499.2	annexin A4			1			1	1		1			1	23	101	74	1	8
gi|161016822	NP_001104301.1	aspartyl aminopeptidase isoform a									1	2		2	2	1	2	2	1
gi|161016833	NP_031902.2	dipeptidase 1 precursor			1		1						1	2	1			1	
gi|162138915	NP_034079.2	carnitine O-palmitoyltransferase 2, mitochondrial precursor																	
gi|163310765	NP_033784.2	serum albumin precursor													1		1		
gi|163914390	NP_033906.2	plasma protease C1 inhibitor precursor						2											
gi|16418335	NP_084072.1	leucine-rich alpha-2-glycoprotein																	
gi|164519050	NP_032720.2	neutrophilic granule protein													2				
gi|164565419	NP_001106839.1	carcinoembryonic antigen-related cell adhesion molecule 2 isoform a										1		1	1				
gi|165932341	NP_291090.2	chymotrypsin-like elastase family, member 1																	
gi|167004347	NP_001107794.1	cadherin-related family member 5 isoform 1													1				
gi|16716569	NP_444473.1	protease, serine, 1		2		1	2	1			1	1		1	1	1		2	1
gi|169790797	NP_001116075.1	Fc fragment of IgG binding protein		1	1	1	3	2	3	2	3	5	3	2	1	6	2		
gi|169790977	NP_001116119.1	major urinary protein 10																	

gi|170172530	NP_690052.2	immunoglobulin J chain precursor		1	1		1	1	1	1	1	1		1		1	1	1	
gi|170650599	NP_613057.2	elongation factor G, mitochondrial precursor																	
gi|17647499	NP_058652.1	hemoglobin subunit beta-2											1		1				
gi|18252782	NP_543120.1	antithrombin-III precursor						2											
gi|187283027	NP_001119790.1	hypothetical protein LOC242711						1						2	1			1	
gi|188219582	NP_036067.2	glucosamine-6-phosphate isomerase 1														1	1		
gi|189491671	NP_766081.1	protein diaphanous homolog 2		1						1									
gi|190684696	NP_033535.2	villin-1																	
gi|194328695	NP_035766.2	phospholipid scramblase 1																	
gi|19527028	NP_598569.1	vigilin						2								2			
gi|19527078	NP_598623.1	fibrinogen gamma chain precursor													3		1		
gi|19527082	NP_598628.1	epidermal growth factor receptor kinase substrate 8-like protein 3																	
gi|19527104	NP_598649.1	acid sphingomyelinase-like phosphodiesterase 3b precursor																	
gi|20330802	NP_598738.1	serotransferrin precursor													2				
gi|209862915	NP_001129527.1	CD82 antigen isoform 2													1				
gi|21105853	NP_631937.1	placenta-specific gene 8 protein													1				
gi|21312316	NP_082492.1	prostate stem cell antigen precursor		2	3	4			1		2	2	2	4	3	1	2	2	5
gi|213417962	NP_067369.2	Z-DNA-binding protein 1 isoform 1												1					
gi|21362335	NP_653120.1	carboxypeptidase A5 precursor																	
gi|21450325	NP_659172.1	flavin reductase				3													
gi|21489935	NP_058654.1	keratin, type I cytoskeletal 14																	
gi|21704144	NP_663544.1	S-adenosylmethionine synthase isoform type-2		1		2	2	2	2	1	2	2							
gi|21704206	NP_663578.1	carboxylesterase 2 precursor						1	1		1	1		1				1	
gi|22122475	NP_666122.1	tetraspanin 8		2				1			1	2		2	13	3	1	2	1
gi|22122641	NP_666231.1	niban-like protein 1			4			1					1			2			
gi|22164776	NP_666175.1	keratin, type II cytoskeletal 79																	
gi|22165388	NP_666261.1	hypothetical protein LOC230579																	
gi|22219434	NP_671734.1	nuclear receptor-binding protein						2								1			
gi|224809376	NP_067620.3	nicastrin precursor																	
gi|224922803	NP_034404.3	glycerol-3-phosphate dehydrogenase, mitochondrial precursor													3				
gi|225007644	NP_001139360.1	zinc finger protein ZFAT isoform 1													1			1	
gi|225637487	NP_032512.2	aminopeptidase N		17	1	3	5	9	3	2	5	12	1	16	21	11	11	16	7
gi|225690549	NP_796240.3	ribosome-releasing factor 2, mitochondrial isoform 1																	
gi|225735649	NP_001139592.1	sulfated glycoprotein 1 isoform A preproprotein													1		1		2
gi|226246531	NP_112467.2	phosphoglycerate kinase 2																	
gi|226823250	NP_034954.2	myeloperoxidase precursor													4				
gi|226874906	NP_033562.3	14-3-3 protein epsilon															2		5
gi|226874935	NP_034712.2	inter-alpha-trypsin inhibitor heavy chain H2						2					1						
gi|226958349	NP_033441.2	triosephosphate isomerase		1	11	1		3							2	2	1	1	6
gi|226958351	NP_038762.1	sodium/potassium-transporting ATPase subunit alpha-4													1				
gi|226958497	NP_035448.2	serum amyloid P-component precursor													28	8	5	3	
gi|227116347	NP_001153027.1	platelet glycoprotein 4																	
gi|227499103	NP_663392.2	aminopeptidase B isoform 1														2			
gi|227908835	NP_835180.2	probable carboxypeptidase PM20D1 precursor																	
gi|236468117	NP_001153622.1	amylase 2a4		1										2	1		1	4	
gi|237874196	NP_001153858.1	dynein, axonemal, heavy chain 7B																	
gi|23943876	NP_081194.1	zymogen granule membrane protein 16 precursor		23	6	7	10	9	8	8	9	9	5	11	14	4	6	4	4
gi|23956140	NP_079914.1	glycerophosphodiester phosphodiesterase domain-containing protein 1													2				
gi|23956196	NP_081487.1	annexin A13													1				
gi|23956222	NP_076224.1	actin-related protein 3														3	1		
gi|242332572	NP_057930.2	ras GTPase-activating-like protein IQGAP1											1			3	3		
gi|242397450	NP_035178.1	paired basic amino acid cleaving system 4																	
gi|24496768	NP_038949.2	serine protease 30 precursor												1	1				
gi|25141335	NP_082045.1	copine-3													1				
gi|251823804	NP_660108.2	chitinase-3-like protein 4 precursor																	
gi|251823824	NP_082166.3	sushi domain-containing protein 2 isoform 1																	
gi|253970410	NP_653127.2	hypothetical protein LOC78354																	
gi|253970442	NP_997125.2	Niemann-Pick C1-like protein 1 precursor																1	

gi|254281348	NP_034022.2	chitinase-3-like protein 3 precursor														2		2		
gi|254540166	NP_071705.3	78 kDa glucose-regulated protein precursor							1							2	7	4		2
gi|254692909	NP_001013044.2	transcription termination factor 2																		
gi|255069715	NP_077754.3	cytosol aminopeptidase								1										
gi|255522937	NP_079859.2	chymotrypsinogen B precursor		3	3	3	9	4	7	1	7	5	1	9	5	2	4	8	2
gi|255683322	NP_766413.2	probable G-protein coupled receptor 128 precursor																		
gi|256222930	NP_663549.2	coiled-coil domain-containing protein 136																		
gi|257467625	NP_001158127.1	Fc fragment of IgG binding protein-like																		
gi|258547152	NP_001158149.1	programmed cell death 6-interacting protein isoform 1															2			
gi|260304980	NP_001159443.1	predicted gene 5803											2							
gi|262073053	NP_081515.2	hypothetical protein LOC69864																		
gi|268836255	NP_573461.2	glycogen phosphorylase, liver form																		
gi|269308203	NP_082776.2	calreticulin-3 isoform 1			1												2			
gi|27151739	NP_032780.2	odorant binding protein Ia											1							
gi|27229028	NP_080265.2	pancreatic secretory granule membrane major glycoprotein GP2																	1	
gi|27370012	NP_766288.1	CAAX prenyl protease 1 homolog													1					
gi|27370038	NP_766299.1	GTP-binding protein GUF1 homolog																		
gi|27754136	NP_075675.2	acidic mammalian chitinase precursor		5			3	4	3		6	1	1	2	2	2	3	2	
gi|282165827	NP_001028536.2	protocadherin 24		5	2	1	7	4	4	2	9	8	3	10	1	3	4	8	4
gi|28316756	NP_783589.1	histone cluster 1, H2aa																		
gi|283483997	NP_001164474.1	maltase-glucoamylase		11		4	5	5	9	2	3	3	1	11	12	2	7	14	4
gi|294489324	NP_001170938.1	MHC class Ib T9																		
gi|294610643	NP_076055.2	mucin-2		1			3		1		1	2		2	1	1		1	
gi|294997230	NP_996988.2	histocompatibility 2, class II antigen A, beta 1 precursor													7		1	2	
gi|29789211	NP_082202.1	carboxypeptidase A4 precursor		2		1	2	1	1			1		1					
gi|29789343	NP_598677.1	eukaryotic translation initiation factor 3 subunit B			4														
gi|29789389	NP_766172.1	inositol-tetrakisphosphate 1-kinase														2	1		
gi|30410738	NP_848499.1	hypothetical protein LOC229688																	
gi|30425250	NP_780706.1	beta-actin-like protein 2		1	1													6	1
gi|30519911	NP_848713.1	transgelin-2													5	1			
gi|30725780	NP_849251.1	protein MAL2																	
gi|31542362	NP_031672.2	ADP-ribosyl cyclase 1																	
gi|31543113	NP_032905.2	plastin-2														1		1	
gi|31543255	NP_032630.2	neprilysin		2		1		1	1					1	6	2		1	1
gi|31543942	NP_033528.3	vinculin				2													
gi|31543974	NP_061223.2	14-3-3 protein beta/alpha					2											2	
gi|31543976	NP_061359.2	14-3-3 protein gamma																	5
gi|31560030	NP_077745.2	tropomyosin alpha-1 chain isoform 3			1												1		3
gi|31560433	NP_059500.2	sorting nexin-3				1										2			
gi|31560677	NP_032548.2	lactotransferrin precursor													1				
gi|31560731	NP_031534.2	V-type proton ATPase catalytic subunit A														2			
gi|31980868	NP_034769.4	kallikrein-1 precursor						1			2	1			1		1		
gi|31981542	NP_035308.2	myeloblastin precursor													1				
gi|31981562	NP_035229.2	pyruvate kinase isozymes M1/M2												1			1		3
gi|31981570	NP_035212.2	polymeric immunoglobulin receptor precursor		2			1	1	1	1	4	1		3	3			4	
gi|31981716	NP_034508.2	histocompatibility 2, class II antigen A, alpha precursor													1	1			
gi|31981890	NP_033865.2	beta-2-microglobulin precursor																	
gi|31982030	NP_598557.3	rho GDP-dissociation inhibitor 1															5		
gi|31982171	NP_032671.2	murinoglobulin-1 precursor												1	2			2	
gi|31982186	NP_032643.2	malate dehydrogenase, mitochondrial precursor															1		
gi|31982199	NP_032611.2	meprin A subunit alpha												1				1	
gi|33563236	NP_031512.1	rho GDP-dissociation inhibitor 2				3										1			
gi|33563240	NP_033736.1	actin, alpha skeletal muscle												1					
gi|33563252	NP_034326.1	fibrinogen, alpha polypeptide isoform 2													3	1	2	1	
gi|33859474	NP_031784.1	complement receptor type 2																	
gi|33859640	NP_035658.1	transaldolase																2	
gi|33859686	NP_079976.1	phosphoglucomutase-2															1	1	
gi|33859809	NP_862897.1	fibrinogen beta chain precursor			6		1								3				

gi|34328436	NP_081138.2	CD177 antigen precursor													1				
gi|34538600	NP_904330.1	cytochrome c oxidase subunit I																	
gi|34538601	NP_904331.1	cytochrome c oxidase subunit II																	
gi|34915988	NP_033898.1	basigin isoform 1							1								1		2
gi|36031132	NP_031530.2	sarcoplasmic/endoplasmic reticulum calcium ATPase 1												2	1			1	
gi|37674236	NP_081201.2	pancreatic triacylglycerol lipase		15	10	5	6	8	6	7	13	10	8	12	16	9	10	14	20
gi|38076713	XP_357312.1	PREDICTED: similar to ferritin light chain 2																	
gi|40556608	NP_032328.2	heat shock protein HSP 90-beta			1	6	1			1							1		5
gi|42415475	NP_035162.1	protein disulfide-isomerase precursor													1			2	
gi|45504394	NP_034708.1	integrin beta-1 precursor													1				
gi|46559389	NP_997507.1	angiotensin-converting enzyme isoform 1																	
gi|50345978	NP_001002012.1	heat shock-related 70 kDa protein 2		1			1					1			2			1	
gi|51010909	NP_001003405.1	trypsin 5		9	5	7	11	21	10	6	7	15	6	12	3	2	7	14	7
gi|51491845	NP_001003908.1	clathrin heavy chain 1							1						2				
gi|51765047	XP_486246.1	PREDICTED: similar to Tubulin, alpha 3c isoform 1												1		1			
gi|54312076	NP_079626.2	carboxypeptidase A1 precursor		3		2	46	98	59	40	18	13	14	30	2	6	8	8	4
gi|56090544	NP_001007553.1	intelectin-1b precursor												1	1				
gi|56550071	NP_083982.1	pancreatic carboxypeptidase B1		2		1	9	15	9	1	6	1	1	8	8	1	2	10	
gi|57222282	NP_001009546.1	N-acetylated-alpha-linked acidic dipeptidase-like protein		8	1	2	4	7	6	3	4	7	3	11	6	1	7	13	5
gi|61097906	NP_598917.1	alpha-actinin-1													1	2			
gi|61743961	NP_033773.1	AHNAK nucleoprotein isoform 1					2												
gi|6671509	NP_031419.1	actin, cytoplasmic 1								236		1					8		
gi|6671539	NP_031464.1	fructose-bisphosphate aldolase A isoform 2														1	1		
gi|6671664	NP_031623.1	calnexin precursor																	
gi|6671672	NP_031630.1	F-actin-capping protein subunit alpha-2						1										1	
gi|6671678	NP_031633.1	carbonic anhydrase 4 precursor													2				
gi|6671746	NP_031714.1	cofilin-2															3	2	
gi|6677703	NP_033068.1	lithostathine-1 precursor													2				
gi|6677705	NP_033069.1	lithostathine-2 precursor													3	3	2		
gi|6677837	NP_033140.1	protein S100-A9													4		2	3	
gi|6678079	NP_033269.1	alpha-1-antitrypsin 1-1 precursor																	
gi|6678085	NP_033272.1	alpha-1-antitrypsin 1-4 precursor																	
gi|6678097	NP_033280.1	serpin B6 isoform b																	
gi|6678329	NP_033399.1	protein-glutamine gamma-glutamyltransferase 2														2	1	1	
gi|6678439	NP_033456.1	anionic trypsin-2 precursor		45	59	29	73	94	65	95	90	67	85	64	23	15	9	34	58
gi|6678581	NP_033541.1	wiskott-Aldrich syndrome protein homolog												1				1	
gi|6679293	NP_033428.1	peptidoglycan recognition protein 1 precursor																	
gi|6679383	NP_032904.1	alpha-2-antiplasmin precursor																3	
gi|6679439	NP_032933.1	peptidyl-prolyl cis-trans isomerase A																	2
gi|6679509	NP_032979.1	Psp																	
gi|6679653	NP_031960.1	glutamyl aminopeptidase		8		1	8	4	9		6	6		10	26	6	8	9	4
gi|6679939	NP_032111.1	glyceraldehyde-3-phosphate dehydrogenase, testis-specific																	
gi|6679995	NP_032142.1	gamma-glutamyltranspeptidase 1		1				1							1	2	1		
gi|6680231	NP_032279.1	high mobility group protein B3														1			2
gi|6680441	NP_032401.1	gastrotropin																	
gi|6680586	NP_032484.1	serine protease inhibitor A3C precursor																	
gi|6680636	NP_031424.1	adenosine deaminase																	
gi|6680710	NP_031498.1	aquaporin-1												1	1				
gi|6680836	NP_031617.1	calreticulin precursor						2							6	1			
gi|6681297	NP_031945.1	chymotrypsin-like elastase family member 2A precursor												1			1	1	
gi|67010039	NP_001019869.1	carboxypeptidase A2 precursor		1			7	5	3	3	6	2		3		1	1	5	
gi|6753060	NP_033803.1	annexin A5			13	6	2	1								4	20	2	1
gi|6753138	NP_033851.1	sodium/potassium-transporting ATPase subunit beta-1					1		1					3	8	1	1	1	
gi|6753244	NP_033920.1	calmodulin														7	11		
gi|6753322	NP_033967.1	T-complex protein 1 subunit delta														2			
gi|6753428	NP_034027.1	creatine kinase U-type, mitochondrial precursor						1						1	1				
gi|6753674	NP_034204.1	dipeptidyl peptidase 4 isoform 1		1		2	1				1			1	1				2
gi|6753798	NP_034298.1	prothrombin								2									

gi|6753912	NP_034369.1	ferritin heavy chain							2										
gi|6754254	NP_034610.1	heat shock protein HSP 90-alpha							8				56		1				
gi|6754388	NP_034714.1	intelectin-1a precursor																	
gi|6754480	NP_034792.1	keratin, type I cytoskeletal 13													1				
gi|6754534	NP_034837.1	galectin-6													1				
gi|6754576	NP_034869.1	mucin-13 precursor												2		1		1	
gi|6754658	NP_034907.1	Golli-Mbp isoform 1																	
gi|6754976	NP_035164.1	peroxiredoxin-1														2			
gi|6754980	NP_035166.1	regenerating islet-derived protein 3-beta precursor												14	10	3	5	13	4
gi|6755090	NP_035237.1	phospholipase A2 precursor																	
gi|6755100	NP_035249.1	proliferation-associated protein 2G4														2			
gi|6755112	NP_035255.1	phospholipid transfer protein precursor																	
gi|6755144	NP_035280.1	galectin-3-binding protein precursor																	
gi|6755210	NP_036005.1	26S proteasome non-ATPase regulatory subunit 13														2			
gi|6755256	NP_035354.1	glycogen phosphorylase, muscle form				1								1					
gi|6755308	NP_035389.1	regenerating islet-derived protein 3-alpha precursor													2				
gi|6755310	NP_035390.1	regenerating islet-derived protein 3-gamma precursor					1							3	5	3	2	6	2
gi|6755364	NP_035424.1	activated RNA polymerase II transcriptional coactivator p15																	
gi|6755372	NP_036182.1	40S ribosomal protein S3														2			
gi|6755863	NP_035761.1	endoplasmin			2	4			1						1		1		1
gi|6755963	NP_035824.1	voltage-dependent anion-selective channel protein 1																	
gi|6755965	NP_035825.1	voltage-dependent anion-selective channel protein 2												2	3				
gi|6755967	NP_035826.1	voltage-dependent anion-selective channel protein 3													2				
gi|6756037	NP_035868.1	14-3-3 protein eta																	2
gi|68226731	NP_032968.2	puromycin-sensitive aminopeptidase													1	1			
gi|6996913	NP_031611.1	annexin A2				1										1			
gi|70778976	NP_032854.2	phosphoglycerate kinase 1			3	10	2	1							25	20	33	2	1
gi|71043961	NP_075822.3	trypsinogen 7		1				1						1	3	1	1	1	
gi|7110683	NP_035174.1	phosphoenolpyruvate carboxykinase, cytosolic [GTP]																	
gi|7304963	NP_038913.1	chloride intracellular channel protein 4													1	8	1		
gi|7305189	NP_038593.1	integrin alpha-3																	
gi|7305453	NP_038678.1	protein S100-A8																	
gi|7305599	NP_038725.1	transthyretin																	
gi|75677435	NP_031463.2	fatty aldehyde dehydrogenase													1				
gi|76443665	NP_001029047.1	chymotrypsin-C precursor		185	190	83	157	143	134	123	119	133	108	135	107	83	61	98	119
gi|76881807	NP_033270.3	alpha-1-antitrypsin 1-2 precursor									1			1	1				
gi|7710018	NP_038543.1	erythrocyte band 7 integral membrane protein													2	2			
gi|7949064	NP_032482.1	kallikrein 1-related peptidase b5 precursor		1			1		1							1	1		
gi|82796081	XP_928726.1	PREDICTED: hypothetical protein																	
gi|82799602	XP_913793.1	PREDICTED: similar to Glyceraldehyde-3-phosphate dehydrogenase		1	2						2				4	1			
gi|82802624	XP_920189.1	PREDICTED: similar to hCG2001479		1			4	2	3		2	3		3	2			3	
gi|82882188	XP_922713.1	PREDICTED: similar to intelectin												2	1		1		
gi|82895363	XP_919532.1	PREDICTED: hypothetical protein												1					1
gi|82935925	XP_913239.1	PREDICTED: similar to myosin, heavy polypeptide 3, skeletal muscle, embryonic												3	2			1	
gi|82998325	XP_904967.1	PREDICTED: hypothetical protein isoform 7														2	1		
gi|83008262	XP_913818.1	PREDICTED: similar to Ab 414.2 heavy chain variable and joining region									1							1	
gi|83014618	XP_909851.1	PREDICTED: similar to Igh protein		1			2	2			1	1		1	1			1	
gi|83020812	XP_920195.1	PREDICTED: similar to Adaptor-related protein complex AP-1, sigma 3		1											1				
gi|83029092	XP_357633.3	PREDICTED: similar to Ig kappa chain V-V region L7 precursor																	
gi|83582782	NP_081562.2	angiotensin-converting enzyme 2 precursor		1			1								2	1	1	1	1
gi|8392847	NP_058556.1	alpha-centractin		2															
gi|84781771	NP_001034085.1	trypsin 10		1			1		2			1	1	1			2		1
gi|84781781	NP_001028485.1	28 kDa heat- and acid-stable phosphoprotein																	2
gi|84872231	NP_950181.2	ectonucleotide pyrophosphatase/phosphodiesterase family member 4 precursor																	
gi|8567336	NP_059502.1	calcium-activated chloride channel regulator 1 precursor						1	1		2			2	1			1	
gi|85701680	NP_001028349.1	keratin, type II cytoskeletal 2 oral				1													

gi|85701714	NP_001028371.1	calcium activated chloride channel		2			1	2			1			3	10	1	2	2	
gi|85719299	NP_001034274.1	carcinoembryonic antigen-related cell adhesion molecule 1 isoform 1													1				
gi|87239967	NP_031795.2	deleted in malignant brain tumors 1 protein		4	2	2	1	3	4		2	3		6	9	4	1	4	2
gi|8850219	NP_059066.1	haptoglobin precursor													1				
gi|9055168	NP_061300.1	neutral ceramidase		6	1	1	2				4	1	2	6	3	1	2	4	1
gi|94363353	XP_484859.3	PREDICTED: similar to MYL6 protein					2	1											
gi|94366612	XP_988506.1	PREDICTED: hypothetical protein																	
gi|94377669	XP_993008.1	PREDICTED: similar to kappa-tnp V-J																	
gi|94377671	XP_993123.1	PREDICTED: similar to anti-A/U antibody																	
gi|94378251	XP_981474.1	PREDICTED: similar to histone H4												1					1
gi|94397546	XP_982057.1	PREDICTED: similar to ZFH-5																	
gi|94400086	XP_984563.1	PREDICTED: similar to Ribosomal protein S27a													1				
gi|94400195	XP_001003388.1	PREDICTED: similar to MSP23 isoform 2														3			
gi|94407590	XP_898907.2	PREDICTED: similar to odorant binding protein Ib											2			1			
gi|9790069	NP_062667.1	spliceosome RNA helicase Bat1				1										1			
gi|9790073	NP_062727.1	cadherin-17 precursor						1	1					4	3	1		1	
gi|9790141	NP_062798.1	actin-related protein 2/3 complex subunit 3														3	1		
gi|9910294	NP_064340.1	keratin, type II cytoskeletal 71													1				

				Biological replicate 2-b															
				CONTROL								INFECTED							
gi number	Reference	Protein description	DAY ->	-1	1	3	6	10	14	21	28	-1	1	3	6	10	14	21	28
gi|10946884	NP_067456.1	trehalase precursor		1	4	1		2	2		1		2	2	7	4	2	1	4
gi|110347469	NP_031402.3	alpha-2-macroglobulin precursor												4	31	8	3	2	
gi|110347479	NP_031458.2	intestinal-type alkaline phosphatase precursor		3	16	4	3	7	8	2	6	6	16	11	6	6	6	7	17
gi|110347564	NP_031778.2	ceruloplasmin isoform b													2				
gi|11037800	NP_067623.1	cell surface A33 antigen precursor		1	3		1	1	1	1	1	1	5	8	24	9	8	4	6
gi|110556625	NP_997091.3	calcium-activated chloride channel regulator 4		2	15	1							16	13	20	7	3	3	10
gi|110625761	NP_081406.1	AFG3-like protein 2									1		1						
gi|111185905	NP_031990.2	ecotropic viral integration site 5 protein		2		2	2	2	2		1			3	6		2	3	2
gi|111607447	NP_032430.2	integrin beta-2													27	1	2		
gi|112181182	NP_031773.2	cytochrome c oxidase subunit 5A, mitochondrial precursor													5				
gi|112181302	NP_001019787.2	putative GTP-binding protein Parf																	
gi|112293264	NP_031978.2	protein disulfide-isomerase A3 precursor													4				
gi|112293275	NP_032558.2	epithelial cell adhesion molecule													4				
gi|113195678	NP_034814.2	lysosome-associated membrane glycoprotein 1													1	1	1		
gi|113195684	NP_034799.2	keratin, type II cytoskeletal 6B		1	2	2	1	1		1		1	1	1	3	1		1	
gi|114052444	NP_056594.2	neutrophil elastase precursor													1				
gi|114145487	NP_059503.2	ras-related GTP-binding protein C									2								
gi|114145561	NP_112447.2	keratin, type II cytoskeletal 8													1				
gi|114158675	NP_079705.2	leukocyte elastase inhibitor A		5	1	2	3				1	1	1	11	63	13	3	11	10
gi|114326446	NP_071855.2	myosin-9 isoform 1																	
gi|114326546	NP_075907.2	phosphoglycerate mutase 1																	
gi|115648048	NP_035343.2	receptor-type tyrosine-protein phosphatase F precursor																	
gi|116089273	NP_032138.3	rab GDP dissociation inhibitor beta																	
gi|118601068	NP_032869.2	prolactin-inducible protein homolog precursor		5	10		2	1	1	3	1	1	12	3		3	3	4	3
gi|119120879	NP_033231.2	solute carrier family 3, member 1		1	6	3	1						11	7	34	10	7	5	5
gi|119392092	NP_444309.2	solute carrier family 15 member 1		2	2	2	2	3	1	1	2		1	4	9	9	6	5	5
gi|119433657	NP_835585.3	histone H2A type 2-B													3				
gi|121674797	NP_032943.2	palmitoyl-protein thioesterase 1 precursor													2				
gi|124053457	NP_034950.2	multiple PDZ domain protein																	
gi|124244098	NP_031459.3	embryonic-type alkaline phosphatase precursor		1	1	2	1	1	1	1	1	5	11	5	5	3		1	10
gi|124248572	NP_035822.2	prefoldin subunit 3																	
gi|124249111	NP_001074245.1	coxsackie adenovirus receptor-like																3	
gi|124339826	NP_034608.2	heat shock 70 kDa protein 1B																	
gi|124430537	NP_001074443.1	sorcin isoform 1																	

gi|124486895	NP_001074743.1	6-phosphogluconate dehydrogenase, decarboxylating															1		1
gi|124487227	NP_001074876.1	phospholipase B1, membrane-associated isoform 1		3	9		1	2	2		2	1	23	5	14	5	3	4	4
gi|124487275	NP_001074606.1	sucrase-isomaltase, intestinal		35	31	50	21	34	28	5	21	20	51	45	62	52	63	43	57
gi|124487297	NP_001074547.1	lactase-phlorizin hydrolase preproprotein		6	5	6		3	1	1			6	9	11	3	1		7
gi|124487323	NP_001074551.1	intestinal alkaline phosphatase		27	68	18	20	35	33	9	28	19	142	63	90	48	37	53	89
gi|124487419	NP_034798.2	keratin, type II cytoskeletal 2 epidermal				1													
gi|124517663	NP_034860.2	annexin A1													3				
gi|125628640	NP_808278.2	dual oxidase 2													6				
gi|126032329	NP_034236.2	elongation factor 1-alpha 1																	
gi|126116585	NP_032499.2	keratin, type II cytoskeletal 1			2										2				
gi|126518317	NP_033908.2	complement C3													8	5	2		
gi|12746442	NP_075547.1	selenocysteine-specific elongation factor		1		1								4			1	2	
gi|12963555	NP_075664.1	omega-amidase NIT2																	
gi|12963733	NP_076046.1	choline transporter-like protein 4													3	1	1		1
gi|12963737	NP_076054.1	exportin-2																	
gi|132626295	NP_032427.2	integrin alpha-M isoform 2 precursor													2	4	2		
gi|13374559	NP_075671.1	chymotrypsin-like								1	1								
gi|133778951	NP_032437.3	CUB and zona pellucida-like domain-containing protein 1		8	2	1	4	6	2	2	6	5	4	5	4	5	3	2	5
gi|133778955	NP_034510.3	H-2 class I histocompatibility antigen, L-D alpha chain precursor													4				
gi|133778994	NP_573476.2	X-prolyl aminopeptidase (aminopeptidase P) 2, membrane-bound isoform 1		8	33	8	1	1	1	2	4	3	42	23	22	10	11	6	18
gi|13384886	NP_079745.1	colipase precursor													2				1
gi|13385840	NP_080612.1	hypothetical protein LOC67719 precursor		255	25	720	313	446	402	167	238	134	25	358	524	252	201	577	199
gi|13385916	NP_080695.1	chymotrypsin-like elastase family, member 3B		8	22	15	17	43	14	18	16	6	81	126	136	60	111	37	11
gi|134031994	NP_032970.2	proteasome subunit alpha type-2																	
gi|134288869	NP_033893.2	ADP-ribosyl cyclase 2			1								3		1				
gi|134288917	NP_084514.2	cytoplasmic dynein 1 heavy chain 1																	
gi|14192922	NP_033738.1	actin, alpha cardiac muscle 1		2		3	3	1		1	3	1		9	9	3	2	4	4
gi|145301549	NP_001077424.1	hemoglobin alpha, adult chain 2													2				
gi|145553963	NP_075022.2	betaine--homocysteine S-methyltransferase 2			1													1	
gi|147901863	NP_032612.2	meprin A subunit beta precursor		2	2	3	1	1	3	1	3	2	4	12	17	26	14	17	13
gi|148229673	NP_001079003.1	hypothetical protein LOC546335			3								12	1	1				
gi|148277039	NP_783327.2	alpha-2-macroglobulin-P precursor													2				
gi|148277591	NP_058087.2	syntenin-1 isoform 2													2				
gi|148747546	NP_035588.2	serine protease inhibitor A3K precursor			2								1						
gi|149234244	XP_001478035.1	PREDICTED: similar to Capping protein (actin filament) muscle Z-line, alpha 1																	
gi|149240743	XP_001476708.1	PREDICTED: hypothetical protein			4								1						
gi|149246448	XP_001472880.1	PREDICTED: similar to RIKEN cDNA 1810030J14 gene		5	5	3	5	2	6	2	3	5	16	14	91	19	9	8	8
gi|149248895	XP_001475565.1	PREDICTED: similar to Glyceraldehyde-3-phosphate dehydrogenase (GAPDH) isoform 1												3	1	3	1	3	
gi|149249272	XP_001476087.1	PREDICTED: similar to Glyceraldehyde-3-phosphate dehydrogenase (GAPDH)																	
gi|149249466	XP_001477320.1	PREDICTED: hypothetical protein																	
gi|149249766	XP_923442.2	PREDICTED: similar to fusion protein: ubiquitin (bases 43_513); ribosomal protein S27a (bases 217_532)													1				
gi|149250381	XP_001475400.1	PREDICTED: similar to cadherin 22									1			1		1			
gi|149250554	XP_001472375.1	PREDICTED: hypothetical protein												1	7	3	2		1
gi|149250606	XP_999253.2	PREDICTED: hypothetical protein										1							
gi|149251724	XP_889997.3	PREDICTED: similar to secretory protein													6	3	8		
gi|149251776	XP_001476579.1	PREDICTED: similar to Nucleophosmin (NPM) (Nucleolar phosphoprotein B23) (Numatrin) (Nucleolar protein NO38)													3			2	
gi|149252769	XP_001477261.1	PREDICTED: hypothetical protein			1								2						
gi|149253386	XP_001471566.1	PREDICTED: hypothetical protein																	
gi|149253488	XP_001473173.1	PREDICTED: similar to Tubulin, beta 4																	
gi|149253544	XP_001479464.1	PREDICTED: similar to cytochrome c																	
gi|149254235	XP_001473686.1	PREDICTED: hypothetical protein													61	8	8		
gi|149254414	XP_001479012.1	PREDICTED: similar to Muc3 protein											1	1	4	2	1		2
gi|149254987	XP_001478026.1	PREDICTED: similar to trypsinogen 15		2	3	2		2		2	3	2	1	2	3	2	1	2	3
gi|149255095	XP_001476664.1	PREDICTED: similar to trypsinogen 15		1		2	2	2	2	2	1	1		2	2	2	2		2

gi|149255293	XP_001480324.1	PREDICTED: similar to Murinoglobulin 1														1			
gi|149255339	XP_996033.2	PREDICTED: similar to calmodulin		1														1	
gi|149255578	XP_001476107.1	PREDICTED: similar to immunoglobulin kappa chain			2	1	1	4	3		1		2	2	1	2	1	3	1
gi|149255583	XP_989825.2	PREDICTED: hypothetical protein														1			
gi|149255600	XP_001476873.1	PREDICTED: similar to light chain of the monoclonal antibody MST2					1	1	1		1		1		2	1	1	2	1
gi|149255605	XP_001477220.1	PREDICTED: similar to [Human Ig rearranged gamma chain mRNA, V-J-C region and complete cds.]		1	5	1	2	1	1	1	1				2	1	1	1	2
gi|149255611	XP_001477384.1	PREDICTED: similar to [Human Ig rearranged gamma chain mRNA, V-J-C region and complete cds.]						1											
gi|149255615	XP_001477602.1	PREDICTED: similar to immunoglobulin kappa-chain			1				1				3		1	3	1		1
gi|149255637	XP_001476714.1	PREDICTED: similar to Chain L, Structural Basis Of Antigen Mimicry In A Clinically Relevant Melanoma Antigen System isoform 1		1	3		2	2		5	3		11	4	7	10	6	6	12
gi|149255823	XP_001480334.1	PREDICTED: similar to Cofilin-1 (Cofilin, non-muscle isoform)																	
gi|149256882	XP_001478116.1	PREDICTED: similar to peptidoglycan recognition protein													9			1	
gi|149256898	XP_001471707.1	PREDICTED: hypothetical protein		1									1	3	14	3		1	
gi|149257837	XP_001471659.1	PREDICTED: hypothetical protein																	
gi|149258165	XP_001472728.1	PREDICTED: similar to beta-globin isoform 1													2				
gi|149258188	XP_001471843.1	PREDICTED: hypothetical protein					2												
gi|149260099	XP_001474833.1	PREDICTED: similar to Annexin A2 (Annexin II) (Lipocortin II) (Calpactin I heavy chain) (Chromobindin-8) (p36) (Protein I) (Placental anticoagulant protein IV) (PAP-IV)			2									1			1		1
gi|149261589	XP_486478.3	PREDICTED: similar to ferritin light chain 2		2	1	1	5	3	6		4		2		2				
gi|149263303	XP_204772.4	PREDICTED: similar to endothelial monocyte-activating polypeptide																	
gi|149263766	XP_001472141.1	PREDICTED: hypothetical protein		38	40	50	109	195	189	12	101	17	76	40	37	47	10	37	32
gi|149263798	XP_001474075.1	PREDICTED: similar to immunoglobulin epsilon		2	15	3	6	10	9	1	9	4	19	5	22	7	6	2	9
gi|149264032	XP_001475950.1	PREDICTED: similar to Hist1h2bj protein			1	1									1				
gi|149264124	XP_920080.3	PREDICTED: hypothetical protein																	
gi|149264591	XP_001477449.1	PREDICTED: hypothetical protein																	
gi|149265958	XP_001474586.1	PREDICTED: similar to purine nucleoside phosphorylase																	
gi|149267864	XP_001479188.1	PREDICTED: similar to apolipoprotein D													2			1	
gi|149269211	XP_908790.3	PREDICTED: similar to class Ib MHC antigen Qa-2 isoform 2													1				
gi|149269220	XP_001473590.1	PREDICTED: similar to MHC H2-K 24 polypeptide isoform 1													3	2	1		
gi|149269696	XP_001475963.1	PREDICTED: hypothetical protein				1	1		1					1	1				
gi|149270372	XP_001474350.1	PREDICTED: hypothetical protein																1	
gi|149270446	XP_001478257.1	PREDICTED: similar to ribosomal protein S7																	
gi|149270512	XP_001479065.1	PREDICTED: hypothetical protein			1							1							
gi|149271597	XP_001473196.1	PREDICTED: similar to spermine synthase																	
gi|149272386	XP_001477701.1	PREDICTED: similar to thymosin beta-4																	
gi|149274531	XP_001473875.1	PREDICTED: similar to heterogeneous nuclear ribonucleoprotein A2/B1																	
gi|149274769	XP_001478965.1	PREDICTED: similar to VH coding region		1			1												
gi|149275191	XP_001473825.1	PREDICTED: hypothetical protein													4				
gi|15022803	NP_035583.1	serine (or cysteine) proteinase inhibitor, clade B, member 9c isoform 2		2	3								1	3	13	2		3	5
gi|153945724	NP_033171.2	neuroplastin precursor													2				
gi|153945747	NP_032672.2	murinoglobulin-2 precursor													2				
gi|154090941	NP_997648.2	keratin, type I cytoskeletal 42													2				
gi|154240724	NP_598766.2	ectonucleotide pyrophosphatase/phosphodiesterase family member 3		6	9	1	2	4	3		4	4	16	4	38	21	9	9	14
gi|157951678	NP_033932.2	carbonic anhydrase 6 precursor		1	2		2		2		1		4					1	
gi|15826844	NP_035584.1	serine (or cysteine) proteinase inhibitor, clade B, member 6b												10	16	2		2	4
gi|160333613	NP_958751.2	serine (or cysteine) proteinase inhibitor, clade B, member 3C			3								6		1				
gi|160333652	NP_081496.2	protease, serine, 32			1								2	1	1	1	1		1
gi|160333869	NP_035258.2	pancreatic lipase-related protein 2		4	24	5							8	2	28	14	4	6	1
gi|160358829	NP_059067.2	hemopexin precursor																	
gi|160707976	NP_036125.4	protein piccolo isoform 1													4				
gi|161016799	NP_038499.2	annexin A4		3	6	3	1				2	2	5	5	33	13	9	1	11
gi|161016822	NP_001104301.1	aspartyl aminopeptidase isoform a		2	1		6	2	2	1	1	2	4	3	16	4	2	4	5
gi|161016833	NP_031902.2	dipeptidase 1 precursor		4	22	3	1	3	6		5	2	26	9	7	4	3	6	8
gi|162138915	NP_034079.2	carnitine O-palmitoyltransferase 2, mitochondrial precursor													2				
gi|163310765	NP_033784.2	serum albumin precursor			2								1		25	1	1		

gi|163914390	NP_033906.2	plasma protease C1 inhibitor precursor																	
gi|16418335	NP_084072.1	leucine-rich alpha-2-glycoprotein													2				
gi|164519050	NP_032720.2	neutrophilic granule protein													8	1	4		
gi|164565419	NP_001106839.1	carcinoembryonic antigen-related cell adhesion molecule 2 isoform a		2	1	1	2	1	2				2	3	2	3	2	2	3
gi|165932341	NP_291090.2	chymotrypsin-like elastase family, member 1																	
gi|167004347	NP_001107794.1	cadherin-related family member 5 isoform 1			2								2		7			1	
gi|16716569	NP_444473.1	protease, serine, 1		1			1												
gi|169790797	NP_001116075.1	Fc fragment of IgG binding protein		7	25	4	10	8	6	1	8	4	30	8	15	10	7	9	11
gi|169790977	NP_001116119.1	major urinary protein 10											4						
gi|170172530	NP_690052.2	immunoglobulin J chain precursor		3	3	1	3	3	4	2	4	3	4	3	3	3	3	4	3
gi|170650599	NP_613057.2	elongation factor G, mitochondrial precursor																	
gi|17647499	NP_058652.1	hemoglobin subunit beta-2																	
gi|18252782	NP_543120.1	antithrombin-III precursor			2														
gi|187283027	NP_001119790.1	hypothetical protein LOC242711						3					5	10	2	8		3	
gi|188219582	NP_036067.2	glucosamine-6-phosphate isomerase 1																	
gi|189491671	NP_766081.1	protein diaphanous homolog 2																	
gi|190684696	NP_033535.2	villin-1													1	1	1	1	1
gi|194328695	NP_035766.2	phospholipid scramblase 1													4				
gi|19527028	NP_598569.1	vigilin																	
gi|19527078	NP_598623.1	fibrinogen gamma chain precursor													12	2	2		
gi|19527082	NP_598628.1	epidermal growth factor receptor kinase substrate 8-like protein 3													2				
gi|19527104	NP_598649.1	acid sphingomyelinase-like phosphodiesterase 3b precursor											1		1				1
gi|20330802	NP_598738.1	serotransferrin precursor													3	3	1	1	
gi|209862915	NP_001129527.1	CD82 antigen isoform 2													2				
gi|21105853	NP_631937.1	placenta-specific gene 8 protein																	
gi|21312316	NP_082492.1	prostate stem cell antigen precursor		1	7	2	3	1	2	2	2	3	16	8	7	2	4	3	16
gi|213417962	NP_067369.2	Z-DNA-binding protein 1 isoform 1			1													1	
gi|21362335	NP_653120.1	carboxypeptidase A5 precursor																	
gi|21450325	NP_659172.1	flavin reductase																	
gi|21489935	NP_058654.1	keratin, type I cytoskeletal 14							1								2		
gi|21704144	NP_663544.1	S-adenosylmethionine synthase isoform type-2			1	2	1	3	3	2	2	2	1	1	1	1		1	
gi|21704206	NP_663578.1	carboxylesterase 2 precursor		1		1		1				1	1	3	1	1	1	1	1
gi|22122475	NP_666122.1	tetraspanin 8		3	6	3	1	2	2		1		4	5	76	37	16	5	11
gi|22122641	NP_666231.1	niban-like protein 1																	
gi|22164776	NP_666175.1	keratin, type II cytoskeletal 79																	
gi|22165388	NP_666261.1	hypothetical protein LOC230579													1				
gi|22219434	NP_671734.1	nuclear receptor-binding protein																	
gi|224809376	NP_067620.3	nicastrin precursor			1										5				
gi|224922803	NP_034404.3	glycerol-3-phosphate dehydrogenase, mitochondrial precursor													28	4	1		
gi|225007644	NP_001139360.1	zinc finger protein ZFAT isoform 1																	
gi|225637487	NP_032512.2	aminopeptidase N		50	18	40	29	47	32	15	25	31	32	66	157	87	64	65	77
gi|225690549	NP_796240.3	ribosome-releasing factor 2, mitochondrial isoform 1		1	1														1
gi|225735649	NP_001139592.1	sulfated glycoprotein 1 isoform A preproprotein												2	6	2	2	2	2
gi|226246531	NP_112467.2	phosphoglycerate kinase 2						1	1										
gi|226823250	NP_034954.2	myeloperoxidase precursor													45	2	8		
gi|226874906	NP_033562.3	14-3-3 protein epsilon																	
gi|226874935	NP_034712.2	inter-alpha-trypsin inhibitor heavy chain H2																	
gi|226958349	NP_033441.2	triosephosphate isomerase							1					2	3	1	1	1	
gi|226958351	NP_038762.1	sodium/potassium-transporting ATPase subunit alpha-4												1	3				1
gi|226958497	NP_035448.2	serum amyloid P-component precursor												4	140	61	49	9	7
gi|227116347	NP_001153027.1	platelet glycoprotein 4													1	1			
gi|227499103	NP_663392.2	aminopeptidase B isoform 1																	
gi|227908835	NP_835180.2	probable carboxypeptidase PM20D1 precursor												1	1	1			
gi|236468117	NP_001153622.1	amylase 2a4		4	5	1		1					15	4	12	4	8	11	1
gi|237874196	NP_001153858.1	dynein, axonemal, heavy chain 7B				1	1	3				1	1	1					
gi|23943876	NP_081194.1	zymogen granule membrane protein 16 precursor		43	41	33	38	49	52	20	43	35	72	45	73	22	23	30	25
gi|23956140	NP_079914.1	glycerophosphodiester phosphodiesterase domain-containing protein 1													5	1			
gi|23956196	NP_081487.1	annexin A13													1				

gi|23956222	NP_076224.1	actin-related protein 3																	
gi|242332572	NP_057930.2	ras GTPase-activating-like protein IQGAP1																	
gi|242397450	NP_035178.1	paired basic amino acid cleaving system 4		1	1														
gi|24496768	NP_038949.2	serine protease 30 precursor																	
gi|25141335	NP_082045.1	copine-3													1				
gi|251823804	NP_660108.2	chitinase-3-like protein 4 precursor													2		2		
gi|251823824	NP_082166.3	sushi domain-containing protein 2 isoform 1													1	1			
gi|253970410	NP_653127.2	hypothetical protein LOC78354													2	1	1		1
gi|253970442	NP_997125.2	Niemann-Pick C1-like protein 1 precursor		1	1	1	1		1				2	2	7	4	1	1	7
gi|254281348	NP_034022.2	chitinase-3-like protein 3 precursor													3	2	2	1	
gi|254540166	NP_071705.3	78 kDa glucose-regulated protein precursor													6	2	2		
gi|254692909	NP_001013044.2	transcription termination factor 2													1				1
gi|255069715	NP_077754.3	cytosol aminopeptidase		1	1				1										
gi|255522937	NP_079859.2	chymotrypsinogen B precursor		18	28	11	21	15	16	8	17	6	48	22	21	15	16	26	20
gi|255683322	NP_766413.2	probable G-protein coupled receptor 128 precursor													2				
gi|256222930	NP_663549.2	coiled-coil domain-containing protein 136									2								
gi|257467625	NP_001158127.1	Fc fragment of IgG binding protein-like																	
gi|258547152	NP_001158149.1	programmed cell death 6-interacting protein isoform 1																	
gi|260304980	NP_001159443.1	predicted gene 5803																	
gi|262073053	NP_081515.2	hypothetical protein LOC69864		1	1	1									4	1		1	4
gi|268836255	NP_573461.2	glycogen phosphorylase, liver form					2	2											
gi|269308203	NP_082776.2	calreticulin-3 isoform 1																	
gi|27151739	NP_032780.2	odorant binding protein Ia		1	12								65	4	1				
gi|27229028	NP_080265.2	pancreatic secretory granule membrane major glycoprotein GP2		3	3	1	1	1	1							1		4	
gi|27370012	NP_766288.1	CAAX prenyl protease 1 homolog													3				
gi|27370038	NP_766299.1	GTP-binding protein GUF1 homolog							2			1							
gi|27754136	NP_075675.2	acidic mammalian chitinase precursor		13	5	4	7	11	7	5	18	7	7	10	13	13	4	7	8
gi|282165827	NP_001028536.2	protocadherin 24		19	34	9	16	23	29	9	31	17	65	25	12	24	9	20	42
gi|28316756	NP_783589.1	histone cluster 1, H2aa			2														
gi|283483997	NP_001164474.1	maltase-glucoamylase		36	25	32	27	32	34	5	18	18	61	36	45	30	20	38	33
gi|294489324	NP_001170938.1	MHC class Ib T9													4				
gi|294610643	NP_076055.2	mucin-2		11	4	3	5	4	3	1	5	3	8	5	6	4	3	7	3
gi|294997230	NP_996988.2	histocompatibility 2, class II antigen A, beta 1 precursor													21	3	3	5	4
gi|29789211	NP_082202.1	carboxypeptidase A4 precursor		1		1	3	4	3	1	1	2	1	4	2		3	2	3
gi|29789343	NP_598677.1	eukaryotic translation initiation factor 3 subunit B																	
gi|29789389	NP_766172.1	inositol-tetrakisphosphate 1-kinase																	
gi|30410738	NP_848499.1	hypothetical protein LOC229688																	
gi|30425250	NP_780706.1	beta-actin-like protein 2													1			1	
gi|30519911	NP_848713.1	transgelin-2																	
gi|30725780	NP_849251.1	protein MAL2													3				
gi|31542362	NP_031672.2	ADP-ribosyl cyclase 1													6				
gi|31543113	NP_032905.2	plastin-2			1														
gi|31543255	NP_032630.2	neprilysin		26	13	8	10	13	7	1	7	3	39	14	54	15	2	9	18
gi|31543942	NP_033528.3	vinculin																	
gi|31543974	NP_061223.2	14-3-3 protein beta/alpha																	
gi|31543976	NP_061359.2	14-3-3 protein gamma																	
gi|31560030	NP_077745.2	tropomyosin alpha-1 chain isoform 3																	
gi|31560433	NP_059500.2	sorting nexin-3																	
gi|31560677	NP_032548.2	lactotransferrin precursor													2				
gi|31560731	NP_031534.2	V-type proton ATPase catalytic subunit A																	
gi|31980868	NP_034769.4	kallikrein-1 precursor		2	3	1	1	3	2	2	2	3	4	2	1	3	2	2	1
gi|31981542	NP_035308.2	myeloblastin precursor													5	1	2	1	
gi|31981562	NP_035229.2	pyruvate kinase isozymes M1/M2																	
gi|31981570	NP_035212.2	polymeric immunoglobulin receptor precursor		4	5	2	5	13	9	9	5	5	11	3	13	8	5	7	11
gi|31981716	NP_034508.2	histocompatibility 2, class II antigen A, alpha precursor													5	7	2	2	2
gi|31981890	NP_033865.2	beta-2-microglobulin precursor													2				
gi|31982030	NP_598557.3	rho GDP-dissociation inhibitor 1																	
gi|31982171	NP_032671.2	murinoglobulin-1 precursor			1								1	2	3	2	2	2	1

gi|31982186	NP_032643.2	malate dehydrogenase, mitochondrial precursor													1		1		
gi|31982199	NP_032611.2	meprin A subunit alpha		3		3	3	5	4		2	1	4	3	5	1	2	3	4
gi|33563236	NP_031512.1	rho GDP-dissociation inhibitor 2																	
gi|33563240	NP_033736.1	actin, alpha skeletal muscle												1		1			
gi|33563252	NP_034326.1	fibrinogen, alpha polypeptide isoform 2													6	2	2	1	
gi|33859474	NP_031784.1	complement receptor type 2													2				
gi|33859640	NP_035658.1	transaldolase																	
gi|33859686	NP_079976.1	phosphoglucomutase-2																	
gi|33859809	NP_862897.1	fibrinogen beta chain precursor													25	3	1		
gi|34328436	NP_081138.2	CD177 antigen precursor													11	3	6		
gi|34538600	NP_904330.1	cytochrome c oxidase subunit I													3				
gi|34538601	NP_904331.1	cytochrome c oxidase subunit II													3				
gi|34915988	NP_033898.1	basigin isoform 1				3	1	1		1	1	1	1	3	13	1	2		2
gi|36031132	NP_031530.2	sarcoplasmic/endoplasmic reticulum calcium ATPase 1		1	1	4								5	6			4	2
gi|37674236	NP_081201.2	pancreatic triacylglycerol lipase		38	58	63	32	26	26	31	41	32	72	45	68	119	41	57	87
gi|38076713	XP_357312.1	PREDICTED: similar to ferritin light chain 2		1	1		1	2	3		2	1	4		3				
gi|40556608	NP_032328.2	heat shock protein HSP 90-beta																	
gi|42415475	NP_035162.1	protein disulfide-isomerase precursor													6		1	1	
gi|45504394	NP_034708.1	integrin beta-1 precursor													7				
gi|46559389	NP_997507.1	angiotensin-converting enzyme isoform 1													3				
gi|50345978	NP_001002012.1	heat shock-related 70 kDa protein 2		1		1	1					1		1	2			1	1
gi|51010909	NP_001003405.1	trypsin 5		13	10	13	11	11	14	8	13	9	15	12	17	10	9	13	10
gi|51491845	NP_001003908.1	clathrin heavy chain 1																	
gi|51765047	XP_486246.1	PREDICTED: similar to Tubulin, alpha 3c isoform 1																	
gi|54312076	NP_079626.2	carboxypeptidase A1 precursor		28	68	51	91	457	151	101	145	66	109	82	6	47	43	45	23
gi|56090544	NP_001007553.1	intelectin-1b precursor		1	1	1									1				3
gi|56550071	NP_083982.1	pancreatic carboxypeptidase B1		13	37	28	35	79	41	9	22	9	88	51	54	23	17	27	19
gi|57222282	NP_001009546.1	N-acetylated-alpha-linked acidic dipeptidase-like protein		48	49	47	25	42	46	44	77	165	116	81	56	35	32	44	42
gi|61097906	NP_598917.1	alpha-actinin-1													3				
gi|61743961	NP_033773.1	AHNAK nucleoprotein isoform 1																	
gi|6671509	NP_031419.1	actin, cytoplasmic 1												1	1				
gi|6671539	NP_031464.1	fructose-bisphosphate aldolase A isoform 2																	
gi|6671664	NP_031623.1	calnexin precursor													2				
gi|6671672	NP_031630.1	F-actin-capping protein subunit alpha-2																	
gi|6671678	NP_031633.1	carbonic anhydrase 4 precursor													4				
gi|6671746	NP_031714.1	cofilin-2																	
gi|6677703	NP_033068.1	lithostathine-1 precursor													6	8	2		
gi|6677705	NP_033069.1	lithostathine-2 precursor													14	14	3		1
gi|6677837	NP_033140.1	protein S100-A9											1		33	11	5	4	3
gi|6678079	NP_033269.1	alpha-1-antitrypsin 1-1 precursor			4								1		3	1			
gi|6678085	NP_033272.1	alpha-1-antitrypsin 1-4 precursor			10													3	
gi|6678097	NP_033280.1	serpin B6 isoform b			12						1		16	2	3	1	1	2	6
gi|6678329	NP_033399.1	protein-glutamine gamma-glutamyltransferase 2																	
gi|6678439	NP_033456.1	anionic trypsin-2 precursor		195	103	274	299	703	545	384	309	145	338	318	139	154	90	171	67
gi|6678581	NP_033541.1	wiskott-Aldrich syndrome protein homolog																	
gi|6679293	NP_033428.1	peptidoglycan recognition protein 1 precursor													1	5	3		
gi|6679383	NP_032904.1	alpha-2-antiplasmin precursor																	
gi|6679439	NP_032933.1	peptidyl-prolyl cis-trans isomerase A																	
gi|6679509	NP_032979.1	Psp																	1
gi|6679653	NP_031960.1	glutamyl aminopeptidase		26	33	25	27	31	31	7	18	16	68	57	162	74	51	54	67
gi|6679939	NP_032111.1	glyceraldehyde-3-phosphate dehydrogenase, testis-specific																	3
gi|6679995	NP_032142.1	gamma-glutamyltranspeptidase 1		1	1			1				1	1	3	6	6	4	3	5
gi|6680231	NP_032279.1	high mobility group protein B3																	
gi|6680441	NP_032401.1	gastrotropin											1						
gi|6680586	NP_032484.1	serine protease inhibitor A3C precursor			5														
gi|6680636	NP_031424.1	adenosine deaminase													3	1			
gi|6680710	NP_031498.1	aquaporin-1			1								1		1				
gi|6680836	NP_031617.1	calreticulin precursor													2	7	2		

gi|6681297	NP_031945.1	chymotrypsin-like elastase family member 2A precursor			1	1		1		1	1	1	1	3	1	4	3	2	1
gi|67010039	NP_001019869.1	carboxypeptidase A2 precursor		23	13	9	17	30	23	10	39	4	17	9	5	14	9	18	15
gi|6753060	NP_033803.1	annexin A5													7				
gi|6753138	NP_033851.1	sodium/potassium-transporting ATPase subunit beta-1		2	4	3	2	2	2	1	3	3	5	9	34	9	6	4	8
gi|6753244	NP_033920.1	calmodulin																	
gi|6753322	NP_033967.1	T-complex protein 1 subunit delta																	
gi|6753428	NP_034027.1	creatine kinase U-type, mitochondrial precursor				1	1				1	1	2	3	8	4	3		1
gi|6753674	NP_034204.1	dipeptidyl peptidase 4 isoform 1		5	4	1	1	3	3	2	2	2	4	5	15	21	15	10	10
gi|6753798	NP_034298.1	prothrombin																	
gi|6753912	NP_034369.1	ferritin heavy chain		3			2	1	4		5		1						
gi|6754254	NP_034610.1	heat shock protein HSP 90-alpha																	
gi|6754388	NP_034714.1	intelectin-1a precursor												1	2		1	1	1
gi|6754480	NP_034792.1	keratin, type I cytoskeletal 13													1				
gi|6754534	NP_034837.1	galectin-6												1	14	6	1		1
gi|6754576	NP_034869.1	mucin-13 precursor		6	12	1	54		1		1	1	4	4	34	50	61	19	49
gi|6754658	NP_034907.1	Golli-Mbp isoform 1																	
gi|6754976	NP_035164.1	peroxiredoxin-1																	
gi|6754980	NP_035166.1	regenerating islet-derived protein 3-beta precursor		2	2	4	5	3	1		1		3	34	43	19	21	25	18
gi|6755090	NP_035237.1	phospholipase A2 precursor																	1
gi|6755100	NP_035249.1	proliferation-associated protein 2G4																	
gi|6755112	NP_035255.1	phospholipid transfer protein precursor															1		
gi|6755144	NP_035280.1	galectin-3-binding protein precursor														1		1	1
gi|6755210	NP_036005.1	26S proteasome non-ATPase regulatory subunit 13																	
gi|6755256	NP_035354.1	glycogen phosphorylase, muscle form												1					
gi|6755308	NP_035389.1	regenerating islet-derived protein 3-alpha precursor													6		1		
gi|6755310	NP_035390.1	regenerating islet-derived protein 3-gamma precursor				1	1							14	31	15	10	14	10
gi|6755364	NP_035424.1	activated RNA polymerase II transcriptional coactivator p15														1	1		
gi|6755372	NP_036182.1	40S ribosomal protein S3																	
gi|6755863	NP_035761.1	endoplasmin																	
gi|6755963	NP_035824.1	voltage-dependent anion-selective channel protein 1													3	1			
gi|6755965	NP_035825.1	voltage-dependent anion-selective channel protein 2												3	20	3	3	1	1
gi|6755967	NP_035826.1	voltage-dependent anion-selective channel protein 3													6				
gi|6756037	NP_035868.1	14-3-3 protein eta																	
gi|68226731	NP_032968.2	puromycin-sensitive aminopeptidase																	
gi|6996913	NP_031611.1	annexin A2																	
gi|70778976	NP_032854.2	phosphoglycerate kinase 1		1														2	
gi|71043961	NP_075822.3	trypsinogen 7		3	2	3	3	5	2	2	4	4	5	6	7	5	5	7	6
gi|7110683	NP_035174.1	phosphoenolpyruvate carboxykinase, cytosolic [GTP]					1				1	1							
gi|7304963	NP_038913.1	chloride intracellular channel protein 4																	
gi|7305189	NP_038593.1	integrin alpha-3													6				
gi|7305453	NP_038678.1	protein S100-A8																	
gi|7305599	NP_038725.1	transthyretin									2		3		1	1			
gi|75677435	NP_031463.2	fatty aldehyde dehydrogenase													5				
gi|76443665	NP_001029047.1	chymotrypsin-C precursor		409	464	489	254	541	504	249	410	231	684	366	348	195	174	409	275
gi|76881807	NP_033270.3	alpha-1-antitrypsin 1-2 precursor		5	18	11		1					10	10	15	4	3	10	5
gi|7710018	NP_038543.1	erythrocyte band 7 integral membrane protein													10	6	4		
gi|7949064	NP_032482.1	kallikrein 1-related peptidase b5 precursor		2	2		1	3	1	1	3	1	11		4	1	1	1	
gi|82796081	XP_928726.1	PREDICTED: hypothetical protein																	
gi|82799602	XP_913793.1	PREDICTED: similar to Glyceraldehyde-3-phosphate dehydrogenase																	
gi|82802624	XP_920189.1	PREDICTED: similar to hCG2001479		9	4	11	7	10	11	2	10	3	20	11	25	5	3	10	15
gi|82882188	XP_922713.1	PREDICTED: similar to intelectin		1	1	1	2	1	1	1	1	1	1	1	6	2	2	1	2
gi|82895363	XP_919532.1	PREDICTED: hypothetical protein		2	4	4	1	4	2				8	4	16	5	3	3	2
gi|82935925	XP_913239.1	PREDICTED: similar to myosin, heavy polypeptide 3, skeletal muscle, embryonic				2	2			1				3	2	3	2	2	2
gi|82998325	XP_904967.1	PREDICTED: hypothetical protein isoform 7																	
gi|83008262	XP_913818.1	PREDICTED: similar to Ab 414.2 heavy chain variable and joining region						1			2		1		2			1	2
gi|83014618	XP_909851.1	PREDICTED: similar to Igh protein		1	1	1	3	2	1	1	2	1	3	2	2	3	1		2
gi|83020812	XP_920195.1	PREDICTED: similar to Adaptor-related protein complex AP-1, sigma 3																	

gi|83029092	XP_357633.3	PREDICTED: similar to Ig kappa chain V-V region L7 precursor					1		1		2		2		1		1		3
gi|83582782	NP_081562.2	angiotensin-converting enzyme 2 precursor		2	1	1	1	5	1	1	1	1	1	1	8	7	7	7	4
gi|8392847	NP_058556.1	alpha-centractin																	
gi|84781771	NP_001034085.1	trypsin 10		3	3	3	3	3	2	1	2	2	4	6	6	3	3	4	4
gi|84781781	NP_001028485.1	28 kDa heat- and acid-stable phosphoprotein																	
gi|84872231	NP_950181.2	ectonucleotide pyrophosphatase/phosphodiesterase family member 4 precursor											1		3				
gi|8567336	NP_059502.1	calcium-activated chloride channel regulator 1 precursor		5	11		3	8	11		6	2	33	6	5	8	2	2	8
gi|85701680	NP_001028349.1	keratin, type II cytoskeletal 2 oral													1				
gi|85701714	NP_001028371.1	calcium activated chloride channel		2	14	1	1	1	1		3	1	20	7	76	21	15	7	14
gi|85719299	NP_001034274.1	carcinoembryonic antigen-related cell adhesion molecule 1 isoform 1		1	1	1	1	1	1					1	6	3		2	2
gi|87239967	NP_031795.2	deleted in malignant brain tumors 1 protein		7	3	2	10	9	10	1	9	5	10	14	43	41	28	7	10
gi|8850219	NP_059066.1	haptoglobin precursor													6	1	1		
gi|9055168	NP_061300.1	neutral ceramidase		26	56	3	17	9	10	45	7	8	93	43	44	36	21	13	41
gi|94363353	XP_484859.3	PREDICTED: similar to MYL6 protein																	
gi|94366612	XP_988506.1	PREDICTED: hypothetical protein														1			
gi|94377669	XP_993008.1	PREDICTED: similar to kappa-tnp V-J			2														
gi|94377671	XP_993123.1	PREDICTED: similar to anti-A/U antibody													2		1	1	1
gi|94378251	XP_981474.1	PREDICTED: similar to histone H4			9										2	2	1	1	1
gi|94397546	XP_982057.1	PREDICTED: similar to ZFH-5																	
gi|94400086	XP_984563.1	PREDICTED: similar to Ribosomal protein S27a													3				
gi|94400195	XP_001003388.1	PREDICTED: similar to MSP23 isoform 2																	
gi|94407590	XP_898907.2	PREDICTED: similar to odorant binding protein Ib		4	22	4	1	2	1	6	5	5	91	19	9	8	4		3
gi|9790069	NP_062667.1	spliceosome RNA helicase Bat1																	
gi|9790073	NP_062727.1	cadherin-17 precursor		1	1	2	2	2	1			2	2	11	22	3	2	4	2
gi|9790141	NP_062798.1	actin-related protein 2/3 complex subunit 3																	
gi|9910294	NP_064340.1	keratin, type II cytoskeletal 71			2	1	1				1	1	2		2		1		

Table S1B: Total filter-passing commensal protein identifications

				Biological replicate 1															
				CONTROL								INFECTED							
Reference	Gene symbol	Protein description	DAY ->	-1	1	3	6	10	14	21	28	-1	1	3	6	10	14	21	28
JCVIGM_070246_20110307	gpmI	2,3-bisphosphoglycerate-independent phosphoglycerate mutase																	
JCVIGM_091222_20110307	gpmI	2,3-bisphosphoglycerate-independent phosphoglycerate mutase									1	1							
JCVIGM_199914_20110307		2-dehydro-3-deoxygluconokinase																	
JCVIGM_044539_20110307	eda	2-dehydro-3-deoxyphosphogluconate aldolase/4-hydroxy-2-oxoglutarate aldolase																	
JCVIGM_057836_20110307	eda	2-dehydro-3-deoxyphosphogluconate aldolase/4-hydroxy-2-oxoglutarate aldolase						1				1							
JCVIGM_092015_20110307	eda	2-dehydro-3-deoxyphosphogluconate aldolase/4-hydroxy-2-oxoglutarate aldolase					1												
JCVIGM_360038_20110307	yhaE	2-hydroxy-3-oxopropionate reductase													1			1	
JCVIGM_374955_20110307		2-hydroxy-3-oxopropionate reductase					1												
JCVIGM_319522_20110307	kdsA	3-deoxy-8-phosphooctulonate synthase																	
JCVIGM_196595_20110307		3-hydroxyacyl-coA dehydrogenase			1		1			1	1	1	1	2					
JCVIGM_037673_20110307	hbd	3-hydroxybutyryl-coA dehydrogenase																	
JCVIGM_037995_20110307	pdxB	4-phosphoerythronate dehydrogenase																	
JCVIGM_259097_20110307	rplJ	50S ribosomal protein L10																	
JCVIGM_024389_20110307	rplK	50S ribosomal protein L11																	
JCVIGM_045877_20110307	rplK	50S ribosomal protein L11										1	1						
JCVIGM_059265_20110307	rplK	50S ribosomal protein L11		1	2	1	2	1	1				1	2	1				
JCVIGM_033470_20110307	rplE	50S ribosomal protein L5					1				1								
JCVIGM_155746_20110307	rplE	50S ribosomal protein L5						1				1							
JCVIGM_232804_20110307	rplE	50S ribosomal protein L5				1		1											
JCVIGM_020270_20110307	rplL	50S ribosomal protein L7/L12																	
JCVIGM_125249_20110307	rplL	50S ribosomal protein L7/L12																	
JCVIGM_100458_20110307	ribH	6,7-dimethyl-8-ribityllumazine synthase			1														
JCVIGM_013177_20110307		60 kDa chaperonin																	
JCVIGM_213822_20110307		60 kDa chaperonin																	

JCVIGM_123049_20110307		ABC transporter periplasmic-binding protein ytfQ																	
JCVIGM_295655_20110307		ABC transporter related											1						
JCVIGM_097008_20110307		ABC transporter, ATP-binding protein										1							
JCVIGM_168201_20110307		ABC transporter, ATP-binding protein																	
JCVIGM_070783_20110307		ABC transporter, permease protein																	
JCVIGM_179750_20110307		ABC transporter, substrate-binding protein																	
JCVIGM_284177_20110307		ABC transporter, substrate-binding protein, family 1						1					1						
JCVIGM_284885_20110307		ABC-type dipeptide transport system, periplasmic component																	
JCVIGM_168588_20110307		ABC-type sugar transport system, periplasmic component											1						
JCVIGM_297706_20110307		ABC-type sugar transport system, periplasmic component					1	2		4									
JCVIGM_162628_20110307		ABC-type transport system, substrate-binding component																	
JCVIGM_197130_20110307		ABC-type xylose transport system, periplasmic component					1	1				1	1						
JCVIGM_034585_20110307	ackA	acetate kinase																	
JCVIGM_090369_20110307	ackA	acetate kinase						1	1										
JCVIGM_103872_20110307	ackA	acetate kinase																	
JCVIGM_105479_20110307	ackA	acetate kinase																	
JCVIGM_313779_20110307	ackA	acetate kinase																	
JCVIGM_120726_20110307	ilvC	acetohydroxy acid isomeroreductase, catalytic domain																	
JCVIGM_136868_20110307	ilvC	acetohydroxy acid isomeroreductase, catalytic domain		2	5	2	4	2	1	3	6	4	6	2		3			
JCVIGM_141542_20110307	ilvC	acetohydroxy acid isomeroreductase, catalytic domain			1	2	3	2		2	3	5	1	2	1				
JCVIGM_256491_20110307	ilvC	acetohydroxy acid isomeroreductase, catalytic domain																	
JCVIGM_298658_20110307	ilvC	acetohydroxy acid isomeroreductase, catalytic domain																	
JCVIGM_383122_20110307	ilvC	acetohydroxy acid isomeroreductase, catalytic domain				1													
JCVIGM_029818_20110307	ilvN	acetolactate synthase, small subunit																	
JCVIGM_125179_20110307		acetyl-coA acetyltransferases																	
JCVIGM_101296_20110307		acetyl-coA C-acetyltransferase									1								
JCVIGM_182295_20110307		acetyl-coA C-acetyltransferase				3	2	3		1	4	1	2	1					
JCVIGM_234799_20110307		acetyl-coA C-acetyltransferase																	
JCVIGM_306189_20110307	phaA	acetyl-CoA C-acetyltransferase																	
JCVIGM_299975_20110307		acyltransferase family																	
JCVIGM_058622_20110307	purA	adenylosuccinate synthase					1		2	1		1							

JCVIGM_064826_20110307	purA	adenylosuccinate synthase																	
JCVIGM_092236_20110307	purA	adenylosuccinate synthase																	
JCVIGM_092238_20110307	purA	adenylosuccinate synthase					2												
JCVIGM_108189_20110307		ahpC/TSA family																	
JCVIGM_145159_20110307	ald	alanine dehydrogenase																	
JCVIGM_391701_20110307	tdh	alcohol dehydrogenase groES domain protein		1									1						
JCVIGM_144742_20110307		alcohol dehydrogenase, iron-dependent																	
JCVIGM_147392_20110307		aldehyde oxidase and xanthine dehydrogenase molybdopterin binding protein					2												
JCVIGM_050239_20110307		alpha-amylase																	
JCVIGM_248140_20110307		alpha-D-mannosidase																	
JCVIGM_337826_20110307		alpha-D-mannosidase																	
JCVIGM_358636_20110307		alpha-D-mannosidase											1						
JCVIGM_076247_20110307		alpha-galactosidase																	
JCVIGM_090969_20110307		alpha-galactosidase																	
JCVIGM_235809_20110307		alpha-galactosidase																	
JCVIGM_094959_20110307		alpha-N-arabinofuranosidase							1	1									
JCVIGM_179557_20110307		alpha-N-arabinofuranosidase						1											
JCVIGM_043430_20110307		arabinogalactan endo-1,4-beta-galactosidase																	
JCVIGM_054434_20110307		arabinogalactan endo-1,4-beta-galactosidase		1				1	1	1									
JCVIGM_030203_20110307	fucI	arabinose isomerase																	
JCVIGM_108898_20110307	fucI	arabinose isomerase			2					1		1	3						
JCVIGM_151443_20110307	fucI	arabinose isomerase					1			2			3						
JCVIGM_188730_20110307	fucI	arabinose isomerase																	
JCVIGM_191949_20110307	fucI	arabinose isomerase																	
JCVIGM_339907_20110307	fucI	arabinose isomerase																	
JCVIGM_341670_20110307	fucI	arabinose isomerase											2						
JCVIGM_019632_20110307	arcA	arginine deiminase																	
JCVIGM_030954_20110307	asnB	asparagine synthase (glutamine-hydrolyzing)																	
JCVIGM_149591_20110307	aspA	aspartate ammonia-lyase																	

JCVIGM_076159_20110307	asd	aspartate-semialdehyde dehydrogenase					1												
JCVIGM_163401_20110307	asd	aspartate-semialdehyde dehydrogenase																	
JCVIGM_146114_20110307	aspS	aspartate--tRNA ligase																	
JCVIGM_225435_20110307	atpA	ATP synthase F1, alpha subunit																	
JCVIGM_193732_20110307		ATP synthase subunit a																	
JCVIGM_181280_20110307		B12 binding domain					1	3	2	1									
JCVIGM_335931_20110307		basic membrane lipoprotein																	
JCVIGM_191196_20110307		beta-galactosidase																	
JCVIGM_065702_20110307	bglA	beta-glucosidase A																	
JCVIGM_026370_20110307		beta-glucosidase-n-terminal domain-containing protein																	
JCVIGM_156507_20110307		beta-lactamase																	
JCVIGM_042339_20110307	purH	bifunctional purine biosynthesis protein purH								1									
JCVIGM_036791_20110307	buk	butyrate kinase									1								
JCVIGM_216035_20110307		butyryl-coA dehydrogenase																	
JCVIGM_265820_20110307		butyryl-coA dehydrogenase					1	1		1		1	1	1					
JCVIGM_290061_20110307		butyryl-coA dehydrogenase																	
JCVIGM_329946_20110307	bcd2	butyryl-coA dehydrogenase																	
JCVIGM_040543_20110307	c7d2-bg	C7D2-BG protein																	
JCVIGM_017812_20110307		carbohydrate ABC transporter substrate-binding protein, CUT1 family				1													
JCVIGM_055645_20110307		carbohydrate ABC transporter substrate-binding protein, CUT1 family				2	1	1	1	2		1							
JCVIGM_306701_20110307		carbohydrate ABC transporter substrate-binding protein, CUT1 family			1			2					1						
JCVIGM_074670_20110307		carbohydrate ABC transporter substrate-binding protein, CUT1 family		5		1	2	3				3							
JCVIGM_324855_20110307		carbohydrate ABC transporter, carbohydrate-binding protein			1		1						2						
JCVIGM_049249_20110307	cooS	carbon-monoxide dehydrogenase, catalytic subunit			1	1			1	1		1							
JCVIGM_000407_20110307	dnaK	chaperone protein dnaK							1										
JCVIGM_021700_20110307	dnaK	chaperone protein dnaK							1										
JCVIGM_031425_20110307	dnaK	chaperone protein dnaK							2			1							
JCVIGM_032528_20110307	dnaK	chaperone protein dnaK					1												
JCVIGM_034511_20110307	dnaK	chaperone protein dnaK																	
JCVIGM_043298_20110307	dnaK	chaperone protein dnaK							1										

JCVIGM_062282_20110307	dnaK	chaperone protein dnaK					1		1										
JCVIGM_122947_20110307	dnaK	chaperone protein dnaK		1			1			1				1					
JCVIGM_147511_20110307	dnaK	chaperone protein dnaK							1			1	1	1					
JCVIGM_213960_20110307	dnaK	chaperone protein dnaK					1												
JCVIGM_236768_20110307	dnaK	chaperone protein dnaK							2										
JCVIGM_086927_20110307	groS	chaperonin 10 Kd subunit		1			2			1	3								
JCVIGM_187049_20110307	groS	chaperonin 10 Kd subunit		6		2	4	4	8	2		2							
JCVIGM_213821_20110307	groS	chaperonin 10 Kd subunit																	
JCVIGM_401604_20110307	groS	chaperonin 10 Kd subunit		1				1											
JCVIGM_413734_20110307		CO dehydrogenase/acetyl-coA synthase complex, beta subunit																	
JCVIGM_049251_20110307	cdhC	CO dehydrogenase/CO-methylating acetyl-coA synthase complex, beta subunit					2		1										
JCVIGM_339054_20110307		coenzyme B12-dependent glycerol dehydratase, large subunit																	
JCVIGM_212787_20110307		cof-like hydrolase																	
JCVIGM_127608_20110307		ComEC/Rec2-like protein																	
JCVIGM_286641_20110307		conserved hypothetical protein			1				1				1						
JCVIGM_049253_20110307		corrinoid/iron-sulfur protein, large subunit																	
JCVIGM_049252_20110307		corrinoid/iron-sulfur protein, small subunit																	
JCVIGM_022873_20110307	cysK	cysteine synthase A				2		1		1									
JCVIGM_307840_20110307		dehydrogenase, FMN-dependent											2						
JCVIGM_102159_20110307	deoC	deoxyribose-phosphate aldolase		1			2		1	1		1							
JCVIGM_056340_20110307		diaminopimelate dehydrogenase																	
JCVIGM_104884_20110307		diaminopimelate dehydrogenase																	
JCVIGM_002401_20110307	lpdA	dihydrolipoyl dehydrogenase																	
JCVIGM_139913_20110307	pyrD	dihydroorotate oxidase																	
JCVIGM_049254_20110307		dihydropteroate synthase, DHPS																	
JCVIGM_130560_20110307	ilvD	dihydroxy-acid dehydratase																	
JCVIGM_377265_20110307	disA	DNA integrity scanning protein disA					1												
JCVIGM_176486_20110307	dnaN	DNA polymerase III, beta subunit											1						
JCVIGM_228057_20110307	etfB	electron transfer flavoprotein, alpha subunit				1													
JCVIGM_310193_20110307	etfB	electron transfer flavoprotein, alpha subunit				1	1			1		1							

JCVIGM_316234_20110307	etfA	electron transfer flavoprotein, alpha subunit/fixB family protein																	
JCVIGM_083913_20110307	etfB	electron transfer flavoprotein, beta subunit																	
JCVIGM_215068_20110307	etfB	electron transfer flavoprotein, beta subunit																	
JCVIGM_316431_20110307		electron transfer flavoprotein, beta subunit																	
JCVIGM_041452_20110307	tuf	elongation factor Tu (fragment)														1			
JCVIGM_148583_20110307		elongation factor Tu (fragment)																	
JCVIGM_209439_20110307	tuf	elongation factor Tu (fragment)					1												
JCVIGM_239217_20110307	tuf1	elongation factor Tu 1																	
JCVIGM_045263_20110307		extracellular solute-binding protein family 1		1	1	1	2	1	1		1	1	1	1					
JCVIGM_088711_20110307		extracellular solute-binding protein family 1		1	1	1	1	1	4			2	1						
JCVIGM_096968_20110307		extracellular solute-binding protein family 1																	
JCVIGM_108783_20110307		extracellular solute-binding protein family 1		6		1	6	4	5	2									
JCVIGM_212868_20110307		extracellular solute-binding protein family 1																	
JCVIGM_275107_20110307		extracellular solute-binding protein family 1																	
JCVIGM_291167_20110307		extracellular solute-binding protein family 1		3				1				1							
JCVIGM_192080_20110307		extracellular solute-binding protein family 5					1		1										
JCVIGM_259518_20110307		ferritin 1																	
JCVIGM_308648_20110307		fibronectin type III domain																	
JCVIGM_400677_20110307		fla1 flagellin		1		1				2	2	1		1		2			
JCVIGM_423664_20110307		fla1 flagellin (fragment)																	
JCVIGM_430871_20110307		fla3 flagellin																	
JCVIGM_416087_20110307	flgE1	flagellar hook protein flgE1																	
JCVIGM_077354_20110307		flagellin (fragment)		1															
JCVIGM_172838_20110307		flagellin (fragment)																	
JCVIGM_022208_20110307		flagellin and related hook-associated proteins		1		3		1	1		2	1	1	1					
JCVIGM_154194_20110307		flagellin and related hook-associated proteins																	
JCVIGM_190042_20110307		flagellin and related hook-associated proteins																	
JCVIGM_389298_20110307		flagellin and related hook-associated proteins								2	2								
JCVIGM_188452_20110307		flagellin domain protein									1								
JCVIGM_346020_20110307		flagellin domain protein																	
JCVIGM_391229_20110307		flagellin domain protein																	

JCVIGM_069861_20110307	fliC1	flagellin fliC1																	
JCVIGM_410616_20110307	fliC1	flagellin fliC1																	
JCVIGM_152287_20110307	fliC2	flagellin fliC2																	
JCVIGM_177955_20110307	fliC2	flagellin fliC2																	
JCVIGM_262501_20110307	fliC2	flagellin fliC2		1			1	3		1	3								
JCVIGM_187473_20110307	fliC4	flagellin fliC4										1							
JCVIGM_189905_20110307	fliC5	flagellin fliC5																	
JCVIGM_392640_20110307		flagellin protein																	
JCVIGM_402856_20110307		flagellin protein		1															
JCVIGM_414958_20110307		flagellin protein					1				1								
JCVIGM_424682_20110307		flagellin protein																	
JCVIGM_152365_20110307	fprA	flavodoxin																	
JCVIGM_148596_20110307		formate acetyltransferase (fragment)											1						
JCVIGM_185080_20110307		formate acetyltransferase (fragment)																	
JCVIGM_246004_20110307		formate acetyltransferase (fragment)																	
JCVIGM_032907_20110307	pflB	formate C-acetyltransferase		1								2	1						
JCVIGM_043008_20110307	pflB	formate C-acetyltransferase		6		3	4	5	1	2	2	4	3						
JCVIGM_157210_20110307		formate C-acetyltransferase																	
JCVIGM_229801_20110307		formate C-acetyltransferase																	
JCVIGM_249731_20110307		formate C-acetyltransferase																	
JCVIGM_282796_20110307		formate C-acetyltransferase																	
JCVIGM_317542_20110307		formate C-acetyltransferase					1						1						
JCVIGM_356609_20110307		formate C-acetyltransferase																	
JCVIGM_417381_20110307		formate C-acetyltransferase																	
JCVIGM_078607_20110307	fhs	formate--tetrahydrofolate ligase																	
JCVIGM_118473_20110307	fhs	formate--tetrahydrofolate ligase		7	3	3	4	4	2	3	4	2	3						
JCVIGM_123964_20110307	fhs	formate--tetrahydrofolate ligase		5	7	5	6	6	1	9	3	2	5						
JCVIGM_138264_20110307	fhs	formate--tetrahydrofolate ligase		1								1	1						
JCVIGM_162244_20110307	fhs	formate--tetrahydrofolate ligase																	
JCVIGM_176151_20110307	fhs	formate--tetrahydrofolate ligase		1				1					1	1					
JCVIGM_245005_20110307	fhs	formate--tetrahydrofolate ligase				1		2											

JCVIGM_400807_20110307	fhs	formate--tetrahydrofolate ligase		1								1	1						
JCVIGM_122704_20110307	fhs1	formate--tetrahydrofolate ligase 1			1					1	1								
JCVIGM_146104_20110307	fhs2	formate--tetrahydrofolate ligase 2		3	10	6	9	7	4	6	1	4	3			2			
JCVIGM_032208_20110307	frc	formyl-coA transferase							1										
JCVIGM_065785_20110307	frc	formyl-CoA transferase				1				1	1				1				
JCVIGM_070965_20110307	frc	formyl-coA transferase		1															
JCVIGM_132967_20110307	frc	formyl-coA transferase		3			2	2	2	3	1	2	1	1	1				
JCVIGM_231668_20110307	frc	formyl-coA transferase																	
JCVIGM_254826_20110307	frc	formyl-coA transferase																	
JCVIGM_051280_20110307		fructokinase					1		1										
JCVIGM_079013_20110307		fructose-1,6-bisphosphate aldolase, class II						1			1								
JCVIGM_087474_20110307	fba	fructose-1,6-bisphosphate aldolase, class II		5		9	1	5		2	10	3							
JCVIGM_118274_20110307	fba	fructose-1,6-bisphosphate aldolase, class II					2	1		1	1								
JCVIGM_000284_20110307	fsa	fructose-6-phosphate aldolase														1			
JCVIGM_153991_20110307	fsa	fructose-6-phosphate aldolase		1	2		3		1	2	1	2	2	2					
JCVIGM_393658_20110307	fsa	fructose-6-phosphate aldolase				1		1											
JCVIGM_231559_20110307		fructose-bisphosphate aldolase																	
JCVIGM_089609_20110307		fructose-bisphosphate aldolase class-I																	
JCVIGM_336730_20110307		galactose ABC transporter, periplasmic galactose-binding protein											1	1					
JCVIGM_027728_20110307		galactose/glucose-binding lipoprotein				1	3	4	2	1		3							
JCVIGM_266085_20110307	galT	galactose-1-phosphate uridylyltransferase galT																	
JCVIGM_165424_20110307		gliding motility-related protein																	
JCVIGM_351864_20110307		glu/leu/phe/val dehydrogenase, dimerization domain protein																	
JCVIGM_138714_20110307		gluconate 5-dehydrogenase																	
JCVIGM_002867_20110307	pgi	glucose-6-phosphate isomerase			1	1	1	1	1			1	1		1				
JCVIGM_015397_20110307	pgi	glucose-6-phosphate isomerase									1								
JCVIGM_015745_20110307	pgi	glucose-6-phosphate isomerase		1	1	1	1		1	1		1	1	1					
JCVIGM_026127_20110307	pgi	glucose-6-phosphate isomerase																	
JCVIGM_030449_20110307	pgi	glucose-6-phosphate isomerase		1	2	1	2	1		2	2	1	1	1					
JCVIGM_042043_20110307	pgi	glucose-6-phosphate isomerase																	

JCVIGM_054834_20110307	pgi	glucose-6-phosphate isomerase																	
JCVIGM_073687_20110307	pgi	glucose-6-phosphate isomerase			2	2		2	2	1	2		3	1		1			
JCVIGM_075891_20110307	pgi	glucose-6-phosphate isomerase																	
JCVIGM_080716_20110307	pgi	glucose-6-phosphate isomerase					2	1		1	1	2							
JCVIGM_091996_20110307	pgi	glucose-6-phosphate isomerase		1		1	2	1	1	1		1	2	1		1			
JCVIGM_116144_20110307	pgi	glucose-6-phosphate isomerase																	
JCVIGM_147397_20110307	pgi	glucose-6-phosphate isomerase		1			2	2	2				2			1			
JCVIGM_150388_20110307	pgi	glucose-6-phosphate isomerase																	
JCVIGM_154594_20110307	pgi	glucose-6-phosphate isomerase									1		2						
JCVIGM_170286_20110307	pgi	glucose-6-phosphate isomerase						1	3	2									
JCVIGM_208717_20110307	pgi	glucose-6-phosphate isomerase																	
JCVIGM_221773_20110307	pgi	glucose-6-phosphate isomerase		1					1					1			1		
JCVIGM_229071_20110307		glucose-6-phosphate isomerase					1			2		1	1						
JCVIGM_245845_20110307	pgi	glucose-6-phosphate isomerase																	
JCVIGM_257476_20110307	pgi	glucose-6-phosphate isomerase																	
JCVIGM_261055_20110307	pgi	glucose-6-phosphate isomerase																	
JCVIGM_286986_20110307	pgi	glucose-6-phosphate isomerase					1												
JCVIGM_296222_20110307	pgi	glucose-6-phosphate isomerase		1		1	1	1		1		1	1						
JCVIGM_296223_20110307	pgi	glucose-6-phosphate isomerase																	
JCVIGM_346043_20110307	pgi	glucose-6-phosphate isomerase																	
JCVIGM_407434_20110307	pgi	glucose-6-phosphate isomerase																	
JCVIGM_433684_20110307	pgi	glucose-6-phosphate isomerase																	
JCVIGM_036452_20110307	pgiA	glucose-6-phosphate isomerase A																	
JCVIGM_001673_20110307	uxaC	glucuronate isomerase																	
JCVIGM_001890_20110307	uxaC	glucuronate isomerase																	
JCVIGM_105608_20110307	uxaC	glucuronate isomerase																	
JCVIGM_028337_20110307		glutamate dehydrogenase																	
JCVIGM_079729_20110307	GDH	glutamate dehydrogenase (fragment)					1	2		1	1								
JCVIGM_026222_20110307		glutamate dehydrogenase (NADP(+))																	
JCVIGM_051813_20110307		glutamate dehydrogenase, nADP-specific				1													

JCVIGM_081547_20110307		glutamate dehydrogenase, NADP-specific		6	2	4	10	8	7	8		5	4						
JCVIGM_083070_20110307		glutamate dehydrogenase, NADP-specific											2						
JCVIGM_112581_20110307		glutamate dehydrogenase, NADP-specific						1				1	1						
JCVIGM_235230_20110307		glutamate dehydrogenase, NADP-specific		1	3	4		1		4	2	2	3	7					
JCVIGM_395419_20110307		glutamate dehydrogenase, NADP-specific																	
JCVIGM_000822_20110307	gdhB	glutamate dehydrogenase, NAD-specific														1			
JCVIGM_031962_20110307	gdhB	glutamate dehydrogenase, NAD-specific																	
JCVIGM_033111_20110307	gdhB	glutamate dehydrogenase, NAD-specific																	
JCVIGM_044177_20110307	gdhB	glutamate dehydrogenase, NAD-specific																	
JCVIGM_072261_20110307	gdh	glutamate dehydrogenase, NAD-specific						3											
JCVIGM_124713_20110307	gdhB	glutamate dehydrogenase, NAD-specific																	
JCVIGM_030438_20110307		glutamate dehydrogenase/leucine dehydrogenase			2	1	1	1			2		2						
JCVIGM_067262_20110307		glutamate dehydrogenase/leucine dehydrogenase		1															
JCVIGM_071296_20110307		glutamate dehydrogenase/leucine dehydrogenase																	
JCVIGM_130579_20110307		glutamate dehydrogenase/leucine dehydrogenase																	
JCVIGM_141521_20110307		glutamate dehydrogenase/leucine dehydrogenase		2	2	2	2	1	2	2	3	1	2	2					
JCVIGM_226798_20110307		glutamate dehydrogenase/leucine dehydrogenase																	
JCVIGM_167424_20110307	gltA	glutamate synthase (NADPH), homotetrameric																	
JCVIGM_009248_20110307	gap	glyceraldehyde 3-phosphate dehydrogenase, C-terminal domain																	
JCVIGM_027319_20110307		glyceraldehyde 3-phosphate dehydrogenase, C-terminal domain										2				1			
JCVIGM_075124_20110307		glyceraldehyde 3-phosphate dehydrogenase, C-terminal domain																	
JCVIGM_079320_20110307		glyceraldehyde 3-phosphate dehydrogenase, C-terminal domain														1			
JCVIGM_083011_20110307		glyceraldehyde 3-phosphate dehydrogenase, C-terminal domain																	
JCVIGM_088090_20110307		glyceraldehyde 3-phosphate dehydrogenase, C-terminal domain					1												
JCVIGM_141419_20110307		glyceraldehyde 3-phosphate dehydrogenase, C-terminal domain																	
JCVIGM_159787_20110307		glyceraldehyde 3-phosphate dehydrogenase, C-terminal domain																	
JCVIGM_169660_20110307		glyceraldehyde 3-phosphate dehydrogenase, C-terminal domain																	
JCVIGM_170407_20110307		glyceraldehyde 3-phosphate dehydrogenase, C-terminal domain																	
JCVIGM_189194_20110307		glyceraldehyde 3-phosphate dehydrogenase, C-terminal domain		2	2	1	2	1	1	1	2	3	2	2	2	1	1		1
JCVIGM_224384_20110307		glyceraldehyde 3-phosphate dehydrogenase, C-terminal domain		2	3	2	1	2		1		3	3			3			
JCVIGM_268659_20110307		glyceraldehyde 3-phosphate dehydrogenase, C-terminal domain											2						

JCVIGM_313872_20110307		glyceraldehyde 3-phosphate dehydrogenase, C-terminal domain																	
JCVIGM_319196_20110307		glyceraldehyde 3-phosphate dehydrogenase, C-terminal domain																	
JCVIGM_360253_20110307		glyceraldehyde 3-phosphate dehydrogenase, C-terminal domain														1		1	
JCVIGM_100377_20110307	gap	glyceraldehyde-3-phosphate dehydrogenase, type I		4	4	2	3	2	1	2	2	4	4	1					
JCVIGM_294702_20110307	pduC	glycerol dehydratase large subunit																	
JCVIGM_338330_20110307		glycerol dehydrogenase and related enzymes																	
JCVIGM_213371_20110307	glyA	glycine hydroxymethyltransferase																	
JCVIGM_015371_20110307		histidine kinase																	
JCVIGM_369000_20110307		hydrogenase, Fe-only																	
JCVIGM_048518_20110307		hydrolyase, tartrate beta subunit/fumarate domain protein, Fe-S type																	
JCVIGM_000577_20110307		hypothetical protein																	
JCVIGM_001656_20110307		hypothetical protein										1	1						
JCVIGM_004031_20110307		hypothetical protein											1						
JCVIGM_006083_20110307		hypothetical protein																	
JCVIGM_006988_20110307		hypothetical protein		2	4							2	1						
JCVIGM_007410_20110307		hypothetical protein																	
JCVIGM_009250_20110307		hypothetical protein																	
JCVIGM_010628_20110307		hypothetical protein					1												
JCVIGM_011271_20110307		hypothetical protein		1				2			1								
JCVIGM_011336_20110307		hypothetical protein			1														
JCVIGM_012967_20110307		hypothetical protein		1					1			1				1			
JCVIGM_014024_20110307		hypothetical protein																	
JCVIGM_018751_20110307		hypothetical protein																	
JCVIGM_020128_20110307		hypothetical protein										2	1						
JCVIGM_021326_20110307		hypothetical protein		9	5	4	7	7	5	4	1	8	6	1		2			
JCVIGM_021364_20110307		hypothetical protein																	
JCVIGM_021629_20110307		hypothetical protein																	
JCVIGM_022560_20110307		hypothetical protein		1	1	1		1			2								
JCVIGM_022561_20110307		hypothetical protein																	
JCVIGM_025687_20110307		hypothetical protein																	
JCVIGM_025940_20110307		hypothetical protein						2		1									

JCVIGM_026248_20110307		hypothetical protein																	
JCVIGM_026306_20110307		hypothetical protein		6	4	5	4	3	3	4	4	5	3	2					
JCVIGM_029504_20110307		hypothetical protein																	
JCVIGM_029868_20110307		hypothetical protein																	
JCVIGM_030581_20110307		hypothetical protein																	
JCVIGM_031963_20110307		hypothetical protein							1										
JCVIGM_036375_20110307		hypothetical protein		1	2	1	1			1									
JCVIGM_036678_20110307		hypothetical protein																	
JCVIGM_037187_20110307		hypothetical protein		1	1	2	1	1	1	2	2	1	1						
JCVIGM_039163_20110307		hypothetical protein		4	1	4	1	2	1	2	6	9	3						
JCVIGM_039195_20110307		hypothetical protein				2	1	1	1		3		2	1					
JCVIGM_042328_20110307		hypothetical protein																	
JCVIGM_043843_20110307		hypothetical protein																	
JCVIGM_044734_20110307		hypothetical protein									1					2			
JCVIGM_045412_20110307		hypothetical protein							1									1	
JCVIGM_047061_20110307		hypothetical protein																	
JCVIGM_048990_20110307		hypothetical protein																	
JCVIGM_052263_20110307		hypothetical protein																	
JCVIGM_054391_20110307		hypothetical protein																	
JCVIGM_055922_20110307		hypothetical protein																	
JCVIGM_056525_20110307		hypothetical protein																	
JCVIGM_057825_20110307		hypothetical protein					1		1			1							
JCVIGM_058381_20110307		hypothetical protein																	
JCVIGM_060468_20110307		hypothetical protein				1	1	1	1	1	1		1						
JCVIGM_060720_20110307		hypothetical protein		1			1												
JCVIGM_061313_20110307		hypothetical protein																	
JCVIGM_061338_20110307		hypothetical protein						3		1	1		1						
JCVIGM_063878_20110307		hypothetical protein																	
JCVIGM_065213_20110307		hypothetical protein																	

JCVIGM_068011_20110307		hypothetical protein							4	1		1							
JCVIGM_068020_20110307		hypothetical protein							1	2									
JCVIGM_070048_20110307		hypothetical protein																	
JCVIGM_070908_20110307		hypothetical protein		1			1	2			1	1	1						
JCVIGM_071303_20110307		hypothetical protein																	
JCVIGM_071361_20110307		hypothetical protein																	
JCVIGM_071586_20110307		hypothetical protein		1								2							
JCVIGM_071718_20110307		hypothetical protein																	
JCVIGM_072139_20110307		hypothetical protein				1	1		1										
JCVIGM_072463_20110307		hypothetical protein																	
JCVIGM_073483_20110307		hypothetical protein																	
JCVIGM_074339_20110307		hypothetical protein					1	1				1							
JCVIGM_074623_20110307		hypothetical protein																	
JCVIGM_075200_20110307		hypothetical protein		3			6	6	3			1							
JCVIGM_077653_20110307		hypothetical protein																	
JCVIGM_081664_20110307		hypothetical protein																	
JCVIGM_083533_20110307		hypothetical protein			1	1	1	2	2	3	1		1						
JCVIGM_084534_20110307		hypothetical protein																	
JCVIGM_084535_20110307		hypothetical protein			1								1						
JCVIGM_088087_20110307		hypothetical protein																	
JCVIGM_088594_20110307		hypothetical protein		1		1		1		1			2						
JCVIGM_090261_20110307		hypothetical protein																	
JCVIGM_090262_20110307		hypothetical protein								2		1							
JCVIGM_090263_20110307		hypothetical protein																	
JCVIGM_090646_20110307		hypothetical protein																	
JCVIGM_091802_20110307		hypothetical protein																	
JCVIGM_091850_20110307		hypothetical protein			1								2						
JCVIGM_092014_20110307		hypothetical protein																	
JCVIGM_092247_20110307		hypothetical protein																	

JCVIGM_092708_20110307		hypothetical protein				1		1				1							
JCVIGM_095744_20110307		hypothetical protein		2								2							
JCVIGM_097721_20110307		hypothetical protein																	
JCVIGM_098373_20110307		hypothetical protein																	
JCVIGM_101299_20110307		hypothetical protein																	
JCVIGM_101300_20110307	etfB	hypothetical protein				2													
JCVIGM_102281_20110307		hypothetical protein			3	2	1	3		2	6	2	2						
JCVIGM_102458_20110307		hypothetical protein		1		1	1	1	1	1			1	1					
JCVIGM_102522_20110307		hypothetical protein																	
JCVIGM_102523_20110307		hypothetical protein																	
JCVIGM_103433_20110307		hypothetical protein		1			1	1		2	1	1	2						
JCVIGM_104408_20110307		hypothetical protein						1		1			1						
JCVIGM_107850_20110307		hypothetical protein																	
JCVIGM_110415_20110307		hypothetical protein																	
JCVIGM_112258_20110307		hypothetical protein							3	1									
JCVIGM_113282_20110307		hypothetical protein																	
JCVIGM_115169_20110307		hypothetical protein																	
JCVIGM_115489_20110307		hypothetical protein																	
JCVIGM_116061_20110307		hypothetical protein																	
JCVIGM_116471_20110307		hypothetical protein																	
JCVIGM_118383_20110307		hypothetical protein		4				1	1	2		2	1						
JCVIGM_121048_20110307		hypothetical protein			1						1		2						
JCVIGM_121384_20110307		hypothetical protein																	
JCVIGM_121883_20110307		hypothetical protein						1					1						
JCVIGM_122516_20110307		hypothetical protein		1					1	1									
JCVIGM_125587_20110307		hypothetical protein																	
JCVIGM_126576_20110307		hypothetical protein																	
JCVIGM_128170_20110307		hypothetical protein																	
JCVIGM_128306_20110307		hypothetical protein		2		1		1	2										
JCVIGM_129017_20110307		hypothetical protein																	
JCVIGM_129660_20110307		hypothetical protein																	
JCVIGM_130216_20110307		hypothetical protein		2	1	1	2			1				1					
JCVIGM_132903_20110307		hypothetical protein		1			3	3	1	2	1								

JCVIGM_133803_20110307		hypothetical protein																	
JCVIGM_134883_20110307		hypothetical protein																	
JCVIGM_135963_20110307		hypothetical protein		1													1		
JCVIGM_141001_20110307		hypothetical protein									1					1			
JCVIGM_141853_20110307		hypothetical protein																	
JCVIGM_142860_20110307		hypothetical protein								1						1			
JCVIGM_144970_20110307		hypothetical protein									1								
JCVIGM_147926_20110307		hypothetical protein		3	2	3	3	1		3	2	3	2	3	1	6			
JCVIGM_148595_20110307		hypothetical protein			4		2	1	1	1		2	1						
JCVIGM_148602_20110307		hypothetical protein																	
JCVIGM_148969_20110307		hypothetical protein									1								
JCVIGM_150315_20110307		hypothetical protein				1					1								
JCVIGM_150334_20110307		hypothetical protein					1				1								
JCVIGM_150335_20110307		hypothetical protein		1		2				2	2	3	2			1			
JCVIGM_153727_20110307		hypothetical protein		1		1		1		2		2	2						
JCVIGM_155277_20110307		hypothetical protein																	
JCVIGM_156043_20110307		hypothetical protein			1								1						
JCVIGM_160972_20110307		hypothetical protein											1						
JCVIGM_161538_20110307		hypothetical protein																	
JCVIGM_161966_20110307		hypothetical protein																	
JCVIGM_163002_20110307		hypothetical protein								1									
JCVIGM_166279_20110307		hypothetical protein																	
JCVIGM_169083_20110307		hypothetical protein								1									
JCVIGM_170674_20110307		hypothetical protein								1		1							
JCVIGM_173056_20110307		hypothetical protein									1								
JCVIGM_173583_20110307		hypothetical protein																	
JCVIGM_175085_20110307		hypothetical protein																	
JCVIGM_175220_20110307		hypothetical protein																	
JCVIGM_176221_20110307		hypothetical protein						1		1									
JCVIGM_181018_20110307		hypothetical protein																	
JCVIGM_182077_20110307		hypothetical protein					1	1				2		2					
JCVIGM_185211_20110307		hypothetical protein							1		1								
JCVIGM_185931_20110307		hypothetical protein																	
JCVIGM_187544_20110307		hypothetical protein																	

JCVIGM_189376_20110307		hypothetical protein		1						1									
JCVIGM_189604_20110307		hypothetical protein																	
JCVIGM_190292_20110307		hypothetical protein																	
JCVIGM_190977_20110307		hypothetical protein																	
JCVIGM_191291_20110307		hypothetical protein						1				1							
JCVIGM_191293_20110307		hypothetical protein																	
JCVIGM_191827_20110307		hypothetical protein																	
JCVIGM_191888_20110307		hypothetical protein																	
JCVIGM_192065_20110307		hypothetical protein																	
JCVIGM_194742_20110307		hypothetical protein																	
JCVIGM_194897_20110307		hypothetical protein																	
JCVIGM_195386_20110307		hypothetical protein																	
JCVIGM_196029_20110307		hypothetical protein																	
JCVIGM_202561_20110307		hypothetical protein																	
JCVIGM_203237_20110307		hypothetical protein			1														
JCVIGM_204096_20110307		hypothetical protein																	
JCVIGM_205608_20110307		hypothetical protein																	
JCVIGM_207243_20110307		hypothetical protein																	
JCVIGM_209517_20110307		hypothetical protein					2	3	1			1	1						
JCVIGM_210696_20110307		hypothetical protein			2								1	1					
JCVIGM_210946_20110307		hypothetical protein		1		1		2		2	1	1	1						
JCVIGM_211394_20110307		hypothetical protein					1						1						
JCVIGM_212706_20110307		hypothetical protein																	
JCVIGM_213389_20110307		hypothetical protein					1	1		2	1	2	1						
JCVIGM_219434_20110307		hypothetical protein																	
JCVIGM_219781_20110307		hypothetical protein																	
JCVIGM_222747_20110307		hypothetical protein																	
JCVIGM_229339_20110307		hypothetical protein				1	2												
JCVIGM_234241_20110307		hypothetical protein																	
JCVIGM_237601_20110307		hypothetical protein																	
JCVIGM_239052_20110307		hypothetical protein																	
JCVIGM_245141_20110307		hypothetical protein					2			1		1	1	1					

JCVIGM_246343_20110307		hypothetical protein																	
JCVIGM_246344_20110307		hypothetical protein																	
JCVIGM_247614_20110307		hypothetical protein																	
JCVIGM_249782_20110307		hypothetical protein																	1
JCVIGM_251147_20110307		hypothetical protein																	
JCVIGM_251362_20110307		hypothetical protein		1				1				1							
JCVIGM_251712_20110307		hypothetical protein									2		1						
JCVIGM_256398_20110307		hypothetical protein																	
JCVIGM_259370_20110307		hypothetical protein																	
JCVIGM_262707_20110307		hypothetical protein																	
JCVIGM_265819_20110307	etfB	hypothetical protein																	
JCVIGM_268646_20110307		hypothetical protein		1				1											
JCVIGM_274621_20110307		hypothetical protein															1		
JCVIGM_277806_20110307		hypothetical protein					1							1					
JCVIGM_281111_20110307		hypothetical protein																	
JCVIGM_281956_20110307		hypothetical protein																	
JCVIGM_282080_20110307		hypothetical protein				2		1			1								
JCVIGM_283350_20110307		hypothetical protein																	
JCVIGM_286196_20110307		hypothetical protein											1						
JCVIGM_286605_20110307		hypothetical protein		2	4	1	3	1				4	1	1					
JCVIGM_288269_20110307		hypothetical protein		1	3	1		1				1		1		1			
JCVIGM_288716_20110307		hypothetical protein				1				2	1								
JCVIGM_291424_20110307		hypothetical protein							1										
JCVIGM_295105_20110307		hypothetical protein																	
JCVIGM_295523_20110307		hypothetical protein																	
JCVIGM_296216_20110307		hypothetical protein						1											
JCVIGM_298432_20110307		hypothetical protein																	
JCVIGM_298496_20110307		hypothetical protein																	
JCVIGM_301982_20110307		hypothetical protein																	

JCVIGM_302456_20110307		hypothetical protein					2	2		1	1	1							
JCVIGM_303429_20110307		hypothetical protein																	
JCVIGM_303566_20110307		hypothetical protein													2				
JCVIGM_303609_20110307		hypothetical protein																	
JCVIGM_303798_20110307		hypothetical protein				4		1		1	3	1							
JCVIGM_306883_20110307		hypothetical protein																	
JCVIGM_306884_20110307		hypothetical protein																	
JCVIGM_307057_20110307		hypothetical protein																	
JCVIGM_307436_20110307		hypothetical protein																	
JCVIGM_307765_20110307		hypothetical protein																	
JCVIGM_309808_20110307		hypothetical protein					1	1				1	2	1					
JCVIGM_310529_20110307		hypothetical protein																	
JCVIGM_313684_20110307		hypothetical protein																	
JCVIGM_314099_20110307		hypothetical protein																	
JCVIGM_314549_20110307		hypothetical protein		1		1	2	3		1	1	1	1						
JCVIGM_319934_20110307		hypothetical protein																	
JCVIGM_322352_20110307		hypothetical protein																	
JCVIGM_323810_20110307		hypothetical protein																	
JCVIGM_327403_20110307		hypothetical protein									1	1							
JCVIGM_328614_20110307		hypothetical protein																	
JCVIGM_332056_20110307		hypothetical protein																	
JCVIGM_332688_20110307		hypothetical protein																	
JCVIGM_333745_20110307		hypothetical protein			1	1	1	1	2	1		1	3	2					
JCVIGM_336004_20110307		hypothetical protein				2													
JCVIGM_336057_20110307		hypothetical protein																	
JCVIGM_340014_20110307		hypothetical protein			2		1	1		1	1	2	1	1					
JCVIGM_340609_20110307		hypothetical protein		2	4	1		1		1	2	3	4	2					
JCVIGM_341200_20110307		hypothetical protein					1			4	2	4							

JCVIGM_344661_20110307		hypothetical protein																	
JCVIGM_345968_20110307		hypothetical protein																	
JCVIGM_348560_20110307		hypothetical protein																	
JCVIGM_351456_20110307		hypothetical protein																	
JCVIGM_359900_20110307		hypothetical protein																	
JCVIGM_362686_20110307		hypothetical protein				1		1				1	1						
JCVIGM_364085_20110307		hypothetical protein					2			1	1	1							
JCVIGM_365092_20110307		hypothetical protein																	
JCVIGM_367321_20110307		hypothetical protein																	
JCVIGM_367720_20110307		hypothetical protein		2			1	2											
JCVIGM_368650_20110307		hypothetical protein																	
JCVIGM_369484_20110307		hypothetical protein				1	1	1											
JCVIGM_371571_20110307		hypothetical protein																	
JCVIGM_372380_20110307		hypothetical protein																	
JCVIGM_373222_20110307		hypothetical protein																	
JCVIGM_373315_20110307		hypothetical protein		1									1						
JCVIGM_375019_20110307		hypothetical protein																	
JCVIGM_379110_20110307		hypothetical protein																	
JCVIGM_380771_20110307		hypothetical protein																	
JCVIGM_387933_20110307		hypothetical protein																	
JCVIGM_390199_20110307		hypothetical protein																	
JCVIGM_390242_20110307		hypothetical protein																	
JCVIGM_390806_20110307		hypothetical protein																	
JCVIGM_391550_20110307		hypothetical protein																	
JCVIGM_393860_20110307		hypothetical protein											1						
JCVIGM_398055_20110307		hypothetical protein																	
JCVIGM_398168_20110307		hypothetical protein																	
JCVIGM_399462_20110307		hypothetical protein					1	1											
JCVIGM_400027_20110307		hypothetical protein			2	2					1	1	1	2					

JCVIGM_400278_20110307		hypothetical protein																	
JCVIGM_400950_20110307		hypothetical protein																	
JCVIGM_405787_20110307		hypothetical protein									1	1							
JCVIGM_410986_20110307		hypothetical protein											2						
JCVIGM_414552_20110307		hypothetical protein					1												
JCVIGM_418955_20110307		hypothetical protein																	
JCVIGM_420396_20110307		hypothetical protein																	
JCVIGM_420773_20110307		hypothetical protein																	
JCVIGM_424310_20110307		hypothetical protein		1		1						1							
JCVIGM_434782_20110307		hypothetical protein																	
JCVIGM_441424_20110307		hypothetical protein																	
JCVIGM_444308_20110307		hypothetical protein																	
JCVIGM_444309_20110307		hypothetical protein																	
JCVIGM_055829_20110307		hypothetical protein BACCOPRO_02435											1						
JCVIGM_158091_20110307		hypothetical protein BACCOPRO_03643					1	1	1	1		1							
JCVIGM_068437_20110307		hypothetical protein CLOST_1033																	
JCVIGM_442772_20110307		hypothetical protein ELI_0537				1													
JCVIGM_010811_20110307		inosine guanosine and xanthosine phosphorylase family					1		2										
JCVIGM_403964_20110307		inosine guanosine and xanthosine phosphorylase family protein																	
JCVIGM_054853_20110307	guaB	inosine-5'-monophosphate dehydrogenase																	
JCVIGM_092237_20110307		inosine-5'-monophosphate dehydrogenase																	
JCVIGM_126259_20110307	guaB	inosine-5'-monophosphate dehydrogenase					2												
JCVIGM_196447_20110307		inosine-5'-monophosphate dehydrogenase																	
JCVIGM_255764_20110307		inosine-5'-monophosphate dehydrogenase																	
JCVIGM_272653_20110307		inosine-5'-monophosphate dehydrogenase																	
JCVIGM_328476_20110307		inosine-5'-monophosphate dehydrogenase																	
JCVIGM_357305_20110307	iolG	inositol 2-dehydrogenase																	
JCVIGM_069994_20110307		inositol-3-phosphate synthase																	

JCVIGM_091917_20110307		inositol-3-phosphate synthase																	
JCVIGM_184954_20110307		integral membrane sensor signal transduction histidine kinase													1		1		
JCVIGM_065899_20110307		internalin-related protein					1	1	1	3			1						
JCVIGM_120727_20110307	ilvC	ketol-acid reductoisomerase																	
JCVIGM_156339_20110307	ilvC	ketol-acid reductoisomerase				1													
JCVIGM_185462_20110307	ilvC	ketol-acid reductoisomerase					1	1											
JCVIGM_251867_20110307	ilvC	ketol-acid reductoisomerase																	
JCVIGM_287230_20110307	ilvC	ketol-acid reductoisomerase																	
JCVIGM_322248_20110307	ilvC	ketol-acid reductoisomerase																	
JCVIGM_062252_20110307		ketose-bisphosphate aldolase, class II						1		1	1								
JCVIGM_128542_20110307		ketose-bisphosphate aldolase, class II					1	3		3									
JCVIGM_260030_20110307		KHG/KDPG family aldolase/carbohydrate kinase, pfkB family																	
JCVIGM_418431_20110307		kinase, pfkB family																	
JCVIGM_025581_20110307	araA	L-arabinose isomerase				1				1						1			
JCVIGM_046489_20110307	araA	L-arabinose isomerase																	
JCVIGM_060257_20110307	araA	L-arabinose isomerase				7		1											
JCVIGM_073944_20110307	araA	L-arabinose isomerase								3	1	2							
JCVIGM_074178_20110307	araA	L-arabinose isomerase								1	1		1						
JCVIGM_086747_20110307	araA	L-arabinose isomerase					3	1		1	1	3							
JCVIGM_088705_20110307	araA	L-arabinose isomerase							1			1							
JCVIGM_168776_20110307	araA	L-arabinose isomerase								1									
JCVIGM_206950_20110307	araA1	L-arabinose isomerase 1																	
JCVIGM_102423_20110307		L-asparaginase																	
JCVIGM_051778_20110307		L-asparaginase, type II																	
JCVIGM_177219_20110307		L-fucose isomerase and related proteins																	
JCVIGM_022476_20110307	fucI	L-fucose isomerase, C-terminal domain																	
JCVIGM_059070_20110307		L-fucose isomerase, C-terminal domain		1															
JCVIGM_066045_20110307		L-fucose isomerase, C-terminal domain																	
JCVIGM_101032_20110307		L-fucose isomerase, C-terminal domain		1								1							
JCVIGM_106686_20110307		L-fucose isomerase, C-terminal domain			1		1			1			2						

JCVIGM_124735_20110307		L-fucose isomerase, C-terminal domain																	
JCVIGM_187352_20110307		L-fucose isomerase, C-terminal domain																	
JCVIGM_278994_20110307		L-fucose isomerase, C-terminal domain																	
JCVIGM_325641_20110307		L-fucose isomerase-like protein																	
JCVIGM_171365_20110307		lipoprotein																	
JCVIGM_083497_20110307	ldh	L-lactate dehydrogenase								1									
JCVIGM_159477_20110307	dapL	LL-diaminopimelate aminotransferase																	
JCVIGM_130368_20110307		L-ribulose-5-phosphate 4-epimerase																	
JCVIGM_060255_20110307	araD	L-ribulose-5-phosphate 4-epimerase araD								1		1							
JCVIGM_160693_20110307	araD	L-ribulose-5-phosphate 4-epimerase araD																	
JCVIGM_295794_20110307	araD	L-ribulose-5-phosphate 4-epimerase araD																	
JCVIGM_000243_20110307	lysS	lysine--tRNA ligase																	
JCVIGM_161438_20110307		M18 family aminopeptidase																	
JCVIGM_203664_20110307		M18 family aminopeptidase																	
JCVIGM_229762_20110307		major outer membrane lipoprotein 1												1	9	2	6	9	2
JCVIGM_006253_20110307	mdh	malate dehydrogenase																	
JCVIGM_012327_20110307	mdh	malate dehydrogenase																	
JCVIGM_017662_20110307		malate dehydrogenase								1		1							
JCVIGM_026640_20110307	mdh	malate dehydrogenase				1	1	1											
JCVIGM_028360_20110307	mdh	malate dehydrogenase					3	2	1	1		2							
JCVIGM_127124_20110307	mdh	malate dehydrogenase		1			1			1		1							
JCVIGM_229225_20110307	mdh	malate dehydrogenase																1	
JCVIGM_079110_20110307		malate dehydrogenase (oxaloacetate-decarboxylating)																	
JCVIGM_020629_20110307	mdh	malate dehydrogenase, NAD-dependent					2						1						
JCVIGM_007627_20110307		maltose-binding protein																	
JCVIGM_201419_20110307		maltose-binding protein					1												
JCVIGM_173530_20110307		membrane protein, bmp family		1			1	1	1										
JCVIGM_186148_20110307		membrane protein, bmp family					1	1	1	2			2						
JCVIGM_052677_20110307		metallo-beta-lactamase superfamily																	
JCVIGM_038699_20110307	metK	methionine adenosyltransferase																	

JCVIGM_046971_20110307	metK	methionine adenosyltransferase					2												
JCVIGM_076017_20110307	metK	methionine adenosyltransferase																	
JCVIGM_211427_20110307		methylmalonate-semialdehyde dehydrogenase (acylating)		2															
JCVIGM_014933_20110307	mmdA	methylmalonyl-coA decarboxylase										1	1						
JCVIGM_034121_20110307		methylmalonyl-coA mutase large subunit																	
JCVIGM_255025_20110307		methylmalonyl-coA mutase large subunit																	
JCVIGM_082989_20110307		methylmalonyl-coA mutase N-terminal domain																	
JCVIGM_069236_20110307	mutA	methylmalonyl-coA mutase, small subunit							1										
JCVIGM_033013_20110307		microcompartments protein																	
JCVIGM_083041_20110307		microcompartments protein						1											
JCVIGM_188817_20110307		monosaccharide ABC transporter substrate-binding protein, CUT2 family																	
JCVIGM_039831_20110307		monosaccharide ABC transporter substrate-binding protein, CUT2 family			1	1	3	2	3	1	1					1			
JCVIGM_192144_20110307		monosaccharide ABC transporter substrate-binding protein, CUT2 family		2	2	1	2	2			1	3	2						
JCVIGM_070547_20110307	purE	N5-carboxyaminoimidazole ribonucleotide mutase		1			1		1	1									
JCVIGM_060713_20110307		N-acetylneuraminate lyase							1										
JCVIGM_233593_20110307	nuoI	NADH-quinone oxidoreductase subunit I											1						
JCVIGM_057221_20110307		NADP-dependent malic enzyme		2			3	1	2										
JCVIGM_231459_20110307		NADPH-dependent butanol dehydrogenase																	
JCVIGM_121448_20110307		nifU-related domain containing protein																	
JCVIGM_028245_20110307		nitrate/sulfonate/bicarbonate ABC transporter, periplasmic nitrate/sulfonate/bicarbonate-binding protein																	
JCVIGM_149307_20110307		nitrite/sulfite reductase, 4Fe-4S iron-sulfur cluster-binding domain protein																	
JCVIGM_243460_20110307		nitrogen regulation protein																	1
JCVIGM_074550_20110307		nitroreductase family																	
JCVIGM_142709_20110307		oligopeptide-binding protein OppA																	
JCVIGM_287642_20110307		oligopeptide-binding protein OppA																	
JCVIGM_291730_20110307		oligopeptide-binding protein OppA																	
JCVIGM_320377_20110307		oligopeptide-binding protein OppA																	
JCVIGM_030295_20110307		ompA family protein																	
JCVIGM_021098_20110307	pyrF	orotidine 5'-phosphate decarboxylase																	

JCVIGM_069275_20110307		outer membrane protein																	
JCVIGM_021757_20110307		oxaloacetate decarboxylase, alpha subunit																	
JCVIGM_185159_20110307		oxidoreductase domain protein																	
JCVIGM_227464_20110307		oxidoreductase domain protein																	
JCVIGM_292816_20110307		oxidoreductase domain protein																	
JCVIGM_098050_20110307		peptide/nickel ABC transporter substrate-binding protein		2		2	1	2											
JCVIGM_051204_20110307		peptidyl-prolyl cis-trans isomerase																	
JCVIGM_077390_20110307		peptidyl-prolyl cis-trans isomerase							2	1									
JCVIGM_089246_20110307		peptidyl-prolyl cis-trans isomerase					1		1										
JCVIGM_359878_20110307		periplasmic binding protein/lacI transcriptional regulator																	
JCVIGM_330291_20110307		periplasmic protein																	
JCVIGM_352406_20110307		periplasmic protein					2			1		1	1						
JCVIGM_063156_20110307	pta	phosphate acetyltransferase																	
JCVIGM_141522_20110307	pta	phosphate acetyltransferase																	
JCVIGM_008179_20110307	pckA	phosphoenolpyruvate carboxykinase (ATP)		1		3	2	2	2	3	2	2							
JCVIGM_042402_20110307	pckA	phosphoenolpyruvate carboxykinase (ATP)																	
JCVIGM_051034_20110307	pckA	phosphoenolpyruvate carboxykinase (ATP)									1								
JCVIGM_066626_20110307	pckA	phosphoenolpyruvate carboxykinase (ATP)				1	2	1	5	4	1								
JCVIGM_070718_20110307	pckA	phosphoenolpyruvate carboxykinase (ATP)		7	3	5	12	7	7	6		4	3	2					
JCVIGM_084152_20110307	pckA	phosphoenolpyruvate carboxykinase (ATP)																	
JCVIGM_094757_20110307	pckA	phosphoenolpyruvate carboxykinase (ATP)																	
JCVIGM_108298_20110307	pckA	phosphoenolpyruvate carboxykinase (ATP)		1		2		1			2	1	1						
JCVIGM_111820_20110307	pckA	phosphoenolpyruvate carboxykinase (ATP)		1		1	2	2	2	2	1	3			2				
JCVIGM_116732_20110307		phosphoenolpyruvate carboxykinase (ATP)								1									
JCVIGM_126450_20110307	pckA	phosphoenolpyruvate carboxykinase (ATP)																	
JCVIGM_145745_20110307	pckA	phosphoenolpyruvate carboxykinase (ATP)						1											
JCVIGM_157605_20110307	pckA	phosphoenolpyruvate carboxykinase (ATP)						2		3									
JCVIGM_168405_20110307	pckA	phosphoenolpyruvate carboxykinase (ATP)																	
JCVIGM_170518_20110307	pckG	phosphoenolpyruvate carboxykinase (ATP)																	
JCVIGM_186619_20110307	pckA	phosphoenolpyruvate carboxykinase (ATP)																	
JCVIGM_188966_20110307	pckA	phosphoenolpyruvate carboxykinase (ATP)			1	1	1	2		3		2							

JCVIGM_200200_20110307	pckA	phosphoenolpyruvate carboxykinase (ATP)		1	1		1				1								
JCVIGM_212975_20110307		phosphoenolpyruvate carboxykinase (ATP)		3	2	1	3	2	1	2	1	4	3	2					
JCVIGM_214913_20110307	pckA	phosphoenolpyruvate carboxykinase (ATP)																	
JCVIGM_219457_20110307	pckA	phosphoenolpyruvate carboxykinase (ATP)																	
JCVIGM_255519_20110307	pckA	phosphoenolpyruvate carboxykinase (ATP)																	
JCVIGM_257324_20110307	pckA	phosphoenolpyruvate carboxykinase (ATP)		1		1	2				2	2	2						
JCVIGM_259940_20110307	pckG	phosphoenolpyruvate carboxykinase (ATP)																	
JCVIGM_297711_20110307	pckA	phosphoenolpyruvate carboxykinase (ATP)		1								1							
JCVIGM_315164_20110307	pckA	phosphoenolpyruvate carboxykinase (ATP)		1			1			1		1							
JCVIGM_315431_20110307	pckA	phosphoenolpyruvate carboxykinase (ATP)		2	3	1	1	2		1	1	3	3						
JCVIGM_423536_20110307		phosphoenolpyruvate carboxykinase (ATP)																	
JCVIGM_070299_20110307	pckA	phosphoenolpyruvate carboxykinase [ATP]		2	1	1	3	2	4	2	2	3	3	1	2				
JCVIGM_127286_20110307	pckA	phosphoenolpyruvate carboxykinase [ATP]																	
JCVIGM_216386_20110307	manL	phosphoenolpyruvate-dependent sugar phosphotransferase system EIIAB, probably mannose specific																	
JCVIGM_007589_20110307	pgk	phosphoglycerate kinase																	
JCVIGM_015380_20110307	pgk	phosphoglycerate kinase																	
JCVIGM_027320_20110307	pgk	phosphoglycerate kinase		1		3	1	2		2	2	1	1						
JCVIGM_061465_20110307	pgk	phosphoglycerate kinase																	
JCVIGM_079319_20110307	pgk	phosphoglycerate kinase																	
JCVIGM_095819_20110307	pgk	phosphoglycerate kinase									1								
JCVIGM_113192_20110307	pgk	phosphoglycerate kinase			1	1	1												
JCVIGM_122299_20110307	pgk	phosphoglycerate kinase																	
JCVIGM_122514_20110307	pgk	phosphoglycerate kinase				1				1			1						
JCVIGM_152568_20110307	pgk	phosphoglycerate kinase																	
JCVIGM_161540_20110307	pgk	phosphoglycerate kinase				1					2								
JCVIGM_195337_20110307	pgk	phosphoglycerate kinase																	
JCVIGM_234057_20110307	pgk	phosphoglycerate kinase																	
JCVIGM_239710_20110307	pgk	phosphoglycerate kinase																	
JCVIGM_240738_20110307	pgk	phosphoglycerate kinase		1	3		1				2								
JCVIGM_249205_20110307	pgk	phosphoglycerate kinase																	
JCVIGM_260432_20110307	pgk	phosphoglycerate kinase													1			1	

JCVIGM_260954_20110307	pgk	phosphoglycerate kinase																1	
JCVIGM_313871_20110307	pgk	phosphoglycerate kinase																	
JCVIGM_325494_20110307	pgk	phosphoglycerate kinase																	
JCVIGM_329407_20110307	pgk	phosphoglycerate kinase		2	1	1		2		1		2		1					
JCVIGM_040605_20110307	eno	phosphopyruvate hydratase																	
JCVIGM_042947_20110307	eno	phosphopyruvate hydratase					1		1										
JCVIGM_045954_20110307	eno	phosphopyruvate hydratase					1					1							
JCVIGM_064103_20110307	eno	phosphopyruvate hydratase					2												
JCVIGM_069773_20110307	eno	phosphopyruvate hydratase																	
JCVIGM_117723_20110307	eno	phosphopyruvate hydratase				1	1		2	1									
JCVIGM_189834_20110307	eno	phosphopyruvate hydratase							1	1			1						
JCVIGM_223379_20110307	eno	phosphopyruvate hydratase																	
JCVIGM_226543_20110307	eno	phosphopyruvate hydratase								1		1							
JCVIGM_231374_20110307	eno	phosphopyruvate hydratase						1	1										
JCVIGM_234175_20110307	eno	phosphopyruvate hydratase					1		1										
JCVIGM_015906_20110307	purD	phosphoribosylamine--glycine ligase					1		2	1									
JCVIGM_074211_20110307	purD	phosphoribosylamine--glycine ligase					1			1	1								
JCVIGM_058619_20110307	purH	phosphoribosylaminoimidazolecarboxamide formyltransferase/IMP cyclohydrolase																	
JCVIGM_089519_20110307	glgP1	phosphorylase																	
JCVIGM_091853_20110307	glgP1	phosphorylase																	
JCVIGM_250822_20110307		phosphorylase																	
JCVIGM_020564_20110307	glgP	phosphorylase, glycogen/starch/alpha-glucan family																	
JCVIGM_132125_20110307	serC	phosphoserine transaminase									2	1							
JCVIGM_151788_20110307	serC	phosphoserine transaminase								1									
JCVIGM_254038_20110307	serC	phosphoserine transaminase																	
JCVIGM_000744_20110307	pnp	polyribonucleotide nucleotidyltransferase																	
JCVIGM_049618_20110307	pnp	polyribonucleotide nucleotidyltransferase																	
JCVIGM_156816_20110307	pnp	polyribonucleotide nucleotidyltransferase																	
JCVIGM_177288_20110307	pnp	polyribonucleotide nucleotidyltransferase																	
JCVIGM_284176_20110307	pnp	polyribonucleotide nucleotidyltransferase																	

JCVIGM_301236_20110307	pnp	polyribonucleotide nucleotidyltransferase																	
JCVIGM_384292_20110307	pnp	polyribonucleotide nucleotidyltransferase																	
JCVIGM_415810_20110307	pnp	polyribonucleotide nucleotidyltransferase		1															
JCVIGM_149742_20110307		possible dehydrogenase																	
JCVIGM_158818_20110307		predicted glycosylase																	
JCVIGM_094962_20110307	araN	probable arabinose-binding protein					2												
JCVIGM_443198_20110307	gcvPA	probable glycine dehydrogenase [decarboxylating] subunit 1																	
JCVIGM_181243_20110307	tal	probable transaldolase																	
JCVIGM_217573_20110307	tal	probable transaldolase																	
JCVIGM_305907_20110307	tal	probable transaldolase																	
JCVIGM_106798_20110307		propanediol utilization protein pduL																	
JCVIGM_343769_20110307	pduD	propanediol utilization: dehydratase pduD																	
JCVIGM_030557_20110307		propionyl-coA carboxylase beta chain							1				1						
JCVIGM_070982_20110307		propionyl-coA carboxylase subunit beta																	
JCVIGM_076862_20110307		propionyl-coA carboxylase subunit beta					1												
JCVIGM_212940_20110307		propionyl-coA carboxylase subunit beta																	
JCVIGM_244599_20110307	deoD	purine nucleoside phosphorylase deoD-type																	
JCVIGM_014093_20110307		purine nucleoside phosphorylase I, inosine and guanosine-specific							1										
JCVIGM_019193_20110307		purine nucleoside phosphorylase I, inosine and guanosine-specific																	
JCVIGM_033253_20110307		putative 6-phosphofructokinase																	
JCVIGM_128651_20110307		putative 6-phosphofructokinase																	
JCVIGM_176874_20110307		putative 6-phosphofructokinase																	
JCVIGM_149395_20110307		putative bacterial extracellular solute-binding protein																	
JCVIGM_215021_20110307		putative bacterial extracellular solute-binding protein																	
JCVIGM_268677_20110307		putative bacterial extracellular solute-binding protein				1													
JCVIGM_273647_20110307		putative bacterial extracellular solute-binding protein					1	2											
JCVIGM_286660_20110307		putative bacterial extracellular solute-binding protein						1				1							
JCVIGM_297731_20110307		putative bacterial extracellular solute-binding protein																	
JCVIGM_417971_20110307		putative bacterial extracellular solute-binding protein																	
JCVIGM_237233_20110307	ytfQ	putative D-ribose transport system periplasmic binding component																	

JCVIGM_192868_20110307		putative endoribonuclease L-PSP					1												
JCVIGM_028951_20110307		putative esterase																	
JCVIGM_101895_20110307		putative fumarate reductase flavoprotein subunit																	
JCVIGM_010624_20110307		putative immunoreactive antigen PG33																	
JCVIGM_182718_20110307		putative IMP cyclohydrolase		3			1												
JCVIGM_071025_20110307		putative inositol-3-phosphate synthase					1		1										
JCVIGM_106169_20110307		putative inositol-3-phosphate synthase																	
JCVIGM_240722_20110307		putative inositol-3-phosphate synthase				1		1					1						
JCVIGM_063721_20110307		putative lipoprotein																	
JCVIGM_094735_20110307		putative lipoprotein																	
JCVIGM_105771_20110307		putative major outer membrane protein											1						
JCVIGM_070279_20110307	spoVG	putative septation protein spoVG								1									
JCVIGM_178430_20110307		putative sugar ABC transporter, sugar-binding protein		1		1													
JCVIGM_194741_20110307		putative uncharacterized protein (fragment)					1	2											
JCVIGM_000105_20110307	ppdK	pyruvate, phosphate dikinase																	
JCVIGM_013898_20110307	ppdK	pyruvate, phosphate dikinase																	
JCVIGM_026505_20110307	ppdK	pyruvate, phosphate dikinase		2			1		1										
JCVIGM_036153_20110307	ppdK	pyruvate, phosphate dikinase					1	1		1									
JCVIGM_066682_20110307	ppdK	pyruvate, phosphate dikinase																	
JCVIGM_109792_20110307	ppdK	pyruvate, phosphate dikinase		1		1	2	3	1	1	2	1							
JCVIGM_135990_20110307	ppdK	pyruvate, phosphate dikinase																	
JCVIGM_173329_20110307		pyruvate, phosphate dikinase																	
JCVIGM_213012_20110307	ppdK	pyruvate, phosphate dikinase																	
JCVIGM_213014_20110307	ppdK	pyruvate, phosphate dikinase																	
JCVIGM_277616_20110307		pyruvate, phosphate dikinase		1			1				1	1							
JCVIGM_335683_20110307		pyruvate/oxaloacetate carboxyltransferase																	
JCVIGM_198898_20110307		pyruvate-formate lyase																	
JCVIGM_255194_20110307		regulator of chromosome condensation, RCC1 (fragment)																	
JCVIGM_036971_20110307	rplJ	ribosomal protein L10																	
JCVIGM_042383_20110307	rplJ	ribosomal protein L10																	
JCVIGM_071597_20110307	rplJ	ribosomal protein L10						1											
JCVIGM_092732_20110307	rplJ	ribosomal protein L10																	

JCVIGM_131224_20110307	rplJ	ribosomal protein L10														2			
JCVIGM_210248_20110307	rplJ	ribosomal protein L10														1			
JCVIGM_226856_20110307	rplJ	ribosomal protein L10													1			1	
JCVIGM_082097_20110307	rplK	ribosomal protein L11																	
JCVIGM_084768_20110307	rplK	ribosomal protein L11				1													
JCVIGM_136348_20110307	rplK	ribosomal protein L11																	
JCVIGM_257392_20110307	rplK	ribosomal protein L11			2														
JCVIGM_077076_20110307	rplK	ribosomal protein L11, N-terminal domain		2	1	7	3	5	1	3	3	3	2			1			
JCVIGM_097373_20110307	rplK	ribosomal protein L11, N-terminal domain					1	1		1									
JCVIGM_045961_20110307	rplB	ribosomal protein L2																	
JCVIGM_119829_20110307	rplB	ribosomal protein L2										1							
JCVIGM_193483_20110307	rplB	ribosomal protein L2																	
JCVIGM_159582_20110307	rplW	ribosomal protein L23																	
JCVIGM_006060_20110307	rplL	ribosomal protein L7/L12																	
JCVIGM_054869_20110307	rplL	ribosomal protein L7/L12																	
JCVIGM_156392_20110307	rplL	ribosomal protein L7/L12																	
JCVIGM_159116_20110307	rplL	ribosomal protein L7/L12		1		3	3	1	8	2		1	1						
JCVIGM_163260_20110307	rplL	ribosomal protein L7/L12								1									
JCVIGM_327331_20110307	rplL	ribosomal protein L7/L12																	
JCVIGM_274754_20110307	rpsJ	ribosomal protein S10																	
JCVIGM_021052_20110307	rpsG	ribosomal protein S7																	
JCVIGM_089527_20110307	rpsH	ribosomal protein S8						1											
JCVIGM_106685_20110307	rpsH	ribosomal protein S8					1	1				1							
JCVIGM_116015_20110307	rpsH	ribosomal protein S8																	
JCVIGM_158733_20110307	rpsH	ribosomal protein S8																	
JCVIGM_202567_20110307	rpsH	ribosomal protein S8																	
JCVIGM_216187_20110307	rpsH	ribosomal protein S8																	
JCVIGM_114301_20110307	rpsI	ribosomal protein S9/S16																	
JCVIGM_393651_20110307	rpoD	RNA polymerase sigma factor												1	1			1	
JCVIGM_229570_20110307		RNA polymerase sigma factor, sigma-70 family																	
JCVIGM_007913_20110307		rubredoxin				2					1					1			

JCVIGM_057213_20110307		rubredoxin		2	4	1	1	5	3	1	6	2	2						
JCVIGM_006262_20110307		rubredoxin/rubrerythrin																	
JCVIGM_020560_20110307		rubredoxin/rubrerythrin		13	8	6	9	11	3	6	8	5	4	7		4			
JCVIGM_060719_20110307		rubredoxin/rubrerythrin		1		1	11	11	2	10		1	2	1	1	1			
JCVIGM_085243_20110307		rubredoxin/rubrerythrin		1			1	1		1	3	1	1	2		1			
JCVIGM_248144_20110307		rubredoxin/rubrerythrin																	
JCVIGM_401158_20110307		rubredoxin/rubrerythrin					1	1	1	1									
JCVIGM_415862_20110307		rubredoxin/rubrerythrin																	
JCVIGM_054682_20110307	rbr	rubrerythrin					2												
JCVIGM_073521_20110307		rubrerythrin							1										
JCVIGM_091745_20110307		rubrerythrin																	
JCVIGM_119026_20110307		rubrerythrin				1	1	1		1	1								
JCVIGM_127859_20110307		rubrerythrin																	
JCVIGM_283228_20110307		rubrerythrin																	
JCVIGM_027483_20110307	metK	S-adenosylmethionine synthetase, N-terminal domain																	
JCVIGM_031546_20110307		seryl-tRNA synthetase N-terminal domain																	
JCVIGM_093445_20110307		short-chain dehydrogenase/reductase SDR																	
JCVIGM_318607_20110307	ssb	single-strand binding protein																	
JCVIGM_437080_20110307		single-stranded DNA-binding protein																	
JCVIGM_166571_20110307		smr domain protein																	
JCVIGM_117941_20110307	luxS	S-ribosylhomocysteine lyase																	
JCVIGM_158998_20110307		SSU ribosomal protein S1P																	
JCVIGM_034245_20110307		subtilase family																	
JCVIGM_125149_20110307		subtilase family domain protein																	
JCVIGM_007432_20110307		succinate CoA transferase																	
JCVIGM_079148_20110307		sugar ABC transporter substrate-binding protein																	
JCVIGM_091077_20110307		sugar ABC transporter substrate-binding protein						1											
JCVIGM_166102_20110307		sugar ABC transporter substrate-binding protein																	
JCVIGM_291360_20110307		sugar ABC transporter substrate-binding protein																	
JCVIGM_400559_20110307		sugar ABC transporter substrate-binding protein																	
JCVIGM_412377_20110307		sugar ABC transporter, periplasmic sugar-binding protein																	

JCVIGM_371869_20110307		sugar ABC transporter, sugar-binding protein																	
JCVIGM_014671_20110307		sugar phosphate isomerases/epimerases				1													
JCVIGM_084352_20110307		superoxide dismutase					1		1		1								
JCVIGM_070610_20110307		susD family							1										
JCVIGM_102265_20110307		susD family			3							1	2	1					
JCVIGM_131087_20110307		tetratricopeptide repeat protein																	
JCVIGM_391275_20110307	thlA3	thlA3			1														
JCVIGM_070609_20110307		tonB dependent receptor																	
JCVIGM_081124_20110307		tonB-dependent receptor plug domain protein																	
JCVIGM_134744_20110307		tonB-dependent receptor plug domain protein											1						
JCVIGM_052711_20110307		tonB-linked outer membrane protein, susC/ragA family			2							1	3	1					
JCVIGM_010680_20110307	fusA	translation elongation factor G									1								
JCVIGM_023291_20110307	fusA	translation elongation factor G								1									
JCVIGM_078720_20110307	fusA	translation elongation factor G																	
JCVIGM_105810_20110307	fusA	translation elongation factor G																	
JCVIGM_112572_20110307	fusA	translation elongation factor G																	
JCVIGM_143844_20110307	fusA	translation elongation factor G			1					1		1	1						
JCVIGM_097131_20110307	tsf	translation elongation factor Ts									1								
JCVIGM_183571_20110307	tsf	translation elongation factor Ts					2	1	1	2									
JCVIGM_277838_20110307	tsf	translation elongation factor Ts																	
JCVIGM_431442_20110307	tsf	translation elongation factor Ts																	
JCVIGM_026960_20110307	tuf	translation elongation factor Tu																	
JCVIGM_029795_20110307	tuf	translation elongation factor Tu																	
JCVIGM_109601_20110307	tuf	translation elongation factor Tu						1											
JCVIGM_124820_20110307	tuf	translation elongation factor Tu																	
JCVIGM_157947_20110307	tuf	translation elongation factor Tu			4														
JCVIGM_166526_20110307	tufB	translation elongation factor Tu												1	2				
JCVIGM_185454_20110307	tuf	translation elongation factor Tu																	
JCVIGM_196046_20110307	tuf	translation elongation factor Tu																	
JCVIGM_196200_20110307	tuf	translation elongation factor Tu																	
JCVIGM_205201_20110307	tuf	translation elongation factor Tu																	
JCVIGM_211136_20110307	tuf	translation elongation factor Tu																	

JCVIGM_237573_20110307	tuf	translation elongation factor Tu																	
JCVIGM_249773_20110307	tuf	translation elongation factor Tu																	
JCVIGM_250936_20110307	tufB	translation elongation factor Tu																1	
JCVIGM_300774_20110307	tuf	translation elongation factor Tu																	
JCVIGM_368873_20110307	tuf	translation elongation factor Tu																	
JCVIGM_385355_20110307		transposase, IS111A/IS1328/IS1533																	
JCVIGM_364669_20110307		TRAP transporter solute receptor, TAXI family							2										
JCVIGM_001774_20110307	tpiA	triose-phosphate isomerase					1			1								1	
JCVIGM_008332_20110307	tpiA	triose-phosphate isomerase		2	3	1	2	2	1	3	3	2	3				1	1	3
JCVIGM_011008_20110307	tpiA	triose-phosphate isomerase			1		1	1	1	1									
JCVIGM_020800_20110307	tpiA	triose-phosphate isomerase																	
JCVIGM_023613_20110307	tpiA	triose-phosphate isomerase		1			2	1	2										
JCVIGM_027321_20110307	tpiA	triose-phosphate isomerase																	
JCVIGM_087250_20110307	tpiA	triose-phosphate isomerase				1	1	1	2	1		3							
JCVIGM_115071_20110307	tpiA	triose-phosphate isomerase								1									
JCVIGM_135680_20110307	tpiA	triose-phosphate isomerase				1		1				1		1	1				
JCVIGM_261662_20110307	tpiA	triose-phosphate isomerase																	
JCVIGM_302433_20110307	tpiA	triose-phosphate isomerase				1				1									
JCVIGM_337367_20110307	tpiA	triose-phosphate isomerase																1	
JCVIGM_366477_20110307	tpiA	triose-phosphate isomerase																	
JCVIGM_114128_20110307		tRNA methyl transferase																	
JCVIGM_117733_20110307		two-component system sensor histidine kinase, with a response regulator receiver domain																	
JCVIGM_373409_20110307		type III restriction protein																	
JCVIGM_014974_20110307	galE	UDP-glucose 4-epimerase																	
JCVIGM_179048_20110307	murE	UDP-N-acetylmuramoyl-L-alanyl-D-glutamate--2,6-diaminopimelate ligase																	
JCVIGM_240853_20110307	uspA	universal stress protein A															1	1	
JCVIGM_311901_20110307		UPF0210 protein DORLON_00661																	
JCVIGM_044460_20110307		UTP--hexose-1-phosphate uridylyltransferase		1								1							
JCVIGM_148949_20110307	pepD	Xaa-His dipeptidase																	

JCVIGM_195935_20110307		xylose ABC transporter substrate-binding protein											1	1					
JCVIGM_086248_20110307	xylA	xylose isomerase																	
JCVIGM_113241_20110307	xylA	xylose isomerase																	
JCVIGM_120909_20110307	xylA	xylose isomerase																	
JCVIGM_229108_20110307		YjeF N-terminal domain protein																	

				Biological replicate 2-a															
				CONTROL								INFECTED							
Reference	Gene symbol	Protein description	DAY ->	-1	1	3	6	10	14	21	28	-1	1	3	6	10	14	21	28
JCVIGM_070246_20110307	gpmI	2,3-bisphosphoglycerate-independent phosphoglycerate mutase																	
JCVIGM_091222_20110307	gpmI	2,3-bisphosphoglycerate-independent phosphoglycerate mutase																	
JCVIGM_199914_20110307		2-dehydro-3-deoxygluconokinase																1	
JCVIGM_044539_20110307	eda	2-dehydro-3-deoxyphosphogluconate aldolase/4-hydroxy-2-oxoglutarate aldolase		4							1							1	
JCVIGM_057836_20110307	eda	2-dehydro-3-deoxyphosphogluconate aldolase/4-hydroxy-2-oxoglutarate aldolase		2	1	1		1	2	10			4						
JCVIGM_092015_20110307	eda	2-dehydro-3-deoxyphosphogluconate aldolase/4-hydroxy-2-oxoglutarate aldolase		2				1				1							
JCVIGM_360038_20110307	yhaE	2-hydroxy-3-oxopropionate reductase												2			1		
JCVIGM_374955_20110307		2-hydroxy-3-oxopropionate reductase		1															
JCVIGM_319522_20110307	kdsA	3-deoxy-8-phosphooctulonate synthase																	
JCVIGM_196595_20110307		3-hydroxyacyl-coA dehydrogenase		1	1			1		4			3						
JCVIGM_037673_20110307	hbd	3-hydroxybutyryl-coA dehydrogenase						1	1			1							
JCVIGM_037995_20110307	pdxB	4-phosphoerythronate dehydrogenase						1	1					1					
JCVIGM_259097_20110307	rplJ	50S ribosomal protein L10																	
JCVIGM_024389_20110307	rplK	50S ribosomal protein L11							1										
JCVIGM_045877_20110307	rplK	50S ribosomal protein L11				1		1				1							
JCVIGM_059265_20110307	rplK	50S ribosomal protein L11		2		1	2	1							1				
JCVIGM_033470_20110307	rplE	50S ribosomal protein L5																	
JCVIGM_155746_20110307	rplE	50S ribosomal protein L5																1	

JCVIGM_232804_20110307	rplE	50S ribosomal protein L5																	
JCVIGM_020270_20110307	rplL	50S ribosomal protein L7/L12																	
JCVIGM_125249_20110307	rplL	50S ribosomal protein L7/L12																	
JCVIGM_100458_20110307	ribH	6,7-dimethyl-8-ribityllumazine synthase		1															
JCVIGM_013177_20110307		60 kDa chaperonin		1															
JCVIGM_213822_20110307		60 kDa chaperonin										2							
JCVIGM_123049_20110307		ABC transporter periplasmic-binding protein ytfQ				1								1					
JCVIGM_295655_20110307		ABC transporter related		1															
JCVIGM_097008_20110307		ABC transporter, ATP-binding protein		1															
JCVIGM_168201_20110307		ABC transporter, ATP-binding protein									1								
JCVIGM_070783_20110307		ABC transporter, permease protein																	
JCVIGM_179750_20110307		ABC transporter, substrate-binding protein																	
JCVIGM_284177_20110307		ABC transporter, substrate-binding protein, family 1							1										
JCVIGM_284885_20110307		ABC-type dipeptide transport system, periplasmic component																	
JCVIGM_168588_20110307		ABC-type sugar transport system, periplasmic component					1	1											
JCVIGM_297706_20110307		ABC-type sugar transport system, periplasmic component																	2
JCVIGM_162628_20110307		ABC-type transport system, substrate-binding component																	
JCVIGM_197130_20110307		ABC-type xylose transport system, periplasmic component		2		1	2		1	2	2	2							
JCVIGM_034585_20110307	ackA	acetate kinase					1												
JCVIGM_090369_20110307	ackA	acetate kinase		2															
JCVIGM_103872_20110307	ackA	acetate kinase									1								
JCVIGM_105479_20110307	ackA	acetate kinase																	
JCVIGM_313779_20110307	ackA	acetate kinase						1											
JCVIGM_120726_20110307	ilvC	acetohydroxy acid isomeroreductase, catalytic domain																	
JCVIGM_136868_20110307	ilvC	acetohydroxy acid isomeroreductase, catalytic domain		3	3	4	2	2	3	1	3	4	6	2		1	1	2	1
JCVIGM_141542_20110307	ilvC	acetohydroxy acid isomeroreductase, catalytic domain		2			3	1	2		1	3						1	
JCVIGM_256491_20110307	ilvC	acetohydroxy acid isomeroreductase, catalytic domain		2															
JCVIGM_298658_20110307	ilvC	acetohydroxy acid isomeroreductase, catalytic domain		2			1				1								
JCVIGM_383122_20110307	ilvC	acetohydroxy acid isomeroreductase, catalytic domain				2	1	1	1		1							1	
JCVIGM_029818_20110307	ilvN	acetolactate synthase, small subunit								1									
JCVIGM_125179_20110307		acetyl-coA acetyltransferases																	

JCVIGM_101296_20110307		acetyl-coA C-acetyltransferase					1		2										
JCVIGM_182295_20110307		acetyl-coA C-acetyltransferase		9			2	1	3		3	3		1	1			2	
JCVIGM_234799_20110307		acetyl-coA C-acetyltransferase					2	3	4		4	3		1	1			6	5
JCVIGM_306189_20110307	phaA	acetyl-CoA C-acetyltransferase					1					1							
JCVIGM_299975_20110307		acyltransferase family					1												
JCVIGM_058622_20110307	purA	adenylosuccinate synthase																	
JCVIGM_064826_20110307	purA	adenylosuccinate synthase																	
JCVIGM_092236_20110307	purA	adenylosuccinate synthase																	
JCVIGM_092238_20110307	purA	adenylosuccinate synthase																	
JCVIGM_108189_20110307		ahpC/TSA family		1															
JCVIGM_145159_20110307	ald	alanine dehydrogenase																	
JCVIGM_391701_20110307	tdh	alcohol dehydrogenase groES domain protein																	
JCVIGM_144742_20110307		alcohol dehydrogenase, iron-dependent																	
JCVIGM_147392_20110307		aldehyde oxidase and xanthine dehydrogenase molybdopterin binding protein		3															
JCVIGM_050239_20110307		alpha-amylase																	
JCVIGM_248140_20110307		alpha-D-mannosidase																	
JCVIGM_337826_20110307		alpha-D-mannosidase									1								
JCVIGM_358636_20110307		alpha-D-mannosidase									2	1							
JCVIGM_076247_20110307		alpha-galactosidase									1							1	
JCVIGM_090969_20110307		alpha-galactosidase																	
JCVIGM_235809_20110307		alpha-galactosidase																	1
JCVIGM_094959_20110307		alpha-N-arabinofuranosidase		2															
JCVIGM_179557_20110307		alpha-N-arabinofuranosidase																	
JCVIGM_043430_20110307		arabinogalactan endo-1,4-beta-galactosidase		2								1							
JCVIGM_054434_20110307		arabinogalactan endo-1,4-beta-galactosidase		2			1				1								
JCVIGM_030203_20110307	fucI	arabinose isomerase		1															
JCVIGM_108898_20110307	fucI	arabinose isomerase		1	1							1							
JCVIGM_151443_20110307	fucI	arabinose isomerase																	
JCVIGM_188730_20110307	fucI	arabinose isomerase							1				2						

JCVIGM_191949_20110307	fucI	arabinose isomerase																	
JCVIGM_339907_20110307	fucI	arabinose isomerase																	
JCVIGM_341670_20110307	fucI	arabinose isomerase										1							
JCVIGM_019632_20110307	arcA	arginine deiminase																1	
JCVIGM_030954_20110307	asnB	asparagine synthase (glutamine-hydrolyzing)		1							1								
JCVIGM_149591_20110307	aspA	aspartate ammonia-lyase																	
JCVIGM_076159_20110307	asd	aspartate-semialdehyde dehydrogenase																1	
JCVIGM_163401_20110307	asd	aspartate-semialdehyde dehydrogenase							1										
JCVIGM_146114_20110307	aspS	aspartate--tRNA ligase																	
JCVIGM_225435_20110307	atpA	ATP synthase F1, alpha subunit																	
JCVIGM_193732_20110307		ATP synthase subunit a																	
JCVIGM_181280_20110307		B12 binding domain		1															
JCVIGM_335931_20110307		basic membrane lipoprotein																	
JCVIGM_191196_20110307		beta-galactosidase						1											
JCVIGM_065702_20110307	bglA	beta-glucosidase A									1							1	
JCVIGM_026370_20110307		beta-glucosidase-n-terminal domain-containing protein																	
JCVIGM_156507_20110307		beta-lactamase																	
JCVIGM_042339_20110307	purH	bifunctional purine biosynthesis protein purH																	
JCVIGM_036791_20110307	buk	butyrate kinase									1							1	
JCVIGM_216035_20110307		butyryl-coA dehydrogenase																	
JCVIGM_265820_20110307		butyryl-coA dehydrogenase		2			3	3	1		2	4		1				1	
JCVIGM_290061_20110307		butyryl-coA dehydrogenase					1												
JCVIGM_329946_20110307	bcd2	butyryl-coA dehydrogenase																	
JCVIGM_040543_20110307	c7d2-bg	C7D2-BG protein																1	
JCVIGM_017812_20110307		carbohydrate ABC transporter substrate-binding protein, CUT1 family																	
JCVIGM_055645_20110307		carbohydrate ABC transporter substrate-binding protein, CUT1 family																	
JCVIGM_306701_20110307		carbohydrate ABC transporter substrate-binding protein, CUT1 family																	
JCVIGM_074670_20110307		carbohydrate ABC transporter substrate-binding protein, CUT1 family		2															
JCVIGM_324855_20110307		carbohydrate ABC transporter, carbohydrate-binding protein		1			1	2	1			2							
JCVIGM_049249_20110307	cooS	carbon-monoxide dehydrogenase, catalytic subunit		3					1			1						1	

JCVIGM_000407_20110307	dnaK	chaperone protein dnaK																1	
JCVIGM_021700_20110307	dnaK	chaperone protein dnaK																	
JCVIGM_031425_20110307	dnaK	chaperone protein dnaK		1											1				
JCVIGM_032528_20110307	dnaK	chaperone protein dnaK		1			1					1			1			1	
JCVIGM_034511_20110307	dnaK	chaperone protein dnaK																	
JCVIGM_043298_20110307	dnaK	chaperone protein dnaK																	
JCVIGM_062282_20110307	dnaK	chaperone protein dnaK																	
JCVIGM_122947_20110307	dnaK	chaperone protein dnaK																	
JCVIGM_147511_20110307	dnaK	chaperone protein dnaK												1					
JCVIGM_213960_20110307	dnaK	chaperone protein dnaK						1											
JCVIGM_236768_20110307	dnaK	chaperone protein dnaK																	
JCVIGM_086927_20110307	groS	chaperonin 10 Kd subunit					1			1									
JCVIGM_187049_20110307	groS	chaperonin 10 Kd subunit		2															
JCVIGM_213821_20110307	groS	chaperonin 10 Kd subunit					1												
JCVIGM_401604_20110307	groS	chaperonin 10 Kd subunit																	
JCVIGM_413734_20110307		CO dehydrogenase/acetyl-coA synthase complex, beta subunit						1	1		1	2							
JCVIGM_049251_20110307	cdhC	CO dehydrogenase/CO-methylating acetyl-coA synthase complex, beta subunit		4															
JCVIGM_339054_20110307		coenzyme B12-dependent glycerol dehydratase, large subunit									1								
JCVIGM_212787_20110307		cof-like hydrolase																	
JCVIGM_127608_20110307		ComEC/Rec2-like protein									1								
JCVIGM_286641_20110307		conserved hypothetical protein																	
JCVIGM_049253_20110307		corrinoid/iron-sulfur protein, large subunit						1			1	1							
JCVIGM_049252_20110307		corrinoid/iron-sulfur protein, small subunit																	
JCVIGM_022873_20110307	cysK	cysteine synthase A																	
JCVIGM_307840_20110307		dehydrogenase, FMN-dependent																	
JCVIGM_102159_20110307	deoC	deoxyribose-phosphate aldolase		3			2	1	1			2						1	
JCVIGM_056340_20110307		diaminopimelate dehydrogenase																	
JCVIGM_104884_20110307		diaminopimelate dehydrogenase						1			1								
JCVIGM_002401_20110307	lpdA	dihydrolipoyl dehydrogenase																	
JCVIGM_139913_20110307	pyrD	dihydroorotate oxidase		1				1	1									1	

JCVIGM_049254_20110307		dihydropteroate synthase, DHPS		1															
JCVIGM_130560_20110307	ilvD	dihydroxy-acid dehydratase																	
JCVIGM_377265_20110307	disA	DNA integrity scanning protein disA																	
JCVIGM_176486_20110307	dnaN	DNA polymerase III, beta subunit									1								
JCVIGM_228057_20110307	etfB	electron transfer flavoprotein, alpha subunit																	
JCVIGM_310193_20110307	etfB	electron transfer flavoprotein, alpha subunit																	
JCVIGM_316234_20110307	etfA	electron transfer flavoprotein, alpha subunit/fixB family protein										1							
JCVIGM_083913_20110307	etfB	electron transfer flavoprotein, beta subunit		2			1	1	1		3	1							
JCVIGM_215068_20110307	etfB	electron transfer flavoprotein, beta subunit										1							
JCVIGM_316431_20110307		electron transfer flavoprotein, beta subunit																	
JCVIGM_041452_20110307	tuf	elongation factor Tu (fragment)																	
JCVIGM_148583_20110307		elongation factor Tu (fragment)																	
JCVIGM_209439_20110307	tuf	elongation factor Tu (fragment)		1									1						
JCVIGM_239217_20110307	tuf1	elongation factor Tu 1																	
JCVIGM_045263_20110307		extracellular solute-binding protein family 1				1	1	1	1	1	2	1							
JCVIGM_088711_20110307		extracellular solute-binding protein family 1		1															
JCVIGM_096968_20110307		extracellular solute-binding protein family 1					1												
JCVIGM_108783_20110307		extracellular solute-binding protein family 1		1															
JCVIGM_212868_20110307		extracellular solute-binding protein family 1																	
JCVIGM_275107_20110307		extracellular solute-binding protein family 1																	
JCVIGM_291167_20110307		extracellular solute-binding protein family 1		1			1	1	1	2		1	1						
JCVIGM_192080_20110307		extracellular solute-binding protein family 5																	
JCVIGM_259518_20110307		ferritin 1																	
JCVIGM_308648_20110307		fibronectin type III domain																	
JCVIGM_400677_20110307		fla1 flagellin						1						1					
JCVIGM_423664_20110307		fla1 flagellin (fragment)																	
JCVIGM_430871_20110307		fla3 flagellin		4			3	3	4		2	2		4			1	2	
JCVIGM_416087_20110307	flgE1	flagellar hook protein flgE1																	
JCVIGM_077354_20110307		flagellin (fragment)					1	2		1		1	3	1		1	2	1	3
JCVIGM_172838_20110307		flagellin (fragment)						1										1	
JCVIGM_022208_20110307		flagellin and related hook-associated proteins		3		1	2	4	3		4	1		3		2	3		1

JCVIGM_154194_20110307		flagellin and related hook-associated proteins																	
JCVIGM_190042_20110307		flagellin and related hook-associated proteins																	
JCVIGM_389298_20110307		flagellin and related hook-associated proteins							1										
JCVIGM_188452_20110307		flagellin domain protein		1		1	1	1	2	1	1	1		1	1			2	1
JCVIGM_346020_20110307		flagellin domain protein																	
JCVIGM_391229_20110307		flagellin domain protein																	
JCVIGM_069861_20110307	fliC1	flagellin fliC1					1	2	1		2							1	
JCVIGM_410616_20110307	fliC1	flagellin fliC1		3			2	2	2	1		1		1					
JCVIGM_152287_20110307	fliC2	flagellin fliC2					1	1		1		1				1			1
JCVIGM_177955_20110307	fliC2	flagellin fliC2		2				1				1							
JCVIGM_262501_20110307	fliC2	flagellin fliC2																	
JCVIGM_187473_20110307	fliC4	flagellin fliC4					1												
JCVIGM_189905_20110307	fliC5	flagellin fliC5								1									
JCVIGM_392640_20110307		flagellin protein																	
JCVIGM_402856_20110307		flagellin protein		1															
JCVIGM_414958_20110307		flagellin protein																1	
JCVIGM_424682_20110307		flagellin protein									1								
JCVIGM_152365_20110307	fprA	flavodoxin																	
JCVIGM_148596_20110307		formate acetyltransferase (fragment)																	
JCVIGM_185080_20110307		formate acetyltransferase (fragment)																	
JCVIGM_246004_20110307		formate acetyltransferase (fragment)									1								
JCVIGM_032907_20110307	pflB	formate C-acetyltransferase		2				2	1	2	2	3						1	1
JCVIGM_043008_20110307	pflB	formate C-acetyltransferase		7			4	1	2	1	1	3		3	1			6	
JCVIGM_157210_20110307		formate C-acetyltransferase																	
JCVIGM_229801_20110307		formate C-acetyltransferase		1				1				1						1	
JCVIGM_249731_20110307		formate C-acetyltransferase		1				1	2		3	5						7	
JCVIGM_282796_20110307		formate C-acetyltransferase																	
JCVIGM_317542_20110307		formate C-acetyltransferase			1		1	1		1	2	1	1	2				1	
JCVIGM_356609_20110307		formate C-acetyltransferase																	
JCVIGM_417381_20110307		formate C-acetyltransferase																	
JCVIGM_078607_20110307	fhs	formate--tetrahydrofolate ligase																	

JCVIGM_118473_20110307	fhs	formate--tetrahydrofolate ligase		2															
JCVIGM_123964_20110307	fhs	formate--tetrahydrofolate ligase		1		1	3	2	2	1	3	4						2	
JCVIGM_138264_20110307	fhs	formate--tetrahydrofolate ligase																	
JCVIGM_162244_20110307	fhs	formate--tetrahydrofolate ligase																	
JCVIGM_176151_20110307	fhs	formate--tetrahydrofolate ligase																	
JCVIGM_245005_20110307	fhs	formate--tetrahydrofolate ligase		4			3	4	3		5	5		2				1	
JCVIGM_400807_20110307	fhs	formate--tetrahydrofolate ligase																	
JCVIGM_122704_20110307	fhs1	formate--tetrahydrofolate ligase 1																	
JCVIGM_146104_20110307	fhs2	formate--tetrahydrofolate ligase 2		20			6	8	6	1	4	8		1				5	
JCVIGM_032208_20110307	frc	formyl-coA transferase																	
JCVIGM_065785_20110307	frc	formyl-CoA transferase										1	1						1
JCVIGM_070965_20110307	frc	formyl-coA transferase					2	1	1		1	1							
JCVIGM_132967_20110307	frc	formyl-coA transferase		5		1	4	1			2	2			1				
JCVIGM_231668_20110307	frc	formyl-coA transferase		1								1							
JCVIGM_254826_20110307	frc	formyl-coA transferase																	
JCVIGM_051280_20110307		fructokinase																	
JCVIGM_079013_20110307		fructose-1,6-bisphosphate aldolase, class II																	
JCVIGM_087474_20110307	fba	fructose-1,6-bisphosphate aldolase, class II		2			3	5	8		2	4						2	
JCVIGM_118274_20110307	fba	fructose-1,6-bisphosphate aldolase, class II					1	2	1		3	2		1				1	
JCVIGM_000284_20110307	fsa	fructose-6-phosphate aldolase		1				1											
JCVIGM_153991_20110307	fsa	fructose-6-phosphate aldolase		1		1	1	1	2		1								
JCVIGM_393658_20110307	fsa	fructose-6-phosphate aldolase																	
JCVIGM_231559_20110307		fructose-bisphosphate aldolase					1	1	2			2		1					
JCVIGM_089609_20110307		fructose-bisphosphate aldolase class-I																	
JCVIGM_336730_20110307		galactose ABC transporter, periplasmic galactose-binding protein										2						1	
JCVIGM_027728_20110307		galactose/glucose-binding lipoprotein																	
JCVIGM_266085_20110307	galT	galactose-1-phosphate uridylyltransferase galT																1	
JCVIGM_165424_20110307		gliding motility-related protein																	
JCVIGM_351864_20110307		glu/leu/phe/val dehydrogenase, dimerization domain protein																	
JCVIGM_138714_20110307		gluconate 5-dehydrogenase					1												

JCVIGM_002867_20110307	pgi	glucose-6-phosphate isomerase									2	1			2			1	
JCVIGM_015397_20110307	pgi	glucose-6-phosphate isomerase							1										
JCVIGM_015745_20110307	pgi	glucose-6-phosphate isomerase		2	1	2			4	1	2	2	1	1			1	3	
JCVIGM_026127_20110307	pgi	glucose-6-phosphate isomerase																	
JCVIGM_030449_20110307	pgi	glucose-6-phosphate isomerase		1			3	5	2		1	1		1				2	
JCVIGM_042043_20110307	pgi	glucose-6-phosphate isomerase																	
JCVIGM_054834_20110307	pgi	glucose-6-phosphate isomerase																	
JCVIGM_073687_20110307	pgi	glucose-6-phosphate isomerase																	
JCVIGM_075891_20110307	pgi	glucose-6-phosphate isomerase																	1
JCVIGM_080716_20110307	pgi	glucose-6-phosphate isomerase																	
JCVIGM_091996_20110307	pgi	glucose-6-phosphate isomerase		2			1	5			2	2							
JCVIGM_116144_20110307	pgi	glucose-6-phosphate isomerase						1											
JCVIGM_147397_20110307	pgi	glucose-6-phosphate isomerase		2															
JCVIGM_150388_20110307	pgi	glucose-6-phosphate isomerase																	
JCVIGM_154594_20110307	pgi	glucose-6-phosphate isomerase		1			1	2	2	10	2	3	7	1		1		3	4
JCVIGM_170286_20110307	pgi	glucose-6-phosphate isomerase		3			1		1		2	3		1				1	
JCVIGM_208717_20110307	pgi	glucose-6-phosphate isomerase					1				1								
JCVIGM_221773_20110307	pgi	glucose-6-phosphate isomerase		1	1	1	1	2	2	1			1			1			2
JCVIGM_229071_20110307		glucose-6-phosphate isomerase		1			1	5	6		2	2		1				2	
JCVIGM_245845_20110307	pgi	glucose-6-phosphate isomerase																	
JCVIGM_257476_20110307	pgi	glucose-6-phosphate isomerase							1		1								
JCVIGM_261055_20110307	pgi	glucose-6-phosphate isomerase																	
JCVIGM_286986_20110307	pgi	glucose-6-phosphate isomerase		2			1	3	2		4	2						1	
JCVIGM_296222_20110307	pgi	glucose-6-phosphate isomerase		1							3	1		1				2	
JCVIGM_296223_20110307	pgi	glucose-6-phosphate isomerase																	
JCVIGM_346043_20110307	pgi	glucose-6-phosphate isomerase																	
JCVIGM_407434_20110307	pgi	glucose-6-phosphate isomerase		4			1	2	1		3							4	2
JCVIGM_433684_20110307	pgi	glucose-6-phosphate isomerase																	
JCVIGM_036452_20110307	pgiA	glucose-6-phosphate isomerase A										1							
JCVIGM_001673_20110307	uxaC	glucuronate isomerase																	

JCVIGM_001890_20110307	uxaC	glucuronate isomerase																	
JCVIGM_105608_20110307	uxaC	glucuronate isomerase																	
JCVIGM_028337_20110307		glutamate dehydrogenase																	
JCVIGM_079729_20110307	GDH	glutamate dehydrogenase (fragment)																	
JCVIGM_026222_20110307		glutamate dehydrogenase (NADP(+))						1											
JCVIGM_051813_20110307		glutamate dehydrogenase, nADP-specific		1															
JCVIGM_081547_20110307		glutamate dehydrogenase, NADP-specific		5	4	3	2	3	3	4	4	4	5	1		1	1	3	6
JCVIGM_083070_20110307		glutamate dehydrogenase, NADP-specific																	
JCVIGM_112581_20110307		glutamate dehydrogenase, NADP-specific																	
JCVIGM_235230_20110307		glutamate dehydrogenase, NADP-specific		1				1		1		1	1					1	
JCVIGM_395419_20110307		glutamate dehydrogenase, NADP-specific									1							1	1
JCVIGM_000822_20110307	gdhB	glutamate dehydrogenase, NAD-specific		1				2						1					
JCVIGM_031962_20110307	gdhB	glutamate dehydrogenase, NAD-specific																	
JCVIGM_033111_20110307	gdhB	glutamate dehydrogenase, NAD-specific		1															
JCVIGM_044177_20110307	gdhB	glutamate dehydrogenase, NAD-specific																	
JCVIGM_072261_20110307	gdh	glutamate dehydrogenase, NAD-specific		1															1
JCVIGM_124713_20110307	gdhB	glutamate dehydrogenase, NAD-specific																	
JCVIGM_030438_20110307		glutamate dehydrogenase/leucine dehydrogenase																	
JCVIGM_067262_20110307		glutamate dehydrogenase/leucine dehydrogenase																	
JCVIGM_071296_20110307		glutamate dehydrogenase/leucine dehydrogenase						1											
JCVIGM_130579_20110307		glutamate dehydrogenase/leucine dehydrogenase						1		1			2						
JCVIGM_141521_20110307		glutamate dehydrogenase/leucine dehydrogenase		2		2	1	3	2	1	5	3	1	1				1	
JCVIGM_226798_20110307		glutamate dehydrogenase/leucine dehydrogenase		1			1	2	2		1	1	1	1	1			1	
JCVIGM_167424_20110307	gltA	glutamate synthase (NADPH), homotetrameric										1							
JCVIGM_009248_20110307	gap	glyceraldehyde 3-phosphate dehydrogenase, C-terminal domain		1														1	
JCVIGM_027319_20110307		glyceraldehyde 3-phosphate dehydrogenase, C-terminal domain		2				2	2		1	2							
JCVIGM_075124_20110307		glyceraldehyde 3-phosphate dehydrogenase, C-terminal domain																	
JCVIGM_079320_20110307		glyceraldehyde 3-phosphate dehydrogenase, C-terminal domain							1			1							
JCVIGM_083011_20110307		glyceraldehyde 3-phosphate dehydrogenase, C-terminal domain																	
JCVIGM_088090_20110307		glyceraldehyde 3-phosphate dehydrogenase, C-terminal domain					1	1			1			1				1	
JCVIGM_141419_20110307		glyceraldehyde 3-phosphate dehydrogenase, C-terminal domain																	

JCVIGM_159787_20110307		glyceraldehyde 3-phosphate dehydrogenase, C-terminal domain																	
JCVIGM_169660_20110307		glyceraldehyde 3-phosphate dehydrogenase, C-terminal domain																	
JCVIGM_170407_20110307		glyceraldehyde 3-phosphate dehydrogenase, C-terminal domain		2	1	1	3	2			2	2	1	2		1	1	2	1
JCVIGM_189194_20110307		glyceraldehyde 3-phosphate dehydrogenase, C-terminal domain		4		2	2	3	2	1	2	1			1		1	1	
JCVIGM_224384_20110307		glyceraldehyde 3-phosphate dehydrogenase, C-terminal domain										1							
JCVIGM_268659_20110307		glyceraldehyde 3-phosphate dehydrogenase, C-terminal domain									1								
JCVIGM_313872_20110307		glyceraldehyde 3-phosphate dehydrogenase, C-terminal domain																	
JCVIGM_319196_20110307		glyceraldehyde 3-phosphate dehydrogenase, C-terminal domain		1				1				1							
JCVIGM_360253_20110307		glyceraldehyde 3-phosphate dehydrogenase, C-terminal domain														1			
JCVIGM_100377_20110307	gap	glyceraldehyde-3-phosphate dehydrogenase, type I		1	1	1		1	3		4	1	1	1				1	
JCVIGM_294702_20110307	pduC	glycerol dehydratase large subunit					2	1	2			2							
JCVIGM_338330_20110307		glycerol dehydrogenase and related enzymes												1					
JCVIGM_213371_20110307	glyA	glycine hydroxymethyltransferase					1												
JCVIGM_015371_20110307		histidine kinase																	
JCVIGM_369000_20110307		hydrogenase, Fe-only					1		1										
JCVIGM_048518_20110307		hydrolyase, tartrate beta subunit/fumarate domain protein, Fe-S type																1	
JCVIGM_000577_20110307		hypothetical protein																	
JCVIGM_001656_20110307		hypothetical protein		2			1		1										
JCVIGM_004031_20110307		hypothetical protein				1								1					1
JCVIGM_006083_20110307		hypothetical protein																	
JCVIGM_006988_20110307		hypothetical protein		1															
JCVIGM_007410_20110307		hypothetical protein		2							2	1							
JCVIGM_009250_20110307		hypothetical protein																	
JCVIGM_010628_20110307		hypothetical protein		1			2				2							1	
JCVIGM_011271_20110307		hypothetical protein						2			1	1							
JCVIGM_011336_20110307		hypothetical protein																	
JCVIGM_012967_20110307		hypothetical protein					1		1	1									
JCVIGM_014024_20110307		hypothetical protein						1	2									1	
JCVIGM_018751_20110307		hypothetical protein																	
JCVIGM_020128_20110307		hypothetical protein		1					1										
JCVIGM_021326_20110307		hypothetical protein		13	2	1	3	5	6	2	2	4	2	3	1	2	2	1	

JCVIGM_021364_20110307		hypothetical protein																	
JCVIGM_021629_20110307		hypothetical protein						1			1								
JCVIGM_022560_20110307		hypothetical protein					1	1	1										
JCVIGM_022561_20110307		hypothetical protein																	
JCVIGM_025687_20110307		hypothetical protein												1				1	
JCVIGM_025940_20110307		hypothetical protein																	
JCVIGM_026248_20110307		hypothetical protein		1				1			1	1							
JCVIGM_026306_20110307		hypothetical protein		9	3	7	10	7	10	7	9	13	6	6	3	4	1	5	2
JCVIGM_029504_20110307		hypothetical protein																	
JCVIGM_029868_20110307		hypothetical protein					1	1											
JCVIGM_030581_20110307		hypothetical protein					1	1	1		3	1							
JCVIGM_031963_20110307		hypothetical protein																	
JCVIGM_036375_20110307		hypothetical protein																	
JCVIGM_036678_20110307		hypothetical protein						1											
JCVIGM_037187_20110307		hypothetical protein		9		3	7	5	5		7	5		1	2	1	3	4	1
JCVIGM_039163_20110307		hypothetical protein		11	39	5	9	7	7	34	7	9	28	1	1	3		3	23
JCVIGM_039195_20110307		hypothetical protein		5	6		6	4	8	3	9	10	2	1				3	3
JCVIGM_042328_20110307		hypothetical protein		3					1			3		1					
JCVIGM_043843_20110307		hypothetical protein																	
JCVIGM_044734_20110307		hypothetical protein																	
JCVIGM_045412_20110307		hypothetical protein																	
JCVIGM_047061_20110307		hypothetical protein																	
JCVIGM_048990_20110307		hypothetical protein		1				1	1		1	1							
JCVIGM_052263_20110307		hypothetical protein																	
JCVIGM_054391_20110307		hypothetical protein										1							
JCVIGM_055922_20110307		hypothetical protein		2			2		2			1							
JCVIGM_056525_20110307		hypothetical protein																	
JCVIGM_057825_20110307		hypothetical protein					1	1	1		1							1	
JCVIGM_058381_20110307		hypothetical protein																	

JCVIGM_060468_20110307		hypothetical protein		1			1	1											
JCVIGM_060720_20110307		hypothetical protein		2															
JCVIGM_061313_20110307		hypothetical protein																	
JCVIGM_061338_20110307		hypothetical protein						2			2	1				1		1	
JCVIGM_063878_20110307		hypothetical protein																	
JCVIGM_065213_20110307		hypothetical protein																	
JCVIGM_068011_20110307		hypothetical protein					1								1				
JCVIGM_068020_20110307		hypothetical protein																	
JCVIGM_070048_20110307		hypothetical protein		1															
JCVIGM_070908_20110307		hypothetical protein																	
JCVIGM_071303_20110307		hypothetical protein																	
JCVIGM_071361_20110307		hypothetical protein									1								
JCVIGM_071586_20110307		hypothetical protein		2			1							1	1			1	
JCVIGM_071718_20110307		hypothetical protein																	
JCVIGM_072139_20110307		hypothetical protein																	1
JCVIGM_072463_20110307		hypothetical protein																	
JCVIGM_073483_20110307		hypothetical protein				1													
JCVIGM_074339_20110307		hypothetical protein		3				2			1	1						1	
JCVIGM_074623_20110307		hypothetical protein																	
JCVIGM_075200_20110307		hypothetical protein		3															
JCVIGM_077653_20110307		hypothetical protein		2			1					1							
JCVIGM_081664_20110307		hypothetical protein																	
JCVIGM_083533_20110307		hypothetical protein		3	1	1	3		1	2	3	3			1		2	1	1
JCVIGM_084534_20110307		hypothetical protein																	
JCVIGM_084535_20110307		hypothetical protein		1	1	1	1		1	2									
JCVIGM_088087_20110307		hypothetical protein																	
JCVIGM_088594_20110307		hypothetical protein		1			2	1	1		1	4		1				2	
JCVIGM_090261_20110307		hypothetical protein						1			1	1	1			1		1	
JCVIGM_090262_20110307		hypothetical protein									1	1						1	

JCVIGM_090263_20110307		hypothetical protein												1					
JCVIGM_090646_20110307		hypothetical protein																	
JCVIGM_091802_20110307		hypothetical protein																	
JCVIGM_091850_20110307		hypothetical protein																	
JCVIGM_092014_20110307		hypothetical protein		1			1		2		1	3				1			
JCVIGM_092247_20110307		hypothetical protein																	
JCVIGM_092708_20110307		hypothetical protein		3			1					2		1				2	
JCVIGM_095744_20110307		hypothetical protein																	
JCVIGM_097721_20110307		hypothetical protein																	
JCVIGM_098373_20110307		hypothetical protein																	
JCVIGM_101299_20110307		hypothetical protein																	
JCVIGM_101300_20110307	etfB	hypothetical protein																	
JCVIGM_102281_20110307		hypothetical protein		1			2	1		1	3	3	1	1				4	
JCVIGM_102458_20110307		hypothetical protein																	
JCVIGM_102522_20110307		hypothetical protein					1		1		1				1				
JCVIGM_102523_20110307		hypothetical protein		2			1		1	2	1	1						1	1
JCVIGM_103433_20110307		hypothetical protein		1			1	1											
JCVIGM_104408_20110307		hypothetical protein		1				1											
JCVIGM_107850_20110307		hypothetical protein																	
JCVIGM_110415_20110307		hypothetical protein						1				1						1	
JCVIGM_112258_20110307		hypothetical protein		1				1			2	1						1	
JCVIGM_113282_20110307		hypothetical protein		1			1	1										1	
JCVIGM_115169_20110307		hypothetical protein					3	2	1		1	2		1				1	
JCVIGM_115489_20110307		hypothetical protein																	
JCVIGM_116061_20110307		hypothetical protein																	
JCVIGM_116471_20110307		hypothetical protein																	
JCVIGM_118383_20110307		hypothetical protein							1			1	1						
JCVIGM_121048_20110307		hypothetical protein																	
JCVIGM_121384_20110307		hypothetical protein																	
JCVIGM_121883_20110307		hypothetical protein		2	2	1		1	4	2	3	1	2	1	2		2	4	1
JCVIGM_122516_20110307		hypothetical protein		1															
JCVIGM_125587_20110307		hypothetical protein																	

JCVIGM_126576_20110307		hypothetical protein					1					1							
JCVIGM_128170_20110307		hypothetical protein										1						1	
JCVIGM_128306_20110307		hypothetical protein		5															
JCVIGM_129017_20110307		hypothetical protein									1	1							
JCVIGM_129660_20110307		hypothetical protein																	
JCVIGM_130216_20110307		hypothetical protein		1															
JCVIGM_132903_20110307		hypothetical protein		4		2	6	7	5		3	5	1			2			
JCVIGM_133803_20110307		hypothetical protein																1	
JCVIGM_134883_20110307		hypothetical protein						1			1								
JCVIGM_135963_20110307		hypothetical protein																	
JCVIGM_141001_20110307		hypothetical protein						1	1			1						2	
JCVIGM_141853_20110307		hypothetical protein		2			2	3	1		3	2							
JCVIGM_142860_20110307		hypothetical protein									1							1	
JCVIGM_144970_20110307		hypothetical protein																	
JCVIGM_147926_20110307		hypothetical protein		1			1	4	3		3	3	1					2	
JCVIGM_148595_20110307		hypothetical protein						2				1							
JCVIGM_148602_20110307		hypothetical protein																	
JCVIGM_148969_20110307		hypothetical protein					1					1	1						1
JCVIGM_150315_20110307		hypothetical protein																	
JCVIGM_150334_20110307		hypothetical protein																	
JCVIGM_150335_20110307		hypothetical protein		1			2	2	3	1	2	1		2				1	
JCVIGM_153727_20110307		hypothetical protein		1				2	1		1							1	
JCVIGM_155277_20110307		hypothetical protein																	
JCVIGM_156043_20110307		hypothetical protein		1								1							
JCVIGM_160972_20110307		hypothetical protein																	
JCVIGM_161538_20110307		hypothetical protein																	
JCVIGM_161966_20110307		hypothetical protein																	
JCVIGM_163002_20110307		hypothetical protein																	
JCVIGM_166279_20110307		hypothetical protein						1											
JCVIGM_169083_20110307		hypothetical protein					1												
JCVIGM_170674_20110307		hypothetical protein		1	1			1		2			2						1
JCVIGM_173056_20110307		hypothetical protein		1								1							
JCVIGM_173583_20110307		hypothetical protein																	
JCVIGM_175085_20110307		hypothetical protein		2		1	2	3	3		1	1		1				2	

JCVIGM_175220_20110307		hypothetical protein						1	2			1							
JCVIGM_176221_20110307		hypothetical protein					1		1										
JCVIGM_181018_20110307		hypothetical protein		4			1	1	1	1		1	1					2	
JCVIGM_182077_20110307		hypothetical protein											1						
JCVIGM_185211_20110307		hypothetical protein																	
JCVIGM_185931_20110307		hypothetical protein		1				1	1										
JCVIGM_187544_20110307		hypothetical protein										2							
JCVIGM_189376_20110307		hypothetical protein																	
JCVIGM_189604_20110307		hypothetical protein																	
JCVIGM_190292_20110307		hypothetical protein																	
JCVIGM_190977_20110307		hypothetical protein																	
JCVIGM_191291_20110307		hypothetical protein					1												
JCVIGM_191293_20110307		hypothetical protein					1			1	2		2						
JCVIGM_191827_20110307		hypothetical protein										1							
JCVIGM_191888_20110307		hypothetical protein																	
JCVIGM_192065_20110307		hypothetical protein																	
JCVIGM_194742_20110307		hypothetical protein									1								
JCVIGM_194897_20110307		hypothetical protein																	
JCVIGM_195386_20110307		hypothetical protein																	
JCVIGM_196029_20110307		hypothetical protein									1	1							
JCVIGM_202561_20110307		hypothetical protein							1									1	
JCVIGM_203237_20110307		hypothetical protein																	
JCVIGM_204096_20110307		hypothetical protein						1											
JCVIGM_205608_20110307		hypothetical protein																	
JCVIGM_207243_20110307		hypothetical protein																	
JCVIGM_209517_20110307		hypothetical protein		2	1		2		2	1		1							
JCVIGM_210696_20110307		hypothetical protein																	
JCVIGM_210946_20110307		hypothetical protein					1	1			1	1							
JCVIGM_211394_20110307		hypothetical protein					1			1	1	1							
JCVIGM_212706_20110307		hypothetical protein						1											
JCVIGM_213389_20110307		hypothetical protein					1			1		1	1						1
JCVIGM_219434_20110307		hypothetical protein					1												
JCVIGM_219781_20110307		hypothetical protein					1											1	

JCVIGM_222747_20110307		hypothetical protein																	
JCVIGM_229339_20110307		hypothetical protein																	
JCVIGM_234241_20110307		hypothetical protein					2	2			1	2		1					
JCVIGM_237601_20110307		hypothetical protein				1										3			
JCVIGM_239052_20110307		hypothetical protein																	
JCVIGM_245141_20110307		hypothetical protein		1	1	1		3	2	2		3	1	1		1	2	4	
JCVIGM_246343_20110307		hypothetical protein																	
JCVIGM_246344_20110307		hypothetical protein																	
JCVIGM_247614_20110307		hypothetical protein									1								
JCVIGM_249782_20110307		hypothetical protein													3			1	
JCVIGM_251147_20110307		hypothetical protein																	
JCVIGM_251362_20110307		hypothetical protein		4		2	4	4	3	2	5	6	1	2			1	3	
JCVIGM_251712_20110307		hypothetical protein					1				1								
JCVIGM_256398_20110307		hypothetical protein		1															
JCVIGM_259370_20110307		hypothetical protein																	
JCVIGM_262707_20110307		hypothetical protein																	
JCVIGM_265819_20110307	etfB	hypothetical protein																	
JCVIGM_268646_20110307		hypothetical protein										1							
JCVIGM_274621_20110307		hypothetical protein													1				
JCVIGM_277806_20110307		hypothetical protein		1					1		1	1							
JCVIGM_281111_20110307		hypothetical protein											1						2
JCVIGM_281956_20110307		hypothetical protein																	
JCVIGM_282080_20110307		hypothetical protein																	
JCVIGM_283350_20110307		hypothetical protein																	
JCVIGM_286196_20110307		hypothetical protein											1				1	1	
JCVIGM_286605_20110307		hypothetical protein		3		1	3	2	5	1		3		1		1	1	1	
JCVIGM_288269_20110307		hypothetical protein		1		1	1	3	1		2	4						2	
JCVIGM_288716_20110307		hypothetical protein																	
JCVIGM_291424_20110307		hypothetical protein						1	1			1							
JCVIGM_295105_20110307		hypothetical protein																	

JCVIGM_295523_20110307		hypothetical protein						3	3		4	3						1	
JCVIGM_296216_20110307		hypothetical protein		6			2	3	1		3	2	2	1	1			6	2
JCVIGM_298432_20110307		hypothetical protein																	
JCVIGM_298496_20110307		hypothetical protein																	
JCVIGM_301982_20110307		hypothetical protein																	
JCVIGM_302456_20110307		hypothetical protein						1	1			1						1	
JCVIGM_303429_20110307		hypothetical protein																	
JCVIGM_303566_20110307		hypothetical protein																	
JCVIGM_303609_20110307		hypothetical protein		1							1								
JCVIGM_303798_20110307		hypothetical protein					1	1	1		2		1						
JCVIGM_306883_20110307		hypothetical protein																	
JCVIGM_306884_20110307		hypothetical protein		1				1				2							
JCVIGM_307057_20110307		hypothetical protein							2										
JCVIGM_307436_20110307		hypothetical protein		1				1			1	1						1	
JCVIGM_307765_20110307		hypothetical protein					1	2	1		1								
JCVIGM_309808_20110307		hypothetical protein		1	1	1	1	1	1	1		1		1				1	
JCVIGM_310529_20110307		hypothetical protein																	
JCVIGM_313684_20110307		hypothetical protein																	
JCVIGM_314099_20110307		hypothetical protein					1												
JCVIGM_314549_20110307		hypothetical protein		2			1	1	1	2	2		1	1				1	
JCVIGM_319934_20110307		hypothetical protein									1	1							
JCVIGM_322352_20110307		hypothetical protein																	
JCVIGM_323810_20110307		hypothetical protein																	
JCVIGM_327403_20110307		hypothetical protein					1					1							
JCVIGM_328614_20110307		hypothetical protein																	
JCVIGM_332056_20110307		hypothetical protein										1							
JCVIGM_332688_20110307		hypothetical protein			1		1				1	1					1		
JCVIGM_333745_20110307		hypothetical protein		1	1			1				1	1						

JCVIGM_336004_20110307		hypothetical protein																	
JCVIGM_336057_20110307		hypothetical protein						1										1	
JCVIGM_340014_20110307		hypothetical protein																	
JCVIGM_340609_20110307		hypothetical protein		2	1		3	4	3	2	2	4	5					2	1
JCVIGM_341200_20110307		hypothetical protein		2				1			1			1					
JCVIGM_344661_20110307		hypothetical protein									1								
JCVIGM_345968_20110307		hypothetical protein		1															
JCVIGM_348560_20110307		hypothetical protein					1	2											
JCVIGM_351456_20110307		hypothetical protein										1							
JCVIGM_359900_20110307		hypothetical protein						1				1							
JCVIGM_362686_20110307		hypothetical protein		1	1							1	2						
JCVIGM_364085_20110307		hypothetical protein		1							1								
JCVIGM_365092_20110307		hypothetical protein																	
JCVIGM_367321_20110307		hypothetical protein																	
JCVIGM_367720_20110307		hypothetical protein		2		1				1	1	1		1				1	
JCVIGM_368650_20110307		hypothetical protein																	
JCVIGM_369484_20110307		hypothetical protein		1							1	3							
JCVIGM_371571_20110307		hypothetical protein																	
JCVIGM_372380_20110307		hypothetical protein																	
JCVIGM_373222_20110307		hypothetical protein									1								
JCVIGM_373315_20110307		hypothetical protein																	
JCVIGM_375019_20110307		hypothetical protein																	
JCVIGM_379110_20110307		hypothetical protein		1				1			1	1							
JCVIGM_380771_20110307		hypothetical protein						1											
JCVIGM_387933_20110307		hypothetical protein																	
JCVIGM_390199_20110307		hypothetical protein																	
JCVIGM_390242_20110307		hypothetical protein																	
JCVIGM_390806_20110307		hypothetical protein					2	1	1		1	1							
JCVIGM_391550_20110307		hypothetical protein																	

JCVIGM_393860_20110307		hypothetical protein																	
JCVIGM_398055_20110307		hypothetical protein																	
JCVIGM_398168_20110307		hypothetical protein																	
JCVIGM_399462_20110307		hypothetical protein		1							1							1	
JCVIGM_400027_20110307		hypothetical protein		1				1	2										
JCVIGM_400278_20110307		hypothetical protein									2							1	
JCVIGM_400950_20110307		hypothetical protein																	
JCVIGM_405787_20110307		hypothetical protein																	
JCVIGM_410986_20110307		hypothetical protein																	
JCVIGM_414552_20110307		hypothetical protein							1					1					
JCVIGM_418955_20110307		hypothetical protein												1	5	1			
JCVIGM_420396_20110307		hypothetical protein					1				1			1					
JCVIGM_420773_20110307		hypothetical protein																	
JCVIGM_424310_20110307		hypothetical protein		1							1								
JCVIGM_434782_20110307		hypothetical protein																	
JCVIGM_441424_20110307		hypothetical protein													1				
JCVIGM_444308_20110307		hypothetical protein																	
JCVIGM_444309_20110307		hypothetical protein		1															
JCVIGM_055829_20110307		hypothetical protein BACCOPRO_02435																	
JCVIGM_158091_20110307		hypothetical protein BACCOPRO_03643					2				2	1							
JCVIGM_068437_20110307		hypothetical protein CLOST_1033						1								1			
JCVIGM_442772_20110307		hypothetical protein ELI_0537																	
JCVIGM_010811_20110307		inosine guanosine and xanthosine phosphorylase family		1			1	2	1		1	1							
JCVIGM_403964_20110307		inosine guanosine and xanthosine phosphorylase family protein																	
JCVIGM_054853_20110307	guaB	inosine-5'-monophosphate dehydrogenase																1	
JCVIGM_092237_20110307		inosine-5'-monophosphate dehydrogenase																	
JCVIGM_126259_20110307	guaB	inosine-5'-monophosphate dehydrogenase																1	
JCVIGM_196447_20110307		inosine-5'-monophosphate dehydrogenase							2										

JCVIGM_255764_20110307		inosine-5'-monophosphate dehydrogenase																	
JCVIGM_272653_20110307		inosine-5'-monophosphate dehydrogenase						1										1	
JCVIGM_328476_20110307		inosine-5'-monophosphate dehydrogenase						1				1		1					
JCVIGM_357305_20110307	iolG	inositol 2-dehydrogenase																	
JCVIGM_069994_20110307		inositol-3-phosphate synthase					1					1							
JCVIGM_091917_20110307		inositol-3-phosphate synthase																	
JCVIGM_184954_20110307		integral membrane sensor signal transduction histidine kinase																	
JCVIGM_065899_20110307		internalin-related protein		5		1	6	5	2		6	7		1	3			3	
JCVIGM_120727_20110307	ilvC	ketol-acid reductoisomerase																	
JCVIGM_156339_20110307	ilvC	ketol-acid reductoisomerase		2		2	1	1	2		2	1	1					1	
JCVIGM_185462_20110307	ilvC	ketol-acid reductoisomerase																	
JCVIGM_251867_20110307	ilvC	ketol-acid reductoisomerase																	
JCVIGM_287230_20110307	ilvC	ketol-acid reductoisomerase						2			1	1							
JCVIGM_322248_20110307	ilvC	ketol-acid reductoisomerase																	
JCVIGM_062252_20110307		ketose-bisphosphate aldolase, class II		1			1		1										
JCVIGM_128542_20110307		ketose-bisphosphate aldolase, class II									1	1							
JCVIGM_260030_20110307		KHG/KDPG family aldolase/carbohydrate kinase, pfkB family					2					1							
JCVIGM_418431_20110307		kinase, pfkB family		2					1		3	3						1	
JCVIGM_025581_20110307	araA	L-arabinose isomerase																	
JCVIGM_046489_20110307	araA	L-arabinose isomerase																	
JCVIGM_060257_20110307	araA	L-arabinose isomerase					1	9	5	1	2	2	3						
JCVIGM_073944_20110307	araA	L-arabinose isomerase		2	1	1	2	2	2	1	1	1	1	1				3	
JCVIGM_074178_20110307	araA	L-arabinose isomerase																	
JCVIGM_086747_20110307	araA	L-arabinose isomerase		3			1	1	1			1	1	1					
JCVIGM_088705_20110307	araA	L-arabinose isomerase																	
JCVIGM_168776_20110307	araA	L-arabinose isomerase																	
JCVIGM_206950_20110307	araA1	L-arabinose isomerase 1																	
JCVIGM_102423_20110307		L-asparaginase																	
JCVIGM_051778_20110307		L-asparaginase, type II																	

JCVIGM_177219_20110307		L-fucose isomerase and related proteins							1										
JCVIGM_022476_20110307	fucI	L-fucose isomerase, C-terminal domain																	
JCVIGM_059070_20110307		L-fucose isomerase, C-terminal domain		1															
JCVIGM_066045_20110307		L-fucose isomerase, C-terminal domain		1				1	1										
JCVIGM_101032_20110307		L-fucose isomerase, C-terminal domain		2			1				1							1	
JCVIGM_106686_20110307		L-fucose isomerase, C-terminal domain		1										1				2	
JCVIGM_124735_20110307		L-fucose isomerase, C-terminal domain																	
JCVIGM_187352_20110307		L-fucose isomerase, C-terminal domain		1				2	1		1	2							
JCVIGM_278994_20110307		L-fucose isomerase, C-terminal domain																	
JCVIGM_325641_20110307		L-fucose isomerase-like protein																	
JCVIGM_171365_20110307		lipoprotein																	
JCVIGM_083497_20110307	ldh	L-lactate dehydrogenase								1			1	1					
JCVIGM_159477_20110307	dapL	LL-diaminopimelate aminotransferase																	
JCVIGM_130368_20110307		L-ribulose-5-phosphate 4-epimerase									1							1	
JCVIGM_060255_20110307	araD	L-ribulose-5-phosphate 4-epimerase araD		1					1									1	
JCVIGM_160693_20110307	araD	L-ribulose-5-phosphate 4-epimerase araD																	
JCVIGM_295794_20110307	araD	L-ribulose-5-phosphate 4-epimerase araD										2							
JCVIGM_000243_20110307	lysS	lysine--tRNA ligase																	
JCVIGM_161438_20110307		M18 family aminopeptidase																	
JCVIGM_203664_20110307		M18 family aminopeptidase																	
JCVIGM_229762_20110307		major outer membrane lipoprotein 1												2	6	2	5	5	
JCVIGM_006253_20110307	mdh	malate dehydrogenase																	
JCVIGM_012327_20110307	mdh	malate dehydrogenase		1			1	1			2			1				1	1
JCVIGM_017662_20110307		malate dehydrogenase																	
JCVIGM_026640_20110307	mdh	malate dehydrogenase																	
JCVIGM_028360_20110307	mdh	malate dehydrogenase		3			1	1			2	2						2	
JCVIGM_127124_20110307	mdh	malate dehydrogenase																	2
JCVIGM_229225_20110307	mdh	malate dehydrogenase																	
JCVIGM_079110_20110307		malate dehydrogenase (oxaloacetate-decarboxylating)					1												
JCVIGM_020629_20110307	mdh	malate dehydrogenase, NAD-dependent		1											1			1	

JCVIGM_007627_20110307		maltose-binding protein																	
JCVIGM_201419_20110307		maltose-binding protein		1	1			2	1	1	1	1	1					1	3
JCVIGM_173530_20110307		membrane protein, bmp family		1															
JCVIGM_186148_20110307		membrane protein, bmp family																	
JCVIGM_052677_20110307		metallo-beta-lactamase superfamily																	
JCVIGM_038699_20110307	metK	methionine adenosyltransferase																	
JCVIGM_046971_20110307	metK	methionine adenosyltransferase																	
JCVIGM_076017_20110307	metK	methionine adenosyltransferase																	
JCVIGM_211427_20110307		methylmalonate-semialdehyde dehydrogenase (acylating)																	
JCVIGM_014933_20110307	mmdA	methylmalonyl-coA decarboxylase		1							1								
JCVIGM_034121_20110307		methylmalonyl-coA mutase large subunit										1							
JCVIGM_255025_20110307		methylmalonyl-coA mutase large subunit																	
JCVIGM_082989_20110307		methylmalonyl-coA mutase N-terminal domain																	
JCVIGM_069236_20110307	mutA	methylmalonyl-coA mutase, small subunit		1															
JCVIGM_033013_20110307		microcompartments protein								1									
JCVIGM_083041_20110307		microcompartments protein		1			1												
JCVIGM_188817_20110307		monosaccharide ABC transporter substrate-binding protein, CUT2 family							1										
JCVIGM_039831_20110307		monosaccharide ABC transporter substrate-binding protein, CUT2 family																	
JCVIGM_192144_20110307		monosaccharide ABC transporter substrate-binding protein, CUT2 family						1	1			1							
JCVIGM_070547_20110307	purE	N5-carboxyaminoimidazole ribonucleotide mutase																	
JCVIGM_060713_20110307		N-acetylneuraminate lyase		1															
JCVIGM_233593_20110307	nuoI	NADH-quinone oxidoreductase subunit I																	
JCVIGM_057221_20110307		NADP-dependent malic enzyme		3															
JCVIGM_231459_20110307		NADPH-dependent butanol dehydrogenase							1										
JCVIGM_121448_20110307		nifU-related domain containing protein		1			1	2	1	2	1	1				1			
JCVIGM_028245_20110307		nitrate/sulfonate/bicarbonate ABC transporter, periplasmic nitrate/sulfonate/bicarbonate-binding protein														1			
JCVIGM_149307_20110307		nitrite/sulfite reductase, 4Fe-4S iron-sulfur cluster-binding domain protein																	
JCVIGM_243460_20110307		nitrogen regulation protein																	
JCVIGM_074550_20110307		nitroreductase family										1							
JCVIGM_142709_20110307		oligopeptide-binding protein OppA																	

JCVIGM_287642_20110307		oligopeptide-binding protein OppA		1					1	1									
JCVIGM_291730_20110307		oligopeptide-binding protein OppA																	
JCVIGM_320377_20110307		oligopeptide-binding protein OppA		1										1					
JCVIGM_030295_20110307		ompA family protein													1			1	
JCVIGM_021098_20110307	pyrF	orotidine 5'-phosphate decarboxylase																	
JCVIGM_069275_20110307		outer membrane protein		1			1					1							
JCVIGM_021757_20110307		oxaloacetate decarboxylase, alpha subunit		1															
JCVIGM_185159_20110307		oxidoreductase domain protein																	
JCVIGM_227464_20110307		oxidoreductase domain protein		1															
JCVIGM_292816_20110307		oxidoreductase domain protein																	
JCVIGM_098050_20110307		peptide/nickel ABC transporter substrate-binding protein																	
JCVIGM_051204_20110307		peptidyl-prolyl cis-trans isomerase																	
JCVIGM_077390_20110307		peptidyl-prolyl cis-trans isomerase																	
JCVIGM_089246_20110307		peptidyl-prolyl cis-trans isomerase					1												
JCVIGM_359878_20110307		periplasmic binding protein/lacI transcriptional regulator																	
JCVIGM_330291_20110307		periplasmic protein		1			1	1	1		2	4							
JCVIGM_352406_20110307		periplasmic protein		5	1		2	2	3	2	2	3		1				3	
JCVIGM_063156_20110307	pta	phosphate acetyltransferase		2			1	1			2							1	
JCVIGM_141522_20110307	pta	phosphate acetyltransferase		1			1		1		2	2							
JCVIGM_008179_20110307	pckA	phosphoenolpyruvate carboxykinase (ATP)		3		1	2	1	5		2	1		1				2	
JCVIGM_042402_20110307	pckA	phosphoenolpyruvate carboxykinase (ATP)							1		1	1							
JCVIGM_051034_20110307	pckA	phosphoenolpyruvate carboxykinase (ATP)														1			
JCVIGM_066626_20110307	pckA	phosphoenolpyruvate carboxykinase (ATP)		4	1		4		1		2	3	2	1	1			4	
JCVIGM_070718_20110307	pckA	phosphoenolpyruvate carboxykinase (ATP)		19	2	2	2	3	3	5	2	7	2	3	2			5	
JCVIGM_084152_20110307	pckA	phosphoenolpyruvate carboxykinase (ATP)																	
JCVIGM_094757_20110307	pckA	phosphoenolpyruvate carboxykinase (ATP)																	
JCVIGM_108298_20110307	pckA	phosphoenolpyruvate carboxykinase (ATP)						1	1										
JCVIGM_111820_20110307	pckA	phosphoenolpyruvate carboxykinase (ATP)					1												
JCVIGM_116732_20110307		phosphoenolpyruvate carboxykinase (ATP)		1		5	2	1	1	1	1		1	2	1	1			
JCVIGM_126450_20110307	pckA	phosphoenolpyruvate carboxykinase (ATP)			37		1		1			1			1				

JCVIGM_145745_20110307	pckA	phosphoenolpyruvate carboxykinase (ATP)																	
JCVIGM_157605_20110307	pckA	phosphoenolpyruvate carboxykinase (ATP)																	
JCVIGM_168405_20110307	pckA	phosphoenolpyruvate carboxykinase (ATP)																	
JCVIGM_170518_20110307	pckG	phosphoenolpyruvate carboxykinase (ATP)										1							
JCVIGM_186619_20110307	pckA	phosphoenolpyruvate carboxykinase (ATP)											1						
JCVIGM_188966_20110307	pckA	phosphoenolpyruvate carboxykinase (ATP)						1	1										
JCVIGM_200200_20110307	pckA	phosphoenolpyruvate carboxykinase (ATP)					1		1		1	1							
JCVIGM_212975_20110307		phosphoenolpyruvate carboxykinase (ATP)		2	61	2	11	10	9	1	13	7	1	4	2			10	1
JCVIGM_214913_20110307	pckA	phosphoenolpyruvate carboxykinase (ATP)										1							
JCVIGM_219457_20110307	pckA	phosphoenolpyruvate carboxykinase (ATP)					1												
JCVIGM_255519_20110307	pckA	phosphoenolpyruvate carboxykinase (ATP)		2								1							
JCVIGM_257324_20110307	pckA	phosphoenolpyruvate carboxykinase (ATP)					1				1								
JCVIGM_259940_20110307	pckG	phosphoenolpyruvate carboxykinase (ATP)							2			1							
JCVIGM_297711_20110307	pckA	phosphoenolpyruvate carboxykinase (ATP)																	
JCVIGM_315164_20110307	pckA	phosphoenolpyruvate carboxykinase (ATP)					1	1	1		2	1							
JCVIGM_315431_20110307	pckA	phosphoenolpyruvate carboxykinase (ATP)		2			2	3	6		5	4		1					
JCVIGM_423536_20110307		phosphoenolpyruvate carboxykinase (ATP)																	
JCVIGM_070299_20110307	pckA	phosphoenolpyruvate carboxykinase [ATP]		2			1	1				2							
JCVIGM_127286_20110307	pckA	phosphoenolpyruvate carboxykinase [ATP]					2	2											1
JCVIGM_216386_20110307	manL	phosphoenolpyruvate-dependent sugar phosphotransferase system EIIAB, probably mannose specific																	
JCVIGM_007589_20110307	pgk	phosphoglycerate kinase															5		
JCVIGM_015380_20110307	pgk	phosphoglycerate kinase																	
JCVIGM_027320_20110307	pgk	phosphoglycerate kinase																	
JCVIGM_061465_20110307	pgk	phosphoglycerate kinase																	
JCVIGM_079319_20110307	pgk	phosphoglycerate kinase						1											
JCVIGM_095819_20110307	pgk	phosphoglycerate kinase																	
JCVIGM_113192_20110307	pgk	phosphoglycerate kinase		5			1	2	3		1	1						2	
JCVIGM_122299_20110307	pgk	phosphoglycerate kinase																	
JCVIGM_122514_20110307	pgk	phosphoglycerate kinase															1		
JCVIGM_152568_20110307	pgk	phosphoglycerate kinase																	
JCVIGM_161540_20110307	pgk	phosphoglycerate kinase																	
JCVIGM_195337_20110307	pgk	phosphoglycerate kinase		1															

JCVIGM_234057_20110307	pgk	phosphoglycerate kinase																	
JCVIGM_239710_20110307	pgk	phosphoglycerate kinase																	
JCVIGM_240738_20110307	pgk	phosphoglycerate kinase			2		1		1		1	1							
JCVIGM_249205_20110307	pgk	phosphoglycerate kinase																	
JCVIGM_260432_20110307	pgk	phosphoglycerate kinase												4	1			4	
JCVIGM_260954_20110307	pgk	phosphoglycerate kinase													1	1	2	1	
JCVIGM_313871_20110307	pgk	phosphoglycerate kinase																	
JCVIGM_325494_20110307	pgk	phosphoglycerate kinase						2	1			1							
JCVIGM_329407_20110307	pgk	phosphoglycerate kinase		3			1	3	3		4						1	2	
JCVIGM_040605_20110307	eno	phosphopyruvate hydratase		2															
JCVIGM_042947_20110307	eno	phosphopyruvate hydratase		2			2	1		2		1	1	1	1				1
JCVIGM_045954_20110307	eno	phosphopyruvate hydratase		1		1	1				1	1			1				
JCVIGM_064103_20110307	eno	phosphopyruvate hydratase																	
JCVIGM_069773_20110307	eno	phosphopyruvate hydratase		2								1						1	
JCVIGM_117723_20110307	eno	phosphopyruvate hydratase		1		1	2			1									
JCVIGM_189834_20110307	eno	phosphopyruvate hydratase		1			1												
JCVIGM_223379_20110307	eno	phosphopyruvate hydratase					1	1	2										
JCVIGM_226543_20110307	eno	phosphopyruvate hydratase																	
JCVIGM_231374_20110307	eno	phosphopyruvate hydratase																	
JCVIGM_234175_20110307	eno	phosphopyruvate hydratase				1									1			1	
JCVIGM_015906_20110307	purD	phosphoribosylamine--glycine ligase																	
JCVIGM_074211_20110307	purD	phosphoribosylamine--glycine ligase										1							
JCVIGM_058619_20110307	purH	phosphoribosylaminoimidazolecarboxamide formyltransferase/IMP cyclohydrolase		2															
JCVIGM_089519_20110307	glgP1	phosphorylase																	
JCVIGM_091853_20110307	glgP1	phosphorylase																	
JCVIGM_250822_20110307		phosphorylase																	
JCVIGM_020564_20110307	glgP	phosphorylase, glycogen/starch/alpha-glucan family																	
JCVIGM_132125_20110307	serC	phosphoserine transaminase																	
JCVIGM_151788_20110307	serC	phosphoserine transaminase		1															

JCVIGM_254038_20110307	serC	phosphoserine transaminase																1	
JCVIGM_000744_20110307	pnp	polyribonucleotide nucleotidyltransferase																	
JCVIGM_049618_20110307	pnp	polyribonucleotide nucleotidyltransferase		1							1								
JCVIGM_156816_20110307	pnp	polyribonucleotide nucleotidyltransferase		2					2			1		1					
JCVIGM_177288_20110307	pnp	polyribonucleotide nucleotidyltransferase																	
JCVIGM_284176_20110307	pnp	polyribonucleotide nucleotidyltransferase					1							1					
JCVIGM_301236_20110307	pnp	polyribonucleotide nucleotidyltransferase																	
JCVIGM_384292_20110307	pnp	polyribonucleotide nucleotidyltransferase		1															
JCVIGM_415810_20110307	pnp	polyribonucleotide nucleotidyltransferase																	
JCVIGM_149742_20110307		possible dehydrogenase																	
JCVIGM_158818_20110307		predicted glycosylase																	
JCVIGM_094962_20110307	araN	probable arabinose-binding protein																	
JCVIGM_443198_20110307	gcvPA	probable glycine dehydrogenase [decarboxylating] subunit 1																	
JCVIGM_181243_20110307	tal	probable transaldolase																	
JCVIGM_217573_20110307	tal	probable transaldolase		2			1												
JCVIGM_305907_20110307	tal	probable transaldolase															1	1	
JCVIGM_106798_20110307		propanediol utilization protein pduL																	
JCVIGM_343769_20110307	pduD	propanediol utilization: dehydratase pduD																	
JCVIGM_030557_20110307		propionyl-coA carboxylase beta chain		1								1							
JCVIGM_070982_20110307		propionyl-coA carboxylase subunit beta																	
JCVIGM_076862_20110307		propionyl-coA carboxylase subunit beta		2															
JCVIGM_212940_20110307		propionyl-coA carboxylase subunit beta																1	
JCVIGM_244599_20110307	deoD	purine nucleoside phosphorylase deoD-type																1	
JCVIGM_014093_20110307		purine nucleoside phosphorylase I, inosine and guanosine-specific		1			3	1	1		1	2		2				1	
JCVIGM_019193_20110307		purine nucleoside phosphorylase I, inosine and guanosine-specific		2			1		1			1		1	1			1	
JCVIGM_033253_20110307		putative 6-phosphofructokinase																	
JCVIGM_128651_20110307		putative 6-phosphofructokinase							1										
JCVIGM_176874_20110307		putative 6-phosphofructokinase																	
JCVIGM_149395_20110307		putative bacterial extracellular solute-binding protein						1	1		1								
JCVIGM_215021_20110307		putative bacterial extracellular solute-binding protein																1	
JCVIGM_268677_20110307		putative bacterial extracellular solute-binding protein					1	1											

JCVIGM_273647_20110307		putative bacterial extracellular solute-binding protein																	
JCVIGM_286660_20110307		putative bacterial extracellular solute-binding protein					1	2			1							1	
JCVIGM_297731_20110307		putative bacterial extracellular solute-binding protein						2	1		4								
JCVIGM_417971_20110307		putative bacterial extracellular solute-binding protein																1	
JCVIGM_237233_20110307	ytfQ	putative D-ribose transport system periplasmic binding component																	
JCVIGM_192868_20110307		putative endoribonuclease L-PSP																	
JCVIGM_028951_20110307		putative esterase																	
JCVIGM_101895_20110307		putative fumarate reductase flavoprotein subunit												1	1	1			1
JCVIGM_010624_20110307		putative immunoreactive antigen PG33																	
JCVIGM_182718_20110307		putative IMP cyclohydrolase		3															
JCVIGM_071025_20110307		putative inositol-3-phosphate synthase																	
JCVIGM_106169_20110307		putative inositol-3-phosphate synthase																	
JCVIGM_240722_20110307		putative inositol-3-phosphate synthase										1							
JCVIGM_063721_20110307		putative lipoprotein																	
JCVIGM_094735_20110307		putative lipoprotein													1				
JCVIGM_105771_20110307		putative major outer membrane protein					1												
JCVIGM_070279_20110307	spoVG	putative septation protein spoVG																	
JCVIGM_178430_20110307		putative sugar ABC transporter, sugar-binding protein																	
JCVIGM_194741_20110307		putative uncharacterized protein (fragment)							1	1	3								
JCVIGM_000105_20110307	ppdK	pyruvate, phosphate dikinase																	
JCVIGM_013898_20110307	ppdK	pyruvate, phosphate dikinase																	
JCVIGM_026505_20110307	ppdK	pyruvate, phosphate dikinase		1							2								
JCVIGM_036153_20110307	ppdK	pyruvate, phosphate dikinase		1															
JCVIGM_066682_20110307	ppdK	pyruvate, phosphate dikinase																	
JCVIGM_109792_20110307	ppdK	pyruvate, phosphate dikinase		1														1	
JCVIGM_135990_20110307	ppdK	pyruvate, phosphate dikinase																	
JCVIGM_173329_20110307		pyruvate, phosphate dikinase																	
JCVIGM_213012_20110307	ppdK	pyruvate, phosphate dikinase																	
JCVIGM_213014_20110307	ppdK	pyruvate, phosphate dikinase																	
JCVIGM_277616_20110307		pyruvate, phosphate dikinase		4								1							
JCVIGM_335683_20110307		pyruvate/oxaloacetate carboxyltransferase							1		1								

JCVIGM_198898_20110307		pyruvate-formate lyase		6			2	5	4	3	6	5	1	1				5	1
JCVIGM_255194_20110307		regulator of chromosome condensation, RCC1 (fragment)																	
JCVIGM_036971_20110307	rplJ	ribosomal protein L10		2															
JCVIGM_042383_20110307	rplJ	ribosomal protein L10						1				1							
JCVIGM_071597_20110307	rplJ	ribosomal protein L10		1			2	1	2		1	1		1			1		
JCVIGM_092732_20110307	rplJ	ribosomal protein L10						1	2		1	1		3	2			5	
JCVIGM_131224_20110307	rplJ	ribosomal protein L10																	
JCVIGM_210248_20110307	rplJ	ribosomal protein L10		2		1	2	4	5	1		2		1	1	1	1	4	
JCVIGM_226856_20110307	rplJ	ribosomal protein L10												2	2	2		2	
JCVIGM_082097_20110307	rplK	ribosomal protein L11							1										
JCVIGM_084768_20110307	rplK	ribosomal protein L11							1										
JCVIGM_136348_20110307	rplK	ribosomal protein L11										1							
JCVIGM_257392_20110307	rplK	ribosomal protein L11																	
JCVIGM_077076_20110307	rplK	ribosomal protein L11, N-terminal domain		3				1	3			2						1	
JCVIGM_097373_20110307	rplK	ribosomal protein L11, N-terminal domain																	
JCVIGM_045961_20110307	rplB	ribosomal protein L2		1		1		1			1								
JCVIGM_119829_20110307	rplB	ribosomal protein L2																	
JCVIGM_193483_20110307	rplB	ribosomal protein L2																	
JCVIGM_159582_20110307	rplW	ribosomal protein L23																	
JCVIGM_006060_20110307	rplL	ribosomal protein L7/L12																	
JCVIGM_054869_20110307	rplL	ribosomal protein L7/L12																	
JCVIGM_156392_20110307	rplL	ribosomal protein L7/L12					1	2											
JCVIGM_159116_20110307	rplL	ribosomal protein L7/L12		3								1							
JCVIGM_163260_20110307	rplL	ribosomal protein L7/L12																	
JCVIGM_327331_20110307	rplL	ribosomal protein L7/L12																	
JCVIGM_274754_20110307	rpsJ	ribosomal protein S10				1			1										
JCVIGM_021052_20110307	rpsG	ribosomal protein S7									1								
JCVIGM_089527_20110307	rpsH	ribosomal protein S8		1			2		1			2							
JCVIGM_106685_20110307	rpsH	ribosomal protein S8		2			2		1			1							
JCVIGM_116015_20110307	rpsH	ribosomal protein S8																	
JCVIGM_158733_20110307	rpsH	ribosomal protein S8									1								

JCVIGM_202567_20110307	rpsH	ribosomal protein S8				1													
JCVIGM_216187_20110307	rpsH	ribosomal protein S8				1		1											
JCVIGM_114301_20110307	rpsI	ribosomal protein S9/S16																	
JCVIGM_393651_20110307	rpoD	RNA polymerase sigma factor												1					
JCVIGM_229570_20110307		RNA polymerase sigma factor, sigma-70 family																	
JCVIGM_007913_20110307		rubredoxin																	
JCVIGM_057213_20110307		rubredoxin									1								
JCVIGM_006262_20110307		rubredoxin/rubrerythrin																	
JCVIGM_020560_20110307		rubredoxin/rubrerythrin		48	107	3	51	41	41	21	46	53	36	9	1	3	1	28	14
JCVIGM_060719_20110307		rubredoxin/rubrerythrin		3	73	1	7	29	27	1	14	41	1	13	2			18	
JCVIGM_085243_20110307		rubredoxin/rubrerythrin		1									1						
JCVIGM_248144_20110307		rubredoxin/rubrerythrin		1	1		1	5	3	1	2	2	1		1			1	
JCVIGM_401158_20110307		rubredoxin/rubrerythrin			2			2			1								
JCVIGM_415862_20110307		rubredoxin/rubrerythrin																	
JCVIGM_054682_20110307	rbr	rubrerythrin									1	1		1	1			1	
JCVIGM_073521_20110307		rubrerythrin					1												
JCVIGM_091745_20110307		rubrerythrin					1				1	1							
JCVIGM_119026_20110307		rubrerythrin		1				3	1		1	3	1					2	1
JCVIGM_127859_20110307		rubrerythrin																	1
JCVIGM_283228_20110307		rubrerythrin																	
JCVIGM_027483_20110307	metK	S-adenosylmethionine synthetase, N-terminal domain							1										
JCVIGM_031546_20110307		seryl-tRNA synthetase N-terminal domain										1							
JCVIGM_093445_20110307		short-chain dehydrogenase/reductase SDR																1	
JCVIGM_318607_20110307	ssb	single-strand binding protein							1										
JCVIGM_437080_20110307		single-stranded DNA-binding protein																	
JCVIGM_166571_20110307		smr domain protein																	
JCVIGM_117941_20110307	luxS	S-ribosylhomocysteine lyase																	
JCVIGM_158998_20110307		SSU ribosomal protein S1P																	
JCVIGM_034245_20110307		subtilase family					1								1				
JCVIGM_125149_20110307		subtilase family domain protein		1					1									1	
JCVIGM_007432_20110307		succinate CoA transferase																	

JCVIGM_079148_20110307		sugar ABC transporter substrate-binding protein				1	1	1				1						1	
JCVIGM_091077_20110307		sugar ABC transporter substrate-binding protein																	
JCVIGM_166102_20110307		sugar ABC transporter substrate-binding protein																	
JCVIGM_291360_20110307		sugar ABC transporter substrate-binding protein																	
JCVIGM_400559_20110307		sugar ABC transporter substrate-binding protein																	
JCVIGM_412377_20110307		sugar ABC transporter, periplasmic sugar-binding protein																	
JCVIGM_371869_20110307		sugar ABC transporter, sugar-binding protein																	
JCVIGM_014671_20110307		sugar phosphate isomerases/epimerases							2			1		1				2	
JCVIGM_084352_20110307		superoxide dismutase																	
JCVIGM_070610_20110307		susD family																	
JCVIGM_102265_20110307		susD family		1				1			1	2							
JCVIGM_131087_20110307		tetratricopeptide repeat protein																	
JCVIGM_391275_20110307	thlA3	thlA3																	
JCVIGM_070609_20110307		tonB dependent receptor																	
JCVIGM_081124_20110307		tonB-dependent receptor plug domain protein																	
JCVIGM_134744_20110307		tonB-dependent receptor plug domain protein																	
JCVIGM_052711_20110307		tonB-linked outer membrane protein, susC/ragA family																	
JCVIGM_010680_20110307	fusA	translation elongation factor G																	
JCVIGM_023291_20110307	fusA	translation elongation factor G																	
JCVIGM_078720_20110307	fusA	translation elongation factor G									1								
JCVIGM_105810_20110307	fusA	translation elongation factor G																	
JCVIGM_112572_20110307	fusA	translation elongation factor G																	
JCVIGM_143844_20110307	fusA	translation elongation factor G		1					3			2							
JCVIGM_097131_20110307	tsf	translation elongation factor Ts																	
JCVIGM_183571_20110307	tsf	translation elongation factor Ts																	
JCVIGM_277838_20110307	tsf	translation elongation factor Ts					1				1								
JCVIGM_431442_20110307	tsf	translation elongation factor Ts						1											
JCVIGM_026960_20110307	tuf	translation elongation factor Tu		8	3	1	2			1	5	2	2		1	1	1	2	2
JCVIGM_029795_20110307	tuf	translation elongation factor Tu		15			2	4	3		3	3		1	1			3	
JCVIGM_109601_20110307	tuf	translation elongation factor Tu																	
JCVIGM_124820_20110307	tuf	translation elongation factor Tu		14	1	1	10	10	13	2	7	5	1	8	4	1	1	3	
JCVIGM_157947_20110307	tuf	translation elongation factor Tu																	

JCVIGM_166526_20110307	tufB	translation elongation factor Tu																	
JCVIGM_185454_20110307	tuf	translation elongation factor Tu									1								
JCVIGM_196046_20110307	tuf	translation elongation factor Tu																	
JCVIGM_196200_20110307	tuf	translation elongation factor Tu		2			1												
JCVIGM_205201_20110307	tuf	translation elongation factor Tu																	
JCVIGM_211136_20110307	tuf	translation elongation factor Tu																	
JCVIGM_237573_20110307	tuf	translation elongation factor Tu		1															
JCVIGM_249773_20110307	tuf	translation elongation factor Tu																	
JCVIGM_250936_20110307	tufB	translation elongation factor Tu													1			1	
JCVIGM_300774_20110307	tuf	translation elongation factor Tu																	
JCVIGM_368873_20110307	tuf	translation elongation factor Tu																	
JCVIGM_385355_20110307		transposase, IS111A/IS1328/IS1533		2															
JCVIGM_364669_20110307		TRAP transporter solute receptor, TAXI family																	
JCVIGM_001774_20110307	tpiA	triose-phosphate isomerase		2		1	2	1	1		1	2		1				1	
JCVIGM_008332_20110307	tpiA	triose-phosphate isomerase		2		1	2	2	2		2						1		
JCVIGM_011008_20110307	tpiA	triose-phosphate isomerase		1			1												
JCVIGM_020800_20110307	tpiA	triose-phosphate isomerase			1										1				
JCVIGM_023613_20110307	tpiA	triose-phosphate isomerase									1	1							
JCVIGM_027321_20110307	tpiA	triose-phosphate isomerase					1	1	2		2	2							
JCVIGM_087250_20110307	tpiA	triose-phosphate isomerase		2			3	1	1		2	2		1				2	
JCVIGM_115071_20110307	tpiA	triose-phosphate isomerase																	
JCVIGM_135680_20110307	tpiA	triose-phosphate isomerase		1			1				2	1							
JCVIGM_261662_20110307	tpiA	triose-phosphate isomerase							1		1	1							
JCVIGM_302433_20110307	tpiA	triose-phosphate isomerase												1				2	
JCVIGM_337367_20110307	tpiA	triose-phosphate isomerase		1			1	1	1		1			1					
JCVIGM_366477_20110307	tpiA	triose-phosphate isomerase																	
JCVIGM_114128_20110307		tRNA methyl transferase																	
JCVIGM_117733_20110307		two-component system sensor histidine kinase, with a response regulator receiver domain																	
JCVIGM_373409_20110307		type III restriction protein												1					

JCVIGM_014974_20110307	galE	UDP-glucose 4-epimerase					1												
JCVIGM_179048_20110307	murE	UDP-N-acetylmuramoyl-L-alanyl-D-glutamate--2,6-diaminopimelate ligase																	
JCVIGM_240853_20110307	uspA	universal stress protein A																1	
JCVIGM_311901_20110307		UPF0210 protein DORLON_00661																	
JCVIGM_044460_20110307		UTP--hexose-1-phosphate uridylyltransferase		2			1	2			1	1						1	
JCVIGM_148949_20110307	pepD	Xaa-His dipeptidase																	
JCVIGM_195935_20110307		xylose ABC transporter substrate-binding protein										1							
JCVIGM_086248_20110307	xylA	xylose isomerase																	
JCVIGM_113241_20110307	xylA	xylose isomerase																	
JCVIGM_120909_20110307	xylA	xylose isomerase																	
JCVIGM_229108_20110307		YjeF N-terminal domain protein																	

				**Biological replicate 2-b**															
				CONTROL								INFECTED							
Reference	Gene symbol	Protein description	DAY ->	-1	1	3	6	10	14	21	28	-1	1	3	6	10	14	21	28
JCVIGM_070246_20110307	gpmI	2,3-bisphosphoglycerate-independent phosphoglycerate mutase		1		1	1	1	4		4	1							
JCVIGM_091222_20110307	gpmI	2,3-bisphosphoglycerate-independent phosphoglycerate mutase																	
JCVIGM_199914_20110307		2-dehydro-3-deoxygluconokinase		1	1						2							4	
JCVIGM_044539_20110307	eda	2-dehydro-3-deoxyphosphogluconate aldolase/4-hydroxy-2-oxoglutarate aldolase		2			2	1		1	2	1						2	1
JCVIGM_057836_20110307	eda	2-dehydro-3-deoxyphosphogluconate aldolase/4-hydroxy-2-oxoglutarate aldolase		10	11			1	2	27									
JCVIGM_092015_20110307	eda	2-dehydro-3-deoxyphosphogluconate aldolase/4-hydroxy-2-oxoglutarate aldolase			2					2	2	2		1	1	1	1		2
JCVIGM_360038_20110307	yhaE	2-hydroxy-3-oxopropionate reductase												2		1	1	1	
JCVIGM_374955_20110307		2-hydroxy-3-oxopropionate reductase																	
JCVIGM_319522_20110307	kdsA	3-deoxy-8-phosphooctulonate synthase			1													1	
JCVIGM_196595_20110307		3-hydroxyacyl-coA dehydrogenase		11	12	19	7	3	5	29	14	22	11	9		2		6	2
JCVIGM_037673_20110307	hbd	3-hydroxybutyryl-coA dehydrogenase		1		1	1	1	1	2	1		2						
JCVIGM_037995_20110307	pdxB	4-phosphoerythronate dehydrogenase		1	1		2	3	1	1	1	2	1			1			
JCVIGM_259097_20110307	rplJ	50S ribosomal protein L10						1	1		2								

JCVIGM_024389_20110307	rplK	50S ribosomal protein L11							1				1						
JCVIGM_045877_20110307	rplK	50S ribosomal protein L11		1	2						2	2	1	2	1	1	1	2	
JCVIGM_059265_20110307	rplK	50S ribosomal protein L11		3	3	1	3		2	2	2	2	3	3	1	2	2		3
JCVIGM_033470_20110307	rplE	50S ribosomal protein L5					1		1									1	
JCVIGM_155746_20110307	rplE	50S ribosomal protein L5						1											
JCVIGM_232804_20110307	rplE	50S ribosomal protein L5						1											
JCVIGM_020270_20110307	rplL	50S ribosomal protein L7/L12		2	2	1						2	8	4		3	2	3	2
JCVIGM_125249_20110307	rplL	50S ribosomal protein L7/L12		2	3	1			3		3		5						
JCVIGM_100458_20110307	ribH	6,7-dimethyl-8-ribityllumazine synthase			2														
JCVIGM_013177_20110307		60 kDa chaperonin				1	1					1							
JCVIGM_213822_20110307		60 kDa chaperonin																	
JCVIGM_123049_20110307		ABC transporter periplasmic-binding protein ytfQ		1			1	1			1	1					1		
JCVIGM_295655_20110307		ABC transporter related																	
JCVIGM_097008_20110307		ABC transporter, ATP-binding protein		1											1				
JCVIGM_168201_20110307		ABC transporter, ATP-binding protein				2													
JCVIGM_070783_20110307		ABC transporter, permease protein										2							
JCVIGM_179750_20110307		ABC transporter, substrate-binding protein		2	2														
JCVIGM_284177_20110307		ABC transporter, substrate-binding protein, family 1			2		3						1						
JCVIGM_284885_20110307		ABC-type dipeptide transport system, periplasmic component		1									1						
JCVIGM_168588_20110307		ABC-type sugar transport system, periplasmic component			2	1	3	1				2							
JCVIGM_297706_20110307		ABC-type sugar transport system, periplasmic component					1	6	3	10	6	3						2	
JCVIGM_162628_20110307		ABC-type transport system, substrate-binding component					1	1	1										1
JCVIGM_197130_20110307		ABC-type xylose transport system, periplasmic component		4	2	1	4	1	5	2	5	4	1					1	
JCVIGM_034585_20110307	ackA	acetate kinase		1			1	2	1		1	1	1						
JCVIGM_090369_20110307	ackA	acetate kinase		3															
JCVIGM_103872_20110307	ackA	acetate kinase						1											
JCVIGM_105479_20110307	ackA	acetate kinase								1		1							
JCVIGM_313779_20110307	ackA	acetate kinase					1	1				1							
JCVIGM_120726_20110307	ilvC	acetohydroxy acid isomeroreductase, catalytic domain		1	1		1	2	1	2	2	1	2					1	
JCVIGM_136868_20110307	ilvC	acetohydroxy acid isomeroreductase, catalytic domain		12	13	15	15	14	15	14	13	12	12	10	1	5	3	5	4

JCVIGM_141542_20110307	ilvC	acetohydroxy acid isomeroreductase, catalytic domain		4	5	1	4	4	4	1	4	3	4	2		1	1	3	1
JCVIGM_256491_20110307	ilvC	acetohydroxy acid isomeroreductase, catalytic domain		2			1					2			1		2		1
JCVIGM_298658_20110307	ilvC	acetohydroxy acid isomeroreductase, catalytic domain		1	5	4	4	4	5	1	3	2	10	2				1	1
JCVIGM_383122_20110307	ilvC	acetohydroxy acid isomeroreductase, catalytic domain		5	5	2	3	6	4	2	3	2	5	1		1	1	2	
JCVIGM_029818_20110307	ilvN	acetolactate synthase, small subunit							1	1									
JCVIGM_125179_20110307		acetyl-coA acetyltransferases		1			1	4	2										
JCVIGM_101296_20110307		acetyl-coA C-acetyltransferase				1		2	1		1	2	2						
JCVIGM_182295_20110307		acetyl-coA C-acetyltransferase		4	5	2	5	8	4	2	7	10	9	1	2			6	
JCVIGM_234799_20110307		acetyl-coA C-acetyltransferase		8	1	2	5	12	12		11	4	2	1				11	
JCVIGM_306189_20110307	phaA	acetyl-CoA C-acetyltransferase		2		1				1	1	2	3	1					
JCVIGM_299975_20110307		acyltransferase family																	1
JCVIGM_058622_20110307	purA	adenylosuccinate synthase		4								1							1
JCVIGM_064826_20110307	purA	adenylosuccinate synthase			1	1													
JCVIGM_092236_20110307	purA	adenylosuccinate synthase		1								1							
JCVIGM_092238_20110307	purA	adenylosuccinate synthase		2															
JCVIGM_108189_20110307		ahpC/TSA family																	1
JCVIGM_145159_20110307	ald	alanine dehydrogenase		1														1	1
JCVIGM_391701_20110307	tdh	alcohol dehydrogenase groES domain protein																	
JCVIGM_144742_20110307		alcohol dehydrogenase, iron-dependent													2				
JCVIGM_147392_20110307		aldehyde oxidase and xanthine dehydrogenase molybdopterin binding protein		2															
JCVIGM_050239_20110307		alpha-amylase		3															
JCVIGM_248140_20110307		alpha-D-mannosidase			1									1					
JCVIGM_337826_20110307		alpha-D-mannosidase			2		1	2	1	1	1	2	1					2	1
JCVIGM_358636_20110307		alpha-D-mannosidase			3			1	1				4					2	
JCVIGM_076247_20110307		alpha-galactosidase				1	1	1	1		2							1	
JCVIGM_090969_20110307		alpha-galactosidase				1	1												
JCVIGM_235809_20110307		alpha-galactosidase		1														3	
JCVIGM_094959_20110307		alpha-N-arabinofuranosidase		4															
JCVIGM_179557_20110307		alpha-N-arabinofuranosidase		5															

JCVIGM_043430_20110307		arabinogalactan endo-1,4-beta-galactosidase				1													
JCVIGM_054434_20110307		arabinogalactan endo-1,4-beta-galactosidase		1		1	1				1								
JCVIGM_030203_20110307	fucI	arabinose isomerase			1		1		2	1		1	4						
JCVIGM_108898_20110307	fucI	arabinose isomerase		1	3			1	1	2	2	1	8	1		1	1	2	
JCVIGM_151443_20110307	fucI	arabinose isomerase			3				1				1						
JCVIGM_188730_20110307	fucI	arabinose isomerase			2		1	1	1	1	1	2	4			1		1	
JCVIGM_191949_20110307	fucI	arabinose isomerase			1		2				1	1	1						
JCVIGM_339907_20110307	fucI	arabinose isomerase						1	1										
JCVIGM_341670_20110307	fucI	arabinose isomerase							1										
JCVIGM_019632_20110307	arcA	arginine deiminase			1						1		1			1		1	
JCVIGM_030954_20110307	asnB	asparagine synthase (glutamine-hydrolyzing)																	
JCVIGM_149591_20110307	aspA	aspartate ammonia-lyase													2			1	
JCVIGM_076159_20110307	asd	aspartate-semialdehyde dehydrogenase																	
JCVIGM_163401_20110307	asd	aspartate-semialdehyde dehydrogenase					1		1			1							1
JCVIGM_146114_20110307	aspS	aspartate--tRNA ligase													2			2	
JCVIGM_225435_20110307	atpA	ATP synthase F1, alpha subunit								5									
JCVIGM_193732_20110307		ATP synthase subunit a												1					1
JCVIGM_181280_20110307		B12 binding domain		1			1	2	2		1								
JCVIGM_335931_20110307		basic membrane lipoprotein			1								1						
JCVIGM_191196_20110307		beta-galactosidase		1	1	1		2	1	1	1							2	
JCVIGM_065702_20110307	bglA	beta-glucosidase A									2							1	
JCVIGM_026370_20110307		beta-glucosidase-n-terminal domain-containing protein		2				2											
JCVIGM_156507_20110307		beta-lactamase									2								
JCVIGM_042339_20110307	purH	bifunctional purine biosynthesis protein purH										1							
JCVIGM_036791_20110307	buk	butyrate kinase		2						2	5							1	
JCVIGM_216035_20110307		butyryl-coA dehydrogenase					1	2	1			1						1	
JCVIGM_265820_20110307		butyryl-coA dehydrogenase		6	4	4	6	4	7	4	9	7	5	2				1	1
JCVIGM_290061_20110307		butyryl-coA dehydrogenase		1		1	2		1	1	1	2	1					1	
JCVIGM_329946_20110307	bcd2	butyryl-coA dehydrogenase		1	1						1								
JCVIGM_040543_20110307	c7d2-bg	C7D2-BG protein		4	4		2	1	2			1	1						
JCVIGM_017812_20110307		carbohydrate ABC transporter substrate-binding protein, CUT1 family		2															

JCVIGM_055645_20110307		carbohydrate ABC transporter substrate-binding protein, CUT1 family						1		1	1								
JCVIGM_306701_20110307		carbohydrate ABC transporter substrate-binding protein, CUT1 family					2	3	2	3	4	2	4					2	3
JCVIGM_074670_20110307		carbohydrate ABC transporter substrate-binding protein, CUT1 family		23															
JCVIGM_324855_20110307		carbohydrate ABC transporter, carbohydrate-binding protein		4	2	1	2	5	2		3	2	12					1	
JCVIGM_049249_20110307	cooS	carbon-monoxide dehydrogenase, catalytic subunit		19	3	1	1	1	2	1	2	2	1	1				2	
JCVIGM_000407_20110307	dnaK	chaperone protein dnaK		1		1	2												1
JCVIGM_021700_20110307	dnaK	chaperone protein dnaK		2			3			1		1			1				
JCVIGM_031425_20110307	dnaK	chaperone protein dnaK		2		1	1			2	1	1			1	1		1	2
JCVIGM_032528_20110307	dnaK	chaperone protein dnaK		1		2	1				1	1		1	2	1		1	2
JCVIGM_034511_20110307	dnaK	chaperone protein dnaK		1			1												
JCVIGM_043298_20110307	dnaK	chaperone protein dnaK					1			1		1							
JCVIGM_062282_20110307	dnaK	chaperone protein dnaK																	
JCVIGM_122947_20110307	dnaK	chaperone protein dnaK		2			1	1			1	1							
JCVIGM_147511_20110307	dnaK	chaperone protein dnaK			1				1				1	1					
JCVIGM_213960_20110307	dnaK	chaperone protein dnaK				1	1	1		1									
JCVIGM_236768_20110307	dnaK	chaperone protein dnaK		2			3								2			1	
JCVIGM_086927_20110307	groS	chaperonin 10 Kd subunit		1	2		1	2	4	2	4	3		1		1		2	2
JCVIGM_187049_20110307	groS	chaperonin 10 Kd subunit		7				1					1						
JCVIGM_213821_20110307	groS	chaperonin 10 Kd subunit			1		1	1			1	1		1					
JCVIGM_401604_20110307	groS	chaperonin 10 Kd subunit			1														
JCVIGM_413734_20110307		CO dehydrogenase/acetyl-coA synthase complex, beta subunit		1		1	5	5	3	1	4	1						2	
JCVIGM_049251_20110307	cdhC	CO dehydrogenase/CO-methylating acetyl-coA synthase complex, beta subunit		17	5		6	1	13		1	1							
JCVIGM_339054_20110307		coenzyme B12-dependent glycerol dehydratase, large subunit			3			2	1										
JCVIGM_212787_20110307		cof-like hydrolase				1		1											
JCVIGM_127608_20110307		ComEC/Rec2-like protein						1											
JCVIGM_286641_20110307		conserved hypothetical protein			1								2						
JCVIGM_049253_20110307		corrinoid/iron-sulfur protein, large subunit		1			2	3	1		1	1							
JCVIGM_049252_20110307		corrinoid/iron-sulfur protein, small subunit		1	2			1		1									
JCVIGM_022873_20110307	cysK	cysteine synthase A																	

JCVIGM_307840_20110307		dehydrogenase, FMN-dependent																	
JCVIGM_102159_20110307	deoC	deoxyribose-phosphate aldolase		9		5	6	4	7	1	6	4	1	1	8	1	1	4	2
JCVIGM_056340_20110307		diaminopimelate dehydrogenase		3			2							1	1				1
JCVIGM_104884_20110307		diaminopimelate dehydrogenase		1	1	1	2	2	3	2	2	2	1			1		2	
JCVIGM_002401_20110307	lpdA	dihydrolipoyl dehydrogenase						1								1			
JCVIGM_139913_20110307	pyrD	dihydroorotate oxidase		1			1	1	2	1	2	1							
JCVIGM_049254_20110307		dihydropteroate synthase, DHPS		7	2								2						
JCVIGM_130560_20110307	ilvD	dihydroxy-acid dehydratase							1		1								
JCVIGM_377265_20110307	disA	DNA integrity scanning protein disA		2			1				1	1							2
JCVIGM_176486_20110307	dnaN	DNA polymerase III, beta subunit																	
JCVIGM_228057_20110307	etfB	electron transfer flavoprotein, alpha subunit				2		2	3										
JCVIGM_310193_20110307	etfB	electron transfer flavoprotein, alpha subunit		1					1		2	1							
JCVIGM_316234_20110307	etfA	electron transfer flavoprotein, alpha subunit/fixB family protein		1				1											
JCVIGM_083913_20110307	etfB	electron transfer flavoprotein, beta subunit		7	1		1	4	4	2	4	2							
JCVIGM_215068_20110307	etfB	electron transfer flavoprotein, beta subunit				1		1	2	1	2			1					
JCVIGM_316431_20110307		electron transfer flavoprotein, beta subunit		3	1	5	1		4		2								
JCVIGM_041452_20110307	tuf	elongation factor Tu (fragment)				1					2								
JCVIGM_148583_20110307		elongation factor Tu (fragment)								1							1		
JCVIGM_209439_20110307	tuf	elongation factor Tu (fragment)		1			1		1	1		1			1	1		1	1
JCVIGM_239217_20110307	tuf1	elongation factor Tu 1															3		
JCVIGM_045263_20110307		extracellular solute-binding protein family 1			2	1	5	3	2	3	3	2	3					1	1
JCVIGM_088711_20110307		extracellular solute-binding protein family 1		7															
JCVIGM_096968_20110307		extracellular solute-binding protein family 1			2	1	3	2					1						
JCVIGM_108783_20110307		extracellular solute-binding protein family 1		4															
JCVIGM_212868_20110307		extracellular solute-binding protein family 1					1	1											
JCVIGM_275107_20110307		extracellular solute-binding protein family 1									1	1							
JCVIGM_291167_20110307		extracellular solute-binding protein family 1		1	2		2	2	3	3	5	3	3	1		1		1	
JCVIGM_192080_20110307		extracellular solute-binding protein family 5		1															
JCVIGM_259518_20110307		ferritin 1																2	
JCVIGM_308648_20110307		fibronectin type III domain			1								1						
JCVIGM_400677_20110307		fla1 flagellin		1	1	1	1	3	4	2	2	3		3	3	4	4	1	

JCVIGM_423664_20110307		fla1 flagellin (fragment)					1	7	3									2	
JCVIGM_430871_20110307		fla3 flagellin		4	3	6	5	8	8	1	10	8	4	7			1	3	2
JCVIGM_416087_20110307	flgE1	flagellar hook protein flgE1									1							1	
JCVIGM_077354_20110307		flagellin (fragment)		4	1	1	2	6	3	8	3	4		4	1	7	4	3	3
JCVIGM_172838_20110307		flagellin (fragment)		1			1			2	3	1							1
JCVIGM_022208_20110307		flagellin and related hook-associated proteins		8	4	3	9	9	8	3	10	10	6	9	6	10	7	8	7
JCVIGM_154194_20110307		flagellin and related hook-associated proteins															1	1	
JCVIGM_190042_20110307		flagellin and related hook-associated proteins		1	1	2	1	2	3		3								
JCVIGM_389298_20110307		flagellin and related hook-associated proteins				1	1	2										1	
JCVIGM_188452_20110307		flagellin domain protein		6	3	6	11	19	24	3	9	5	13	7	2	2		5	1
JCVIGM_346020_20110307		flagellin domain protein		2		1	4	5	5									1	
JCVIGM_391229_20110307		flagellin domain protein											2					2	
JCVIGM_069861_20110307	fliC1	flagellin fliC1		1		2	3	3	3	1	5	1						2	
JCVIGM_410616_20110307	fliC1	flagellin fliC1		6	1	5	6	11	7		4	4	3	3	1			3	
JCVIGM_152287_20110307	fliC2	flagellin fliC2		2	1		2	2	2	3		1	1	2		2	2	2	
JCVIGM_177955_20110307	fliC2	flagellin fliC2				3	2	3	3		1								
JCVIGM_262501_20110307	fliC2	flagellin fliC2																	
JCVIGM_187473_20110307	fliC4	flagellin fliC4		1			2		1				1	2				2	
JCVIGM_189905_20110307	fliC5	flagellin fliC5						1	2	1						4	2		
JCVIGM_392640_20110307		flagellin protein		1				1	1								2		
JCVIGM_402856_20110307		flagellin protein		1	1				1								1		
JCVIGM_414958_20110307		flagellin protein																	1
JCVIGM_424682_20110307		flagellin protein						1			1								
JCVIGM_152365_20110307	fprA	flavodoxin									2	1							
JCVIGM_148596_20110307		formate acetyltransferase (fragment)		1	1			1			3	1	2					3	
JCVIGM_185080_20110307		formate acetyltransferase (fragment)			6			1		1	1	2	8						
JCVIGM_246004_20110307		formate acetyltransferase (fragment)			2	1		2	1										
JCVIGM_032907_20110307	pflB	formate C-acetyltransferase		4	4	2	3	4	3	4	4	6	4	2		2	1	4	2
JCVIGM_043008_20110307	pflB	formate C-acetyltransferase		6	3		6	9	9	6	10	10	8	5	1	1	1	9	7
JCVIGM_157210_20110307		formate C-acetyltransferase				1					1			2					
JCVIGM_229801_20110307		formate C-acetyltransferase		2	1		1	1	2		2							1	1

JCVIGM_249731_20110307		formate C-acetyltransferase		11	7	4	13	31	31		17		7					7	
JCVIGM_282796_20110307		formate C-acetyltransferase			2		1			1		2	1			1			
JCVIGM_317542_20110307		formate C-acetyltransferase		2	3	1		3	2	2	3	2	3	2		2		3	2
JCVIGM_356609_20110307		formate C-acetyltransferase		2	2			1		2		1	1	2			1		
JCVIGM_417381_20110307		formate C-acetyltransferase		1				1			1							1	
JCVIGM_078607_20110307	fhs	formate--tetrahydrofolate ligase			1			1											
JCVIGM_118473_20110307	fhs	formate--tetrahydrofolate ligase		4	3		3	1	1	1	1	2	3						
JCVIGM_123964_20110307	fhs	formate--tetrahydrofolate ligase		3	3	2	5	6	5	5	6	8	5	2	1	2	1	4	
JCVIGM_138264_20110307	fhs	formate--tetrahydrofolate ligase																	
JCVIGM_162244_20110307	fhs	formate--tetrahydrofolate ligase					1				1								
JCVIGM_176151_20110307	fhs	formate--tetrahydrofolate ligase																	
JCVIGM_245005_20110307	fhs	formate--tetrahydrofolate ligase		3	5	2	7	11	10	1	7	5	11	4				1	
JCVIGM_400807_20110307	fhs	formate--tetrahydrofolate ligase																	
JCVIGM_122704_20110307	fhs1	formate--tetrahydrofolate ligase 1									1								
JCVIGM_146104_20110307	fhs2	formate--tetrahydrofolate ligase 2		50	14	11	27	37	34	11	26	28	25	9	1			9	
JCVIGM_032208_20110307	frc	formyl-coA transferase				1		1			1								
JCVIGM_065785_20110307	frc	formyl-CoA transferase		2		1	1	2		1	2	2			1	2	1	1	2
JCVIGM_070965_20110307	frc	formyl-coA transferase		6		3	6	3	1	3	6	6			2	1		1	4
JCVIGM_132967_20110307	frc	formyl-coA transferase		8		6	11	5	4	3	6	5		1	5	3	1	6	4
JCVIGM_231668_20110307	frc	formyl-coA transferase		1											1				
JCVIGM_254826_20110307	frc	formyl-coA transferase		2															1
JCVIGM_051280_20110307		fructokinase																	
JCVIGM_079013_20110307		fructose-1,6-bisphosphate aldolase, class II																	
JCVIGM_087474_20110307	fba	fructose-1,6-bisphosphate aldolase, class II		4		3	7	19	19	7	16	9				1			
JCVIGM_118274_20110307	fba	fructose-1,6-bisphosphate aldolase, class II		3	1	1	4	6	6	1	2	6	2	1					
JCVIGM_000284_20110307	fsa	fructose-6-phosphate aldolase		1	1				1			1							
JCVIGM_153991_20110307	fsa	fructose-6-phosphate aldolase		7	4	1		1	2	1	3	2	3	1		1	1		
JCVIGM_393658_20110307	fsa	fructose-6-phosphate aldolase		10	4			9		1								1	
JCVIGM_231559_20110307		fructose-bisphosphate aldolase		2	1		2	2	2	3	2	4	1	2				1	
JCVIGM_089609_20110307		fructose-bisphosphate aldolase class-I		8															

JCVIGM_336730_20110307		galactose ABC transporter, periplasmic galactose-binding protein		1	1		2	2	1		2	2	3					1	
JCVIGM_027728_20110307		galactose/glucose-binding lipoprotein																	
JCVIGM_266085_20110307	galT	galactose-1-phosphate uridylyltransferase galT																1	
JCVIGM_165424_20110307		gliding motility-related protein			1						1	1	2						
JCVIGM_351864_20110307		glu/leu/phe/val dehydrogenase, dimerization domain protein									1		1						
JCVIGM_138714_20110307		gluconate 5-dehydrogenase							1		2		1					1	
JCVIGM_002867_20110307	pgi	glucose-6-phosphate isomerase			3		1	2	4	3	1	2	2	4		3	1	4	3
JCVIGM_015397_20110307	pgi	glucose-6-phosphate isomerase																	
JCVIGM_015745_20110307	pgi	glucose-6-phosphate isomerase		4	5	1	5	6	8	3	7	4	5	2		2	1	5	2
JCVIGM_026127_20110307	pgi	glucose-6-phosphate isomerase		1				1	1										
JCVIGM_030449_20110307	pgi	glucose-6-phosphate isomerase		7	11	10	7	16	9		8	3	10	1				8	
JCVIGM_042043_20110307	pgi	glucose-6-phosphate isomerase						1	1							1			
JCVIGM_054834_20110307	pgi	glucose-6-phosphate isomerase						1	1										
JCVIGM_073687_20110307	pgi	glucose-6-phosphate isomerase		1	1				1		1								
JCVIGM_075891_20110307	pgi	glucose-6-phosphate isomerase			1					2	2	1	1	1				3	
JCVIGM_080716_20110307	pgi	glucose-6-phosphate isomerase							1										
JCVIGM_091996_20110307	pgi	glucose-6-phosphate isomerase		3	6	5	6	8	11		1		6	1	1	1		2	
JCVIGM_116144_20110307	pgi	glucose-6-phosphate isomerase		1	1		2				1								
JCVIGM_147397_20110307	pgi	glucose-6-phosphate isomerase		18	9	3		11	5		1		1		1			16	
JCVIGM_150388_20110307	pgi	glucose-6-phosphate isomerase		3	1														1
JCVIGM_154594_20110307	pgi	glucose-6-phosphate isomerase		15	20	10	9	19	20	15	19	14	10	7	5	6	1	12	
JCVIGM_170286_20110307	pgi	glucose-6-phosphate isomerase		17		17	18	6	10	3	10	8		1	14	1		8	8
JCVIGM_208717_20110307	pgi	glucose-6-phosphate isomerase																	
JCVIGM_221773_20110307	pgi	glucose-6-phosphate isomerase		5	2	3	2	4	3		4	1	3	1	3	1	2	1	
JCVIGM_229071_20110307		glucose-6-phosphate isomerase		5	4	15	8	17	15	4	11	8	6	4	4	4	1	5	1
JCVIGM_245845_20110307	pgi	glucose-6-phosphate isomerase									1							1	
JCVIGM_257476_20110307	pgi	glucose-6-phosphate isomerase		4	2	1	1	1	2	3	2	5						3	
JCVIGM_261055_20110307	pgi	glucose-6-phosphate isomerase								2									
JCVIGM_286986_20110307	pgi	glucose-6-phosphate isomerase		7	4	3	7	12	13	3	13	7	7	3				4	3
JCVIGM_296222_20110307	pgi	glucose-6-phosphate isomerase		3	5	3	3	7	4		1	1	4	1				4	

JCVIGM_296223_20110307	pgi	glucose-6-phosphate isomerase							1			1							
JCVIGM_346043_20110307	pgi	glucose-6-phosphate isomerase				1						1							
JCVIGM_407434_20110307	pgi	glucose-6-phosphate isomerase		8	6		8	12	6	11	28	13	5	4				15	5
JCVIGM_433684_20110307	pgi	glucose-6-phosphate isomerase			2	8	4	5	7				1		6			3	
JCVIGM_036452_20110307	pgiA	glucose-6-phosphate isomerase A		2	1	1		1	2		1	2	1	2					
JCVIGM_001673_20110307	uxaC	glucuronate isomerase							1		1								
JCVIGM_001890_20110307	uxaC	glucuronate isomerase		1				2											
JCVIGM_105608_20110307	uxaC	glucuronate isomerase		1			1				1							2	
JCVIGM_028337_20110307		glutamate dehydrogenase					1						1	1					1
JCVIGM_079729_20110307	GDH	glutamate dehydrogenase (fragment)																	
JCVIGM_026222_20110307		glutamate dehydrogenase (NADP(+))		2	1		1	2	1			2						1	1
JCVIGM_051813_20110307		glutamate dehydrogenase, nADP-specific		1	3		1		1		1	2	2		1	1			1
JCVIGM_081547_20110307		glutamate dehydrogenase, NADP-specific		19	7	6	7	15	9	13	11	16	7	7		5	3	7	7
JCVIGM_083070_20110307		glutamate dehydrogenase, NADP-specific		2	7	1	1	1					2						
JCVIGM_112581_20110307		glutamate dehydrogenase, NADP-specific		1	4														
JCVIGM_235230_20110307		glutamate dehydrogenase, NADP-specific		5	4	3	5	9	9	5	6	5	6	3	2	2	2	3	2
JCVIGM_395419_20110307		glutamate dehydrogenase, NADP-specific		2			2	1	1	2	1	1		1					
JCVIGM_000822_20110307	gdhB	glutamate dehydrogenase, NAD-specific		3	4	4	3	3	1	1	2	1	1	2	1			1	3
JCVIGM_031962_20110307	gdhB	glutamate dehydrogenase, NAD-specific		1	1														
JCVIGM_033111_20110307	gdhB	glutamate dehydrogenase, NAD-specific		1		1					1								
JCVIGM_044177_20110307	gdhB	glutamate dehydrogenase, NAD-specific											1		1				
JCVIGM_072261_20110307	gdh	glutamate dehydrogenase, NAD-specific		2	2	1	1	2		1	1	1	1					2	
JCVIGM_124713_20110307	gdhB	glutamate dehydrogenase, NAD-specific		2	2		2				1				2			1	
JCVIGM_030438_20110307		glutamate dehydrogenase/leucine dehydrogenase		3	3	5	4	7	7	2	3	3	5	3	4	1		3	1
JCVIGM_067262_20110307		glutamate dehydrogenase/leucine dehydrogenase		2					1		1	1		1		1		1	1
JCVIGM_071296_20110307		glutamate dehydrogenase/leucine dehydrogenase		2	2		1	2			2	2	2					2	
JCVIGM_130579_20110307		glutamate dehydrogenase/leucine dehydrogenase		1	6	1	1	1	1	3	2	3	3	2		1		1	
JCVIGM_141521_20110307		glutamate dehydrogenase/leucine dehydrogenase		10	6	3	6	10	7	8	8	9	7	3	1	4	3	2	4
JCVIGM_226798_20110307		glutamate dehydrogenase/leucine dehydrogenase		5	3	2	2	9	6	6	5	3	4	4		1	1	5	1
JCVIGM_167424_20110307	gltA	glutamate synthase (NADPH), homotetrameric			1		1	1	1				2						

JCVIGM_009248_20110307	gap	glyceraldehyde 3-phosphate dehydrogenase, C-terminal domain		5		2	2	1	2	1	3	4		1		1		3	2
JCVIGM_027319_20110307		glyceraldehyde 3-phosphate dehydrogenase, C-terminal domain		5	1	2	1	5	3	2	4	3	2	1		1	1	2	
JCVIGM_075124_20110307		glyceraldehyde 3-phosphate dehydrogenase, C-terminal domain		2	2	3	1	1	1	2	3	4		2	2	5		2	5
JCVIGM_079320_20110307		glyceraldehyde 3-phosphate dehydrogenase, C-terminal domain						1		4	1	2				1			
JCVIGM_083011_20110307		glyceraldehyde 3-phosphate dehydrogenase, C-terminal domain												4	3	4	4	6	
JCVIGM_088090_20110307		glyceraldehyde 3-phosphate dehydrogenase, C-terminal domain		1		1	2			1	1	2	1	1	3	2	1	1	2
JCVIGM_141419_20110307		glyceraldehyde 3-phosphate dehydrogenase, C-terminal domain		3		1	3			1	2	7		4	2	2		4	5
JCVIGM_159787_20110307		glyceraldehyde 3-phosphate dehydrogenase, C-terminal domain		2			1	1			2	1				1			
JCVIGM_169660_20110307		glyceraldehyde 3-phosphate dehydrogenase, C-terminal domain		2			1	1			2	2						1	1
JCVIGM_170407_20110307		glyceraldehyde 3-phosphate dehydrogenase, C-terminal domain		4	1	1	5	2	3	3	4	7	1	3	3	2	2	4	4
JCVIGM_189194_20110307		glyceraldehyde 3-phosphate dehydrogenase, C-terminal domain		4	3	2	2	3	3	4	6	3	2	1		1	1		
JCVIGM_224384_20110307		glyceraldehyde 3-phosphate dehydrogenase, C-terminal domain		2	1	2	2	2	5		3	1	1			1		1	
JCVIGM_268659_20110307		glyceraldehyde 3-phosphate dehydrogenase, C-terminal domain		3	3		1	2	4		6						1	2	
JCVIGM_313872_20110307		glyceraldehyde 3-phosphate dehydrogenase, C-terminal domain		1								1							1
JCVIGM_319196_20110307		glyceraldehyde 3-phosphate dehydrogenase, C-terminal domain								1		1	2						
JCVIGM_360253_20110307		glyceraldehyde 3-phosphate dehydrogenase, C-terminal domain												2	1	2	1	3	
JCVIGM_100377_20110307	gap	glyceraldehyde-3-phosphate dehydrogenase, type I		12	16	6	9	12	18	10	13	8	15	6		3	3	3	3
JCVIGM_294702_20110307	pduC	glycerol dehydratase large subunit		3	10	9	5	10	10		2	2	2						
JCVIGM_338330_20110307		glycerol dehydrogenase and related enzymes		2	2	6	2	2	2	2	1	2		1					
JCVIGM_213371_20110307	glyA	glycine hydroxymethyltransferase																	1
JCVIGM_015371_20110307		histidine kinase			1			1					1						
JCVIGM_369000_20110307		hydrogenase, Fe-only										1							
JCVIGM_048518_20110307		hydrolyase, tartrate beta subunit/fumarate domain protein, Fe-S type		1		1	3				1								
JCVIGM_000577_20110307		hypothetical protein					1				2								2
JCVIGM_001656_20110307		hypothetical protein		3	2		1	2	3	1	1	2	3	1		1	1		
JCVIGM_004031_20110307		hypothetical protein			1		1		1		1	2	1	1				2	1
JCVIGM_006083_20110307		hypothetical protein					1	2	1										
JCVIGM_006988_20110307		hypothetical protein			2				2										
JCVIGM_007410_20110307		hypothetical protein		1			1	1	1	1	2	2							
JCVIGM_009250_20110307		hypothetical protein		3								1							

JCVIGM_010628_20110307		hypothetical protein		2		1	1				1	2				1		1	2
JCVIGM_011271_20110307		hypothetical protein		2		1		4			2		2					2	
JCVIGM_011336_20110307		hypothetical protein		1															
JCVIGM_012967_20110307		hypothetical protein		1		1		1	1			2	2	1		1		1	
JCVIGM_014024_20110307		hypothetical protein		1	1			1	1		1	1				1		2	
JCVIGM_018751_20110307		hypothetical protein		1						2	1			1				2	
JCVIGM_020128_20110307		hypothetical protein		1	1		1						3						1
JCVIGM_021326_20110307		hypothetical protein		20	19	6	13	12	20	7	7	8	15	7	2	7	9	1	1
JCVIGM_021364_20110307		hypothetical protein		1			1												
JCVIGM_021629_20110307		hypothetical protein			1		1		1		2	1							
JCVIGM_022560_20110307		hypothetical protein		1	2			1	1	1	2	1							
JCVIGM_022561_20110307		hypothetical protein		1							1								
JCVIGM_025687_20110307		hypothetical protein																	
JCVIGM_025940_20110307		hypothetical protein					1												
JCVIGM_026248_20110307		hypothetical protein		2			1	1	1	1	1	2						1	1
JCVIGM_026306_20110307		hypothetical protein		23	16	12	17	23	24	14	32	21	20	11	6	8	6	13	7
JCVIGM_029504_20110307		hypothetical protein		1	1			1										1	
JCVIGM_029868_20110307		hypothetical protein		2	2				1	2	2	2	2	1		1		1	1
JCVIGM_030581_20110307		hypothetical protein		3	2	1	3	4	4	2	4	6	3					1	
JCVIGM_031963_20110307		hypothetical protein				1													
JCVIGM_036375_20110307		hypothetical protein		5		1	6	2	3	1	4	2						2	
JCVIGM_036678_20110307		hypothetical protein					1	1	1	2	1								
JCVIGM_037187_20110307		hypothetical protein		13	7	3	8	18	15	8	16	11	8	4	2	4	3	9	5
JCVIGM_039163_20110307		hypothetical protein		26	16	7	35	40	63	99	25	31	6	6	2	6	4	16	7
JCVIGM_039195_20110307		hypothetical protein		18	22	6	14	41	18	56	37	78	62	15	12	7	2	29	12
JCVIGM_042328_20110307		hypothetical protein		3		2	2	1		1	1	2							1
JCVIGM_043843_20110307		hypothetical protein		2															1
JCVIGM_044734_20110307		hypothetical protein																	
JCVIGM_045412_20110307		hypothetical protein																	
JCVIGM_047061_20110307		hypothetical protein		1			2												

JCVIGM_048990_20110307		hypothetical protein		1	4			3	3	1	2	2	3						
JCVIGM_052263_20110307		hypothetical protein		1				1											
JCVIGM_054391_20110307		hypothetical protein		1															
JCVIGM_055922_20110307		hypothetical protein		8	6	13	8	3	5		1	3	12	1				3	
JCVIGM_056525_20110307		hypothetical protein			3							1	1						
JCVIGM_057825_20110307		hypothetical protein		2	2	1	2	2	4	1	3	2		1	1	1		1	1
JCVIGM_058381_20110307		hypothetical protein										1							1
JCVIGM_060468_20110307		hypothetical protein		3	1		3	3	1										
JCVIGM_060720_20110307		hypothetical protein		2															
JCVIGM_061313_20110307		hypothetical protein		3	5					2	2	2						2	
JCVIGM_061338_20110307		hypothetical protein		1	2	1	2	3	3	2	6	2	3	1	1	2		3	1
JCVIGM_063878_20110307		hypothetical protein		1			1												
JCVIGM_065213_20110307		hypothetical protein				1			1										
JCVIGM_068011_20110307		hypothetical protein		3		2	4	1			3	1				1		1	
JCVIGM_068020_20110307		hypothetical protein																	
JCVIGM_070048_20110307		hypothetical protein		2															
JCVIGM_070908_20110307		hypothetical protein						1	1		2		2						
JCVIGM_071303_20110307		hypothetical protein		1									1						4
JCVIGM_071361_20110307		hypothetical protein			2			1					2						
JCVIGM_071586_20110307		hypothetical protein		7	1	2	12	1		1	1	1	1	1	8	2	1	4	2
JCVIGM_071718_20110307		hypothetical protein			1	1	1												
JCVIGM_072139_20110307		hypothetical protein		2		1	1	1		1	2	1		1				2	
JCVIGM_072463_20110307		hypothetical protein		1		1	3		1									1	
JCVIGM_073483_20110307		hypothetical protein				1	1	2											
JCVIGM_074339_20110307		hypothetical protein		6	4		1	7	4	3	11	6	3					2	
JCVIGM_074623_20110307		hypothetical protein		1	1														
JCVIGM_075200_20110307		hypothetical protein		10	1	1				3	1								
JCVIGM_077653_20110307		hypothetical protein		1			1		1		1							1	
JCVIGM_081664_20110307		hypothetical protein		1				1	1									1	

JCVIGM_083533_20110307		hypothetical protein		4	1		2	3	4	4	4	4	5	4	1	2	1	3	1
JCVIGM_084534_20110307		hypothetical protein		1	1		2	1	1		2	1	2					2	1
JCVIGM_084535_20110307		hypothetical protein			2	1	2	1	2	1	2	1		2				2	
JCVIGM_088087_20110307		hypothetical protein		1	1								3						1
JCVIGM_088594_20110307		hypothetical protein		4	3	2	3	3	8	2	3	9	6	2				3	
JCVIGM_090261_20110307		hypothetical protein		1	2	1	1	2	2	1	4	1	4	2	1	3	1		2
JCVIGM_090262_20110307		hypothetical protein		2		3	3	4	5	3	3	4		1	1	2		3	
JCVIGM_090263_20110307		hypothetical protein		1	2	1			1				2						
JCVIGM_090646_20110307		hypothetical protein		3															
JCVIGM_091802_20110307		hypothetical protein		1	4	1	3	4	5		1							2	
JCVIGM_091850_20110307		hypothetical protein			3								6	1					
JCVIGM_092014_20110307		hypothetical protein		4	2	2	14	14	14	1	9	2						2	
JCVIGM_092247_20110307		hypothetical protein						1								1			
JCVIGM_092708_20110307		hypothetical protein		9		3	27	10	5	1	7	1						2	
JCVIGM_095744_20110307		hypothetical protein		5															
JCVIGM_097721_20110307		hypothetical protein					1								1				1
JCVIGM_098373_20110307		hypothetical protein		3		2	2			2	3	4					1	3	2
JCVIGM_101299_20110307		hypothetical protein				1	1	1	1			1							
JCVIGM_101300_20110307	etfB	hypothetical protein		6	1	2		4	7	7	1	2					1	1	
JCVIGM_102281_20110307		hypothetical protein		17	13	2	6	7	3	1	7	1	10	1				16	2
JCVIGM_102458_20110307		hypothetical protein		4															
JCVIGM_102522_20110307		hypothetical protein		1		1	1	2	2	2	3	2	1	1		1			
JCVIGM_102523_20110307		hypothetical protein		3		1	2	2	3	4	2	4		2					
JCVIGM_103433_20110307		hypothetical protein		4	2	2	2	3	2	1	2	4	2	5				1	
JCVIGM_104408_20110307		hypothetical protein				1		1	1										
JCVIGM_107850_20110307		hypothetical protein			2														
JCVIGM_110415_20110307		hypothetical protein		1					2		2					1			
JCVIGM_112258_20110307		hypothetical protein		3		1	2	1	1	1	1	2					1	1	4
JCVIGM_113282_20110307		hypothetical protein			3			1				1	5						
JCVIGM_115169_20110307		hypothetical protein		4		1	9	1			6	2							
JCVIGM_115489_20110307		hypothetical protein		2															

JCVIGM_116061_20110307		hypothetical protein		1	1														
JCVIGM_116471_20110307		hypothetical protein		1	1														
JCVIGM_118383_20110307		hypothetical protein		6	1	1	1		2										
JCVIGM_121048_20110307		hypothetical protein																	
JCVIGM_121384_20110307		hypothetical protein		3															
JCVIGM_121883_20110307		hypothetical protein		8	7	4	5	6	6	6	12	9	7	4	3	3	2	6	2
JCVIGM_122516_20110307		hypothetical protein																	
JCVIGM_125587_20110307		hypothetical protein						1				1							
JCVIGM_126576_20110307		hypothetical protein					2		1		1								
JCVIGM_128170_20110307		hypothetical protein		3	4		1			4	3	4	5	3				2	
JCVIGM_128306_20110307		hypothetical protein		26	1														
JCVIGM_129017_20110307		hypothetical protein		6			1				2								
JCVIGM_129660_20110307		hypothetical protein						2											
JCVIGM_130216_20110307		hypothetical protein																	
JCVIGM_132903_20110307		hypothetical protein		22	15	16	4	31	38	23	23	110	3	3	2	3	1	28	1
JCVIGM_133803_20110307		hypothetical protein			1														
JCVIGM_134883_20110307		hypothetical protein		2	1	1			2	1	1	1	1						
JCVIGM_135963_20110307		hypothetical protein																	
JCVIGM_141001_20110307		hypothetical protein						1	1									1	
JCVIGM_141853_20110307		hypothetical protein		3		4	3	5	6		4	2	3		1				
JCVIGM_142860_20110307		hypothetical protein																	
JCVIGM_144970_20110307		hypothetical protein						1											
JCVIGM_147926_20110307		hypothetical protein		5	2	3	5	9	1	3	8	2	11	2				2	1
JCVIGM_148595_20110307		hypothetical protein		1	3	1	1	1	2				2					1	
JCVIGM_148602_20110307		hypothetical protein		1				1											
JCVIGM_148969_20110307		hypothetical protein		7			4	13	1	25	28	26		5				8	
JCVIGM_150315_20110307		hypothetical protein									2								
JCVIGM_150334_20110307		hypothetical protein		1				1	1									1	
JCVIGM_150335_20110307		hypothetical protein		4	2	2	5	7	4	2	4	5	4	2	2			2	
JCVIGM_153727_20110307		hypothetical protein		3	3	2	2	4	3	1	3	4	3	1	1	2		1	1
JCVIGM_155277_20110307		hypothetical protein		1			2	3	3	1		2							
JCVIGM_156043_20110307		hypothetical protein		3	1		1	2	2	2	2	2	2	1		1		1	
JCVIGM_160972_20110307		hypothetical protein		2	7		1		1				1						
JCVIGM_161538_20110307		hypothetical protein						2	1	1	1								

JCVIGM_161966_20110307		hypothetical protein		2		2	2	3			1		2	1					
JCVIGM_163002_20110307		hypothetical protein		2	3	1	3	2	4		2		3					4	
JCVIGM_166279_20110307		hypothetical protein						2		1	1			2					
JCVIGM_169083_20110307		hypothetical protein																	
JCVIGM_170674_20110307		hypothetical protein		8	2	1	2	2	3	12	3	4	1	3		2	2	2	
JCVIGM_173056_20110307		hypothetical protein		8	3		1	3	1		1								
JCVIGM_173583_20110307		hypothetical protein			3			1					1						
JCVIGM_175085_20110307		hypothetical protein		5	3			4	2	5	12	7	5	2		1	2	3	2
JCVIGM_175220_20110307		hypothetical protein		2		1	1	1	5	1	1								
JCVIGM_176221_20110307		hypothetical protein		1			1		1	1	1	1						1	
JCVIGM_181018_20110307		hypothetical protein		3	1	2		3	3		3	1	5		1	1	1	1	1
JCVIGM_182077_20110307		hypothetical protein		3	2	1	3	2	2	2	2	3	1						
JCVIGM_185211_20110307		hypothetical protein																	
JCVIGM_185931_20110307		hypothetical protein									1							1	
JCVIGM_187544_20110307		hypothetical protein											2						
JCVIGM_189376_20110307		hypothetical protein																	
JCVIGM_189604_20110307		hypothetical protein			1					1			1						
JCVIGM_190292_20110307		hypothetical protein									1	1	1						
JCVIGM_190977_20110307		hypothetical protein									1	1	1						
JCVIGM_191291_20110307		hypothetical protein			1					1	1	2							
JCVIGM_191293_20110307		hypothetical protein		2	4	2	5	2	4	8	10	7						1	
JCVIGM_191827_20110307		hypothetical protein										1							
JCVIGM_191888_20110307		hypothetical protein					1	1			4	2	1					5	
JCVIGM_192065_20110307		hypothetical protein						1	1	1	1						1		
JCVIGM_194742_20110307		hypothetical protein					1		2	3	5	1							
JCVIGM_194897_20110307		hypothetical protein		2	2		1	4	3	2	3	2	2					2	
JCVIGM_195386_20110307		hypothetical protein							1	1		1				1			
JCVIGM_196029_20110307		hypothetical protein		2		1		1	1	1	2	1			1			1	1
JCVIGM_202561_20110307		hypothetical protein		3			2	2	1	2	4	3	1	1				3	
JCVIGM_203237_20110307		hypothetical protein		1			1	1			1	1							
JCVIGM_204096_20110307		hypothetical protein					1												
JCVIGM_205608_20110307		hypothetical protein		1		1	4												
JCVIGM_207243_20110307		hypothetical protein						1	2										

JCVIGM_209517_20110307		hypothetical protein		4	4		3	4	4	3	3	4	2	1		2		5	2
JCVIGM_210696_20110307		hypothetical protein											4						
JCVIGM_210946_20110307		hypothetical protein		3		2	2	1	4	2	2	1							
JCVIGM_211394_20110307		hypothetical protein		1	3	1	2	2	2	1	2	2	1					3	1
JCVIGM_212706_20110307		hypothetical protein		7	4	1	1	10	5	1	9	2	5					3	
JCVIGM_213389_20110307		hypothetical protein		2	2	2	3	3	2	3	3	5	3	3		1		2	
JCVIGM_219434_20110307		hypothetical protein														1			
JCVIGM_219781_20110307		hypothetical protein					2		1	1	1	1	1		1				
JCVIGM_222747_20110307		hypothetical protein			2							1							
JCVIGM_229339_20110307		hypothetical protein																	
JCVIGM_234241_20110307		hypothetical protein		2	1	2	2	4	3		3	3	3	2				1	
JCVIGM_237601_20110307		hypothetical protein			1									6	10			1	1
JCVIGM_239052_20110307		hypothetical protein									1				1				
JCVIGM_245141_20110307		hypothetical protein		5	15	2	7	6	6	3	8	8	11	8		3	2	7	3
JCVIGM_246343_20110307		hypothetical protein							1		1								
JCVIGM_246344_20110307		hypothetical protein		1				1											
JCVIGM_247614_20110307		hypothetical protein					1				1						1		2
JCVIGM_249782_20110307		hypothetical protein													3		2		
JCVIGM_251147_20110307		hypothetical protein		1			1												
JCVIGM_251362_20110307		hypothetical protein		9	8	5	9	10	11	7	10	15	10	9	4	4	4	7	6
JCVIGM_251712_20110307		hypothetical protein		3			1	1	1	3	5	3				1	1		2
JCVIGM_256398_20110307		hypothetical protein		2															
JCVIGM_259370_20110307		hypothetical protein											4						
JCVIGM_262707_20110307		hypothetical protein			1							1							
JCVIGM_265819_20110307	etfB	hypothetical protein		5	4	2	1		1		3		1						
JCVIGM_268646_20110307		hypothetical protein		2		1	4	2	2									1	
JCVIGM_274621_20110307		hypothetical protein																	1
JCVIGM_277806_20110307		hypothetical protein		2	1	1		1	2	2	2	2		1					
JCVIGM_281111_20110307		hypothetical protein		2	2			1	1	3	2	2	1	1				2	
JCVIGM_281956_20110307		hypothetical protein		2	1	3		2	4		2			2	2			2	
JCVIGM_282080_20110307		hypothetical protein		1	1		1				2								

JCVIGM_283350_20110307		hypothetical protein		1	1														
JCVIGM_286196_20110307		hypothetical protein		1	2	1	1	2			2	2	3	2		1	2	3	2
JCVIGM_286605_20110307		hypothetical protein		6	7	2	5	6	6	2	7	3	8	3	3	2	1	6	4
JCVIGM_288269_20110307		hypothetical protein		7	8	1	4	5	6	1	7	2	3	1				3	
JCVIGM_288716_20110307		hypothetical protein																	
JCVIGM_291424_20110307		hypothetical protein					1	1	1		1		1						
JCVIGM_295105_20110307		hypothetical protein			2					1		2	2	1				1	
JCVIGM_295523_20110307		hypothetical protein		3	3	6	8	11	13		3		18					1	
JCVIGM_296216_20110307		hypothetical protein		15	8	5	13	17	18	10	26	12	15	8				19	9
JCVIGM_298432_20110307		hypothetical protein		1						1	1	1	1						
JCVIGM_298496_20110307		hypothetical protein		2	1	1	2	2	1		2	1	3					1	
JCVIGM_301982_20110307		hypothetical protein		2	1	1			3										
JCVIGM_302456_20110307		hypothetical protein		3			3	3	1		2	2	1					2	
JCVIGM_303429_20110307		hypothetical protein						1					1						
JCVIGM_303566_20110307		hypothetical protein																	
JCVIGM_303609_20110307		hypothetical protein					1	1	1	1	2	1						1	
JCVIGM_303798_20110307		hypothetical protein		2	2	1	2	1	2	3	3	3	2	1				1	
JCVIGM_306883_20110307		hypothetical protein		1	1														1
JCVIGM_306884_20110307		hypothetical protein			1			1				2							1
JCVIGM_307057_20110307		hypothetical protein				1	1	3	2	1	3			1				1	
JCVIGM_307436_20110307		hypothetical protein		1	4					1	1	3	6	1				2	
JCVIGM_307765_20110307		hypothetical protein				1	1	1	1	1	1								
JCVIGM_309808_20110307		hypothetical protein		2	2	2	2	2	2	2	2	2	2	2				1	1
JCVIGM_310529_20110307		hypothetical protein			4	6													
JCVIGM_313684_20110307		hypothetical protein						4	5										
JCVIGM_314099_20110307		hypothetical protein										1							
JCVIGM_314549_20110307		hypothetical protein		4	3	1	2	3	3	3	3	1	1					4	1
JCVIGM_319934_20110307		hypothetical protein		6	6	12	16	11	19		5		8					8	
JCVIGM_322352_20110307		hypothetical protein			2														

JCVIGM_323810_20110307		hypothetical protein											1	1					
JCVIGM_327403_20110307		hypothetical protein						1			1								
JCVIGM_328614_20110307		hypothetical protein		1	1														
JCVIGM_332056_20110307		hypothetical protein			2														
JCVIGM_332688_20110307		hypothetical protein		3	4	2	1	6	4		2	2	8	1		1	1	1	
JCVIGM_333745_20110307		hypothetical protein		3	2	1		1		1	3	2	5	1			1	1	
JCVIGM_336004_20110307		hypothetical protein				2		1	1									1	
JCVIGM_336057_20110307		hypothetical protein												1					
JCVIGM_340014_20110307		hypothetical protein																	
JCVIGM_340609_20110307		hypothetical protein		8	15	1	7	5	7	5	8	11	15	3		3	1	4	4
JCVIGM_341200_20110307		hypothetical protein		1		1	3	3	5		3	1	3		2			1	
JCVIGM_344661_20110307		hypothetical protein							1		5	1							
JCVIGM_345968_20110307		hypothetical protein					2	1			4								
JCVIGM_348560_20110307		hypothetical protein																	
JCVIGM_351456_20110307		hypothetical protein			3			1				1	2						
JCVIGM_359900_20110307		hypothetical protein						1				1							
JCVIGM_362686_20110307		hypothetical protein		3	2	1		1	2	3	2	3	1	2					
JCVIGM_364085_20110307		hypothetical protein																	
JCVIGM_365092_20110307		hypothetical protein			2								1						
JCVIGM_367321_20110307		hypothetical protein														1	1		
JCVIGM_367720_20110307		hypothetical protein		3		1	2	2	2	2	4	3						2	
JCVIGM_368650_20110307		hypothetical protein		3	2														
JCVIGM_369484_20110307		hypothetical protein		2		1	1	1	2	1	3	2							
JCVIGM_371571_20110307		hypothetical protein									1							2	
JCVIGM_372380_20110307		hypothetical protein					2	1	1		2		1						
JCVIGM_373222_20110307		hypothetical protein		1			1	2			3	1							
JCVIGM_373315_20110307		hypothetical protein																	
JCVIGM_375019_20110307		hypothetical protein					1		3									1	
JCVIGM_379110_20110307		hypothetical protein		1	1		1	1			2	1	1					1	

JCVIGM_380771_20110307		hypothetical protein					1	1										1	1
JCVIGM_387933_20110307		hypothetical protein																1	4
JCVIGM_390199_20110307		hypothetical protein						1	1										
JCVIGM_390242_20110307		hypothetical protein		1	1		3												
JCVIGM_390806_20110307		hypothetical protein		1		2	3	3	3	1	3	2						2	2
JCVIGM_391550_20110307		hypothetical protein			1								1						
JCVIGM_393860_20110307		hypothetical protein			1														
JCVIGM_398055_20110307		hypothetical protein			1								1						
JCVIGM_398168_20110307		hypothetical protein									1	5							
JCVIGM_399462_20110307		hypothetical protein																	
JCVIGM_400027_20110307		hypothetical protein		4	3	3	3	10	7	4	5	3	5					2	
JCVIGM_400278_20110307		hypothetical protein						1										2	
JCVIGM_400950_20110307		hypothetical protein					2												
JCVIGM_405787_20110307		hypothetical protein											1						
JCVIGM_410986_20110307		hypothetical protein																	
JCVIGM_414552_20110307		hypothetical protein		3	3	5	3	3	2		4	4	9	2				4	
JCVIGM_418955_20110307		hypothetical protein																	
JCVIGM_420396_20110307		hypothetical protein						1	1										
JCVIGM_420773_20110307		hypothetical protein		1			1		1								1		
JCVIGM_424310_20110307		hypothetical protein		2	1	2	1	1	1	3	4	2	3	2		1		1	
JCVIGM_434782_20110307		hypothetical protein						1	1										
JCVIGM_441424_20110307		hypothetical protein													1				
JCVIGM_444308_20110307		hypothetical protein		1								1							
JCVIGM_444309_20110307		hypothetical protein		1	1			1	1				2						
JCVIGM_055829_20110307		hypothetical protein BACCOPRO_02435							1										
JCVIGM_158091_20110307		hypothetical protein BACCOPRO_03643		2	2	2	2	2	2	2	3	2	2		2			1	2
JCVIGM_068437_20110307		hypothetical protein CLOST_1033																	
JCVIGM_442772_20110307		hypothetical protein ELI_0537		1	2													1	
JCVIGM_010811_20110307		inosine guanosine and xanthosine phosphorylase family		2		2	1	1	1	1	1	1	1		1			2	3

JCVIGM_403964_20110307		inosine guanosine and xanthosine phosphorylase family protein		1	1	1	1				3	3	1	2					5
JCVIGM_054853_20110307	guaB	inosine-5'-monophosphate dehydrogenase					1				1		1						
JCVIGM_092237_20110307		inosine-5'-monophosphate dehydrogenase		7															
JCVIGM_126259_20110307	guaB	inosine-5'-monophosphate dehydrogenase		2			1				1	1	2						1
JCVIGM_196447_20110307		inosine-5'-monophosphate dehydrogenase		1		1	1	3	3				2						
JCVIGM_255764_20110307		inosine-5'-monophosphate dehydrogenase		1	1	2	1	5	4										
JCVIGM_272653_20110307		inosine-5'-monophosphate dehydrogenase		1	1	1	1	2	1		2	1	2					1	
JCVIGM_328476_20110307		inosine-5'-monophosphate dehydrogenase			1					1	1	1		1				1	1
JCVIGM_357305_20110307	iolG	inositol 2-dehydrogenase				1		1	1									1	
JCVIGM_069994_20110307		inositol-3-phosphate synthase		1		1	2				2								
JCVIGM_091917_20110307		inositol-3-phosphate synthase				1	1												
JCVIGM_184954_20110307		integral membrane sensor signal transduction histidine kinase													2	2	2	1	
JCVIGM_065899_20110307		internalin-related protein		26	8	15	24	12	11	7	15	15	6		24			5	10
JCVIGM_120727_20110307	ilvC	ketol-acid reductoisomerase		6	2	2	1	4	2	8		13		1				2	
JCVIGM_156339_20110307	ilvC	ketol-acid reductoisomerase		9	17	7	11	12	11	6	16	9	17	5		1	1	8	5
JCVIGM_185462_20110307	ilvC	ketol-acid reductoisomerase		1	2	3													
JCVIGM_251867_20110307	ilvC	ketol-acid reductoisomerase						2					1				1		1
JCVIGM_287230_20110307	ilvC	ketol-acid reductoisomerase		2	2			2	1		1								
JCVIGM_322248_20110307	ilvC	ketol-acid reductoisomerase		2			1	2			1			1					
JCVIGM_062252_20110307		ketose-bisphosphate aldolase, class II					1		1		1								
JCVIGM_128542_20110307		ketose-bisphosphate aldolase, class II		3					1										
JCVIGM_260030_20110307		KHG/KDPG family aldolase/carbohydrate kinase, pfkB family					1		1										
JCVIGM_418431_20110307		kinase, pfkB family		3	1		1	2	2		9	2	4					7	1
JCVIGM_025581_20110307	araA	L-arabinose isomerase		1	2	1	1	2	2	3	2	3	1	1		1			
JCVIGM_046489_20110307	araA	L-arabinose isomerase		1	1			1			1	1						2	
JCVIGM_060257_20110307	araA	L-arabinose isomerase		2	2	1	1	4	3	28	1	1							
JCVIGM_073944_20110307	araA	L-arabinose isomerase		4	3	4	8	9	11	3	5	3	4	4		3	1	4	2
JCVIGM_074178_20110307	araA	L-arabinose isomerase																	
JCVIGM_086747_20110307	araA	L-arabinose isomerase		4	4	5	11	7	15	1	12	6	5	1					

JCVIGM_088705_20110307	araA	L-arabinose isomerase		2							1								
JCVIGM_168776_20110307	araA	L-arabinose isomerase				1	2	2	1		1		1						
JCVIGM_206950_20110307	araA1	L-arabinose isomerase 1		1			2	1	1				1					1	
JCVIGM_102423_20110307		L-asparaginase		1		2	2	1			1	1							
JCVIGM_051778_20110307		L-asparaginase, type II									2								
JCVIGM_177219_20110307		L-fucose isomerase and related proteins		2					2										
JCVIGM_022476_20110307	fucI	L-fucose isomerase, C-terminal domain			3		1	2	2		1	1	5						
JCVIGM_059070_20110307		L-fucose isomerase, C-terminal domain		6		3		1	2		2	1							
JCVIGM_066045_20110307		L-fucose isomerase, C-terminal domain		2	2	2	2	3	3	1	2	2	2	1			2		1
JCVIGM_101032_20110307		L-fucose isomerase, C-terminal domain		5	3	3	2	3	3	1	3	5	2	2	2		1		2
JCVIGM_106686_20110307		L-fucose isomerase, C-terminal domain		1	4	1	3	2	2		3	2	8	1	1			2	
JCVIGM_124735_20110307		L-fucose isomerase, C-terminal domain		1	2		1												
JCVIGM_187352_20110307		L-fucose isomerase, C-terminal domain		2	2	1	2	1	2	2	3	2	3	2	1		1	2	1
JCVIGM_278994_20110307		L-fucose isomerase, C-terminal domain								1	1	1							1
JCVIGM_325641_20110307		L-fucose isomerase-like protein			1	1	2	3	3				1						
JCVIGM_171365_20110307		lipoprotein														2			
JCVIGM_083497_20110307	ldh	L-lactate dehydrogenase		1	1	1	1	1	1	2	1	2	1	2		1			
JCVIGM_159477_20110307	dapL	LL-diaminopimelate aminotransferase			1				1				1						
JCVIGM_130368_20110307		L-ribulose-5-phosphate 4-epimerase		1	1			2	1		2	1						2	
JCVIGM_060255_20110307	araD	L-ribulose-5-phosphate 4-epimerase araD		2	2		1	2	2	1	2	1		1		1		2	1
JCVIGM_160693_20110307	araD	L-ribulose-5-phosphate 4-epimerase araD			1		1	2	1										
JCVIGM_295794_20110307	araD	L-ribulose-5-phosphate 4-epimerase araD		1			1	1	2		1								
JCVIGM_000243_20110307	lysS	lysine--tRNA ligase		1			1												
JCVIGM_161438_20110307		M18 family aminopeptidase			1					1	1	1	1						
JCVIGM_203664_20110307		M18 family aminopeptidase		2															
JCVIGM_229762_20110307		major outer membrane lipoprotein 1												5	27	6	7	12	
JCVIGM_006253_20110307	mdh	malate dehydrogenase		2			1										1		2
JCVIGM_012327_20110307	mdh	malate dehydrogenase		3		1	2	1	2	3	2	3	2	1	1	1	1	1	2
JCVIGM_017662_20110307		malate dehydrogenase		3		2	3			2	2	3			2	1		1	2
JCVIGM_026640_20110307	mdh	malate dehydrogenase																	

JCVIGM_028360_20110307	mdh	malate dehydrogenase		4		4	6	2	3	1	6	3	3	2	3	1	2	5	4
JCVIGM_127124_20110307	mdh	malate dehydrogenase		3	2	1	2		1	6	2	5	1	3		6	3	3	4
JCVIGM_229225_20110307	mdh	malate dehydrogenase													1				
JCVIGM_079110_20110307		malate dehydrogenase (oxaloacetate-decarboxylating)		1		1	1	1	1										
JCVIGM_020629_20110307	mdh	malate dehydrogenase, NAD-dependent		2	1	1	2	2	1		2	2	1		3			1	2
JCVIGM_007627_20110307		maltose-binding protein					1					1							
JCVIGM_201419_20110307		maltose-binding protein		3	2			2	2	4	5	3	1	1		1		5	2
JCVIGM_173530_20110307		membrane protein, bmp family		2															
JCVIGM_186148_20110307		membrane protein, bmp family		5															
JCVIGM_052677_20110307		metallo-beta-lactamase superfamily								2		2							
JCVIGM_038699_20110307	metK	methionine adenosyltransferase				1		1											
JCVIGM_046971_20110307	metK	methionine adenosyltransferase																	
JCVIGM_076017_20110307	metK	methionine adenosyltransferase						1	1										
JCVIGM_211427_20110307		methylmalonate-semialdehyde dehydrogenase (acylating)																	
JCVIGM_014933_20110307	mmdA	methylmalonyl-coA decarboxylase		1	2		1						1						
JCVIGM_034121_20110307		methylmalonyl-coA mutase large subunit										1							1
JCVIGM_255025_20110307		methylmalonyl-coA mutase large subunit		2															
JCVIGM_082989_20110307		methylmalonyl-coA mutase N-terminal domain					1				1								
JCVIGM_069236_20110307	mutA	methylmalonyl-coA mutase, small subunit		1			1											1	
JCVIGM_033013_20110307		microcompartments protein		4	4	1	4	2	1	1	7	3	6	2		2	1	3	
JCVIGM_083041_20110307		microcompartments protein		2		1	2	2	1	1	3	3	2	1				3	
JCVIGM_188817_20110307		monosaccharide ABC transporter substrate-binding protein, CUT2 family					1	1	1	1	4	2							
JCVIGM_039831_20110307		monosaccharide ABC transporter substrate-binding protein, CUT2 family		1				1						1				1	
JCVIGM_192144_20110307		monosaccharide ABC transporter substrate-binding protein, CUT2 family			1	1	2	2										1	
JCVIGM_070547_20110307	purE	N5-carboxyaminoimidazole ribonucleotide mutase																	
JCVIGM_060713_20110307		N-acetylneuraminate lyase		6	2			1	1		1		1						
JCVIGM_233593_20110307	nuoI	NADH-quinone oxidoreductase subunit I			1								1		1				
JCVIGM_057221_20110307		NADP-dependent malic enzyme		4															
JCVIGM_231459_20110307		NADPH-dependent butanol dehydrogenase				1													
JCVIGM_121448_20110307		nifU-related domain containing protein		5	1	1	2	4	4	3	3	3	2	2		1	1	2	1

JCVIGM_028245_20110307		nitrate/sulfonate/bicarbonate ABC transporter, periplasmic nitrate/sulfonate/bicarbonate-binding protein							1										
JCVIGM_149307_20110307		nitrite/sulfite reductase, 4Fe-4S iron-sulfur cluster-binding domain protein		2															
JCVIGM_243460_20110307		nitrogen regulation protein				1													
JCVIGM_074550_20110307		nitroreductase family		10		3	3	1	2		1							1	
JCVIGM_142709_20110307		oligopeptide-binding protein OppA		1			1	1				1							
JCVIGM_287642_20110307		oligopeptide-binding protein OppA		3	5	1	1	1	2	6	1	1	1	2			1		
JCVIGM_291730_20110307		oligopeptide-binding protein OppA										1	3						
JCVIGM_320377_20110307		oligopeptide-binding protein OppA																	
JCVIGM_030295_20110307		ompA family protein												2	2			1	
JCVIGM_021098_20110307	pyrF	orotidine 5'-phosphate decarboxylase						1	1	1	1	1				1			
JCVIGM_069275_20110307		outer membrane protein		2		2		2											
JCVIGM_021757_20110307		oxaloacetate decarboxylase, alpha subunit							1		2								
JCVIGM_185159_20110307		oxidoreductase domain protein		1	1														
JCVIGM_227464_20110307		oxidoreductase domain protein		1		1			2									1	
JCVIGM_292816_20110307		oxidoreductase domain protein			1					1				1					
JCVIGM_098050_20110307		peptide/nickel ABC transporter substrate-binding protein		1															
JCVIGM_051204_20110307		peptidyl-prolyl cis-trans isomerase									1								4
JCVIGM_077390_20110307		peptidyl-prolyl cis-trans isomerase		3															
JCVIGM_089246_20110307		peptidyl-prolyl cis-trans isomerase		3			2								1				1
JCVIGM_359878_20110307		periplasmic binding protein/lacI transcriptional regulator			2														
JCVIGM_330291_20110307		periplasmic protein		7	8	5	8	6	3	1	3	4	13					1	
JCVIGM_352406_20110307		periplasmic protein		10	15	1	3	9	10	5	11	7	20	5	1	2	1	8	5
JCVIGM_063156_20110307	pta	phosphate acetyltransferase		1	2	1		1	2	1	1	1	2	1		1		1	1
JCVIGM_141522_20110307	pta	phosphate acetyltransferase		2	2	1	2	2	3	2	3	2	3	2				1	1
JCVIGM_008179_20110307	pckA	phosphoenolpyruvate carboxykinase (ATP)		11	4	5	12	11	16	3	17	6	14	5	2	2	1	2	2
JCVIGM_042402_20110307	pckA	phosphoenolpyruvate carboxykinase (ATP)		1					1			2							
JCVIGM_051034_20110307	pckA	phosphoenolpyruvate carboxykinase (ATP)		1	1				1		1		1			1			
JCVIGM_066626_20110307	pckA	phosphoenolpyruvate carboxykinase (ATP)		16		6	11	6	6	3	13	16		2	8	4	2	6	15
JCVIGM_070718_20110307	pckA	phosphoenolpyruvate carboxykinase (ATP)		84	18	10	14	15	20	28	24	29	20	9	1	6	3	15	4

JCVIGM_084152_20110307	pckA	phosphoenolpyruvate carboxykinase (ATP)		3															
JCVIGM_094757_20110307	pckA	phosphoenolpyruvate carboxykinase (ATP)		2							9								1
JCVIGM_108298_20110307	pckA	phosphoenolpyruvate carboxykinase (ATP)					1	1	2	1	4	1	2						
JCVIGM_111820_20110307	pckA	phosphoenolpyruvate carboxykinase (ATP)		3															
JCVIGM_116732_20110307		phosphoenolpyruvate carboxykinase (ATP)		3	6	3	5	7	5	6	5	5	7	3		2	1	3	
JCVIGM_126450_20110307	pckA	phosphoenolpyruvate carboxykinase (ATP)		3		2	2	2	1		2								3
JCVIGM_145745_20110307	pckA	phosphoenolpyruvate carboxykinase (ATP)		1				2					1						
JCVIGM_157605_20110307	pckA	phosphoenolpyruvate carboxykinase (ATP)																	
JCVIGM_168405_20110307	pckA	phosphoenolpyruvate carboxykinase (ATP)					1						2			2		1	
JCVIGM_170518_20110307	pckG	phosphoenolpyruvate carboxykinase (ATP)					2	1			1								
JCVIGM_186619_20110307	pckA	phosphoenolpyruvate carboxykinase (ATP)		4		9	6	4	3		3							1	2
JCVIGM_188966_20110307	pckA	phosphoenolpyruvate carboxykinase (ATP)		2	4			3	1	2	4	2	3	1		1		2	1
JCVIGM_200200_20110307	pckA	phosphoenolpyruvate carboxykinase (ATP)		1			1	2	3			2	2	1					
JCVIGM_212975_20110307		phosphoenolpyruvate carboxykinase (ATP)		21	21	12	37	37	40	5	44	7	78	18	22			13	6
JCVIGM_214913_20110307	pckA	phosphoenolpyruvate carboxykinase (ATP)					1		1		2		1						
JCVIGM_219457_20110307	pckA	phosphoenolpyruvate carboxykinase (ATP)		2		1	4	2			2	2			3			1	5
JCVIGM_255519_20110307	pckA	phosphoenolpyruvate carboxykinase (ATP)		1		1	4				2								2
JCVIGM_257324_20110307	pckA	phosphoenolpyruvate carboxykinase (ATP)		3		2	7	5	4		4	2	3		1				3
JCVIGM_259940_20110307	pckG	phosphoenolpyruvate carboxykinase (ATP)		1	1		2		1	2	2	1				1		1	
JCVIGM_297711_20110307	pckA	phosphoenolpyruvate carboxykinase (ATP)		2						1									1
JCVIGM_315164_20110307	pckA	phosphoenolpyruvate carboxykinase (ATP)		3	2	2	3	5	7		8	9	5	3					
JCVIGM_315431_20110307	pckA	phosphoenolpyruvate carboxykinase (ATP)		6	8	8	9	17	28		15	6	10						
JCVIGM_423536_20110307		phosphoenolpyruvate carboxykinase (ATP)									3								
JCVIGM_070299_20110307	pckA	phosphoenolpyruvate carboxykinase [ATP]		8		7	5	4	3	1	1	3			5			4	3
JCVIGM_127286_20110307	pckA	phosphoenolpyruvate carboxykinase [ATP]		4		2	5	4		1	5	2			2	1	1	2	2
JCVIGM_216386_20110307	manL	phosphoenolpyruvate-dependent sugar phosphotransferase system EIIAB, probably mannose specific						2			1								
JCVIGM_007589_20110307	pgk	phosphoglycerate kinase																	
JCVIGM_015380_20110307	pgk	phosphoglycerate kinase		2															
JCVIGM_027320_20110307	pgk	phosphoglycerate kinase		2	1	1	2	2	4	1	4	2		1		1		3	
JCVIGM_061465_20110307	pgk	phosphoglycerate kinase		1											2				
JCVIGM_079319_20110307	pgk	phosphoglycerate kinase						3	1	1	2	2			1	1	1		1

JCVIGM_095819_20110307	pgk	phosphoglycerate kinase		1								1							
JCVIGM_113192_20110307	pgk	phosphoglycerate kinase		12	5	4	5	18	19	2	11	9	8	3	2			10	1
JCVIGM_122299_20110307	pgk	phosphoglycerate kinase		1			1												1
JCVIGM_122514_20110307	pgk	phosphoglycerate kinase				1			1	1									
JCVIGM_152568_20110307	pgk	phosphoglycerate kinase					1											1	
JCVIGM_161540_20110307	pgk	phosphoglycerate kinase				1			1			1							
JCVIGM_195337_20110307	pgk	phosphoglycerate kinase		2		1	1												
JCVIGM_234057_20110307	pgk	phosphoglycerate kinase																2	
JCVIGM_239710_20110307	pgk	phosphoglycerate kinase			1	1		1	2	1	2								
JCVIGM_240738_20110307	pgk	phosphoglycerate kinase		2	2	1		1	1	2	2	3	2	2	1			2	1
JCVIGM_249205_20110307	pgk	phosphoglycerate kinase		1							1								
JCVIGM_260432_20110307	pgk	phosphoglycerate kinase												5	3	1		5	
JCVIGM_260954_20110307	pgk	phosphoglycerate kinase												2		1	1	3	
JCVIGM_313871_20110307	pgk	phosphoglycerate kinase				1	1	2	3		1								
JCVIGM_325494_20110307	pgk	phosphoglycerate kinase		4		1	2	3	4		2							2	
JCVIGM_329407_20110307	pgk	phosphoglycerate kinase		6	4	4	3	4	9	4	8	3	1	2		1	1	4	2
JCVIGM_040605_20110307	eno	phosphopyruvate hydratase		1			1												
JCVIGM_042947_20110307	eno	phosphopyruvate hydratase		3	2	1	1	1	2	2	2	2	2	2	1			1	
JCVIGM_045954_20110307	eno	phosphopyruvate hydratase		6		8	4	1	1	2	1	2		1	2			1	2
JCVIGM_064103_20110307	eno	phosphopyruvate hydratase				1													
JCVIGM_069773_20110307	eno	phosphopyruvate hydratase		1	1	1	1								1				
JCVIGM_117723_20110307	eno	phosphopyruvate hydratase		1			1			1	1	1	1	1	1				1
JCVIGM_189834_20110307	eno	phosphopyruvate hydratase		1			2	1			1								
JCVIGM_223379_20110307	eno	phosphopyruvate hydratase																	
JCVIGM_226543_20110307	eno	phosphopyruvate hydratase																	
JCVIGM_231374_20110307	eno	phosphopyruvate hydratase																	
JCVIGM_234175_20110307	eno	phosphopyruvate hydratase				1	1								1				1
JCVIGM_015906_20110307	purD	phosphoribosylamine--glycine ligase																	
JCVIGM_074211_20110307	purD	phosphoribosylamine--glycine ligase																	
JCVIGM_058619_20110307	purH	phosphoribosylaminoimidazolecarboxamide formyltransferase/IMP cyclohydrolase		7		1	1				1								

JCVIGM_089519_20110307	glgP1	phosphorylase		3															
JCVIGM_091853_20110307	glgP1	phosphorylase		1				1											
JCVIGM_250822_20110307		phosphorylase																3	
JCVIGM_020564_20110307	glgP	phosphorylase, glycogen/starch/alpha-glucan family					2	2											
JCVIGM_132125_20110307	serC	phosphoserine transaminase																	
JCVIGM_151788_20110307	serC	phosphoserine transaminase		4			14			3	4	2			1	2	1	4	2
JCVIGM_254038_20110307	serC	phosphoserine transaminase			1														
JCVIGM_000744_20110307	pnp	polyribonucleotide nucleotidyltransferase			1				2		1		1						
JCVIGM_049618_20110307	pnp	polyribonucleotide nucleotidyltransferase			1		1					2	3	1					
JCVIGM_156816_20110307	pnp	polyribonucleotide nucleotidyltransferase		3	7	3	5	4	3	1	6	8	11	2		2		3	
JCVIGM_177288_20110307	pnp	polyribonucleotide nucleotidyltransferase		1				2		1									
JCVIGM_284176_20110307	pnp	polyribonucleotide nucleotidyltransferase		1	3	4	3	3	6	1	3	1	5		1			2	
JCVIGM_301236_20110307	pnp	polyribonucleotide nucleotidyltransferase		1	1							1	1			1			
JCVIGM_384292_20110307	pnp	polyribonucleotide nucleotidyltransferase		1			1					1	1						
JCVIGM_415810_20110307	pnp	polyribonucleotide nucleotidyltransferase								1		1							
JCVIGM_149742_20110307		possible dehydrogenase			1	1		1	1				1	1					
JCVIGM_158818_20110307		predicted glycosylase						1										1	
JCVIGM_094962_20110307	araN	probable arabinose-binding protein																	
JCVIGM_443198_20110307	gcvPA	probable glycine dehydrogenase [decarboxylating] subunit 1																	2
JCVIGM_181243_20110307	tal	probable transaldolase						2											
JCVIGM_217573_20110307	tal	probable transaldolase		17	17	22								18				10	
JCVIGM_305907_20110307	tal	probable transaldolase		1		1	1	3	2	1	1		1	1				1	
JCVIGM_106798_20110307		propanediol utilization protein pduL		2															
JCVIGM_343769_20110307	pduD	propanediol utilization: dehydratase pduD			2														
JCVIGM_030557_20110307		propionyl-coA carboxylase beta chain		1	1							1	1						1
JCVIGM_070982_20110307		propionyl-coA carboxylase subunit beta		1		1	1											1	1
JCVIGM_076862_20110307		propionyl-coA carboxylase subunit beta			1	1						2	2		1				1
JCVIGM_212940_20110307		propionyl-coA carboxylase subunit beta			1							1	1		1				
JCVIGM_244599_20110307	deoD	purine nucleoside phosphorylase deoD-type												3	2	2	2	2	
JCVIGM_014093_20110307		purine nucleoside phosphorylase I, inosine and guanosine-specific		10		2	10	4	2	1	3	2	3	1	3			6	
JCVIGM_019193_20110307		purine nucleoside phosphorylase I, inosine and guanosine-specific		3			2	1	2	1	3	3	1	1	2				

JCVIGM_033253_20110307		putative 6-phosphofructokinase			2			1		1	1	2							
JCVIGM_128651_20110307		putative 6-phosphofructokinase		1	1		2	2	1		2	2	2	1		1	1	3	
JCVIGM_176874_20110307		putative 6-phosphofructokinase		1								1							
JCVIGM_149395_20110307		putative bacterial extracellular solute-binding protein						2	1		1								
JCVIGM_215021_20110307		putative bacterial extracellular solute-binding protein			3								1						
JCVIGM_268677_20110307		putative bacterial extracellular solute-binding protein			1	1		1		1								2	1
JCVIGM_273647_20110307		putative bacterial extracellular solute-binding protein																	
JCVIGM_286660_20110307		putative bacterial extracellular solute-binding protein		2	1						2							4	1
JCVIGM_297731_20110307		putative bacterial extracellular solute-binding protein						11	10	1	12	1							
JCVIGM_417971_20110307		putative bacterial extracellular solute-binding protein		1	2	1						1	2						
JCVIGM_237233_20110307	ytfQ	putative D-ribose transport system periplasmic binding component						1	1		1			1				1	2
JCVIGM_192868_20110307		putative endoribonuclease L-PSP		3															
JCVIGM_028951_20110307		putative esterase			1	1													
JCVIGM_101895_20110307		putative fumarate reductase flavoprotein subunit																	
JCVIGM_010624_20110307		putative immunoreactive antigen PG33			1								2						
JCVIGM_182718_20110307		putative IMP cyclohydrolase		7															
JCVIGM_071025_20110307		putative inositol-3-phosphate synthase		1															
JCVIGM_106169_20110307		putative inositol-3-phosphate synthase					1			1									
JCVIGM_240722_20110307		putative inositol-3-phosphate synthase							1	2	2	2			1				
JCVIGM_063721_20110307		putative lipoprotein								1									1
JCVIGM_094735_20110307		putative lipoprotein													1				
JCVIGM_105771_20110307		putative major outer membrane protein		1	3	1	1			1		1	2		1				1
JCVIGM_070279_20110307	spoVG	putative septation protein spoVG		1															
JCVIGM_178430_20110307		putative sugar ABC transporter, sugar-binding protein		2															
JCVIGM_194741_20110307		putative uncharacterized protein (fragment)		2	1	1	5	1	3	6	8	7							1
JCVIGM_000105_20110307	ppdK	pyruvate, phosphate dikinase				1						1							
JCVIGM_013898_20110307	ppdK	pyruvate, phosphate dikinase		1								1							1
JCVIGM_026505_20110307	ppdK	pyruvate, phosphate dikinase		3	2	1	1	2										3	
JCVIGM_036153_20110307	ppdK	pyruvate, phosphate dikinase		3														1	1
JCVIGM_066682_20110307	ppdK	pyruvate, phosphate dikinase		2														1	2
JCVIGM_109792_20110307	ppdK	pyruvate, phosphate dikinase		2														4	

JCVIGM_135990_20110307	ppdK	pyruvate, phosphate dikinase																	3
JCVIGM_173329_20110307		pyruvate, phosphate dikinase							1		1								
JCVIGM_213012_20110307	ppdK	pyruvate, phosphate dikinase		2															
JCVIGM_213014_20110307	ppdK	pyruvate, phosphate dikinase		2															
JCVIGM_277616_20110307		pyruvate, phosphate dikinase						1	1			3							
JCVIGM_335683_20110307		pyruvate/oxaloacetate carboxyltransferase		2			1	1	2	1	3	1	1					2	1
JCVIGM_198898_20110307		pyruvate-formate lyase		6	9	4	8	11	6	5	6	10	11	8		1	1	8	5
JCVIGM_255194_20110307		regulator of chromosome condensation, RCC1 (fragment)							1	1									
JCVIGM_036971_20110307	rplJ	ribosomal protein L10												2					
JCVIGM_042383_20110307	rplJ	ribosomal protein L10		1		1	2	3	2			2					1	1	
JCVIGM_071597_20110307	rplJ	ribosomal protein L10		3	4	1	1	4	6	1		3	4	3		3	5	2	1
JCVIGM_092732_20110307	rplJ	ribosomal protein L10		9	5	17	9	7	10	1	4	1	37	4			1	11	2
JCVIGM_131224_20110307	rplJ	ribosomal protein L10																	
JCVIGM_210248_20110307	rplJ	ribosomal protein L10		3	3	3	5	9	7	5	6	5	4	5	2	6	3	5	2
JCVIGM_226856_20110307	rplJ	ribosomal protein L10												4	4	3	4	6	
JCVIGM_082097_20110307	rplK	ribosomal protein L11					1	1	1		1		1	1		1			
JCVIGM_084768_20110307	rplK	ribosomal protein L11		1	1	1	1	1	1	4	1	1	3	3			1	3	
JCVIGM_136348_20110307	rplK	ribosomal protein L11			2								1					1	
JCVIGM_257392_20110307	rplK	ribosomal protein L11																	
JCVIGM_077076_20110307	rplK	ribosomal protein L11, N-terminal domain		1	2		1	5	4		2	4	11	1		1		3	
JCVIGM_097373_20110307	rplK	ribosomal protein L11, N-terminal domain																	
JCVIGM_045961_20110307	rplB	ribosomal protein L2		1	2	1	1		1	1			2						
JCVIGM_119829_20110307	rplB	ribosomal protein L2			1	2													
JCVIGM_193483_20110307	rplB	ribosomal protein L2					1					1							
JCVIGM_159582_20110307	rplW	ribosomal protein L23				1	1												
JCVIGM_006060_20110307	rplL	ribosomal protein L7/L12				1							2	2				1	
JCVIGM_054869_20110307	rplL	ribosomal protein L7/L12		2															
JCVIGM_156392_20110307	rplL	ribosomal protein L7/L12						2	1		1	1				1		1	
JCVIGM_159116_20110307	rplL	ribosomal protein L7/L12		5	3	3	1		1	4	3	3	1		1				1
JCVIGM_163260_20110307	rplL	ribosomal protein L7/L12					1												
JCVIGM_327331_20110307	rplL	ribosomal protein L7/L12							1		1								

JCVIGM_274754_20110307	rpsJ	ribosomal protein S10						1	1	1		1		1					
JCVIGM_021052_20110307	rpsG	ribosomal protein S7		1							1								
JCVIGM_089527_20110307	rpsH	ribosomal protein S8		2		1	2	2	2		3	1	1					1	
JCVIGM_106685_20110307	rpsH	ribosomal protein S8		4	1	1	2	2	3		2	2		1					
JCVIGM_116015_20110307	rpsH	ribosomal protein S8			1	1		2	1	2	1							1	
JCVIGM_158733_20110307	rpsH	ribosomal protein S8				1				1									
JCVIGM_202567_20110307	rpsH	ribosomal protein S8				1	1	3	1										
JCVIGM_216187_20110307	rpsH	ribosomal protein S8					1	1				1							
JCVIGM_114301_20110307	rpsI	ribosomal protein S9/S16			1	1													
JCVIGM_393651_20110307	rpoD	RNA polymerase sigma factor												1	3	1			
JCVIGM_229570_20110307		RNA polymerase sigma factor, sigma-70 family				14								3	24	11	4	1	
JCVIGM_007913_20110307		rubredoxin		3															
JCVIGM_057213_20110307		rubredoxin					1	2	1		2								
JCVIGM_006262_20110307		rubredoxin/rubrerythrin						1	1										
JCVIGM_020560_20110307		rubredoxin/rubrerythrin		61	62	22	46	58	68	33	76	101	223	33	15	12	9	50	18
JCVIGM_060719_20110307		rubredoxin/rubrerythrin		66	36	36	46	85	75	12	122	20	177	38	19	7	4	29	10
JCVIGM_085243_20110307		rubredoxin/rubrerythrin		2	2		1				2	2	3						
JCVIGM_248144_20110307		rubredoxin/rubrerythrin		3	6	2	5	5	1	3	4	5	13	4				3	
JCVIGM_401158_20110307		rubredoxin/rubrerythrin		3			2	1	4				3					1	
JCVIGM_415862_20110307		rubredoxin/rubrerythrin		1	2	2	5	4	4		1		1			1		1	
JCVIGM_054682_20110307	rbr	rubrerythrin		2			2	1		1	2	2		1	1	1	1	1	1
JCVIGM_073521_20110307		rubrerythrin		2			3					1							
JCVIGM_091745_20110307		rubrerythrin		3			1			1	1	3							
JCVIGM_119026_20110307		rubrerythrin		7	4	1	13	14	9	3	6	6	5	1				6	2
JCVIGM_127859_20110307		rubrerythrin		1															
JCVIGM_283228_20110307		rubrerythrin		2	3	1	2	2	3	3	2	1	2					1	
JCVIGM_027483_20110307	metK	S-adenosylmethionine synthetase, N-terminal domain							1										
JCVIGM_031546_20110307		seryl-tRNA synthetase N-terminal domain		3															
JCVIGM_093445_20110307		short-chain dehydrogenase/reductase SDR		1				1	1									1	1
JCVIGM_318607_20110307	ssb	single-strand binding protein							1	1									
JCVIGM_437080_20110307		single-stranded DNA-binding protein										1	1						

JCVIGM_166571_20110307		smr domain protein												1			1		
JCVIGM_117941_20110307	luxS	S-ribosylhomocysteine lyase		3					1									1	
JCVIGM_158998_20110307		SSU ribosomal protein S1P		2															
JCVIGM_034245_20110307		subtilase family					2		1			1	1						1
JCVIGM_125149_20110307		subtilase family domain protein		1		1	1				2						1		2
JCVIGM_007432_20110307		succinate CoA transferase		1			1				1				1				1
JCVIGM_079148_20110307		sugar ABC transporter substrate-binding protein		2	1	1	1	2	1	1	1	3	1	1				1	
JCVIGM_091077_20110307		sugar ABC transporter substrate-binding protein		9															
JCVIGM_166102_20110307		sugar ABC transporter substrate-binding protein		1			2				1	2							
JCVIGM_291360_20110307		sugar ABC transporter substrate-binding protein						1	1		1	1							
JCVIGM_400559_20110307		sugar ABC transporter substrate-binding protein		1			1	3	2	2	2	2							
JCVIGM_412377_20110307		sugar ABC transporter, periplasmic sugar-binding protein								1		1							
JCVIGM_371869_20110307		sugar ABC transporter, sugar-binding protein			1							1	2					1	
JCVIGM_014671_20110307		sugar phosphate isomerases/epimerases		1		2	1	2	2	2	1			1				1	
JCVIGM_084352_20110307		superoxide dismutase		1		1	5											2	1
JCVIGM_070610_20110307		susD family																	1
JCVIGM_102265_20110307		susD family		5	23	4	3	1	2		3	4	36	2				1	
JCVIGM_131087_20110307		tetratricopeptide repeat protein			1	1					1			1		1		2	
JCVIGM_391275_20110307	thlA3	thlA3		1	1								2						
JCVIGM_070609_20110307		tonB dependent receptor																	2
JCVIGM_081124_20110307		tonB-dependent receptor plug domain protein			2								2						
JCVIGM_134744_20110307		tonB-dependent receptor plug domain protein																	2
JCVIGM_052711_20110307		tonB-linked outer membrane protein, susC/ragA family			3								3						
JCVIGM_010680_20110307	fusA	translation elongation factor G							2										
JCVIGM_023291_20110307	fusA	translation elongation factor G		1							1								
JCVIGM_078720_20110307	fusA	translation elongation factor G		1						2	2								
JCVIGM_105810_20110307	fusA	translation elongation factor G																	2
JCVIGM_112572_20110307	fusA	translation elongation factor G		1						1	2	3	1						
JCVIGM_143844_20110307	fusA	translation elongation factor G		2	2	1	1	2	2	2	4	3	3	1	1	1		1	1
JCVIGM_097131_20110307	tsf	translation elongation factor Ts		1	1	1		1	1	1				1		1			
JCVIGM_183571_20110307	tsf	translation elongation factor Ts		1															
JCVIGM_277838_20110307	tsf	translation elongation factor Ts				1		1	1		1								

JCVIGM_431442_20110307	tsf	translation elongation factor Ts				1	1	1			1			2		1		1	1
JCVIGM_026960_20110307	tuf	translation elongation factor Tu		14		3	7	2	4	4	11	8		1	3	4	4	4	10
JCVIGM_029795_20110307	tuf	translation elongation factor Tu		9		8	9	10	8	4	10	7		2	8	2	1	4	8
JCVIGM_109601_20110307	tuf	translation elongation factor Tu		2					2										
JCVIGM_124820_20110307	tuf	translation elongation factor Tu		14	6	48	40	149	81	12	24	11	11	16	18	8	3	14	9
JCVIGM_157947_20110307	tuf	translation elongation factor Tu																	
JCVIGM_166526_20110307	tufB	translation elongation factor Tu																	
JCVIGM_185454_20110307	tuf	translation elongation factor Tu						1	1										
JCVIGM_196046_20110307	tuf	translation elongation factor Tu		3									1						
JCVIGM_196200_20110307	tuf	translation elongation factor Tu																	
JCVIGM_205201_20110307	tuf	translation elongation factor Tu		5															
JCVIGM_211136_20110307	tuf	translation elongation factor Tu						1	1										
JCVIGM_237573_20110307	tuf	translation elongation factor Tu		1		1													1
JCVIGM_249773_20110307	tuf	translation elongation factor Tu					1			1		1							
JCVIGM_250936_20110307	tufB	translation elongation factor Tu												5	11		4	3	
JCVIGM_300774_20110307	tuf	translation elongation factor Tu				1			1		1								
JCVIGM_368873_20110307	tuf	translation elongation factor Tu		2		1	2				1								2
JCVIGM_385355_20110307		transposase, IS111A/IS1328/IS1533																	
JCVIGM_364669_20110307		TRAP transporter solute receptor, TAXI family		1															
JCVIGM_001774_20110307	tpiA	triose-phosphate isomerase		3		1	2	2	1	2	2	2		1	1	1	1	1	2
JCVIGM_008332_20110307	tpiA	triose-phosphate isomerase		4	4	7	6	4	8	2	3	3	2	2	5	2	3	5	3
JCVIGM_011008_20110307	tpiA	triose-phosphate isomerase					2		1						1	1			3
JCVIGM_020800_20110307	tpiA	triose-phosphate isomerase												1					
JCVIGM_023613_20110307	tpiA	triose-phosphate isomerase		2			1	1	1	2		1		1	1			1	
JCVIGM_027321_20110307	tpiA	triose-phosphate isomerase		3	2	2	3	6	1	1	2	3	3						
JCVIGM_087250_20110307	tpiA	triose-phosphate isomerase		10		8	7	5	6	2	9	8		1	6			4	6
JCVIGM_115071_20110307	tpiA	triose-phosphate isomerase					1												
JCVIGM_135680_20110307	tpiA	triose-phosphate isomerase				1						1		1	2			1	
JCVIGM_261662_20110307	tpiA	triose-phosphate isomerase		1	1		1	4	2	4	1	1						1	
JCVIGM_302433_20110307	tpiA	triose-phosphate isomerase									1			1			1	7	

JCVIGM_337367_20110307	tpiA	triose-phosphate isomerase		2	1	3	1	5	1	2	3	2	1	4	3	3			2
JCVIGM_366477_20110307	tpiA	triose-phosphate isomerase					2	3	2		1								
JCVIGM_114128_20110307		tRNA methyl transferase									1	1							
JCVIGM_117733_20110307		two-component system sensor histidine kinase, with a response regulator receiver domain								1		1							
JCVIGM_373409_20110307		type III restriction protein		1															
JCVIGM_014974_20110307	galE	UDP-glucose 4-epimerase				1	1					2			1				
JCVIGM_179048_20110307	murE	UDP-N-acetylmuramoyl-L-alanyl-D-glutamate--2,6-diaminopimelate ligase												1		2			
JCVIGM_240853_20110307	uspA	universal stress protein A																	
JCVIGM_311901_20110307		UPF0210 protein DORLON_00661					1		1										
JCVIGM_044460_20110307		UTP--hexose-1-phosphate uridylyltransferase		4	1		2	2	3		3	1	3					4	
JCVIGM_148949_20110307	pepD	Xaa-His dipeptidase		2															
JCVIGM_195935_20110307		xylose ABC transporter substrate-binding protein																	
JCVIGM_086248_20110307	xylA	xylose isomerase				2	1											1	
JCVIGM_113241_20110307	xylA	xylose isomerase				3													1
JCVIGM_120909_20110307	xylA	xylose isomerase				1												1	1
JCVIGM_229108_20110307		YjeF N-terminal domain protein										1		1					

Table S1C: Total filter-passing S. Typhimurium protein identifications

					Biological replicate 1															
					CONTROL								INFECTED							
Reference	Gene symbol	Protein description	Few (<= 10) previous observations	DAY ->	-1	1	3	6	10	14	21	28	-1	1	3	6	10	14	21	28
ORF04041		2-dehydro-3-deoxy-6-phosphogalactonate aldolase																	1	
ORF04923		hypothetical protein																		
STM14_0013	dnaK	molecular chaperone DnaK														1	1		3	
STM14_0121	araA	L-arabinose isomerase					1		1		1			1			2			
STM14_0248	dgt	deoxyguanosinetriphosphate triphosphohydrolase																		
STM14_0266	yaeT	outer membrane protein assembly factor YaeT																1	2	
STM14_0287	metQ	DL-methionine transporter substrate-binding subunit														1				
STM14_0476		putative thiol-alkyl hydroperoxide reductase																		
STM14_0489	tsx	nucleoside channel														2	1	2	4	
STM14_0600	sfbA	putative ABC-type transport system ATPase component																		
STM14_0614	ybbY	putative purine permease YbbY																	1	
STM14_0674	nfnB	dihydropteridine reductase														1			1	
STM14_0689	fepG	iron-enterobactin transporter permease																		
STM14_0707	ahpC	alkyl hydroperoxide reductase subunit C														1	1	2	5	
STM14_0756		hypothetical protein																		
STM14_0810	fldA	flavodoxin FldA																		
STM14_0871	pal	peptidoglycan-associated outer membrane lipoprotein																		
STM14_0906		hypothetical protein	X							1										1
STM14_1084	ftsK	DNA translocase FtsK																		
STM14_1099	pflB	pyruvate formate lyase I			1	1		1					1	1		1				1
STM14_1105	serC	phosphoserine aminotransferase																		
STM14_1110	rpsA	30S ribosomal protein S1											1							
STM14_1176		minor tail protein	X																	
STM14_1214	ompA	outer membrane protein A														12	2	4	3	
STM14_1565	gapA	glyceraldehyde-3-phosphate dehydrogenase														7	2	3	3	
STM14_1577	gdhA	glutamate dehydrogenase			2				1								1			
STM14_1670	lppB	putative methyl-accepting chemotaxis protein													1	8	3	5	7	2
STM14_1671	lpp	murein lipoprotein														3	1	4	5	
STM14_1672	pykF	pyruvate kinase																	1	

STM14_1694		translocation machinery component	X																	
STM14_1729	sodB	superoxide dismutase														1		1	3	
STM14_1898	nmpC	putative outer membrane porin precursor														1				
STM14_2095	ompW	outer membrane protein W															1			
STM14_2113	adhE	bifunctional acetaldehyde-CoA/alcohol dehydrogenase																	2	
STM14_2133		hypothetical protein																	2	
STM14_2312	aspS	aspartyl-tRNA synthetase																	1	
STM14_2353	ftn	ferritin																		
STM14_2378	fliC	flagellin																		
STM14_2514	cbiJ	cobalt-precorrin-6x reductase																		
STM14_2531	pduE	propanediol dehydratase small subunit														1				
STM14_2534	pduJ	polyhedral body protein								1		1								
STM14_2538	pduN	polyhedral body protein	X																	
STM14_2584	rfbH	CDP-6-deoxy-D-xylo-4-hexulose-3-dehydrase																		
STM14_2625	yegN	multidrug efflux system subunit MdtB													1		1			
STM14_2719	yeiH	putative inner membrane protein	X																	
STM14_2817	glpQ	glycerophosphodiester phosphodiesterase															2			
STM14_2857	nuoN	NADH dehydrogenase subunit N	X																	
STM14_2949	pgtC	phosphoglycerate transport regulatory protein precurs	X																	
STM14_2990	ptsI	phosphoenolpyruvate-protein phosphotransferase			1					2			1							
STM14_2991	crr	glucose-specific PTS system component														1			1	
STM14_3014	eutL	putative carboxysome structural protein															1			
STM14_3023	eutM	putative detox protein												1						
STM14_3031	maeB	malic enzyme																		
STM14_3052	dapA	dihydrodipicolinate synthase																		
STM14_3091	yfgL	outer membrane protein assembly complex subunit YfgL															1	2	2	
STM14_3111	pepB	aminopeptidase B																	1	
STM14_3159	recO	DNA repair protein RecO	X																	
STM14_3261	clpB	protein disaggregation chaperone																		
STM14_3365		hypothetical protein												1					2	
STM14_3425	srlD	sorbitol-6-phosphate dehydrogenase																		
STM14_3557	eno	phosphopyruvate hydratase																	1	
STM14_3587	fucA	L-fuculose phosphate aldolase	X													2				
STM14_3590	fucI	L-fucose isomerase														15	3	3		

STM14_3592	fucU	putative L-fucose-binding protein	X																	
STM14_3687	gcvP	glycine dehydrogenase																	1	
STM14_3688	gcvH	glycine cleavage system protein H																		
STM14_3708	fba	fructose-bisphosphate aldolase														1			1	
STM14_3709	pgk	phosphoglycerate kinase														1	1	2	9	
STM14_3717	tktA	transketolase														1				
STM14_3733	metK	S-adenosylmethionine synthetase																		
STM14_3750	ansB	L-asparaginase II														4	2	2	3	
STM14_3767		putative acetyl-CoA hydrolase	X																	
STM14_3797	uxaC	uronate isomerase																		
STM14_3811	hybC	hydrogenase 2 large subunit																2	2	
STM14_3859	tolC	outer membrane channel protein																	1	
STM14_3923	tdcE	pyruvate formate-lyase 4/2-ketobutyrate formate-lyase																		
STM14_3926	tdcB	threonine dehydratase					1					1								
STM14_3930	garR	tartronate semialdehyde reductase														1		1	1	
STM14_3931	garL	alpha-dehydro-beta-deoxy-D-glucarate aldolase														1				
STM14_3939	gatY_1	tagatose-bisphosphate aldolase	X													3	2	1		
STM14_4030	nanA	N-acetylneuraminate lyase														1				
STM14_4033	sspA	stringent starvation protein A																		
STM14_4052	mdh	malate dehydrogenase																		
STM14_4129	rpsH	30S ribosomal protein S8																	1	
STM14_4136	rplP	50S ribosomal protein L16																1	2	
STM14_4142	rplD	50S ribosomal protein L4																		
STM14_4144	rpsJ	30S ribosomal protein S10																	2	
STM14_4149	tuf_1	elongation factor Tu											1		1	7		2	1	
STM14_4150	fusA	elongation factor G						3	2		2	3	3		1	2	1	1	4	
STM14_4188	bigA	putative surface-exposed virulence protein	X																	
STM14_4215	pckA	phosphoenolpyruvate carboxykinase			2		3	2	2	3	2	4	2						1	
STM14_4232	malP	maltodextrin phosphorylase																		
STM14_4320	uspA	universal stress protein A															1	1	1	
STM14_4328		putative L-asparaginase																		
STM14_4331		putative phosphosugar isomerase													1	1	1		2	
STM14_4443	mtlA	mannitol-specific enzyme IIABC component																	1	
STM14_4464	pmgI	phosphoglyceromutase																	2	

STM14_4620	dgoA	D-galactonate dehydratase	X																1	
STM14_4674	asnA	asparagine synthetase AsnA																		
STM14_4681	rbsB	D-ribose transporter subunit RbsB											1			1				
STM14_4698	ilvG	acetolactate synthase 2 catalytic subunit																		
STM14_4700	ilvE	branched-chain amino acid aminotransferase														1	1	1		
STM14_4701	ilvD	dihydroxy-acid dehydratase											1	1						
STM14_4713	trxA	thioredoxin														1	1		1	
STM14_4790	pepQ	proline dipeptidase																		
STM14_4806	dsbA	periplasmic protein disulfide isomerase I														1				
STM14_4852	fdoG	formate dehydrogenase alpha subunit	X																	
STM14_4902	yneA	putative sugar transport protein																		
STM14_4906	tpiA	triosephosphate isomerase					1	1	2	3	2		2			1		1	2	
STM14_4936	katG	hydroperoxidase														1	1		1	
STM14_4940	talC	fructose-6-phosphate aldolase																		
STM14_4986	rplK	50S ribosomal protein L11					1	1	1						1					
STM14_4988	rplJ	50S ribosomal protein L10														1		3	1	
STM14_4989	rplL	50S ribosomal protein L7/L12																	1	
STM14_4990	rpoB	DNA-directed RNA polymerase subunit beta																		2
STM14_5075	pgi	glucose-6-phosphate isomerase														2			1	
STM14_5080	yjbA	phosphate-starvation-inducible protein PsiE	X																	
STM14_5085	malE	maltose ABC transporter periplasmic protein														4	1	3	3	
STM14_5114	ssb	single-strand DNA-binding protein																		
STM14_5202	aspA	aspartate ammonia-lyase														2	1		6	
STM14_5207	groEL	chaperonin GroEL														3	1	1	1	
STM14_5223	frdA	fumarate reductase flavoprotein subunit																		
STM14_5244	hflK	FtsH protease regulator HflK																		
STM14_5253	yjfJ	putative phage shock protein A	X																	
STM14_5270	sgaE	L-ribulose-5-phosphate 4-epimerase	X																	
STM14_5301	ppa	inorganic pyrophosphatase														1			1	
STM14_5317		putative dehydrogenase	X																	
STM14_5327		putative endonuclease	X										1							
STM14_5358		ornithine carbamoyltransferase	X																	
STM14_5373	pepA	leucyl aminopeptidase																		
STM14_5419	iadA	isoaspartyl dipeptidase																	1	

STM14_5487	deoC	deoxyribose-phosphate aldolase															1			
STM14_5490	deoD	purine nucleoside phosphorylase																		
STM14_5502	yjjK	putative ABC transporter ATP-binding protein						1												
STM14_931	moaB	molybdopterin biosynthetic protein B														2				
STM14_966	dps	DNA starvation/stationary phase protection protein Dps																		

					Biological replicate 2-a															
					CONTROL								INFECTED							
Reference	Gene symbol	Protein description	Few (<= 10) previous observations	DAY ->	-1	1	3	6	10	14	21	28	-1	1	3	6	10	14	21	28
ORF04041		2-dehydro-3-deoxy-6-phosphogalactonate aldolase													4			2	1	
ORF04923		hypothetical protein																		
STM14_0013	dnaK	molecular chaperone DnaK			1			1					1			1			1	
STM14_0121	araA	L-arabinose isomerase																		
STM14_0248	dgt	deoxyguanosinetriphosphate triphosphohydrolase																		
STM14_0266	yaeT	outer membrane protein assembly factor YaeT														1				
STM14_0287	metQ	DL-methionine transporter substrate-binding subunit																1		
STM14_0476		putative thiol-alkyl hydroperoxide reductase													2				1	
STM14_0489	tsx	nucleoside channel														1				
STM14_0600	sfbA	putative ABC-type transport system ATPase component																		
STM14_0614	ybbY	putative purine permease YbbY																		
STM14_0674	nfnB	dihydropteridine reductase																		
STM14_0689	fepG	iron-enterobactin transporter permease										1								
STM14_0707	ahpC	alkyl hydroperoxide reductase subunit C													1	3	1	4	11	
STM14_0756		hypothetical protein																		
STM14_0810	fldA	flavodoxin FldA																		
STM14_0871	pal	peptidoglycan-associated outer membrane lipoprotein																		1
STM14_0906		hypothetical protein	**X**																	
STM14_1084	ftsK	DNA translocase FtsK																		
STM14_1099	pflB	pyruvate formate lyase I			1			1	1	2	1	1	2	1		4		1	3	
STM14_1105	serC	phosphoserine aminotransferase																	1	
STM14_1110	rpsA	30S ribosomal protein S1																		
STM14_1176		minor tail protein	**X**		1					1										
STM14_1214	ompA	outer membrane protein A													5	10		5	2	

STM14_1565	gapA	glyceraldehyde-3-phosphate dehydrogenase						1				1			8		5	3	8	3
STM14_1577	gdhA	glutamate dehydrogenase			2		1	1	1	1	1	1			1				1	
STM14_1670	lppB	putative methyl-accepting chemotaxis protein													1	3	2	2	2	
STM14_1671	lpp	murein lipoprotein													3	4	1	4	5	
STM14_1672	pykF	pyruvate kinase													2	1		1	2	
STM14_1694		translocation machinery component	X																	
STM14_1729	sodB	superoxide dismutase														1		1		
STM14_1898	nmpC	putative outer membrane porin precursor														1				
STM14_2095	ompW	outer membrane protein W														1				
STM14_2113	adhE	bifunctional acetaldehyde-CoA/alcohol dehydrogenase																		
STM14_2133		hypothetical protein																		
STM14_2312	aspS	aspartyl-tRNA synthetase																		
STM14_2353	ftn	ferritin													2			1	1	
STM14_2378	fliC	flagellin																		
STM14_2514	cbiJ	cobalt-precorrin-6x reductase																		
STM14_2531	pduE	propanediol dehydratase small subunit																		
STM14_2534	pduJ	polyhedral body protein			1			1		1		1	2	1						
STM14_2538	pduN	polyhedral body protein	X																	
STM14_2584	rfbH	CDP-6-deoxy-D-xylo-4-hexulose-3-dehydrase																	1	
STM14_2625	yegN	multidrug efflux system subunit MdtB																		
STM14_2719	yeiH	putative inner membrane protein	X													1	1			
STM14_2817	glpQ	glycerophosphodiester phosphodiesterase																	1	
STM14_2857	nuoN	NADH dehydrogenase subunit N	X																	
STM14_2949	pgtC	phosphoglycerate transport regulatory protein precurs	X																	
STM14_2990	ptsI	phosphoenolpyruvate-protein phosphotransferase																		
STM14_2991	crr	glucose-specific PTS system component																		
STM14_3014	eutL	putative carboxysome structural protein																		
STM14_3023	eutM	putative detox protein						1			1				1	1	1			
STM14_3031	maeB	malic enzyme																		
STM14_3052	dapA	dihydrodipicolinate synthase																		
STM14_3091	yfgL	outer membrane protein assembly complex subunit YfgL																		
STM14_3111	pepB	aminopeptidase B																		
STM14_3159	recO	DNA repair protein RecO	X			1														
STM14_3261	clpB	protein disaggregation chaperone																		

STM14_3365		hypothetical protein																		
STM14_3425	srlD	sorbitol-6-phosphate dehydrogenase																		
STM14_3557	eno	phosphopyruvate hydratase																		
STM14_3587	fucA	L-fuculose phosphate aldolase	**X**																	
STM14_3590	fucI	L-fucose isomerase			1	1							1	1	3	2			2	
STM14_3592	fucU	putative L-fucose-binding protein	**X**												1					
STM14_3687	gcvP	glycine dehydrogenase																		
STM14_3688	gcvH	glycine cleavage system protein H				1														
STM14_3708	fba	fructose-bisphosphate aldolase													3	1			1	
STM14_3709	pgk	phosphoglycerate kinase													16	5	2	3	15	1
STM14_3717	tktA	transketolase																		
STM14_3733	metK	S-adenosylmethionine synthetase							1	1										
STM14_3750	ansB	L-asparaginase II													2			1	2	
STM14_3767		putative acetyl-CoA hydrolase	**X**																	
STM14_3797	uxaC	uronate isomerase													3				1	
STM14_3811	hybC	hydrogenase 2 large subunit													1	1			1	
STM14_3859	tolC	outer membrane channel protein														1				
STM14_3923	tdcE	pyruvate formate-lyase 4/2-ketobutyrate formate-lyase																		
STM14_3926	tdcB	threonine dehydratase																		
STM14_3930	garR	tartronate semialdehyde reductase													4	1		1		
STM14_3931	garL	alpha-dehydro-beta-deoxy-D-glucarate aldolase													1			2		
STM14_3939	gatY_1	tagatose-bisphosphate aldolase	**X**												3					
STM14_4030	nanA	N-acetylneuraminate lyase													1					
STM14_4033	sspA	stringent starvation protein A											1							
STM14_4052	mdh	malate dehydrogenase													1				1	
STM14_4129	rpsH	30S ribosomal protein S8													1					
STM14_4136	rplP	50S ribosomal protein L16																		
STM14_4142	rplD	50S ribosomal protein L4																		
STM14_4144	rpsJ	30S ribosomal protein S10																		
STM14_4149	tuf_1	elongation factor Tu													9	6			3	1
STM14_4150	fusA	elongation factor G							1			1	3		3	3			2	
STM14_4188	bigA	putative surface-exposed virulence protein	**X**																	
STM14_4215	pckA	phosphoenolpyruvate carboxykinase			4		1	3	1	3		3	4		2				5	5
STM14_4232	malP	maltodextrin phosphorylase																		

STM14_4320	uspA	universal stress protein A																	1	
STM14_4328		putative L-asparaginase																1		
STM14_4331		putative phosphosugar isomerase													4	1			2	2
STM14_4443	mtlA	mannitol-specific enzyme IIABC component																	3	
STM14_4464	pmgI	phosphoglyceromutase													1				1	
STM14_4620	dgoA	D-galactonate dehydratase	X												10	1	1	3	10	
STM14_4674	asnA	asparagine synthetase AsnA																		
STM14_4681	rbsB	D-ribose transporter subunit RbsB													1					
STM14_4698	ilvG	acetolactate synthase 2 catalytic subunit																		
STM14_4700	ilvE	branched-chain amino acid aminotransferase																		
STM14_4701	ilvD	dihydroxy-acid dehydratase																		
STM14_4713	trxA	thioredoxin														1				
STM14_4790	pepQ	proline dipeptidase																	2	
STM14_4806	dsbA	periplasmic protein disulfide isomerase I																		
STM14_4852	fdoG	formate dehydrogenase alpha subunit	X																	
STM14_4902	yneA	putative sugar transport protein														1				
STM14_4906	tpiA	triosephosphate isomerase										1	1		1	1			3	
STM14_4936	katG	hydroperoxidase														2		3	4	
STM14_4940	talC	fructose-6-phosphate aldolase																		
STM14_4986	rplK	50S ribosomal protein L11					1		1	2									1	
STM14_4988	rplJ	50S ribosomal protein L10													3	2	2		3	
STM14_4989	rplL	50S ribosomal protein L7/L12																		
STM14_4990	rpoB	DNA-directed RNA polymerase subunit beta																		
STM14_5075	pgi	glucose-6-phosphate isomerase													2				1	
STM14_5080	yjbA	phosphate-starvation-inducible protein PsiE	X																	
STM14_5085	malE	maltose ABC transporter periplasmic protein													3	1	1	1	7	
STM14_5114	ssb	single-strand DNA-binding protein																		
STM14_5202	aspA	aspartate ammonia-lyase													2	2			3	
STM14_5207	groEL	chaperonin GroEL													3	1				
STM14_5223	frdA	fumarate reductase flavoprotein subunit																		
STM14_5244	hflK	FtsH protease regulator HflK																		
STM14_5253	yjfJ	putative phage shock protein A	X					1												
STM14_5270	sgaE	L-ribulose-5-phosphate 4-epimerase	X																	
STM14_5301	ppa	inorganic pyrophosphatase													1				1	

STM14_5317		putative dehydrogenase	X																	
STM14_5327		putative endonuclease	X						1	2					1				1	
STM14_5358		ornithine carbamoyltransferase	X																	
STM14_5373	pepA	leucyl aminopeptidase																		
STM14_5419	iadA	isoaspartyl dipeptidase																		1
STM14_5487	deoC	deoxyribose-phosphate aldolase													1					
STM14_5490	deoD	purine nucleoside phosphorylase																	1	
STM14_5502	yjjK	putative ABC transporter ATP-binding protein						1												
STM14_931	moaB	molybdopterin biosynthetic protein B																		
STM14_966	dps	DNA starvation/stationary phase protection protein Dps														4				

					**Biological replicate 2-b**															
					CONTROL								INFECTED							
Reference	Gene symbol	Protein description	Few (<= 10) previous observations	DAY ->	-1	1	3	6	10	14	21	28	-1	1	3	6	10	14	21	28
ORF04041		2-dehydro-3-deoxy-6-phosphogalactonate aldolase													3				4	
ORF04923		hypothetical protein				2	1	2	1	1		1	2	2					1	
STM14_0013	dnaK	molecular chaperone DnaK			1		1	1					1		3	6			1	1
STM14_0121	araA	L-arabinose isomerase			1	2	1	1	2	2	3	2	3	1	1		1			
STM14_0248	dgt	deoxyguanosinetriphosphate triphosphohydrolase							1	1										
STM14_0266	yaeT	outer membrane protein assembly factor YaeT																		
STM14_0287	metQ	DL-methionine transporter substrate-binding subunit														3				
STM14_0476		putative thiol-alkyl hydroperoxide reductase													2	2			6	
STM14_0489	tsx	nucleoside channel														10			1	
STM14_0600	sfbA	putative ABC-type transport system ATPase component														2	1	1		
STM14_0614	ybbY	putative purine permease YbbY										1								
STM14_0674	nfnB	dihydropteridine reductase																		
STM14_0689	fepG	iron-enterobactin transporter permease							1											
STM14_0707	ahpC	alkyl hydroperoxide reductase subunit C													5	31	9	8	44	
STM14_0756		hypothetical protein						1	1							1			1	
STM14_0810	fldA	flavodoxin FldA													1		1	1		
STM14_0871	pal	peptidoglycan-associated outer membrane lipoprotein														2	3	4	1	
STM14_0906		hypothetical protein	X									1				1		1		1
STM14_1084	ftsK	DNA translocase FtsK										2								

STM14_1099	pflB	pyruvate formate lyase I			3	3	1	2	5	2	2	3	2	3	4	10	2	5	7	2
STM14_1105	serC	phosphoserine aminotransferase										1								
STM14_1110	rpsA	30S ribosomal protein S1			3															
STM14_1176		minor tail protein	X																	
STM14_1214	ompA	outer membrane protein A													14	29	7	3	11	
STM14_1565	gapA	glyceraldehyde-3-phosphate dehydrogenase			1		1	3			1	1	2		19	8	26	20	36	2
STM14_1577	gdhA	glutamate dehydrogenase			6	4	3	4	5	2	2	4	4	3	4	2	3	1	4	5
STM14_1670	lppB	putative methyl-accepting chemotaxis protein													3	21	5	5	7	
STM14_1671	lpp	murein lipoprotein													2	16	4	4	5	
STM14_1672	pykF	pyruvate kinase													3	5		2	3	
STM14_1694		translocation machinery component	X						2									1		
STM14_1729	sodB	superoxide dismutase													2	1	2	2	1	
STM14_1898	nmpC	putative outer membrane porin precursor																		
STM14_2095	ompW	outer membrane protein W													1	3	1		2	
STM14_2113	adhE	bifunctional acetaldehyde-CoA/alcohol dehydrogenase																		
STM14_2133		hypothetical protein																		
STM14_2312	aspS	aspartyl-tRNA synthetase														2			4	
STM14_2353	ftn	ferritin													2	2		1	4	
STM14_2378	fliC	flagellin			1											2		1		1
STM14_2514	cbiJ	cobalt-precorrin-6x reductase				1								1	1					
STM14_2531	pduE	propanediol dehydratase small subunit				2														
STM14_2534	pduJ	polyhedral body protein			4	3	1	4	5	3	3	5	9	5	1				4	2
STM14_2538	pduN	polyhedral body protein	X		1					2								2		
STM14_2584	rfbH	CDP-6-deoxy-D-xylo-4-hexulose-3-dehydrase															1	1	1	
STM14_2625	yegN	multidrug efflux system subunit MdtB																		
STM14_2719	yeiH	putative inner membrane protein	X																	
STM14_2817	glpQ	glycerophosphodiester phosphodiesterase																		
STM14_2857	nuoN	NADH dehydrogenase subunit N	X						1										1	
STM14_2949	pgtC	phosphoglycerate transport regulatory protein precurs	X							2		1								
STM14_2990	ptsI	phosphoenolpyruvate-protein phosphotransferase														1				
STM14_2991	crr	glucose-specific PTS system component													1					
STM14_3014	eutL	putative carboxysome structural protein													1					
STM14_3023	eutM	putative detox protein			2	2	1	2	1		1	4	2	5	4	1	2	1	4	
STM14_3031	maeB	malic enzyme													1	1				

STM14_3052	dapA	dihydrodipicolinate synthase															1	1	1	
STM14_3091	yfgL	outer membrane protein assembly complex subunit YfgL																		
STM14_3111	pepB	aminopeptidase B															1	1		
STM14_3159	recO	DNA repair protein RecO	X			2	1				2		1		1	1	3	2		2
STM14_3261	clpB	protein disaggregation chaperone					1								2				2	1
STM14_3365		hypothetical protein																		
STM14_3425	srlD	sorbitol-6-phosphate dehydrogenase													3		1	1	2	
STM14_3557	eno	phosphopyruvate hydratase														1				
STM14_3587	fucA	L-fuculose phosphate aldolase	X																	
STM14_3590	fucI	L-fucose isomerase			1	3			1	1	1	1	1	5	8	6	8	5	11	
STM14_3592	fucU	putative L-fucose-binding protein	X												1	1				
STM14_3687	gcvP	glycine dehydrogenase															1	1	1	
STM14_3688	gcvH	glycine cleavage system protein H							1											
STM14_3708	fba	fructose-bisphosphate aldolase													5	2		2	4	
STM14_3709	pgk	phosphoglycerate kinase			5	4									29	12	9	7	40	
STM14_3717	tktA	transketolase						1		1										
STM14_3733	metK	S-adenosylmethionine synthetase							3	1		1	1				1			
STM14_3750	ansB	L-asparaginase II													3	2	2	3	7	
STM14_3767		putative acetyl-CoA hydrolase	X		1			1	1				1		1					
STM14_3797	uxaC	uronate isomerase													2					
STM14_3811	hybC	hydrogenase 2 large subunit													2	3	1	2	2	
STM14_3859	tolC	outer membrane channel protein														1				
STM14_3923	tdcE	pyruvate formate-lyase 4/2-ketobutyrate formate-lyase						1		1						1		1		
STM14_3926	tdcB	threonine dehydratase																		
STM14_3930	garR	tartronate semialdehyde reductase													9		1	2	3	
STM14_3931	garL	alpha-dehydro-beta-deoxy-D-glucarate aldolase													5		2	5	1	
STM14_3939	gatY_1	tagatose-bisphosphate aldolase	X												13				1	
STM14_4030	nanA	N-acetylneuraminate lyase																	2	
STM14_4033	sspA	stringent starvation protein A			2			1	1			1			1		2			1
STM14_4052	mdh	malate dehydrogenase													2	5	1	2	5	
STM14_4129	rpsH	30S ribosomal protein S8															1	1	2	
STM14_4136	rplP	50S ribosomal protein L16																		
STM14_4142	rplD	50S ribosomal protein L4			2	1														
STM14_4144	rpsJ	30S ribosomal protein S10																		

STM14_4149	tuf_1	elongation factor Tu													14	49		9	9	
STM14_4150	fusA	elongation factor G			3	1		1		4	1	4	2	2	4	8	2	2	5	
STM14_4188	bigA	putative surface-exposed virulence protein	X		1					1										
STM14_4215	pckA	phosphoenolpyruvate carboxykinase			6	2	2	8	3	5	4	5	6	4	9	7	3	5	12	3
STM14_4232	malP	maltodextrin phosphorylase			2			2	2											
STM14_4320	uspA	universal stress protein A																		
STM14_4328		putative L-asparaginase													3			2	1	
STM14_4331		putative phosphosugar isomerase													7	2			5	
STM14_4443	mtlA	mannitol-specific enzyme IIABC component													2		3	1	7	
STM14_4464	pmgI	phosphoglyceromutase													3	1		1	1	
STM14_4620	dgoA	D-galactonate dehydratase	X												27	7	6	9	27	
STM14_4674	asnA	asparagine synthetase AsnA					1			2				1						
STM14_4681	rbsB	D-ribose transporter subunit RbsB													1					
STM14_4698	ilvG	acetolactate synthase 2 catalytic subunit											1		2					
STM14_4700	ilvE	branched-chain amino acid aminotransferase																1		
STM14_4701	ilvD	dihydroxy-acid dehydratase																		
STM14_4713	trxA	thioredoxin													1		1	2		
STM14_4790	pepQ	proline dipeptidase																	1	
STM14_4806	dsbA	periplasmic protein disulfide isomerase I															1			
STM14_4852	fdoG	formate dehydrogenase alpha subunit	X													4				
STM14_4902	yneA	putative sugar transport protein														2				
STM14_4906	tpiA	triosephosphate isomerase			3		2	3		2	2	3	3		3	4	2	3	11	3
STM14_4936	katG	hydroperoxidase													2	9	2	6	12	
STM14_4940	talC	fructose-6-phosphate aldolase													1	1			1	
STM14_4986	rplK	50S ribosomal protein L11			1	1	3	4	3	3	4	2	2	6	5		3	2	3	
STM14_4988	rplJ	50S ribosomal protein L10													4	4	3	4	6	
STM14_4989	rplL	50S ribosomal protein L7/L12														1			94	
STM14_4990	rpoB	DNA-directed RNA polymerase subunit beta																		
STM14_5075	pgi	glucose-6-phosphate isomerase													2	2	1	2	6	
STM14_5080	yjbA	phosphate-starvation-inducible protein PsiE	X		1			2		1		1		2						
STM14_5085	malE	maltose ABC transporter periplasmic protein										1			7	9	6	4	12	
STM14_5114	ssb	single-strand DNA-binding protein													1				1	
STM14_5202	aspA	aspartate ammonia-lyase													8	19	9	10	13	
STM14_5207	groEL	chaperonin GroEL			1		1								4	10	5	3		

STM14_5223	frdA	fumarate reductase flavoprotein subunit													1			1		
STM14_5244	hflK	FtsH protease regulator HflK					1			1										
STM14_5253	yjfJ	putative phage shock protein A	X							1										
STM14_5270	sgaE	L-ribulose-5-phosphate 4-epimerase	X			1					1									
STM14_5301	ppa	inorganic pyrophosphatase													3	2	1	1	3	
STM14_5317		putative dehydrogenase	X													3		1		
STM14_5327		putative endonuclease	X		1		1	1	1	1								1	3	
STM14_5358		ornithine carbamoyltransferase	X		1		1	1	3	1	1	2	2	1		2				
STM14_5373	pepA	leucyl aminopeptidase											1			1				
STM14_5419	iadA	isoaspartyl dipeptidase													3	2			6	
STM14_5487	deoC	deoxyribose-phosphate aldolase													4	8	1	1	3	
STM14_5490	deoD	purine nucleoside phosphorylase													3	2	2	2	2	
STM14_5502	yjjK	putative ABC transporter ATP-binding protein																		1
STM14_931	moaB	molybdopterin biosynthetic protein B																		
STM14_966	dps	DNA starvation/stationary phase protection protein Dps														12	2	2		
