# Supplementary material for: A Multi-Omic View of Host-Pathogen-Commensal Interplay in Salmonella-Mediated Intestinal Infection
Source: PLoS One. 2013 Jun 26;8(6):e67155. doi: 10.1371/journal.pone.0067155 (PMC3694140; doi:10.1371/journal.pone.0067155)
Supplement: Table S2 — Selected mouse proteins identified during infection. The immune response to gastrointestinal S. Typhimurium infection in humans is characterized by an inflammatory response and neutrophil activation. Proteomic results support a similar host response in 129/SvJ mice, which includes (but is not limited to) expression of neutrophil-related proteins, cell surface proteins, and inflammatory molecules. Selected protein names and known/proposed functions are shown. Asterisks highlight host proteins that have not, to our knowledge, been previously linked to the anti-Salmonella immune response. (PDF) [file pone.0067155.s009.pdf]

Table S2: Selected mouse proteins identified during infection

Reference	Protein Name	Protein Function(s) where known	Citation
Neutrophil-related proteins			
NP_034954.2	myeloperoxidase precursor	Enzymatic component of neutrophil azurophilic granules	(7)
NP_079705.2	leukocyte elastase inhibitor	Regulator of neutrophil proteases	
NP_032720.2	neutrophilic granule protein (proteinase-3)	Antimicrobial	(12)
NP_035308.2	myeloblastin precursor*	Azurophilic neutrophil granule protein released during activation	(11)
NP_081138.2	CD177 antigen precursor*	Neutrophil-specific cell surface glycoprotein	(10)
NP_032430.2	integrin beta-2 (CD18)	Mediates transendothelial migration of leukocytes during inflammation	(10)
Cell surface proteins			
NP_034508.2	histocompatibility 2, class II antigen A, α precursor	Antigen presentation	(6)
NP_996988.2	histocompatibility 2, class II antigen A, β 1 precursor	Antigen presentation	(6)
NP_034204.1	dipeptidyl peptidase 4 isoform 1 (CD26)*	T-cell activation	
NP_067623.1	cell surface A33 antigen precursor*	Intestinal cell surface antigen of the immunoglobulin family	(5)
NP_666122.1	tetraspanin 8*	Cell surface protein that complexes with integrins	
Inflammatory molecules			
NP_035389.1	regenerating islet-derived protein 3-α precursor*		
NP_035166.1	regenerating islet-derived protein 3-β precursor	Antimicrobial	(13)
NP_035390.1	regenerating islet-derived protein 3-γ precursor	Antimicrobial	(1)
NP_033908.2	complement C3	Initiator of the complement cascade	(9)
NP_033140.1	protein S100-A9	Heterodimer = calprotectin; surface glycoprotein reduces bacterial invasion and epithelial cell binding; abundant PMN cytoplasmic protein	(4)
NP_038678.1	protein S100-A8		
NP_033068.1	lithostathine-1 precursor*	Bacterial aggregation, pancreatic stone formation	(3)
NP_033069.1	lithostathine-2 precursor*		
NP_031795.2	deleted in malignant brain tumors 1 protein*	Secreted scavenger receptor, binds bacterial cells, immune modulation	(8)
NP_035448.2	serum amyloid P-component precursor*	Binds LPS, impacts activation of complement (classical pathway)	(2)

*Not previously characterized as part of the immune response against *S*. Typhimurium.

1. **Cash, H. L., C. V. Whitham, C. L. Behrendt, and L. V. Hooper.** 2006. Symbiotic bacteria direct expression of an intestinal bactericidal lectin. Science **313:**1126-1130.
2. **de Haas, C. J., E. M. van Leeuwen, T. van Bommel, J. Verhoef, K. P. van Kessel, and J. A. van Strijp.** 2000. Serum amyloid P component bound to gram-negative bacteria prevents lipopolysaccharide-mediated classical pathway complement activation. Infect Immun **68:**1753-1759.
3. **Jin, C. X., T. Hayakawa, S. B. Ko, H. Ishiguro, and M. Kitagawa.** 2011. Pancreatic stone protein/regenerating protein family in pancreatic and gastrointestinal diseases. Intern Med **50:**1507-1516.

4. **Johne, B., M. K. Fagerhol, T. Lyberg, H. Prydz, P. Brandtzaeg, C. F. Naess-Andresen, and I. Dale.** 1997. Functional and clinical aspects of the myelomonocyte protein calprotectin. Mol Pathol **50:**113-123.
5. **Joosten, C. E., L. S. Cohen, G. Ritter, C. A. Batt, and M. L. Shuler.** 2004. Glycosylation profiles of the human colorectal cancer A33 antigen naturally expressed in the human colorectal cancer cell line SW1222 and expressed as recombinant protein in different insect cell lines. Biotechnol Prog **20:**1273-1279.
6. **Kaufmann, S. H., and U. E. Schaible.** 2005. Antigen presentation and recognition in bacterial infections. Curr Opin Immunol **17:**79-87.
7. **Klebanoff, S. J.** 2005. Myeloperoxidase: friend and foe. J Leukoc Biol **77:**598-625.
8. **Ligtenberg, A. J., E. C. Veerman, A. V. Nieuw Amerongen, and J. Mollenhauer.** 2007. Salivary agglutinin/glycoprotein-340/DMBT1: a single molecule with variable composition and with different functions in infection, inflammation and cancer. Biol Chem **388:**1275-1289.
9. **Makela, P. H., M. Hovi, H. Saxen, M. Valtonen, and V. Valtonen.** 1988. Salmonella, complement and mouse macrophages. Immunol Lett **19:**217-222.
10. **Muschter, S., T. Berthold, and A. Greinacher.** 2011. Developments in the definition and clinical impact of human neutrophil antigens. Curr Opin Hematol **18:**452-460.
11. **Rao, N. V., N. G. Wehner, B. C. Marshall, W. R. Gray, B. H. Gray, and J. R. Hoidal.** 1991. Characterization of proteinase-3 (PR-3), a neutrophil serine proteinase. Structural and functional properties. J Biol Chem **266:**9540-9548.
12. **Scocchi, M., B. Skerlavaj, D. Romeo, and R. Gennaro.** 1992. Proteolytic cleavage by neutrophil elastase converts inactive storage proforms to antibacterial bactenecins. Eur J Biochem **209:**589-595.
13. **Stelter, C., R. Kappeli, C. Konig, A. Krah, W. D. Hardt, B. Stecher, and D. Bumann.** 2011. Salmonella-induced mucosal lectin RegIIIbeta kills competing gut microbiota. PLoS One **6:**e20749.
