# Supplementary material for: A Multi-Omic View of Host-Pathogen-Commensal Interplay in Salmonella-Mediated Intestinal Infection
Source: PLoS One. 2013 Jun 26;8(6):e67155. doi: 10.1371/journal.pone.0067155 (PMC3694140; doi:10.1371/journal.pone.0067155)
Supplement: Table S3 — Proteins determined to be statistically significantly different in control and infected mice. (PDF) [file pone.0067155.s010.pdf]

Table S3. Proteins found to be statistically significantly different in control and infected mice.

Commensal Proteins	
Protein	Protein description
JCVIGM_226856_20110307	ribosomal protein L10
JCVIGM_229762_20110307	major outer membrane lipoprotein 1
JCVIGM_210946_20110307	hypothetical protein
JCVIGM_022560_20110307	hypothetical protein
JCVIGM_260954_20110307	phosphoglycerate kinase
JCVIGM_118473_20110307	formate--tetrahydrofolate ligase
JCVIGM_060468_20110307	hypothetical protein
JCVIGM_307765_20110307	hypothetical protein
JCVIGM_369484_20110307	hypothetical protein
JCVIGM_360038_20110307	2-hydroxy-3-oxopropionate reductase
JCVIGM_108298_20110307	phosphoenolpyruvate carboxykinase (ATP)
JCVIGM_260432_20110307	phosphoglycerate kinase
JCVIGM_360253_20110307	glyceraldehyde 3-phosphate dehydrogenase, C-terminal domain
JCVIGM_037673_20110307	3-hydroxybutyryl-coA dehydrogenase
JCVIGM_055645_20110307	carbohydrate ABC transporter substrate-binding protein, CUT1 family
JCVIGM_118383_20110307	hypothetical protein
JCVIGM_181280_20110307	B12 binding domain
JCVIGM_054434_20110307	arabinogalactan endo-1,4-beta-galactosidase
JCVIGM_045961_20110307	ribosomal protein L2
JCVIGM_104408_20110307	hypothetical protein
JCVIGM_291424_20110307	hypothetical protein
JCVIGM_062252_20110307	ketose-bisphosphate aldolase, class II
JCVIGM_213960_20110307	chaperone protein dnaK
JCVIGM_087474_20110307	fructose-1,6-bisphosphate aldolase, class II
JCVIGM_045263_20110307	extracellular solute-binding protein family 1
JCVIGM_194741_20110307	putative uncharacterized protein (fragment)
JCVIGM_244599_20110307	purine nucleoside phosphorylase deoD-type
JCVIGM_393651_20110307	RNA polymerase sigma factor
JCVIGM_184954_20110307	integral membrane sensor signal transduction histidine kinase
JCVIGM_057213_20110307	rubredoxin
JCVIGM_123964_20110307	formate--tetrahydrofolate ligase
JCVIGM_027321_20110307	triose-phosphate isomerase
JCVIGM_188817_20110307	monosaccharide ABC transporter substrate-binding protein, CUT2 family
JCVIGM_079110_20110307	malate dehydrogenase (oxaloacetate-decarboxylating)
JCVIGM_083913_20110307	electron transfer flavoprotein, beta subunit
JCVIGM_294702_20110307	glycerol dehydratase large subunit
JCVIGM_083011_20110307	glyceraldehyde 3-phosphate dehydrogenase, C-terminal domain
JCVIGM_197130_20110307	ABC-type xylose transport system, periplasmic component
JCVIGM_177955_20110307	flagellin fliC2

JCVIGM_036678_20110307	hypothetical protein
JCVIGM_189834_20110307	phosphopyruvate hydratase
JCVIGM_030295_20110307	ompA family protein
JCVIGM_168776_20110307	L-arabinose isomerase
JCVIGM_101296_20110307	acetyl-coA C-acetyltransferase
JCVIGM_189194_20110307	glyceraldehyde 3-phosphate dehydrogenase, C-terminal domain
JCVIGM_081547_20110307	glutamate dehydrogenase, NADP-specific

**Salmonella proteins**	
Dataset	Description
STM14_4988	rplJ 50S ribosomal protein L10
STM14_3750	ansB L-asparaginase II
STM14_5085	malE maltose ABC transporter periplasmic protein
STM14_3811	hybC hydrogenase 2 large subunit
STM14_1671	lpp murein lipoprotein
STM14_1729	sodB superoxide dismutase
STM14_1670	lppB putative methyl-accepting chemotaxis protein
STM14_1214	ompA outer membrane protein A
STM14_5301	ppa inorganic pyrophosphatase
STM14_1672	pykF pyruvate kinase
STM14_3709	pgk phosphoglycerate kinase
STM14_5202	aspA aspartate ammonia-lyase
STM14_3708	fba fructose-bisphosphate aldolase
STM14_4713	trxA thioredoxin
STM14_4331	putative phosphosugar isomerase
STM14_4936	katG hydroperoxidase
STM14_5075	pgi glucose-6-phosphate isomerase
STM14_3590	fucI L-fucose isomerase
STM14_1565	gapA glyceraldehyde-3-phosphate dehydrogenase
STM14_4620	dgoA 2-oxo-3-deoxygalactonate 6-phosphate aldolase/galacto
STM14_4464	pmgI phosphoglyceromutase
STM14_5490	deoD purine nucleoside phosphorylase
STM14_2353	ftn ferritin
STM14_3930	garR tartronate semialdehyde reductase
STM14_0707	ahpC alkyl hydroperoxide reductase subunit C
ORF04041	2-dehydro-3-deoxy-6-phosphogalactonate aldolase (6-phospho-2-dehydro-3-deoxygalactonate aldola
STM14_2095	ompW outer membrane protein W
STM14_3931	garL alpha-dehydro-beta-deoxy-D-glucarate aldolase
STM14_4052	mdh malate dehydrogenase
STM14_4129	rpsH 30S ribosomal protein S8
STM14_5207	groEL chaperonin GroEL
STM14_4149	tuf_1 elongation factor Tu; Duplicate proteins: STM14_4982
STM14_2584	rfbH CDP-6-deoxy-D-xylo-4-hexulose-3-dehydrase

STM14_3687	gcvP glycine dehydrogenase
STM14_4320	uspA universal stress protein A
STM14_4700	ilvE branched-chain amino acid aminotransferase
STM14_4443	mtlA mannitol-specific enzyme IIABC component
STM14_5487	deoC deoxyribose-phosphate aldolase
STM14_0489	tsx nucleoside channel
STM14_0476	putative thiol-alkyl hydroperoxide reductase
STM14_0871	pal peptidoglycan-associated outer membrane lipoprotein
STM14_3425	srlD sorbitol-6-phosphate dehydrogenase
STM14_4328	putative L-asparaginase

**Mouse proteins**	
Dataset	Description
gi|149248895	PREDICTED: similar to Glyceraldehyde-3-phosphate dehydrogenase (GAPDH) isoform 1 [Mus musculus] ref|XP_001475565.1
gi|149251776	PREDICTED: similar to Nucleophosmin (NPM) (Nucleolar phosphoprotein B23) (Numatrin) (Nucleolar protein NO38) [Mus musculus] ref|XP_001476579.1
gi|94378251	PREDICTED: similar to histone H4 [Mus musculus] ref|XP_981474.1
gi|160333652	protease, serine, 32 [Mus musculus] ref|NP_081496.2
gi|6678097	serpin B6 isoform b [Mus musculus] ref|NP_033280.1
gi|31982171	murinoglobulin-1 precursor [Mus musculus] ref|NP_032671.2
gi|87239967	deleted in malignant brain tumors 1 protein [Mus musculus] ref|NP_031795.2
gi|160333869	pancreatic lipase-related protein 2 [Mus musculus] ref|NP_035258.2
gi|6754980	regenerating islet-derived protein 3-beta precursor [Mus musculus] ref|NP_035166.1
gi|226958497	serum amyloid P-component precursor [Mus musculus] ref|NP_035448.2
gi|6755308	regenerating islet-derived protein 3-alpha precursor [Mus musculus] ref|NP_035389.1
gi|254281348	chitinase-3-like protein 3 precursor [Mus musculus] ref|NP_034022.2
gi|20330802	serotransferrin precursor [Mus musculus] ref|NP_598738.1
gi|33563252	fibrinogen, alpha polypeptide isoform 2 [Mus musculus] ref|NP_034326.1
gi|147901863	meprin A subunit beta precursor [Mus musculus] ref|NP_032612.2
gi|225735649	sulfated glycoprotein 1 isoform A preproprotein [Mus musculus] ref|NP_001139592.1
gi|6681297	chymotrypsin-like elastase family member 2A precursor [Mus musculus] ref|NP_031945.1
gi|11037800	cell surface A33 antigen precursor [Mus musculus] ref|NP_067623.1
gi|6755310	regenerating islet-derived protein 3-gamma precursor [Mus musculus] ref|NP_035390.1
gi|31981716	histocompatibility 2, class II antigen A, alpha precursor [Mus musculus] ref|NP_034508.2
gi|6677703	lithostathine-1 precursor [Mus musculus] ref|NP_033068.1
gi|6677705	lithostathine-2 precursor [Mus musculus] ref|NP_033069.1
